# Addition of Lithium Silylamides to 1,2-Dicyanobenzene: Isoindoline-1,3-diimine Derivatives Investigated by NMR/XRD/DFT Approach

**DOI:** 10.1021/acs.inorgchem.5c00573

**Published:** 2025-04-08

**Authors:** Stanislava Majerová, Tomáš Chlupatý, Maksim A. Samsonov, Josef Cvačka, Eliška Procházková, Aleš Růžička

**Affiliations:** † Department of General and Inorganic Chemistry, Faculty of Chemical Technology, University of Pardubice, Studentská 573, Pardubice 532 10, Czech Republic; ‡ Institute of Organic Chemistry and Biochemistry, Czech Academy of Sciences, Flemingovo nám. 2, Prague 160 00, Czech Republic

## Abstract

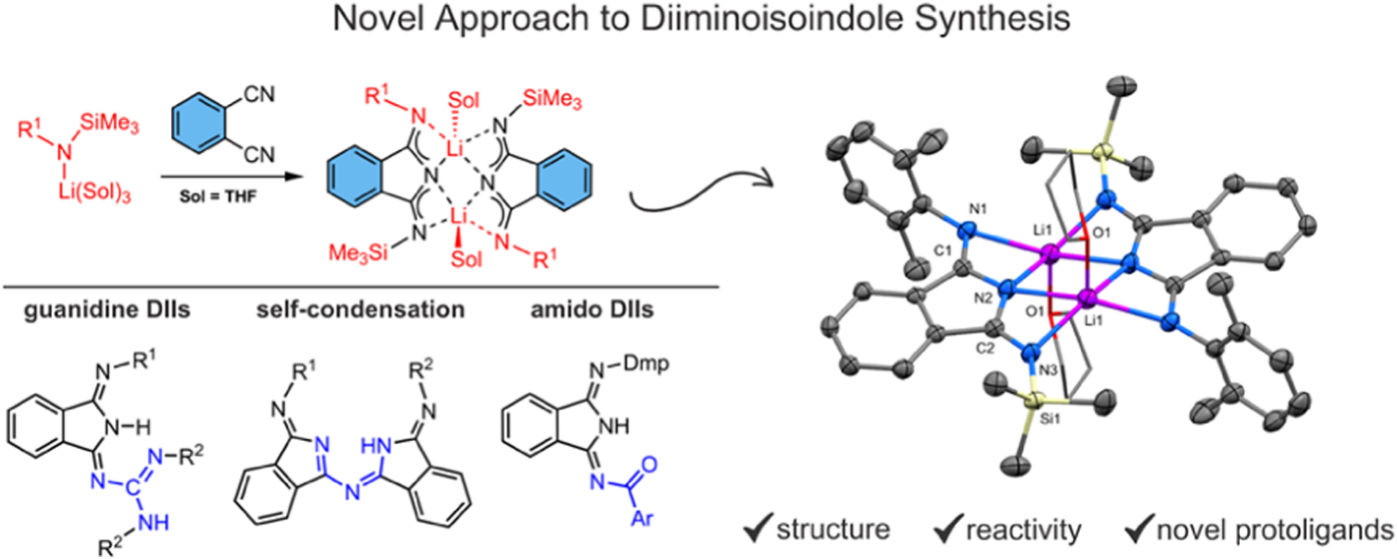

Phthalocyanines and their building blocks, isoindoline-1,3-diimines (diiminoisoindoles, DIIs), represent a structurally diverse class of compounds with the ability to make metal complexes and perform in various fields from medicine to photovoltaics and homogeneous catalysis. According to the present study, monosubstituted diiminoisoindoles, their higher homologues, and complexes can be effectively prepared by addition of silylated lithium amides to 1,2-dicyanobenzene followed by mild protonolysis or a condensation. An addition of DII to carbodiimides or reactions of lithiated DIIs with acyl chlorides give DII-guanidines and amido derivatives. The imino group of the amido derivatives is preferentially and quantitatively reduced by sodium borohydride. Dynamic behavior and structure of all studied classes of compounds were investigated from the stereochemical point of view—possible *E*/*Z*-isomerization and dimerization (DIIs and amido derivatives), tautomerism (guanidines), and stability both in solution and in solid state. The resonance-assisted hydrogen bonds are present in all species except reduced amides, predetermining them to be exceptional ligands in coordination chemistry.

## Abstract 2

Various diiminoisoindoles and its derivatives were prepared by reaction of 1,2-dicyanobenzene and silylated lithium amides. Follow-up reactivity such as protonolysis, condensation, addition, or C−N coupling was studied. *E*/*Z*-isomerism, tautomerism, and agglomeration of compounds were described in solution and solid state. Possibilities to use such compounds as ligands in coordination chemistry are documented by the presence of resonance-assisted hydrogen bonds and the formation of lithium complexes.

## Introduction

**Figure 1:**
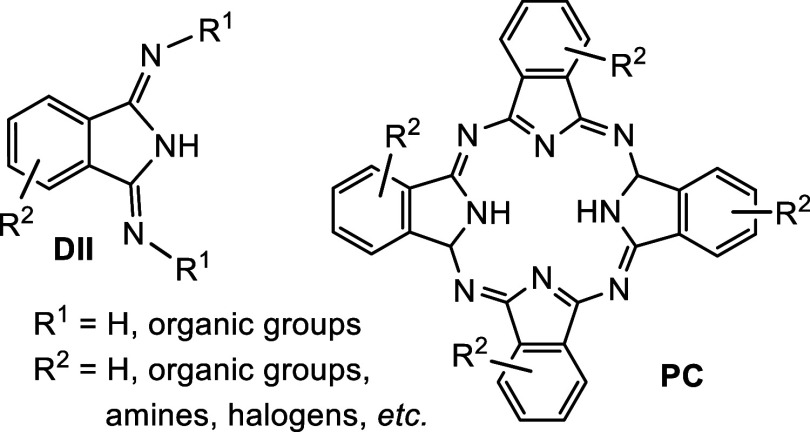
Structures of the DIIs and PCs.

Phthalocyanines (PC) are nitrogen-rich, planar, aromatic compounds with many possible substitutional patterns. Their versatility, thermal robustness, supramolecular assembly, and electronic structure are reflected in a wide range of applications such as dyes and pigments or in photoelectricity. Doubly deprotonated PCs are perfect macrocyclic ligands capable of accommodating almost all metals from the periodic table in a plethora of bonding motifs. Taking into account tunable electronic and optical properties, coupled with stability, some of these complexes were identified as invaluable species for organic electronics, catalysis, and medicinal chemistry.^1^ Virtual building blocks of PCs are undoubtedly the isoindoline-1,3-diimine (DII) derivatives, from which the synthesis of many PCs additionally starts (Figure 1).

Although the chemistry and applications of PCs could be seen as the major purpose of DIIs studies, they are also involved as construction parts in a plethora of materials. Biologically active species and their isoindole fragments can be found in a number of natural products^2^ and dyes.^3^ Concretely, they are used for the synthesis of phthalocyanine analogues such as hemiporphyrazines, hydroxybenzipthalocyanines, or biliazine;^4−7^ synthesis of fluorophores BOSHPY (BODIPY analogues);^8,9^ and design of anion receptors^10^ and electrocatalysts for oxygen evolution.^11^ Mentionable from biologically active compounds are C3a antagonists^12^ and antimalarial candidates.^13^

Various DIIs and vast majority of derivatives have been prepared from 1,3-diiminoisoindoline and primary amines, following the seminal work of Linstead, who was the first to describe this condensation reaction almost 70 years ago.^14,15^ In order to increase the yields of both symmetrical (bis-derivative) and dissymmetrical species, several modifications of that method appeared. Much later, Siegl and others addressed the same issue, finding that phthalonitrile adds anilines, specifically to bis-1,3-(arylimino)isoindoline, when reactions are catalyzed by anhydrous calcium chloride.^16−18^ They were followed by Ziegler, who reinvestigated the preparation of bis-1,3-(arylimino)isoindoles and bis-1,3-(alkylimino)isoindoles both in the solid state and in solution.^19^ All methods are characterized by long reaction times, significantly increased temperature, low to moderate yields, necessity of a catalyst, and challenging purification with necessary workup by recrystallization or flash chromatography.

Surprisingly, not many metal complexes containing DIIs and their derivatives (excluding PCs) have been prepared thus far. Majority of coordination compounds containing DII moiety are based on using 2-pyridyl (or similar) substituents. These allow the use of the DII part as a terdentate ligand using one isoindole nitrogen atom and two auxiliary pyridyl nitrogens in a planar fashion. Such complexes of transition metals, prepared mainly by Gade,^20−23^ Ziegler,^24,25^ and others, are further used as epoxidation or hydrogenation catalysts. Besides that, Ziegler used rhenium ion as a template for preparation of condensed DII compounds,^26^ Sanford investigated Ni complexes as potential anolyte materials used in nonaqueous redox flow batteries,^27^ and Bochkarev synthesized structurally interesting series of rare earth metals complexes.^28^

This paper describes a novel approach to the synthesis of variously substituted dissymmetrical DIIs, by a similar reaction as described earlier for amidinates.^29−33^ In the first part, we describe reaction pathways with crystallographically determined structures of key complexes and byproducts. In the second part, the reactivity of monosubstituted DIIs is reported to synthesize compounds usable as ligands for various metals, including investigation of their molecular dynamics and isomerism in solid state and solution.

## Results and Discussion

### Synthesis and Characterization of Lithium Complexes

As a part of our research program, one of the possible synthetic strategies for main group metal amidinates, addition of silylated lithium amide to nitrile group,^29−31^ has been explored for (oligo)nitriles with aliphatic chain as well as several di- up to tetracyanobenzenes.^32,33^

**Scheme 1:**
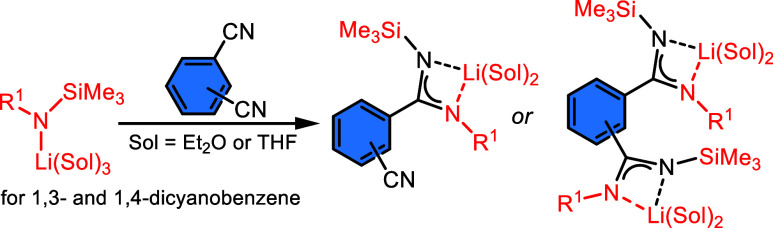
Method for the Synthesis of Lithium Amidinates from Lithium Amides and Dicyanobenzenes,^33^ Sol = THF, or Diethyl Ether

**Scheme 2:**
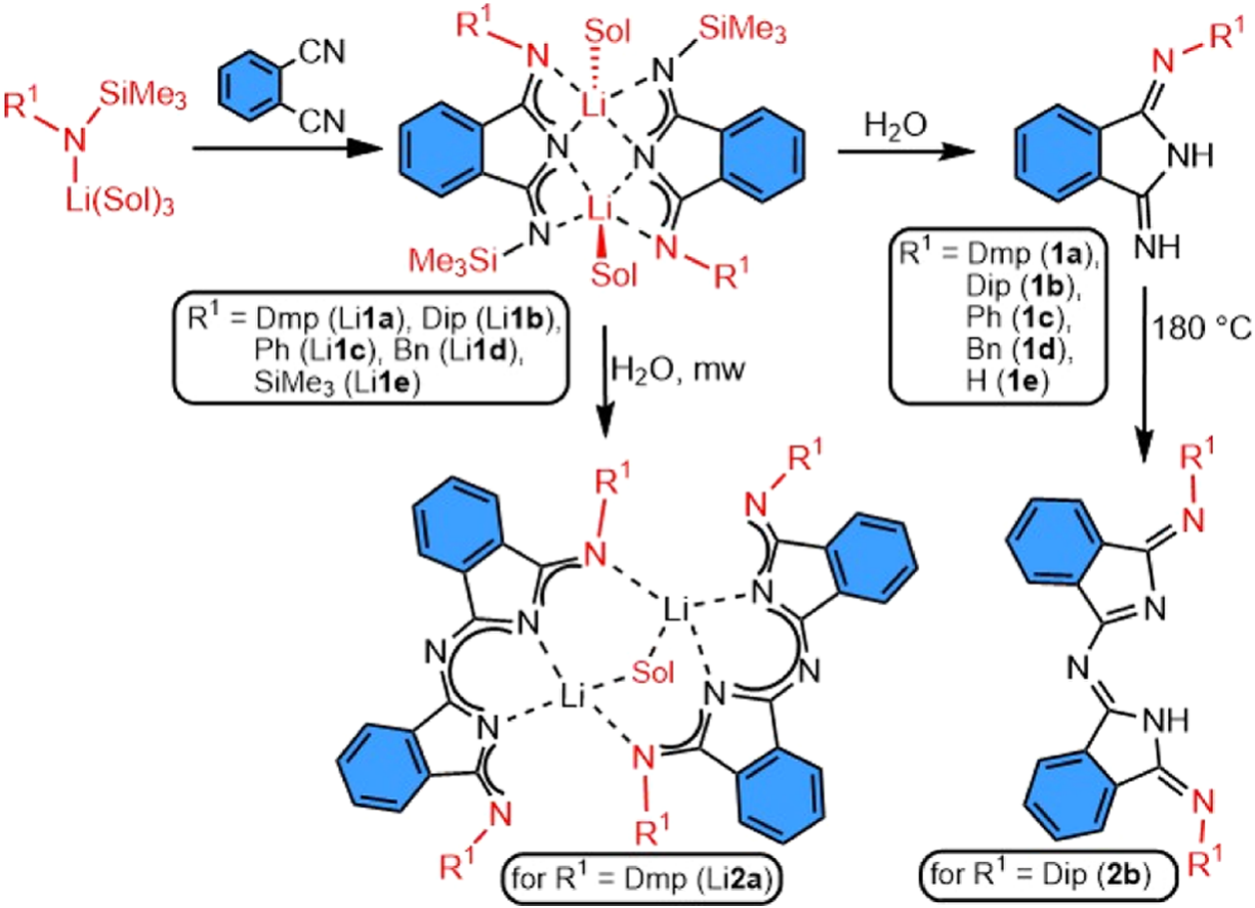
Observed Reactivity of Silylated Lithium Amides to DIIs Dmp = 2,6-dimethylphenyl-; Dip = 2,6-diisopropylphenyl-; sol = THF, diethyl ether, mw = microwave reactor.

In the series of dicyanobenzenes, the 1,3- and 1,4-derivatives gave, according to the stoichiometry, desired lithium nitriloamidinates and dilithium bisamidinates (Scheme 1).^33^ Inspired by these transformations, we carried out the reaction of 1 equiv of 1,2-dicyanobenzene^33^ with several trimethylsilylated lithium amides. In strong contrast to reactions of 1,3- and 1,4-dicyanobenzene, it gives cyclization products lithium 1-(trimethylsilylimino)-3-(arylimino)isoindolines in high yields (Scheme 2). Addition of lithium amide to an unsaturated nitrile bond followed by a migration of the trimethylsilyl group and ring closure is a logical explanation of the process. According to ^1^H and ^13^C NMR spectra, it seems the complete conversion is accomplished within a couple of hours at room temperature. The ^1^H and ^13^C NMR spectra of all lithium complexes are in line with spectral assignments made by Spiessens or Ziegler^16−19^ for alkyl- or aryliminoisoindolines. Only the ^13^C signals of endocyclic carbon atoms C1 and C7a (see the Supporting Information (SI)) are shifted to lower field by ∼7 and ∼5 ppm, respectively, which can be attributed to the influence of lithium coordination. Thanks to the symmetrical structure of Li**1e**, we can observe only three broader signals in the ^1^H NMR spectra in tetrahydrofuran (THF)-*d*_8_, namely, the aromatic hydrogens of isoindole and the trimethylsilyl group with the corresponding integral intensity. There are five ^13^C signals that can be unambiguously assigned to the isoindole unit and the SiMe_3_ groups. In the ^29^Si NMR spectrum, one resonance of the SiMe_3_ group at −8 ppm is observed similarly as in the ^7^Li NMR spectrum, where a relatively narrow signal occurs at 1.1 ppm.

**Figure 2:**
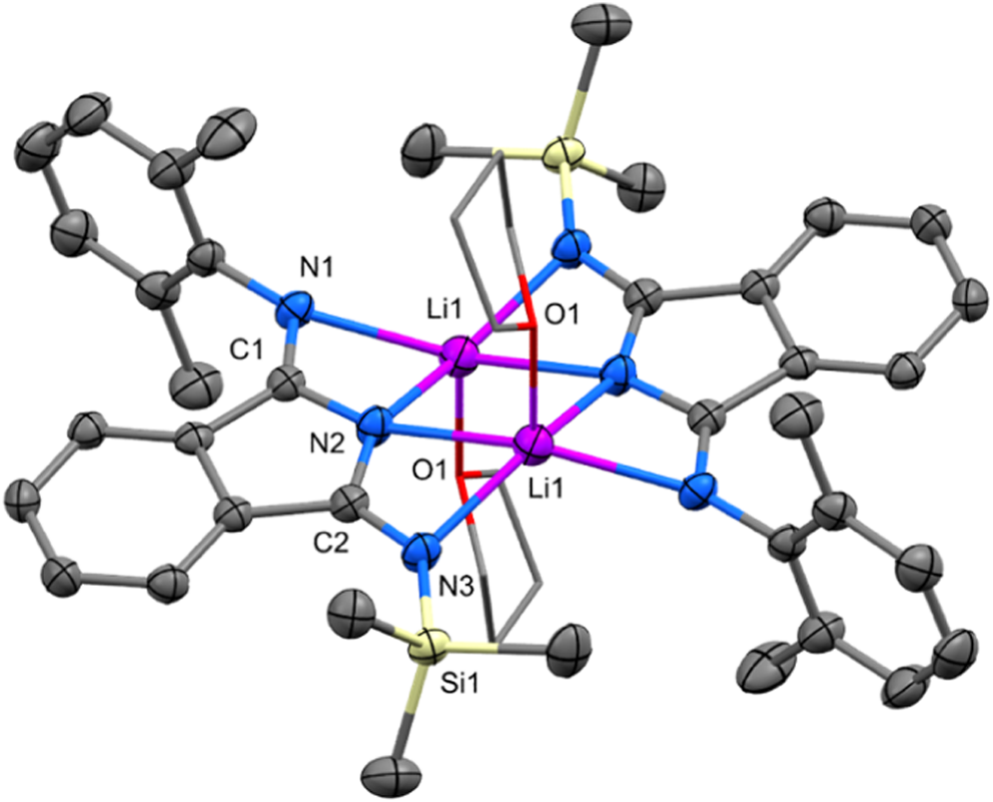
Oak Ridge thermal ellipsoid plot (ORTEP) view 50% probability level of the molecular structure of Li**1a**. Hydrogen atoms are omitted for clarity; THF ligands are shown in wireframe mode. Selected bond lengths (Å) and angles (deg): N1–C9 1.418(3), N1–C1 1.289(3), C1–N2 1.372(3), N2–C2 1.363(3), C2–N3 1.296(3), N3–Si1 1.713(2), N1–Li1 2.209(4), N2–Li1 2.127(4), N3–Li1 2.100(4), C1–N1–C9 120.79(18), N1–C1–C4 132.14(19), N1–Li1–N2 63.7(1), N2–Li1–N3 64.1(1). Symmetry code: −*x*, −*y*, −*z*.

Lithium complexes Li**1a** and Li**1b** (Scheme 2) were crystallized as single-crystalline materials in the first crop from the reaction mixture with yields of about 65%. Both complexes are dimeric with a nearly planar arrangement of isoindoline rings (Figures 2 and S47). Each of two lithium atoms, which are ∼0.5 Å below and above the central plane, connecting two endocyclic and two exocyclic nitrogen atoms of both isoindoline moieties. Thus, the lithium atoms are found in unusual geometry of nearly perfect square pyramid as found only for some lithium porphyrinoids.^34^ Dmp and Dip groups in Li**1a** and Li**1b** are perpendicularly oriented to the central plane. The interatomic distances and angles between lithium and nitrogen or oxygen atoms are in line with similar type of distances previously found in structures of dissymmetric trimethylsilyl substituted lithium amidinates.^23,24^ On the other hand, interatomic distances found in the ligand (see Figure 2) clearly demonstrate that double bonds are located at the peripheral imino groups and thus a delocalized character of NCN chelating unit in amidinates is not taking place in lithium diiminoisoindolines Li**1a** and Li**1b**.

Low quality of Li**1c**, Li**1d**, and Li**1e** crystals did not allow structure determination by single crystal X-ray diffraction (sc-XRD), but an interesting adduct of dinuclear Li**1e** with partially hydrolyzed species was crystallized as fortuitous minor material (see Figure S72). This adduct consists of four diiminoisoindoline units, and two of them are partially hydrolyzed, which is manifested by the replacement of one SiMe_3_ substituent with a hydrogen atom. Four lithium atoms are coordinated in the structure, each by means of four diiminoisoindoline nitrogens. The N–Li interatomic distances are in the range of 2.07–2.17 Å, which are comparable with the sum of the covalent radii of nitrogen and lithium atoms of 1.99 Å^35^ and similar types of separations in Li**1a** and Li**1b**.

**Scheme 3:**
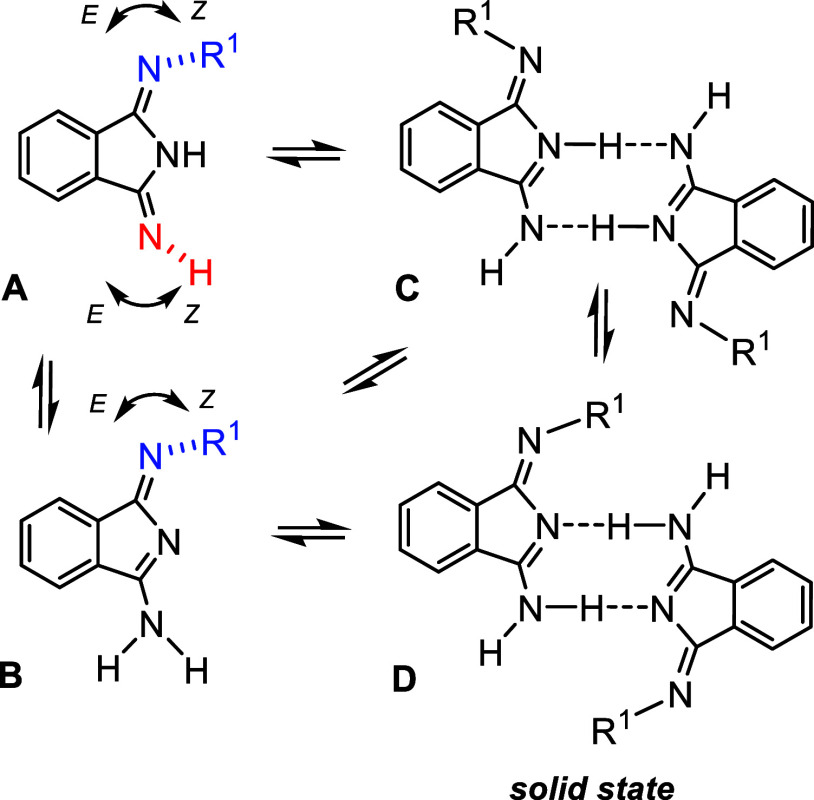
Plausible Isomerization Pathways of Dissymmetrically Substituted DIIs Monomeric (A, B) and dimeric (C, D) species. Circular arrows illustrate *E*/*Z* isomerism at imino groups—*E*-isomers are shown only for dimeric species of the type C.

Protonolysis of all mentioned lithium complexes occurs just by letting solutions or crystals on the air for a period of a couple of minutes to hours or adding nondried solvent to the crude reaction mixture. Alkyl- or aryliminoisoindolines **1a**–**e** can be crystallized from solutions after treatment with water in very good yields. According to the ^1^H and ^13^C NMR spectra in dimethyl sulfoxide (DMSO-*d*_6_), the prepared compounds exhibit similar behavior in DMSO-*d*_6_ solution as described by Spiessens or Shishkin for non- to symmetrically disubstituted DIIs.^16,17,36−38^ The imino form A, *Z*-isomer at the nitrogen atom highlighted in blue color in Scheme 3, predominates in all of the compounds. In contrast to reported works, we are strongly convinced by the set of two-dimensional (2D) NMR (correlation spectroscopy (COSY), heteronuclear multiple bond correlation (HMBC), and nuclear Overhauser effect spectroscopy (NOESY)) experiments that the tautomeric equilibrium between A and B forms does not exist in solutions of THF-*d*_8_ and CDCl_3_, and only the same *E*/*Z*-isomerization of diimino forms A is taking place. In addition, there is no evidence of dimer formation (Scheme 3C or D), suggested by other authors as a part of the tautomerization process, in the solvents used. The solid proof for that statement is also the diffusion-ordered spectroscopy (DOSY) NMR measurement (Figures S41 and S42) of **1a**, which gave traces with very close diffusion coefficients for both sets of signals (for *E*- and *Z*-isomers of diamino form A) observed in the spectra. Also, the mixture of **1a** and **5ab**, which is not capable of making a dimer, was investigated by this technique in order to evaluate **1a** not being aggregated in solution.

**Figure 3:**
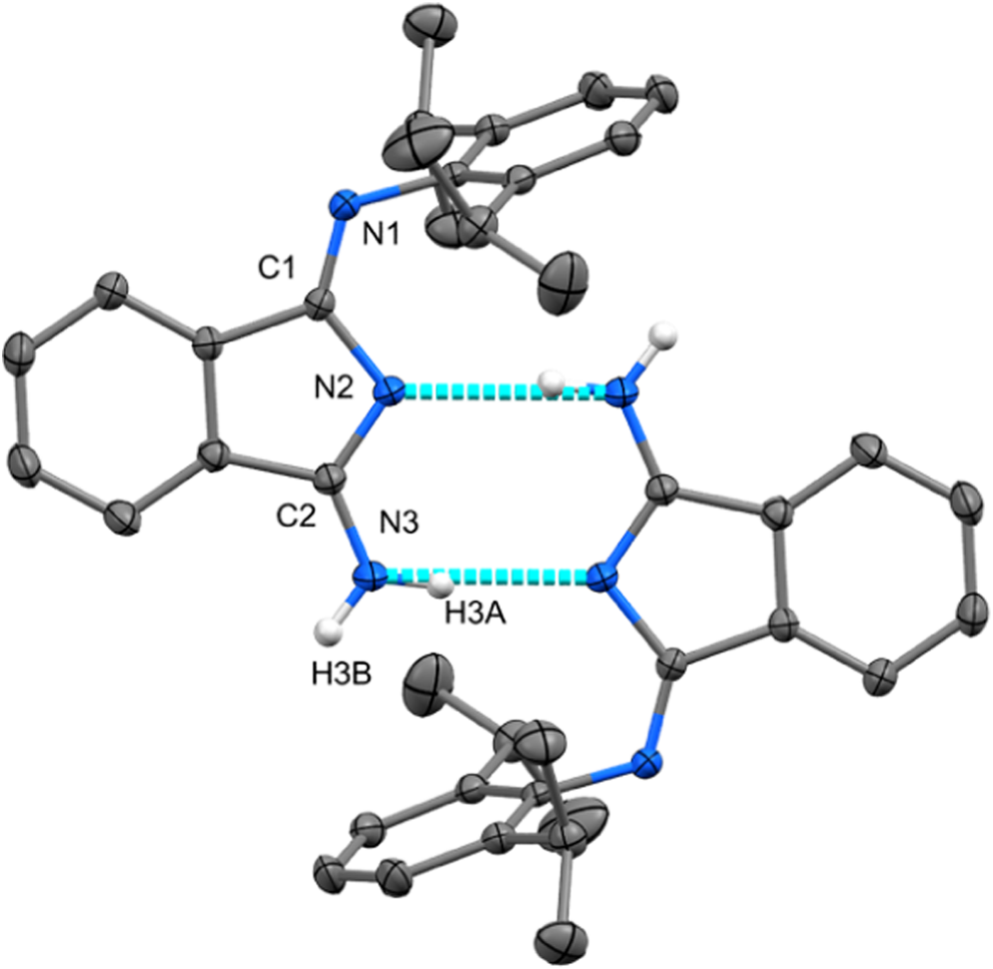
Molecular structure of **1b**, ORTEP view 50% probability level. Hydrogen atoms have been omitted for the sake of clarity. Selected bond lengths (Å) and angles (deg): N1–C9 1.4241(13), N1–C1 1.2790(13), C1–N2 1.4052(13), N2–C2 1.3309(13), C2–N3 1.3253(14), C1–N1–C9 119.70(9), N1–C1–C4 123.60(9).

The molecular structures of **1a**, **1b**, and **1c** determined in the solid state are the best to be described as centrosymmetric dimers. These dimers are connected by two strong H-bridges mediated by isoindole nitrogen atoms as donors of electron density and NH_2_ groups as acceptors (Scheme 3D). Structure of **1c**, previously described in the literature,^39^ was redetermined in order to have the direct comparison with structures of **1a** and **1b** (Figure 3). In both structures (analogously to **1c**) the acidic hydrogen atoms are unambiguously located at the terminal exocyclic nitrogen atom (Scheme 3D), which is in contrast to the observations made in solution. The shortest distance was identified between C1 and the exocyclic nitrogen atom N1, while C2–N2 and C2–N3 are mutually comparable and significantly longer. One would expect, the hydrogen atoms will be localized as in solution at N2 (judged from the longest C1–N2 separation) and N3 atoms. But the presence of both hydrogen atoms at the N3 atom prevailed probably thanks to the formation of the dimer via strong H-bonds (Figure 3). While the structures of **1a** and **1b** exhibit planar organization of the DII fragments and perpendicular orientation of the Dmp and Dipp substituents (Figure 3), respectively, the interplanar angle of DII groups in **1c** is 87.14(12)° most probably due to steric reasons (see Figure S55).^39^ In fact, this rather polarized arrangement found in the solid state is not in line with theoretical calculations reported elsewhere.^36^ We took the opportunity to reinvestigate the dimerization of **1a** carefully.

It seems, the tautomer with NH_2_ moiety dimerizes upon formation of both planar and perpendicular dimers (vide supra for structures of **1a**, **1b**, and **1c**) as the lowest energy species when compared to other possible dimers and monomers by ∼5 kcal/mol (for more details, see Figures S1 and S2). The reason for this tautomeric rearrangement going from solution to the solid state is the stabilization of the lowest energy and optimal structure by resonance-assisted hydrogen bonding (RAHB),^40−42^ which is responsible not only for conformation of the dimer but also for elongation of C1–N2 bond while bonds within N2–C2–N3 fragment retains π-conjugated.

When 2 molar equiv of appropriate lithium amide are added to the 1,2-dicyanobenzene, only the mixture of Li**1a**, Li**1b**, or Li**1d** together with unreacted amides were obtained, which supports the fact that no further addition reactions to the silylated lithium DIIs are possible at specified conditions. Unfortunately, these reactions cannot be performed at elevated temperatures, as the starting amides are unstable in coordinating solvents above 40 °C.

In addition, the microwave treatment of the reaction mixture after the water treatment of Li**1a** in xylenes yields the product of DII condensation and subsequent coordination of lithium atom—Li**2a** (Scheme 2). In this dinuclear centrosymmetric structure, the lithium atom is bound by one DII inside the six-membered heterocyclic ring and by the second DII via exocyclic nitrogen atom. Oxygen atom of the diethyl ether molecule (recrystallization solvent), as a center of symmetry, is coordinated to both lithium atoms. A similar DII fragment can be also prepared by heating neat compound **1b** to 180 °C. Upon heating, elimination of ammonia occurs and self-condensation product **2b** consisting of two DII units is formed (inseparable mixture with **1b**) (Scheme 2, see Figures S46, S47, and S52 for more details and crystal structures).

**Scheme 4:**
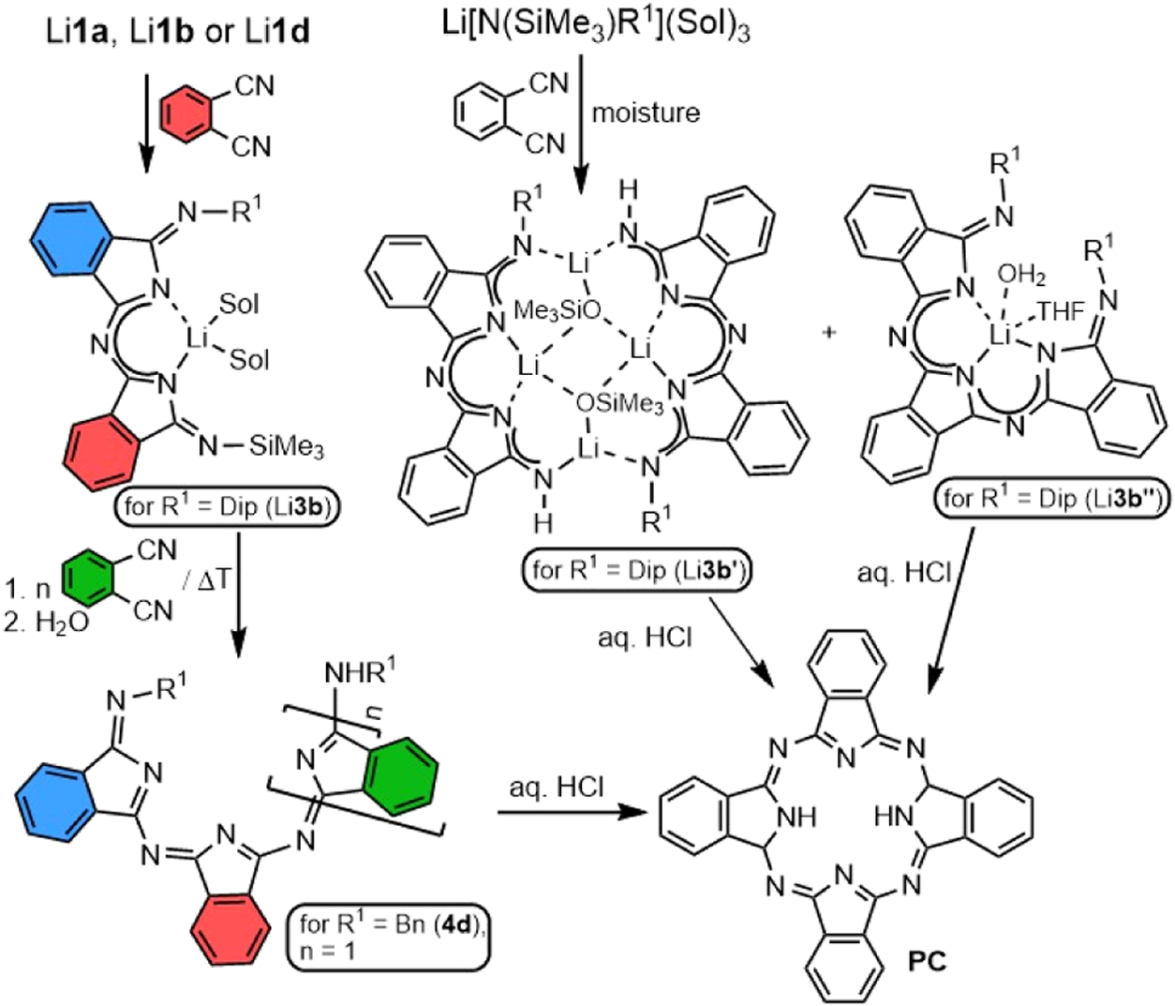
Observed Reactivity of Silylated Lithium DIIs Dmp = 2,6-dimethylphenyl-; Dip = 2,6-diisopropylphenyl-; sol = THF, diethyl ether.

On the other hand, when we repeated the preparation of the complex Li**1b**, a significant amount of air and moisture was accidentally allowed to penetrate into the reaction vessel after a couple of minutes of the reaction. It seems moisture destroyed the unreacted portion of lithium amide, and thus, the 1,2-dicyanobenzene became slightly excessive. Unknown type of lithium complex Li**3b′**, as a partially hydrolyzed product of the addition of Li**1b** to 1,2-dicyanobenzene, was isolated as orange crystals and characterized in solution as well as in the solid state (Scheme 4, see Figure S49).

Obviously, the second bis(imino)isoindoline moiety was created via further migration of the trimethylsilyl group, which is essential for the second addition. This has been also documented by the fact that diiminoisoindoline **1b** does not provide any product containing two bis(imino)isoindoline units, when deprotonated by lithium diisopropylamide, treated with 1,2-dicyanobenzene and subsequently hydrolyzed. Li**3b′** consists of a dimeric arrangement of slightly deformed planar heterocyclic units interconnected by four lithium atoms where two of them compensate negative charge of each of the ligands and the second two came from the product of the partial hydrolysis of the complex—the lithium trimethylsilanolate. The first two are found in the plane of the ligand completing the central six-membered ring together with exocyclic nitrogen atoms, and the second couple of lithium atoms are located at peripheries complexed by endocyclic nitrogen atoms below and above the central ring by ca. 0.66 Å. Lithium atoms in the central area are four-coordinated in the vicinity of distorted tetrahedra, while the peripheral ones have trigonal planar geometry and both are connected by the trimethylsilylanolate units. The only deprotonable hydrogen atom is localized on the peripheral imino nitrogen on the opposite side to the Dip group. Very minor species Li**3b″**, characterized exclusively by sc-XRD methods, arose as a byproduct of that reaction alongside Li**3b′** (Scheme 4, see SI S49). Complex Li**3b″** consists of three interconnected DII units, where the terminal nitrogen atoms are substituted by Dip groups (Figure S50). All three isoindole nitrogen atoms of that monoanionic ligand coordinate the lithium atom in a planar fashion similar to the four DII units in neutral lithium phthalocyanines.^43^ Further donors to lithium ion in Li**3b″** are THF and a water molecule, which forms H-bonds to peripheral imino groups. For all of the compounds with more than one DII unit, the terminal imino groups *E*-isomers were observed in the solid state, and one can also suggest the same arrangement in solution. By acidolysis of Li**3b′** and Li**3b″** by aqueous HCl, new unprecedented pathway to phthalocyanine **PC** (characterized by IR spectroscopy and mass spectrometry—Figures S140 and S145) is available (Scheme 4). The plausible mechanism of this reaction can be similar to various sequential mechanisms initiated and templated by metals reported earlier.^44^

**Figure 4:**
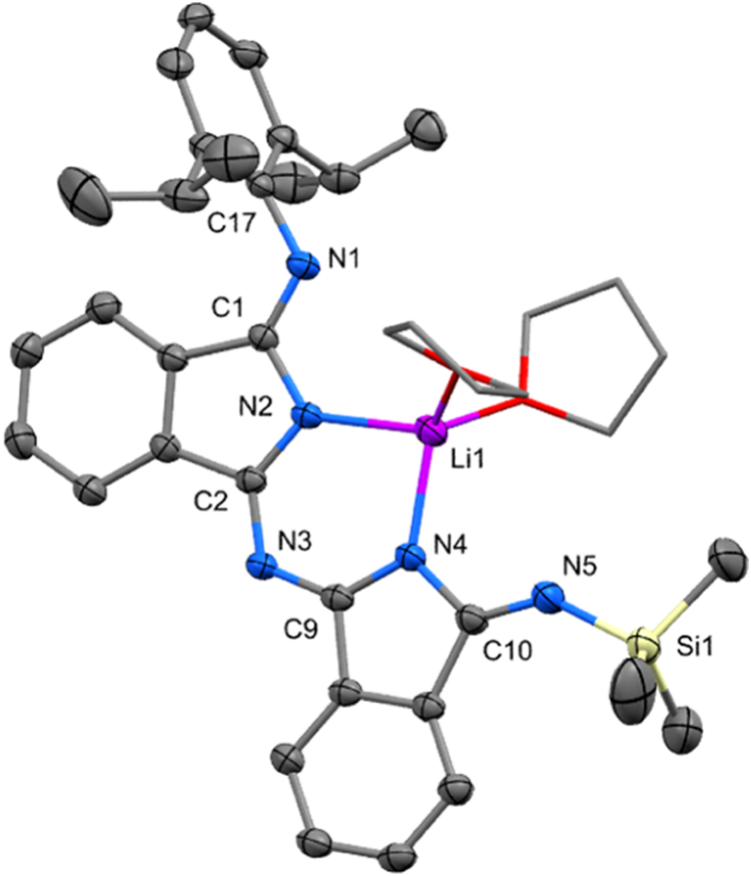
Molecular structure of Li**3b**, ORTEP view at 50% probability level. Hydrogen atoms are omitted for clarity. Selected bond lengths (Å) and angles (deg): N1–C17 1.427(4), N1–C1 1.273(3), C1–N2 1.414(4), N2–C2 1.332(3), C2–N3 1.343(3), N3–C9 1.349(4), N4–C9 1.330(3), N4–C10 1.422(4), N5–C10 1.270(3), N5–Si1 1.732(3), N2–Li1 2.019(5), N4–Li1 1.985(5), C1–N1–C17 121.8(2), N1–C1–C4 131.1(2), N2–Li1–N4 90.0(2), C10–N5–Si1 138.7(2).

Characterization of Li**3b′** led us to the idea of preparing a lithium complex containing the trimethylsilyl and Dip group connected to the moiety composed of two diiminoisoindoline fragments. The excess of 1,2-dicyanobenzene (2 molar equiv) was added to solutions of the parent amides. After the conversion of the amide Li[N(SiMe_3_)Dip](THF)_3_, two types of crystals were isolated from the solution—minor number of green-yellow ones determined to be Li**1b** and orange crystals of Li**3b**. Complex Li**3b** (Figure 4), as expected, contains two diiminoisoindoline fragments as well as Dip and SiMe_3_ substituents on exocyclic nitrogen atoms. This complex is mononuclear, and the lithium atom is coordinated by two isoindole nitrogen atoms in planar arrangement; furthermore, two donor THF molecules are present.

Insoluble green, presumably oligomeric material **4d**, has been isolated when an excess of 1,2-dicyanobenzene was added to Li**1d** or Li**3d** (Scheme 4), warmed to 50 °C for 4 h, and poured into cold water. This procedure was followed by filtration and Soxhlet extraction of both solid samples by dichloromethane in order to remove unreacted material 1,2-dicyanobenzene and substituted DIIs with lower molar masses. Crude products treated by water or HCl gave deep blue crystalline material. The extracts were subjected to high-resolution mass spectrometry (HR-MS) investigation. In the deep blue sample, prepared at acidic conditions, almost pure unsubstituted phthalocyanine (**PC** Pigment Blue 16) was identified by a mass of 515.1727 Da and identical IR spectrum. The treatment at neutral conditions gave a petrol green sample, a mixture of compounds consisting of two or three DII units and benzyl substituent, characterized by exact masses of 582.2408, 492.1936, and 364.1563 Da and molecular formulas C_38_H_28_N_7_, C_31_H_22_N_7_, and C_23_H_18_N_5_, respectively. Further treatment of green sample by aqueous HCl immediately gave a blue solid, identical by spectral analysis to **PC** from the first procedure.

### DII-Guanidines: Synthesis, Structure, and Tautomerism Investigation

**Scheme 5:**
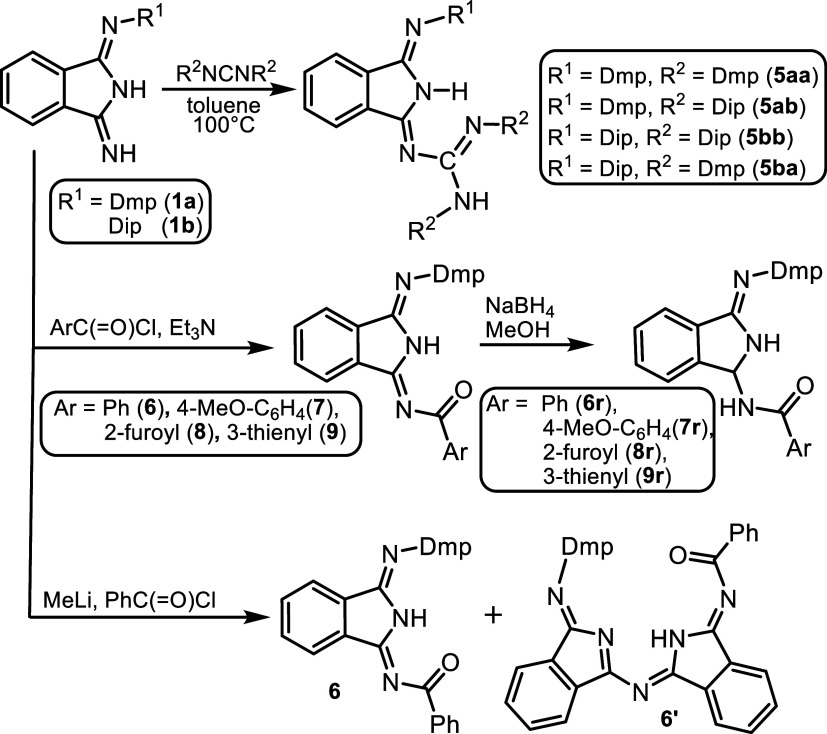
Derivatization of DIIs **1a** and **1b**

To the best of our knowledge, there are a limited number of species where the direct connection of DII and guanidine fragments is present. The first class of these organic compounds and metal (Re, Mn, Cu, Fe) complexes is characterized by middle DII group symmetrically *N*-substituted by another diiminoisoindoyl fragments with a planar structure of terdentate ligand for Mn, Cu, and Fe.^45−48^ While the second class of DIIs substituted by two pyridin-2-yl or pyrimidin-2-yl fragments are used as *C*,*N*,*N*- or *N*,*N*,*N*-chelating ligands for palladium^49^ or as preorganized ion traps.^50^ Both compounds **1a** and **1b** exhibit an activated nature of NH or NH_2_ functional groups in the sense of very good performance in the nucleophilic addition to cumulated unsaturated system of carbodiimides, which is simple for aliphatic amines and/or carbodiimides.^51,52^ On the other hand, the synthesis of guanidines bearing aromatic or bulky substituents usually needs harsh conditions or metal catalysis.^53−56^ DII-guanidines **5aa**–**bb** (Scheme 5) were prepared in very good yields in warm toluene without any catalyst in 48 h. The chemical structure of **5aa**–**bb** in solution was investigated by NMR spectroscopy.

**Figure 5:**
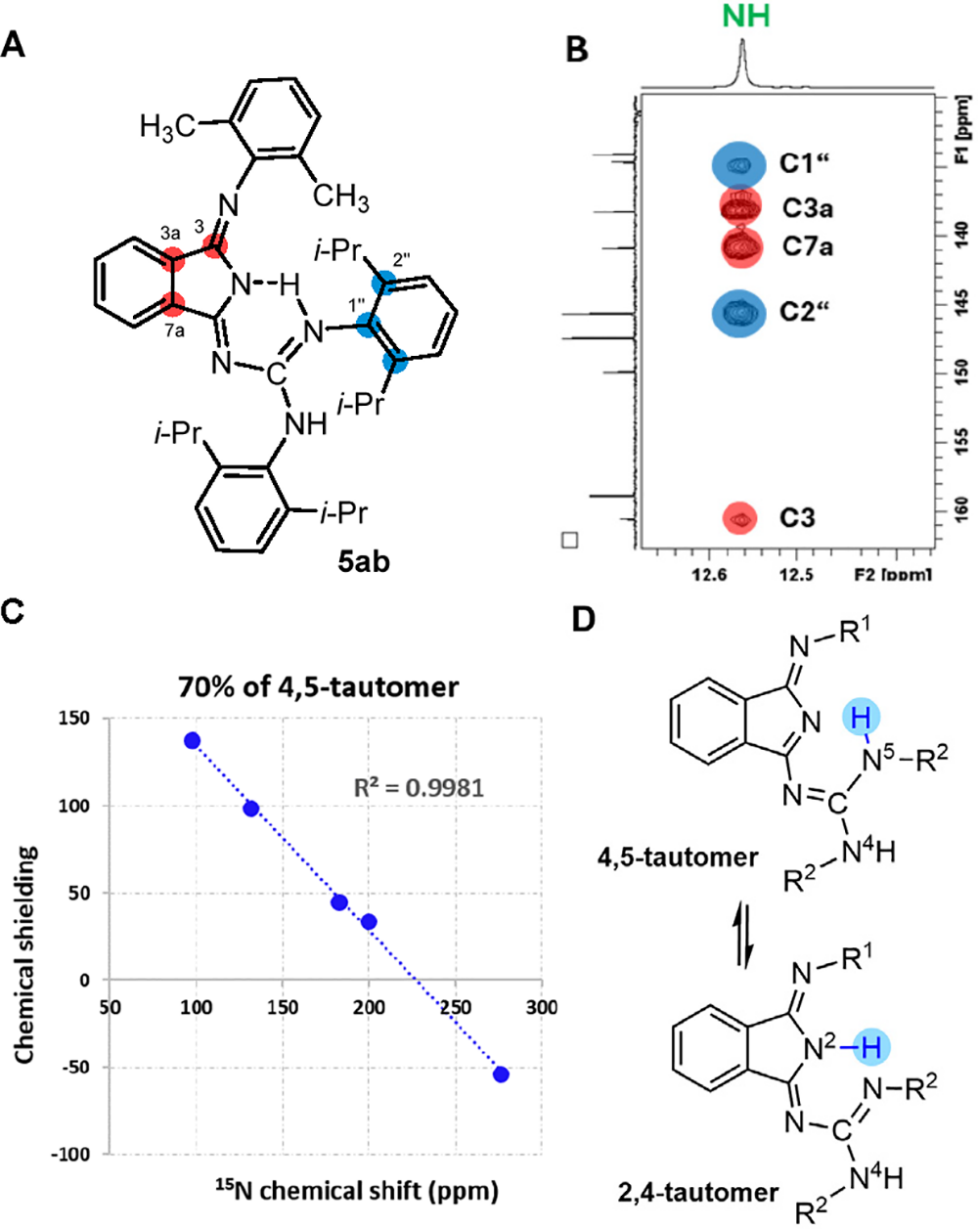
(A) Structure of compound **5ab**. (B) A part of HMBC of **5ab** showing that NH is involved in intramolecular hydrogen bond (12.56 ppm) provides cross-peaks corresponding to spin–spin interaction through 2 and 3 chemical bonds to both moieties indicating tautomeric equilibrium. (C) ^15^N NMR/density functional theory (DFT) correlation of **5ab**. ^15^N NMR were measured in THF-*d*_8_ at 25 °C, the geometry was optimized at B3LYP/6-31g(d,p)/PCM(THF) level of theory, and the NMR parameters were calculated at B3LYP/6-311+g(d,p) PCM(THF) level of theory. (D) Possible tautomeric equilibrium.

NMR spectroscopy revealed a significant solvent effect in all four compounds **5aa**–**bb**. While in C_6_D_6_, two sets of signals in 95:5 ratio were observed, in THF-*d*_8_, the only one set of NMR signals indicating just one stable form was found. ^1^H spectra of all compounds in this series revealed one downfield shifted signal of NH involved in strong intramolecular hydrogen bond (12.56 ppm for **5ab** as an illustrative example—see Figure 5B). There are two hypothetical tautomers fulfilling the criterion for a strong RAHB, with hydrogen atom attached to N2 or N5 atoms (Figure 5). The position of NH could be assigned by ^1^H, ^13^C HMBC NMR experiment. However, HMBC revealed spin–spin interaction of NH with carbon atoms from the indole part as well as from the phenyl part, as shown in Figure 5. This indicates the possible coexistence of both tautomers. To inspect this, we measured NMR spectra at a low temperature (−100 °C), but no additional set of signals was detected. Therefore, the tautomeric equilibrium must be investigated by an advanced NMR approach including quantum-chemical calculations.

When investigating the tautomeric equilibria of small molecules, the experimentally obtained NMR parameters are systematically correlated with the calculated values of each particular tautomer. First, the geometry of all possible tautomers is optimized, and from the low-energy forms, the NMR parameters are calculated. Usually, ^13^C chemical shifts are used for the study of tautomeric equilibria. However, we obtained all the correlations with correlation factor *R*^2^ > 0.99 (Figures S4–S10 in the SI), precluding unambiguous determination of the present tautomeric forms. With respect to the fact that guanidines are rich in nitrogen atoms, we decided to use ^15^N NMR parameters for our investigation. Although the natural abundance of ^15^N is low (0.4%), we succeeded in the extraction of ^15^N chemical shifts from 2D ^1^H, ^15^N NMR experiments. Moreover, five nitrogen atoms in the molecule are a sufficient number for construction of linear regression needed for correlation of experimental data with the calculations. As an example, we show tautomeric equilibria of compound **5ab** (Figure 5). In **5ab**, ^15^N NMR/DFT correlations of 2,4-tautomer showed *R*^2^ = 0.5414; on the other hand, *R*^2^ for 4,5-tautomer tautomer was 0.9640 (Figures S11–S14 in SI). The calculated data did not match the experiment perfectly. Furthermore, the energy difference between 2,4- and 4,5-tautomers was estimated to be 0.46 kcal/mol corresponding to a ca. 7:3 ratio with 4,5-tautomer to be a predominant form. Averaging the calculated ^15^N shielding constants according to Boltzmann distribution provided the best-fit *R*^2^ = 0.9981 (Figure 5). Based on these results, we believe that in solution is a tautomeric equilibrium where 70% of 4,5-tautomer is present. The same approach was applied to the rest of the compounds from this series as shown in Figures S11–S14 in the SI.

The tautomeric equilibria of **5aa**–**bb** were also investigated in the solid state by XRD. With respect to the maxima attributable to the N–H atoms on the Fourier difference electron density maps, the hydrogen atoms were placed on N5 atom of guanidine in cases of **5aa** and **5ab**, and to the N2 atom of the DII moiety in cases of **5ba** and **5bb**, respectively. This placement is in line with the description of the bonding situation within the central DII-guanidine moiety and the concept of conjugation of π-electron density. On the other hand, we are aware of alternative descriptions operating with the mixture of both discrete tautomers in the solid state and as such with a placement of hydrogen atom to both positions with partial occupancy as first described by Bertolasi and Gilli.^57^

**Figure 6:**
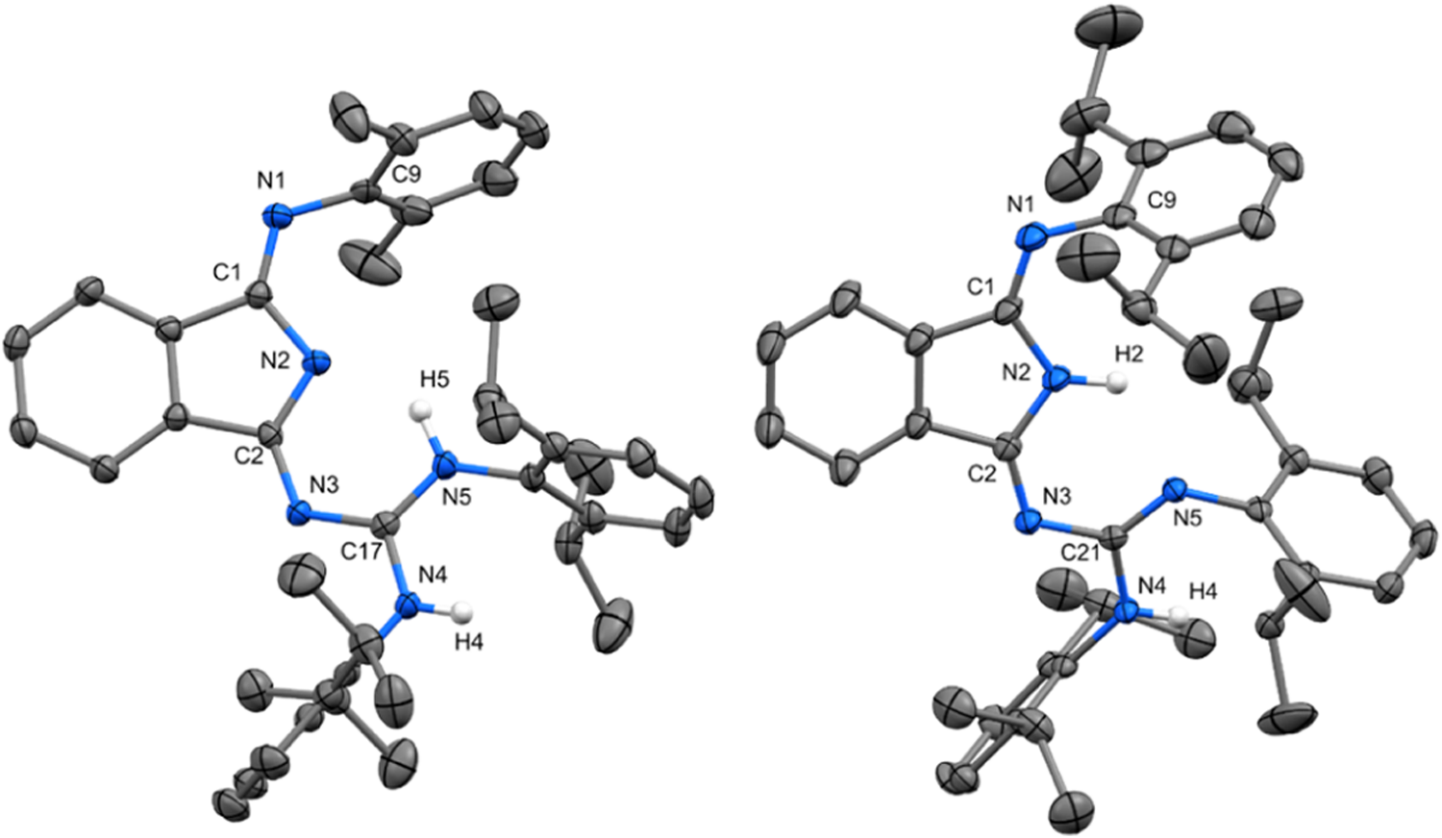
Molecular structures of **5ab** (left) and **5bb** (right); ORTEP views 50% probability level. Hydrogen atoms are omitted for clarity. Selected bond lengths (Å) and angles (deg) for **5ab**: N1–C9 1.4216(17), N1–C1 1.2753(16), C1–N2 1.4055(15), N2–C2 1.3467(15), C2–N3 1.3249(15), N3–C17 1.3573(15), N4–C17 1.3556(15), N5–C17 1.3278(16), C1–N1–C9 119.95(11), N1–C1–C4 124.69(11), C2–N3–C17 119.42(10), N4–C17–N5 119.96(11). For **5bb**: N1–C9 1.419(2), N1–C1 1.271(2), C1–N2 1.4085(18), N2–C2 1.361(2), C2–N3 1.3013(18), N3–C21 1.3573(15), N4–C21 1.3880(18), N5–C21 1.3005(19), C1–N1–C9 121.07(13), N1–C1–C4 125.30(13), C2–N3–C21 118.88(13), N4–C21–N5 122.16(12).

Although all of the **5aa**–**bb** have very close molecular structures one to each other, some discrepancies are observable. Predictably, the compounds with smaller Dmp substituents, especially at the DII fragment are a bit more compact with nearly ideal planar arrangement of the DII and guanidine groups, thus forming the structure with characteristic six-membered ring N=C–N=C–N(H) connected by relatively strong H-bond. The presence of the Dip groups in the molecules causes a non-negligible distortion of the N5 atom from the plane defined by DII and C3 atom of the guanidine part (see Figure S58). Differences between structures of **5aa**–**bb** are also seen when careful comparison of the interatomic distances inside the DII and guanidine fragment is made. Compounds **5aa** and **5ab** are the most representative 4,5-tautomer containing both hydrogen atoms on the guanidine unit (Figure 6). Bond distribution consists of a localized set of single and double bonds C9–N1–C1–N2 and then a delocalized system within N–C–N guanidine fragment. Compounds **5ba** and **5bb** are represented as 2,4-tautomers, with one hydrogen atom located on isoindole and the second one on the guanidine fragment (Figure 6). Taking into account atom separations within whole molecules of both tautomers, major differences are found in the guanidine fragments. Guanidines in 2,4-tautomers of **5ba** and **5bb** exhibit a higher localization of the π-electron density. Depending on substituent, interatomic distances within guanidine are prolonged due to the effect of steric hindrance.

With the aim to use compounds **5aa**–**bb** as protoligands in coordination chemistry, their reactivity with *n*-BuLi, LDA, and MeLi was explored to find the most efficient deprotonating agent. One molar equivalent of *n*-BuLi and MeLi did not provide quantitative deprotonation in the case of **5aa**–**bb**. However, 1 equiv of LDA performs deprotonation of the most acidic hydrogen atom in **5aa** and **5ab** quantitatively. For sterically more hindered compounds **5ba** and **5bb**, conversions of analogous reactions are only around 46% and can be increased by the addition of an excess of LDA; unfortunately, the quantitative conversion has never been achieved (see Table S10).

### Amido Derivatives: Synthesis, Structure, *E*/*Z*-Isomerization, and Dimerization Investigation

As the next target, DII **1a** substituted by an acyl moiety was selected. The major motivation was to establish a novel type of species with DII unit and the carbonyl group in the structure, which would be capable of coordinating the metal center. The only example of similar species is found in the attempted synthesis of novel type of helical structure.^58^ The major idea is based on the formation of compounds with two substituted DII fragments bridged by 2,6-pyridinedicarbonyl. Instead of the tautomeric form necessary for the formation of the suggested intramolecular H-bridge, a different arrangement was observed.

The proposed synthetic procedure can be based on a direct reaction of silylated DII Li**1a** (Scheme 2) or **1a** with an acyl chloride in the presence of a base. We have chosen the second pathway, which would presumably give a lower number of byproducts. The selected procedure took into account the problems connected to high steric hindrance of Dip-substituted species and thus lower efficiency of the deprotonation process described above for DII-guanidine compounds.

**Figure 7:**
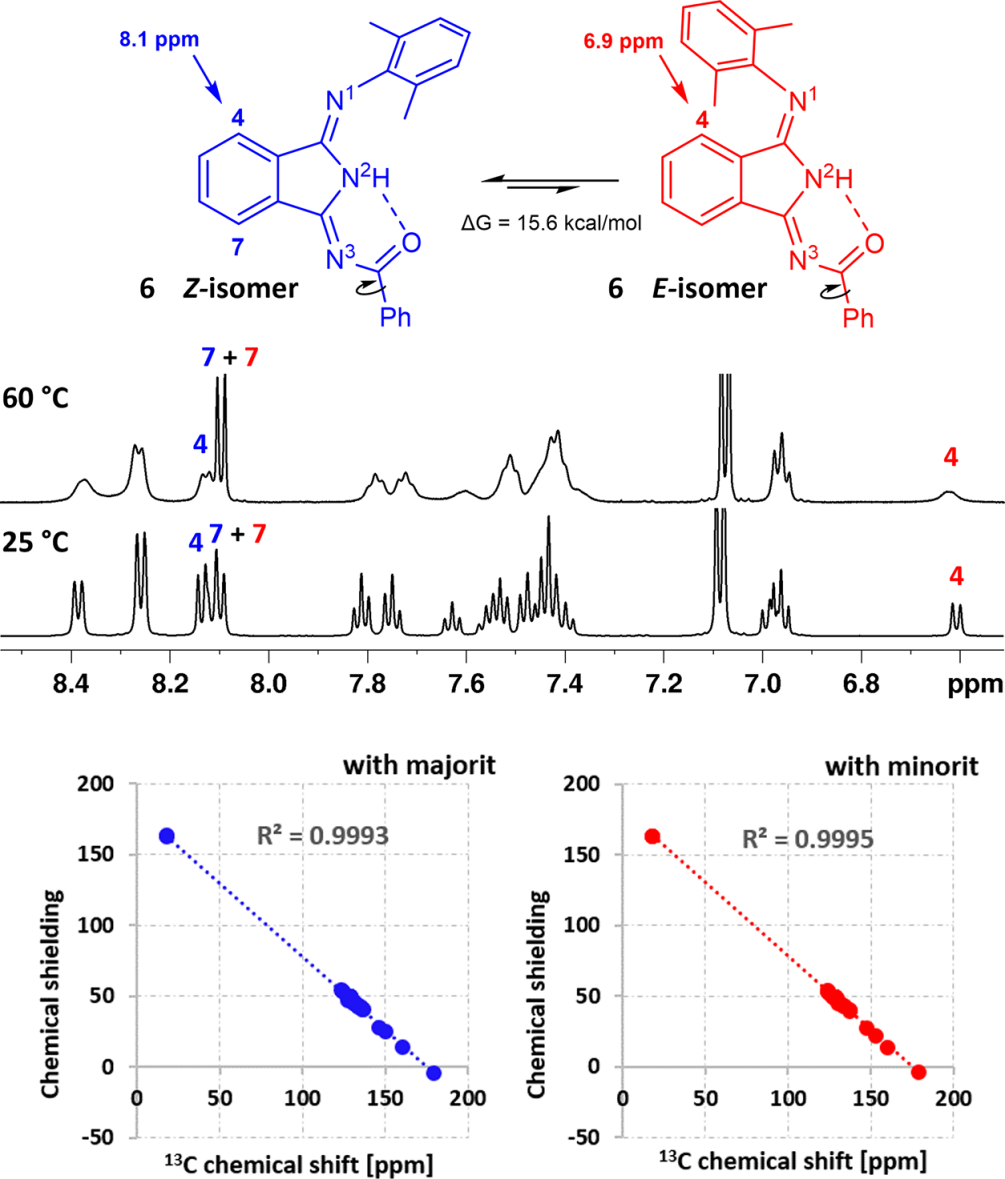
Proposed *E*/*Z* isomerization of **6**. Variable-temperature ^1^H NMR spectra of **6** showed significant signal broadening of H4 compared to H7 at 60 °C. ^13^C NMR/DFT correlation of **6**. ^13^C NMR spectra were measured in THF-*d*_8_ at 25 °C, the geometry was optimized at B3LYP/6-31g(d,p)/PCM(THF) level of theory, and the NMR parameters were calculated using GIAO at B3LYP/6-311+g(d,p) PCM(THF) level of theory.

**Figure 8:**
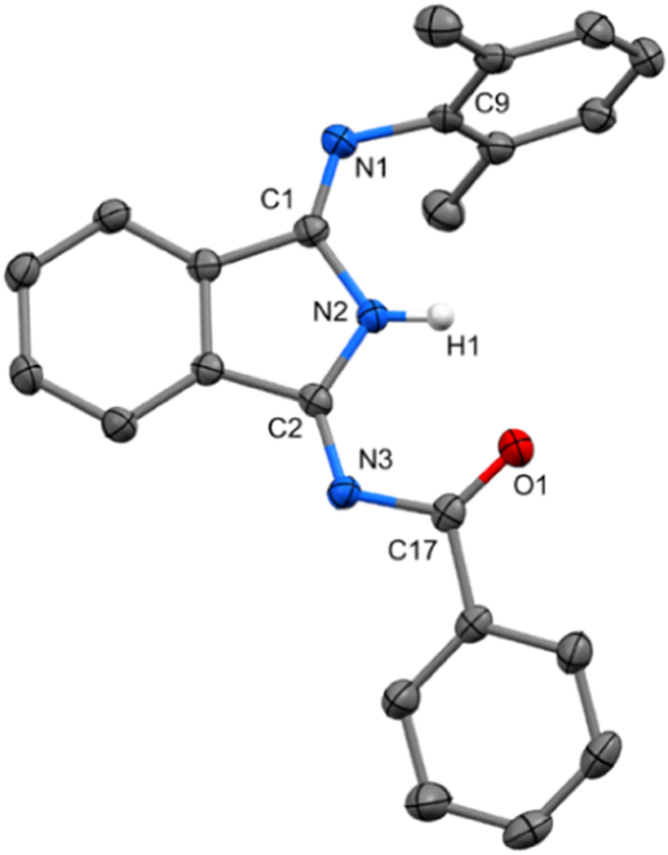
ORTEP view 50% probability level of the molecular structure of **6**. Hydrogen atoms are omitted for clarity. Selected bond lengths (Å) and angles (deg): N1–C9 1.427(2), N1–C1 1.267(2), C1–N2 1.407(2), N2–C2 1.368(2), C2–N3 1.296(2), N3–C17 1.392(2), O1–C17 1.227(2), C1–N1–C9 120.28(16), N1–C1–C4 126.08(17), C2–N3–C17 118.77(15), N3–C17–O1 125.08(17).

First, we used **1a** and deprotonated it in situ by MeLi followed by an addition of acyl chlorides (Scheme 5). Surprisingly enough, only two types of acyl-substituted DIIs were formed according to NMR monitoring. Separation of these types of compounds by crystallization of reaction mixtures gave yellow crystals of desired species **6**–**9** in about 85–90% yields. For the red crystals, they can be separated from the yellow ones only mechanically. The identification is based on further NMR studies and crystal structure determination of phenyl-substituted compounds **6** and **6′** having two interconnected DII units as described below. The formation of these minor species is precluded by using the second procedure, the addition of triethyl amine to the mixture of **1a** with acyl chlorides. ^1^H NMR spectra of all analytically pure **6**–**9** provided two sets of signals in a 2:1 ratio, indicating two distinct forms. ^1^H spectrum combined with ^1^H, ^15^N HSQC showed that each form has one NH group with the hydrogen atom involved in strong intramolecular hydrogen bond indicated by downfield shifted signals (10.5 ppm for the major form and 11.2 ppm for the minor form). The careful analysis of ^1^H spectra uncovered the biggest chemical shift difference between both forms at H4 hydrogen atom (Δδ 1.5 ppm) and ^15^N chemical shift of NH nitrogen atom differing of more than 6 ppm (see Figure S45). This indicates sterically hindered rotation/inversion at the C1–N1 bond (Figure 8), which can be further investigated by variable-temperature NMR. The ^1^H spectra measured at 60 °C showed significant signal broadening of H4 signal compared to H7 (Figure 7).

This supports our hypothesis about a coexistence of two isomers differing in orientation of dimethylphenyl moiety (*E*/*Z*). Moreover, H4 of the major form (*Z*-isomer) is less shielded (higher chemical shift) than the same atom in the minor form—*E*-isomer. This makes the H4 signal a diagnostic for molecular geometry evaluation in solution, which may be useful for more structurally complex molecular systems such as metal complexes. To support our experimental results, we employed DFT calculations to all studied derivatives **6**–**9**. From the optimized structures of each isomer, the NMR parameters were calculated. ^13^C shielding constants of each particular isomer were correlated with the experimentally obtained ^13^C chemical shifts. Calculated ^13^C shielding constants of *Z*-isomer nicely matched with the set of signals for more populated form, simultaneously, *E*-isomer fits with the set of minor signals (*R*^2^ > 0.999, details in the SI). Calculated transition-state (TS) structures and the free energy barrier Δ*G*^TS^ were estimated to be around 15.5 kcal/mol for all derivatives, the OMe derivative **7** displayed the highest barrier of 15.8 kcal/mol (details in Figures S15–S38). These values are reasonable and support our hypothesis that the two forms are correctly identified as *E*/*Z* isomers. In turn, we also calculated Δ*G*^TS^ of flipping the coplanar heterocyclic ring of **8** and **9** (depicted by circular arrows in Figure 7), which was estimated to be 11.5 and 9.2 kcal/mol, respectively. Such a low Δ*G*^TS^ cannot result in two distinct sets of NMR signals, even at low temperatures.

In the solid state, all of the structures of **6**–**9** (for illustration, see **6** in Figure 8) exhibit a perpendicular arrangement of the Dmp substituent to the planar molecular structure of the isoindole core. Acyl substituents in compounds **7**–**9** are in planar arrangement with isoindole core too, while substituent in compound **6** exhibits deviation from the plane by ca. 18°. Within their structure, a five-membered N–C=N–C=O arrangement is formed and allows an ideal neighborhood for metal coordination. The direct comparison of those structures with **1a**–**5bb** shows differences in bond distribution and hydrogen localization. Compounds **6**–**9** contain a discrete system of single and double bonds without any delocalization or polarization influence. Hydrogen atom is undoubtedly found at the endocyclic nitrogen of DII and is involved in very short resonance-assisted H-bond with oxygen atom from acyl substituent. The case of structures **8** and **9** is furthermore possible to observe static disorder caused by a flip of furoyl or thienyl substituent similarly as predicted by DFT (vide supra).

**Figure 9:**
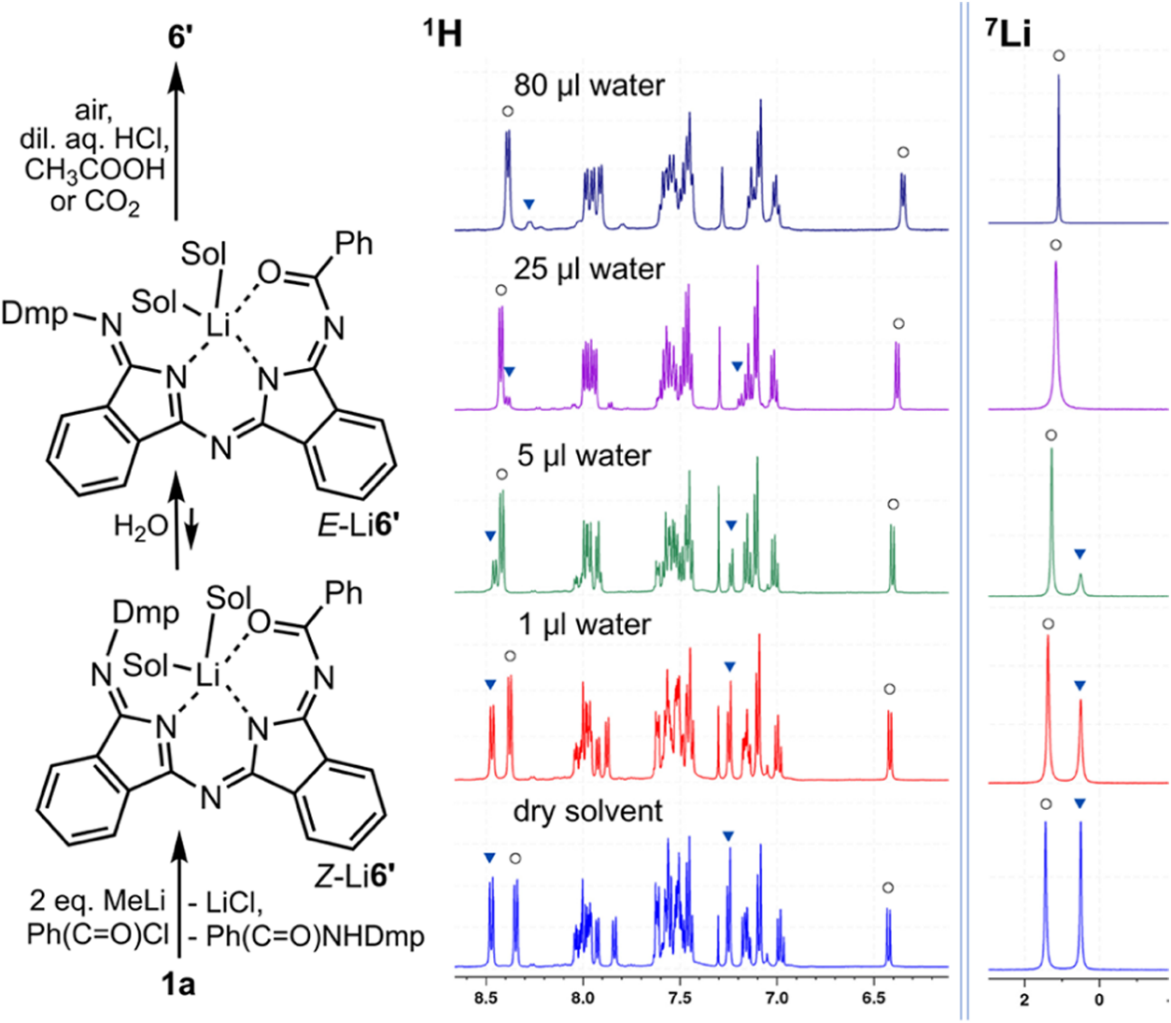
Side reaction of benzoyl derivatization of **1a** (left). ^1^H (middle) and ^7^Li (right) NMR spectra of the equilibrium between *Z*-Li**6′** and *E*-Li**6′** influenced by gradual addition of water.

**Figure 10:**
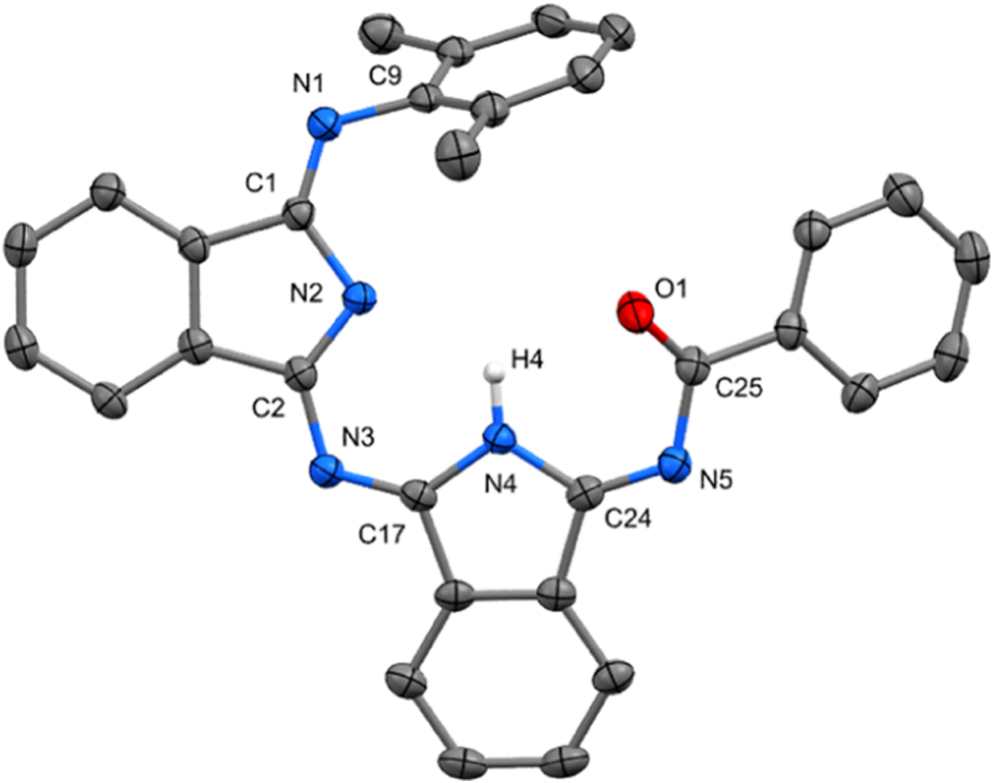
ORTEP view 50% probability level of molecular structure of **6′**. Hydrogen atoms are omitted for clarity. Selected bond lengths (Å) and angles (deg): N1–C9 1.4211(18), N1–C1 1.272(2), C1–N2 1.4219(19), N2–C2 1.3168(18), C2–N3 1.373(2), N3–C17 1.2985(19), N4–C17 1.3810(17), N4–C24 1.3908(19), N5–C24 1.2863(18), N5–C25 1.399(2), O1–C25 1.2237(18), C1–N1–C9 121.13(13), N1–C1–C4 123.26(14), C2–N3–C17 119.97(12), C24–N5–C25 119.15(13).

Minor product **6′** of the reaction between **1a**, deprotonated by MeLi, and benzoyl chloride (Scheme 5) was characterized in the solid state. It consists of two DII fragments with Dmp and an acyl substituent on exocyclic nitrogens (Figure 10). Dmp substituent is in perpendicular arrangement to the DII dimeric planar structure, and the acyl substituent, similarly to compound **6**, deviates from the plane by about 30°. Hydrogen is located on endocyclic nitrogen N4 near the acyl substituent, which allows formation of intramolecular hydrogen bond with oxygen atom. The second hydrogen bond takes place as well, this time with endocyclic isoindole nitrogen N2. Compounds **6′** as well as **6**–**9** contain a localized system of single and double bonds. With a closer look, a formation of **6′** is not that simple and consists of several steps (Figure 9). This side reaction of the preparation of **6** by the MeLi procedure is allowed only by equilibria between **1b** and its mono- and dilithiated forms during the synthesis. Twice deprotonated dilithiated complex reacts with benzoyl chloride to form compound Li**6′** with two fused DII moieties. Li**6′** exists in solution in the form of two monolithiated isomers, where the lithium atom is coordinated by two solvent molecules (THF or water). Both *E-* and *Z*-isomers coexist in a nearly equimolar ratio (Figure 9) after the reaction under inert conditions in THF-*d*_8_, presumably as bis-tetrahydrofuranates. In CD_2_Cl_2_ only the *E*-isomer exists. Both isomers of Li**6′** exhibit almost the same diffusion coefficients in both DOSY NMR spectra, recorded in CD_2_Cl_2_ and THF-*d*_*8*_, corresponding to a monomeric behavior (see Figures S43 and S44). When the complex is crystallized from diethyl ether on air, the dimer (Li**6′**)_2_·H_2_O connected by one water molecule crystallizes out of the solution (see Figure S70). Both pairs of connected DII-DII units exhibit the *E*-isomer structure with the Dmp group nearly (89.02(9)°) perpendicularly oriented toward them. The interplanar angle between DII-DII parts is 62.08(12)°, while the benzoyl groups are a bit deviated from the major plane (25.82(11)°). The lithium atoms are found in centers of pseudo square pyramidal vicinity formed by ligands in a terdentate fashion and with carbonyl groups in the bridging apical position. Bridging water molecule is found in the basement of the polyhedra. In contrast to compounds **6′** as well as **6**–**9**, the structure of compound Li**6′** contains an extensive delocalized bond system from N2 to C25 (see Figure S70), as a result of lithium atom coordination.

The equilibrium of Li**6′** was monitored by ^1^H and ^7^Li NMR spectroscopy in THF-*d*_8_ solution (Figures 9, S39, and S40). At the beginning, the *E-* and *Z*-isomers are present in a 1:1 ratio, but upon opening of the tube to the air and gradual addition of water, the equilibrium is shifted significantly toward the *E*-isomer characterized by the diagnostic signal of H4 atom (δ = 6.4), similarly as for **6** (Figure 8). When the sample is left on the air or treated with diluted aqueous HCl, CH_3_COOH or CO_2_, as a weak acid, is bubbled through the sample (see Figure 9), lithium and solvents are extruded from the structure of Li**6′**, forming the *Z*-isomer of **6′** in the solid state (Figure 10). Both possible isomers were detected in CD_2_Cl_2_ or THF-*d*_8_ solutions with more populated *Z*-isomer in ratios of 2:1 and 5:1, respectively.

### Amido Derivatives: Reduction

As the last topic, we explored a novel synthetic route to amido derivatives of reduced DIIs, which can be seen as possible precursors of hemiporphyrazines, valuable species with new metal complexation patterns, fluorescent dyes or 2D materials.^59−61^ Simple reduction of **6**–**9** (Scheme 5) by sodium borohydride in methanol gives after recrystallization from diethyl ether **6r**–**9r** in the form of etherates. Diethyl ether can be easily removed from the solid samples by dynamic vacuo. Formal hydrogenation takes place at the imino group of the diiminoisoindole instead of amide reduction. The ability of these compounds to make intramolecular H-bonds is suspended by the reduction of π-electron density and the C2 atom became sp^3^ hybridized. Also, the *E*/*Z* isomerism and tautomeric exchanges observed in previously described compounds are expected to be vanished. It is worth to be mentioned **6r**–**9r** were prepared as racemic mixtures with chiral center at C2 atom.

NMR analysis of **6r**–**9r** showed only one set of signals clearly determining the proposed structures. By the reduction, the pyrrole ring lost the key double bond needed for π-electron conjugation of both neighboring imino groups, which also led to discontinuation of the RAHB and nonexistence of any other intramolecular hydrogen bond. This is clearly reflected in ^1^H NMR spectrum, where the NH signal of H2 atom is shifted from ca. 10 to 6.7 ppm (in the case of **6r**). The loss of aromaticity is also demonstrated by a loss of color.

**Figure 11:**
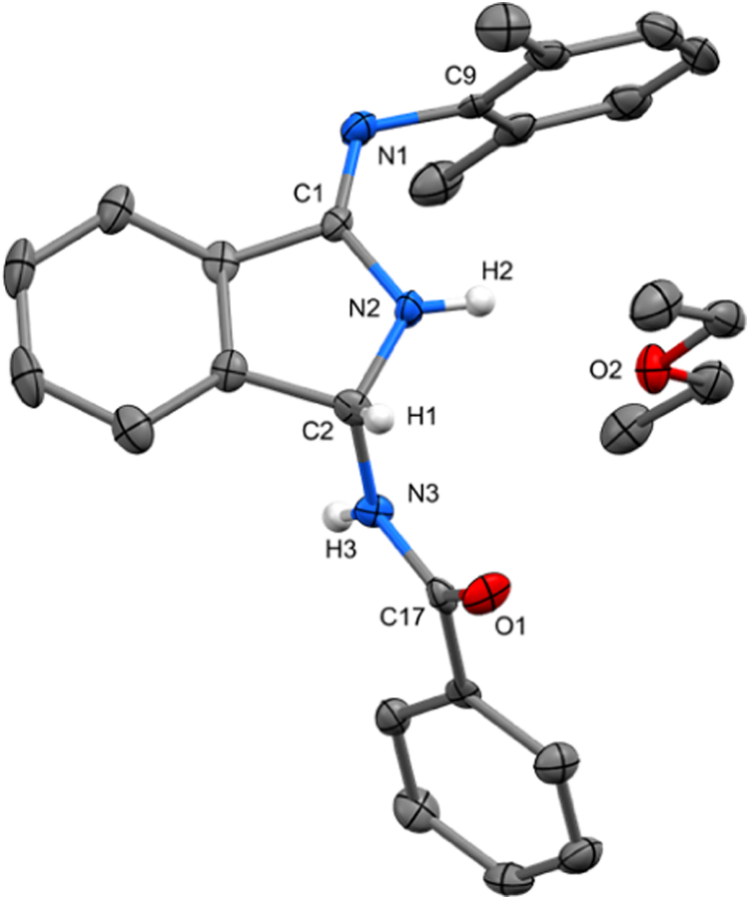
ORTEP view 50% probability level of the molecular structure of **6r**. Hydrogen atoms are omitted for clarity. Selected bond lengths (Å) and angles (deg): N1–C9 1.421(6), N1–C1 1.280(6), C1–N2 1.359(6), N2–C2 1.455(6), C2–N3 1.450(6), N3–C17 1.346(6), O1–C17 1.237(5), C1–N1–C9 117.0(4), N1–C1–C4 124.4(4), C2–N3–C17 122.7(4), N3–C17–O1 121.9(4).

In the solid state, all the compounds of this series are prone to crystallize (Figures S66–S69) in the same achiral space group *Pna*2_1_. The planarity of the reduced DII fragment was almost ideal. The Dmp substituent is oriented perpendicularly as described for less saturated **6**–**9**. However, the position of the acyl substituent is significantly different, and its deflection from the DII plane is approximately 84°. Bond length distribution differs (see Figure 11) when compared to the rest of substituted DIIs, and as a direct consequence of chemical reduction, interatomic distances in N2–C2–N3 fragments are elongated. The hydrogen bond is no longer intramolecular, but between the N3–H3 group and carbonyl oxygen atom from another DII molecule occurs the infinite chain (peptide-like) intermolecular hydrogen bond. The NH groups of DIIs are saturated by an H-bond electron transfer from the oxygen atom of diethyl ether solvate.

## Conclusions

As a contribution to the chemistry of macrocycles and reactivity of lithium amides with unsaturated systems, this work opens a new pathway to the synthesis of dissymmetrically substituted DIIs. These can be used as precursors to a large variety of phthalocyanines with variable structures. Novel types of organic compounds with preorganized structures usable as starting materials for the construction of hemiporphyrazines and similar macrocyclic compounds have been investigated. Several types of isomerisms in solution have been studied by NMR/DFT approach—*E*/*Z*-isomerization and dimerization (DII **1a** and amido derivatives **6**–**9**), tautomerism (guanidines **5aa**–**bb**) and stability both in solution and solid state. This could help the selection of appropriate precursor or ligand, solvent, and conditions when targeted applications will be quested, which is further documented by the synthesis of appropriate lithium complexes. The presence of strong resonance-assisted hydrogen bonds in a series of compounds **1a**–**e**, **2b**, **5aa**–**bb**, and **6**–**9** predetermined their use as novel ligands for coordination chemistry similarly to acetylacetonates or β-diketiminates.

## Materials and Methods

### Synthesis

Multiple manipulations and reactions were carried out under an argon atmosphere using standard Schlenk techniques (stated in the experimental procedures). Reagents were purchased from commercial suppliers (Merck or Avantor/VWR) or were already available at our laboratories. The solvents were dried and degassed using a PureSolv solvent drying system (Innovative Technology, Inc., USA). For detailed description, see the Supporting Information.

#### General Procedure for the Synthesis of Compounds Li**1a**, Li**1b**, Li**1c**, Li**1d**, and Li**1e**

Aniline was dissolved in hexane, and *n*-butyllithium was added at 0 °C. The yellow solution was stirred for another hour and concentrated in vacuo, and the yellow crystalline intermediate was isolated. Intermediate was dissolved in THF and a solution of 1,2-dicyanobenzene in THF was added while cooling to −50 °C. A dark green solution was formed, which was left to stir for 12 h, and subsequently, at a low temperature (−20 °C), the product was recrystallized from THF or a mixture of THF and petroleum ether. The product was filtered off, washed twice with hexane, and dried in vacuo. Reactions were performed under an argon atmosphere using standard Schlenk techniques.

#### General Procedure for the Synthesis of Compounds **1a**, **1b**, and **1c**

Lithium complex was dissolved in methanol on air, and 2 molar equiv of water were added. The solution was left to stir for 12 h, and then the solvent was evaporated under vacuo. The product was extracted by dichloromethane, filtrated, dried in vacuo, and isolated in the form of a yellow powder.

#### General Procedure for the Synthesis of Compounds **5aa**–**bb**

The corresponding isoindole was dissolved in 30 mL of toluene at 50 °C. Subsequently, 1 mol equiv of carbodiimide was added to the solution. The reaction mixture was stirred for 48 h at 100 °C, then cooled to room temperature, and concentrated in vacuo. The product was further washed with hexane, dried, and isolated as a yellow powder.

#### General Procedures for the Synthesis of Compounds **6**–**9**

##### Procedure A

The corresponding isoindole was dissolved in 30 mL of diethyl ether. The solution was cooled to 0 °C, and 1 mol equiv of methyllithium was added. The reaction mixture was stirred for 1 h at room temperature. Subsequently, 1 mol equiv of acyl chloride was added to the solution. The solution was stirred for 4 h with the formation of a yellow precipitate, then concentrated in vacuo to half. The product was filtrated from a solution and extracted with benzene. After evaporation of benzene in vacuo, the product was dried and isolated as a yellow powder. The reactions were carried out under an argon atmosphere using standard Schlenk techniques.

##### Procedure B

The corresponding isoindole was dissolved in 50 mL of diethyl ether. Subsequently, 1 molar equiv of acyl chloride and two equiv of triethylamine were added to the solution. The solution was stirred for 12 h and filtrated on a frit. Product was gained from the solution by slow evaporation of diethyl ether. After filtration, the product was dried and isolated as yellow crystals.

#### General Procedure for the Reduction of Compounds **6**–**9**

To a mixture of isoindole and sodium tetraborohydride, hexane was added, the suspension was cooled down to −30 °C, and 0.250 mL of methanol was added. The solution was left to stir for 2 h. Then, the solution was filtrated from a highly viscous byproduct, and the solvent was evaporated under vacuo. The product was dried in vacuo and isolated in the form of a pale-yellow powder. Reactions were carried out under an argon atmosphere using standard Schlenk techniques.

### NMR Spectroscopy

NMR experiments were recorded on a Bruker Avance III spectrometer equipped with a broad-band cryo-probe with an ATM module (5 mm CPBBO BB-^1^H/^19^F/^15^N/D Z-GRD) operating at 499.98 MHz for ^1^H, 125.73 MHz for ^13^C and 50.67 for ^15^N; and also on a Bruker Avance III 600 spectrometer equipped with an inverse triple resonance cryo-probe with ATM module (5 mm CPTCI ^1^H/^13^C/^15^N/D Z-GRD) operating at 600.13 MHz for ^1^H and 60.82 MHz for ^15^N. Low-temperature NMR spectra were recorded on a Bruker Avance II spectrometer with a triple resonance broad-band probe with ATM (5 mm PATBO BB-^1^H/^19^F/D Z-GRD) operating at 499.94 MHz for ^1^H and 125.72 MHz for ^13^C. For NMR signal assignment, standard Bruker pulse sequences were employed for both 1D (^1^H, ^13^C-APT) and 2D (COSY, ROESY, HSQC, HMBC) NMR experiments at a corrected temperature. All NMR data was interpreted using Topspin 3.5. For reference, the following solvent signals were used: DMSO-*d*_6_: 2.50 (^1^H) and 39.5 (^13^C) ppm; THF-*d*_8_: 3.57 (^1^H) and 67.57 (^13^C) ppm, toluene-*d*_8_: 2.08 (^1^H) and 20.43 (^13^C) ppm. The solutions were obtained by dissolving approximately 20 mg of each compound in 0.6 mL of a deuterated solvent.

The DOSY spectra were acquired in 5 mm NMR tubes, and all of the experiments were performed at 25 °C and without sample spinning to avoid convection. All DOSY experiments were performed using a standard Bruker pulse sequence, dstebpgp3s, a double-stimulated echo sequence with bipolar gradient pulses and three spoil gradients with convection compensation. The diffusion time was 0.1 s (*D*). The duration of the magnetic field pulse gradients was adjusted for each polymer in the range of 500–2000 ms (*d*/2). The delay for gradient recovery was 0.2 ms, and the eddy current delay was 5 ms. For each DOSY NMR experiment, a series of 16 spectra on 32 000 data points were collected. The pulse gradients were incremented from 2 to 98% of the maximum gradient strength in a linear ramp with a total experiment time of 23 min. The temperature was set and controlled at 295 K with an air flow of 400 L/h in order to avoid any temperature fluctuations due to sample heating during the magnetic field pulse gradients. After Fourier transformation and baseline correction, the diffusion dimension was processed with Topspin 3.6.1 software and Dynamic Center 2.4.4.

### Mass Spectrometry

High-resolution EI spectra were measured using an Agilent 7250 GC/Q-TOF mass spectrometer (Agilent). The conditions were optimized for suitable ionization in the source (electron voltage of 70 V, source temperature of 230 °C). The sample was applied either by direct injection or using an attached GC module (column DB-5, 30 m; flow rate of helium 1 mL/min).

High-resolution ESI spectra were measured using an LTQ Orbitrap XL (Thermo Fisher Scientific). The conditions were optimized for suitable ionization in the source (capillary voltage 9 V, tube lens voltage 150 V, temperature 275 °C). The sample was applied by direct injection in positive, and the mobile phase was 80% MeOH with the same flow rate.

### sc-XRD

Full sets of diffraction data were collected at 150(2) K with a Bruker D8-Venture diffractometer equipped with Cu (Cu/Kα radiation; λ = 1.54178 Å) or Mo (Mo/Kα radiation; λ = 0.71073 Å) microfocus X-ray (IμS) source, Photon I or III CMOS detectors, and Oxford Cryosystems cooling device was used for data collection. Some data were collected at the same conditions with a Nonius KappaCCD diffractometer with Mo Kα radiation (λ = 0.71073 Å), a graphite monochromator, and the ϕ and χ scan mode. The frames were integrated with the Bruker SAINT software package using a narrow frame algorithm. Data were corrected for absorption effects using the multiscan method (SADABS).^62^ Obtained data were treated by XT-versions 2014/5, SHELXT 2018/2,^63^ and SHELXL-2018/3 software^64^ implemented in APEX3/APEX4 (Bruker AXS) system.^65^ The hydrogen atoms were placed in calculated positions and refined in the “riding model”. Some H atoms were localized on a difference Fourier map. Heavy atoms were refined anisotropically. Hydrogen atoms were mostly located on the difference Fourier map; however, for the final solution of the crystal structure, all hydrogen atoms were recalculated into ideal positions (riding model) according to the assigned temperature factors *H*_iso_(*H*) = 1.2 U_eq_ for aryl groups and *H*_iso_(*H*) = 1.5 Ueq for aliphatic groups with C–H bond lengths = 0.96; 0.97; 0.98; and 0.93 Å for methyl, methylene, methine, and hydrogen atoms of aromatic rings, respectively, 0.86 or 0.82 Å for N–H or O–H bonds. For some of the 26 crystal structures, only small weakly diffracting crystals were grown, which caused the B alerts in the checkcif evaluation procedure (Li**3b″**, **5bb**, and **8r**). Structures of **5ba** and Li**6′** contain residual electron density (<1 e Å^–3^) in the area of solvent molecules, which result in the B alerts in checkcif evaluation procedure. The solvent molecules in Li**1e** and Li**3b′** were masked by SQUEEZE procedure. In **1c**, the RAHB contact caused a separation of molecules within the dimer, which caused the A alert for a not properly connected set of atoms. All mentioned phenomena producing respective alerts have no significant influence on the quality of the structure determination.

Crystallographic data for structural analysis of all compounds have been deposited with the Cambridge Crystallographic Data Centre, CCDC nos. 2417495–2417520. Copies of this information may be obtained free of charge from The Director, CCDC, 12 Union Road, Cambridge CB2 1EY, U.K. (fax: +44-1223-336033; e-mail: deposit@ccdc.cam.ac.uk or www: http://www.ccdc.cam.ac.uk).

### DFT Calculations and QTAIM Analysis

All of the calculations were performed with the Gaussian 16 program.^66^ The structures were optimized at the DFT level of theory using the B3LYP^67,68^ functional and a standard 6-31g(d,p) basis set with the polarizable continuum model (PCM) used for implicit tetrahydrofuran solvation.^69,70^ Transition-state (TS) structures of the reaction were found using TS Berny algorithm^71^ and QST3^72,73^ approach, where the structures of the reactant, product, and estimated TS were used as input for the TS search. The vibrational frequencies and free energies were calculated for all of the optimized structures, and the stationary-point character (a minimum or a first-order saddle point) was thus confirmed. The NMR parameters were calculated using the GIAO^174^ method with the 6-311+g(d,p) basis set with PCM. Calculations associated with the dimerization and isomerization mechanism of **1a** were performed at the B3LYP-D3(BJ)/6-311+g(d,p)/PCM(THF) level of theory, dispersion corrections were considered, employing the D3 version of Grimme’s dispersion method.^74^

### IR Spectroscopy

Infrared (single-bounce diamond attenuated total reflection (ATR)) and Raman (vacuum-sealed capillary excitation laser 1064 nm) spectra were recorded on a Nicolet iS50 FTIR spectrometer equipped with an iS50 Raman module.

### UV–Vis Spectroscopy

Electronic absorption spectra (195–1100 nm) were obtained on a Maya2000 Proconcave grating spectrometer using transmission cell with an optical path 10 mm with THF and toluene as a solvent at room temperature.

## Supporting Information Available

Synthetic procedures, explanation of structures and equilibria, cif files (PDF)
