# Supplementary material for: Addition of Lithium Silylamides to 1,2-Dicyanobenzene: Isoindoline-1,3-diimine Derivatives Investigated by NMR/XRD/DFT Approach
Source: Inorg Chem. 2025 Apr 8;64(15):7592–606. doi: 10.1021/acs.inorgchem.5c00573 (PMC12015960; doi:10.1021/acs.inorgchem.5c00573)
Supplement: Supplementary file 1 — ic5c00573_si_001.pdf [file ic5c00573_si_001.pdf]

Supporting Infromation

Addition of Lithium Silylamides to 1,2-dicyanobenzene; Isoindoline-1,3-diimine Derivatives Investigated by NMR/XRD/DFT Approach

Stanislava Majerová,† Tomáš Chlupatý,† Maksim A. Samsonov,† Josef Cvačka,‖ Eliška Procházková,‖* Aleš Růžička†*

†Department of General and Inorganic Chemistry, Faculty of Chemical Technology, University of Pardubice, Studentská 573, Pardubice 532 10, Czech Republic

‖Institute of Organic Chemistry and Biochemistry, Czech Academy of Sciences, Flemingovo nám. 2, Prague 160 00, Czech Republic

*Eliška Procházková –prochazkova@uochb.cas.cz, Aleš Růžička – ales.ruzicka@upce.cz

Table of content

1 Experimental Procedures S3

2 DFT calculations S5

2.1 Investigation of E/Z isomerism for compound **1a** S5

2.2 Investigation of dimerization process for compound **1a** S6

3 NMR/DFT correlations S7

3.1 Investigation of tautomeric equilibria for compounds **5aa** – **5bb** by ^{13}C NMR combined with DFT calculations S7

3.2 Investigation of tautomeric equilibria for compounds **5aa** – **5bb** by ^{15}N NMR combined with DFT calculations S11

3.3 Investigation of isomer equilibria for compounds **6** – **9** by ^{13}C NMR and DFT data correlations S15

4 NMR spectroscopy S26

4.1 E/Z-isomerisation of complex **Li6'** promoted by water addition S26

4.2 DOSY NMR spectroscopy S28

4.3 $^{1}H,^{15}N$-HSQC NMR spectroscopy S32

5 Solid state structure determination S33

6 General methods, preparation and characterization of compounds S48

6.1 General procedure for synthesis of compounds **Li1a**, **Li1b**, **Li1c**, **Li1d** and **Li1e** S48

6.2 General procedure for synthesis of compounds **1a**, **1b** and **1c** S50

6.3 General procedure for the synthesis of compounds **5aa** – **5bb** S51

6.4 General procedures for synthesis compounds **6** – **9** S54

6.5 General procedure for reduction compounds **6** – **9** S57

7 Crystal data and structure refinements of prepared compounds S62

8 NMR spectra of prepared compounds S88

9 IR spectra of prepared compounds S131

10 UV-Vis spectra of prepared compounds S137

11 Mass spectra of prepared compounds S139

12 References S141

1 Experimental Procedures

Synthesis

Multiple manipulations and reactions were carried out under an argon atmosphere using standard Schlenk techniques (stated in experimental procedures). Reagents were purchased from commercial suppliers (Merck or Avantor/VWR) or were already available at our labs. The solvents were dried and degassed using PureSolv™ solvent drying system (Innovative Technology Inc., USA).

NMR spectroscopy

NMR experiments were performed on a Bruker Avance III spectrometer equipped with a broad-band cryo probe with an ATM module (5 mm CPBBO BB-1H/19F/15N/D Z-GRD) operating at 499.98 MHz for ^{1}H, 125.73 MHz for ^{13}C and 50.67 for ^{15}N; and also on a Bruker Avance III 600 spectrometer equipped with an inverse triple resonance cryo-probe with ATM module (5 mm CPTCI 1H/13C/15N/D Z-GRD) operating at 600.13 MHz for ^{1}H and 60.82 MHz for ^{15}N. Low-temperature NMR spectra were recorded on a Bruker Avance II spectrometer with a triple resonance broad-band probe with ATM (5 mm PATBO BB−1H/19F/D Z-GRD) operating at 499.94 MHz for ^{1}H and 125.72 MHz for ^{13}C. For NMR signal assignment, standard Bruker pulse sequences were employed for both 1D (^{1}H, ^{13}C-APT) and 2D (COSY, ROESY, HSQC, HMBC) NMR experiments at a corrected temperature. All NMR data was interpreted using Topspin 3.5. For reference, the following solvent signals were used: DMSO-d_6: 2.50 (^{1}H) and 39.5 (^{13}C) ppm; THF-d_8: 3.57 (^{1}H) and 67.57 (^{13}C) ppm, toluene-d_8: 2.08 (^{1}H) and 20.43 (^{13}C) ppm. The solutions were obtained by dissolving approximately 20 mg of each compound in 0.6 mL of the deuterated solvent.

For compounds of the series **1a** – **4** (diiminoisoindols), **5aa** – **5bb** (guanidines) and **6** – **9** (amides), two sets of signals, referred to as majorit and minorit, were recorded in various solvents. The explanation of such an appearance is given in the main text.

The DOSY spectra were acquired in 5 mm NMR tubes and all the experiments were performed at 25°C and without sample spinning to avoid convection. All DOSY experiments were performed using standard Bruker pulse sequence - dstebpgp3s - a double stimulated echo sequence with bipolar gradient pulses and three spoil gradients with convection compensation. The diffusion time was 0.1 s (D). The duration of the magnetic field pulse gradients was adjusted for each polymer in a range of 500–2000 ms (d/2). The delay for gradient recovery was 0.2 ms and the eddy current delay 5 ms. For each DOSY-NMR experiment, a series of 16 spectra on 32 K data points were collected. The pulse gradients were incremented from 2% to 98% of the maximum gradient strength in a linear ramp with a total experiment time of 23 min. The temperature was set and controlled at 295 K with an air flow of 400 L/h in order to avoid any temperature fluctuations due to sample heating during the magnetic field pulse gradients. After Fourier transformation and baseline correction, the diffusion dimension was processed with the Topspin 3.6.1 software and Dynamic Center 2.4.4.

Mass spectrometry

High resolution EI spectra were measured using Agilent 7250 GC/Q-TOF mass spectrometer (Agilent). The conditions were optimized for suitable ionization in the source (electron voltage 70V, source temperature 230 °C). The sample was applied either by direct injection or using attached GC module (column DB-5, 30 m; flow rate of helium 1 ml/min).

High resolution ESI spectra were measured using LTQ Orbitrap XL (Thermo Fisher Scientific). The conditions were optimized for suitable ionization in the source (capillary voltage 9V, tube lens voltage 150V, temperature 275 °C). The sample was applied by direct injection in positive and the mobile phase was 80% MeOH with the same flow rate.

sc-XRD

Full-sets of diffraction data were collected at 150(2)K with a Bruker D8-Venture diffractometer equipped with Cu (Cu/Kα radiation; λ =1.54178 Å) or Mo (Mo/Kα radiation; λ = 0.71073 Å) microfocus X-ray (IμS) source, Photon I or III CMOS detectors and Oxford Cryosystems cooling device was used for data collection. Some data were collected at the same conditions at Nonius KappaCCD diffractometer with Mo K_α radiation (λ = 0.71073 Å), a graphite monochromator, and the ϕ and χ scan mode. The frames were integrated with the Bruker SAINT software package using a narrow frame algorithm. Data were corrected for absorption effects using the Multi-Scan method (SADABS).[1] Obtained data were treated by XT-versions 2014/5, SHELXT 2018/2[2] and SHELXL-2018/3 software[3] implemented in APEX3 / APEX4 (Bruker AXS) system.[4] The hydrogen atoms were placed in calculated positions and refined in the "riding model". Some H atoms were localized on a difference Fourier map. Heavy atoms were refined anisotropically. Hydrogen atoms were mostly located on the difference Fourier map, however, for the final solution of the crystal structure, all hydrogen atoms were recalculated into ideal positions (riding model) according to the assigned temperature factors Hiso(H) = 1.2 Ueq for aryl groups and Hiso(H) = 1.5Ueq for aliphatic groups with C-H bond lengths = 0.96; 0.97; 0.98 and 0.93 Å for methyl, methylene, methine and hydrogen atoms of aromatic rings, respectively 0.86 or 0.82 Å for N-H or O-H bonds. For some of the 26 crystal structures only small weakly diffracting crystals were grown, which caused the B alerts in checkcif evaluation procedure (Li**3b''**, **5bb** and **8r**). Structures of **5ba** and Li**6'** contain residual electron density (<1 e$Å^{-3}$) in the area of solvent molecules, which result in the B alerts in checkcif evaluation procedure. The solvent molecules in Li**1e** and Li**3b'** were masked by SQUEEZE procedure. In **1c**, the RAHB contact caused a separation of molecules within the dimer, which caused the A alert for not properly connected set of atoms. All mentioned phenomena producing respective alerts have no significant influence to the quality of the structure determination.

Crystallographic data for structural analysis of all compounds have been deposited with the Cambridge Crystallographic Data Centre, CCDC nos. 2417495–2417520. Copies of this information may be obtained free of charge from The Director, CCDC, 12 Union Road, Cambridge CB2 1EY, UK (fax: +44-1223-336033; e-mail: deposit@ccdc.cam.ac.uk or www: http://www.ccdc.cam.ac.uk).

DFT calculations and QTAIM analysis

All the calculations were performed with the Gaussian 16 program.[5] The structures were optimized at the DFT level of theory using the B3LYP[6,7] functional and a standard 6-31g(d,p) basis set with the polarizable continuum model (PCM) used for implicit tetrahydrofuran solvation.[8,9] Transition state (TS) structures of the reaction were found using TS Berny algorithm[10] and QST3[11,12] approach; where the structures of the reactant, product, and estimated TS were used as input for the TS search. The vibrational frequencies and free energies were calculated for all of the optimized structures, and the stationary-point character (a minimum or a first-order saddle point) was thus confirmed. The NMR parameters were calculated using the GIAO method with the 6-311+g(d,p) basis set with PCM. Calculations associated with the dimerization and isomerization mechanism of **1a** were performed at the B3LYP-D3(BJ)/6-311+g(d,p)/PCM(THF) level of theory, dispersion corrections were considered, employing the D3 version of Grimme's dispersion method.[13]

IR spectroscopy

Infrared (single-bounce diamond ATR) and Raman (vacuum-sealed capillary excitation laser 1064 nm) spectra were recorded on a Nicolet iS50 FTIR spectrometer equipped with the iS50 Raman module.

UV-VIS spectroscopy

Electronic absorption spectra (195−1100 nm) were obtained on Maya2000 Proconcave grating spectrometer using transmission cell with optical path 10 mm with THF and toluene as a solvent at room temperature.

2 DFT calculations

2.1 Investigation of E/Z isomerism for compound 1a

Compound **1a** can potentially exhibit a wide range of isomers due to the different orientations of the phenyl ring (*E*/*Z*) and the relative positions of hydrogen atoms. This phenomenon has already been described as dynamics in solution and is influenced by various factors such as temperature and the nature of the solvent.[14] During the study, crystallographic data were collected, which allowed us to determine the specific structure of the isomer in the solid state. Based on these data, we performed theoretical calculations at the B3LYP-D3(BJ)/6-311+g(d,p)/PCM(THF) level of theory to examine the processes of isomerization and dimerization of compound **1a**.

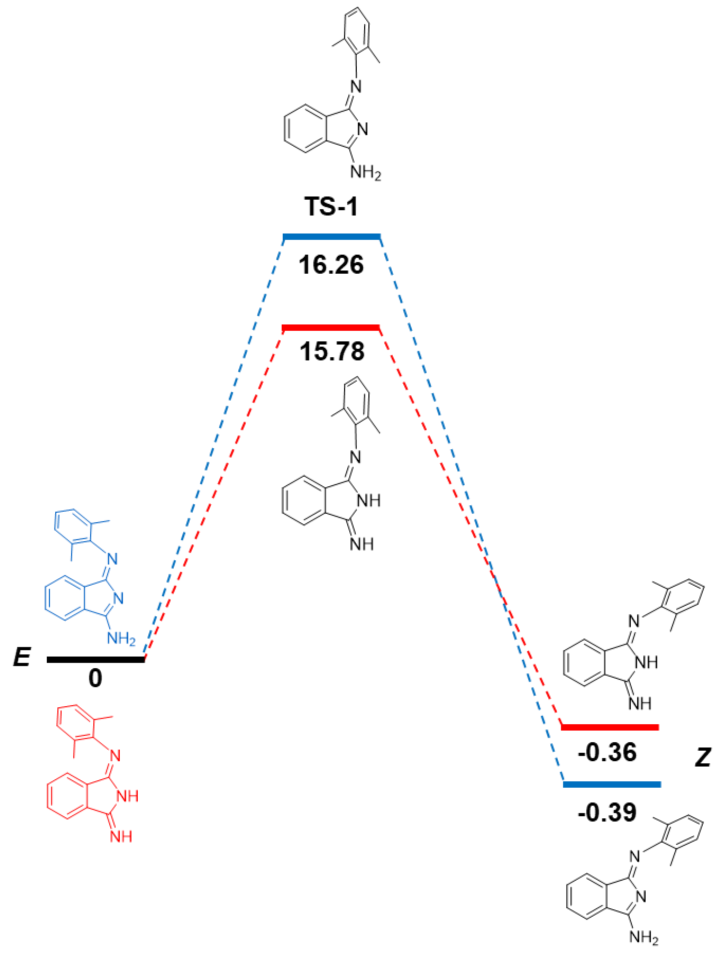

Figure S1. The DFT-estimated Gibbs' free energy profile (kcal/mol) for the isomerization of **1a**.

2.2 Investigation of dimerization process for compound 1a

The mechanism for dimer formation from two **1a** (NH_2) molecules was modeled for three possible isomer combinations: *ZZ, EE, and ZE* isomers (Fig. S2). Based on the data obtained, the most energetically favorable dimerization is the *ZZ* configuration, followed by the *ZE* variant, while the *EE* configuration is the least favorable. It is worth noting that the geometry of the final *ZZ*-NH-dimer is not planar, with the angle between the planes of the central five-membered rings being 78.6°, which aligns well with the experimental data of compound **1c**. Attempts to optimize a planar geometry for the *ZZ*-NH-dimer were unsuccessful. In contrast, the geometry of the *EE*-NH-dimer is planar, whereas for the *ZE*-NH-dimer, it was possible to optimize both planar and non-planar configurations. Thus, all three mechanisms, or a combination, could be realized.

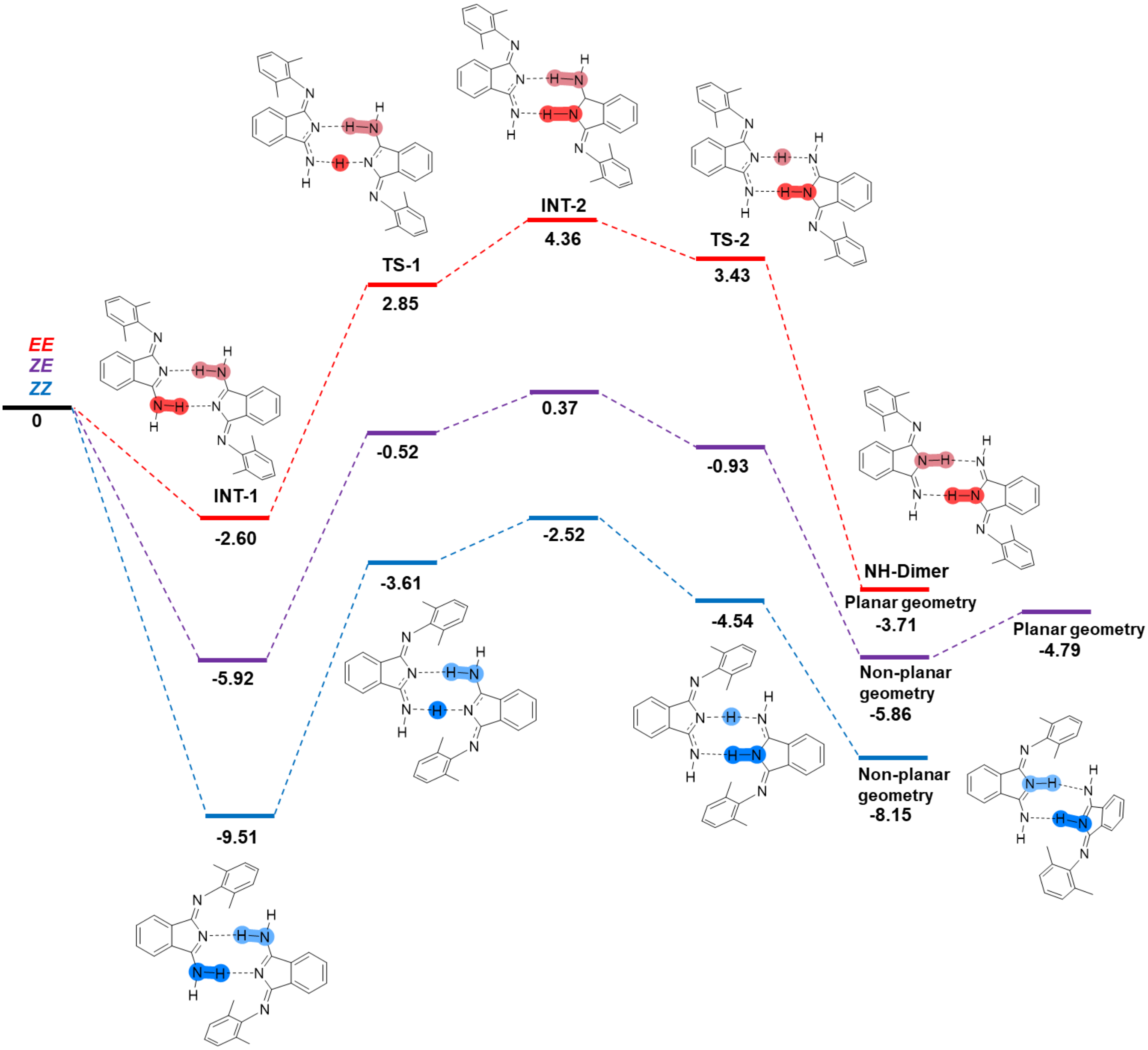

Figure S2. The DFT-estimated Gibbs' free energy profile (kcal/mol) for the dimerization process of **1a** from different isomers.

3 NMR/DFT correlations

3.1 Investigation of tautomeric equilibria for compounds 5aa – 5bb by ^{13}C NMR combined with DFT calculations

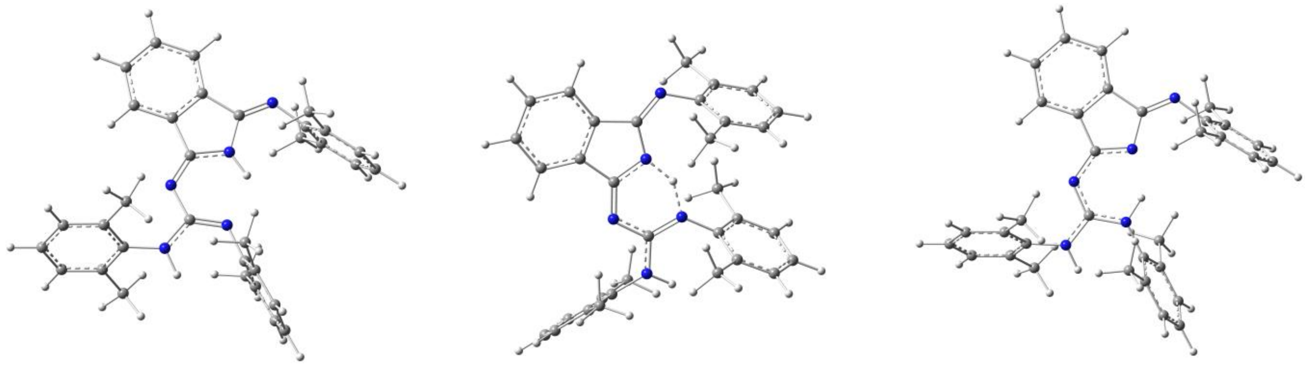

2,4-tautomer **TS** **4,5-tautomer**

Figure S3. Optimized structures and transition state for compound **5aa**.

Table S1. Relative energy for tautomers of compound **5aa**.

	2,4-tautomer	TS	4,5-tautomer
ΔG [kcal/mol]	0	3.21	0.18

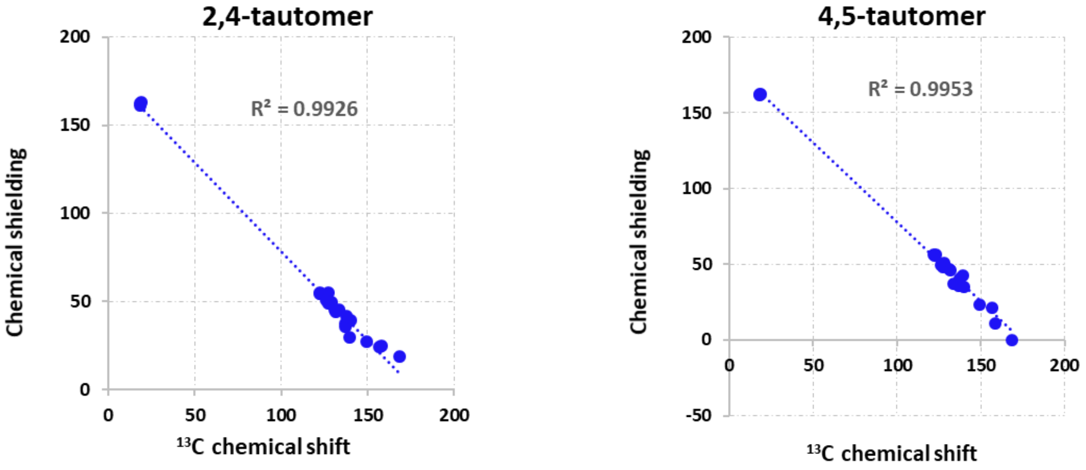

Figure S4. Correlation between experimental ^{13}C chemical shift of **5aa** with calculated shielding constants of 2,4-tautomer (left) and 4,5-tautomer (right).

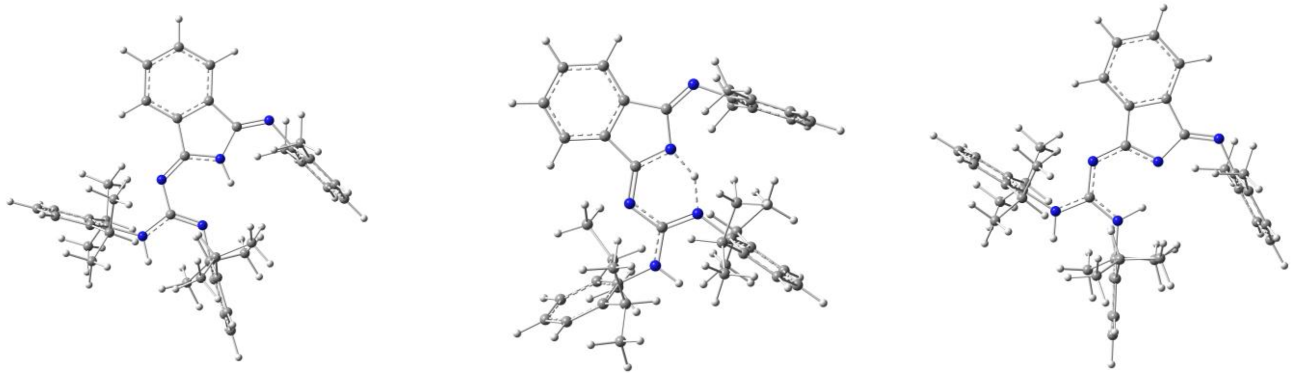

2,4-tautomer **TS** **4,5-tautomer**

Figure S5. Optimized structures and transition state for compound **5ab**.

Table S2. Relative energy for tautomers of compound **5ab.**

	2,4-tautomer	TS	4,5-tautomer
**ΔG [kcal/mol]**	0.30	5.03	0

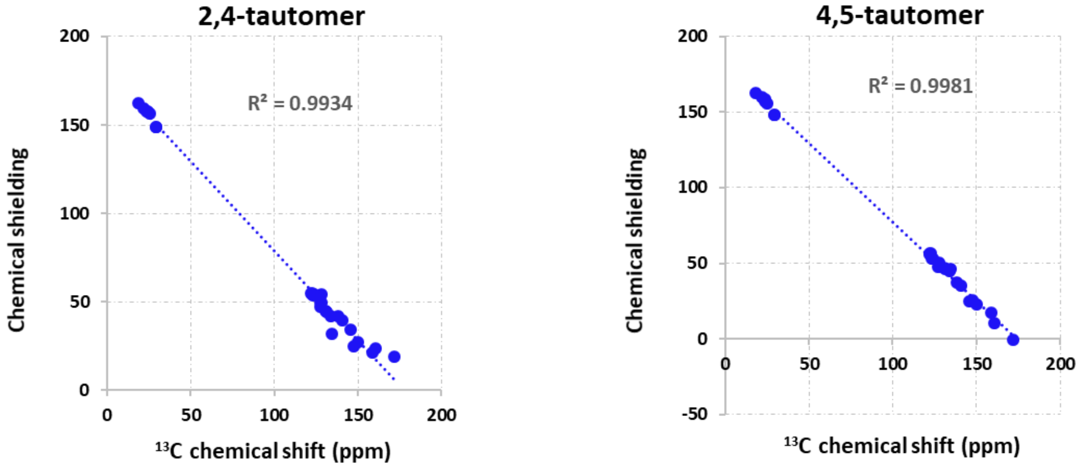

Figure S6. Correlation between experimental ^{13}C chemical shift of **5ab** with calculated shielding constants of 2,4-tautomer (left) and 4,5-tautomer (right).

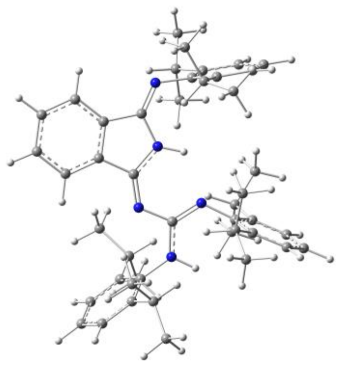

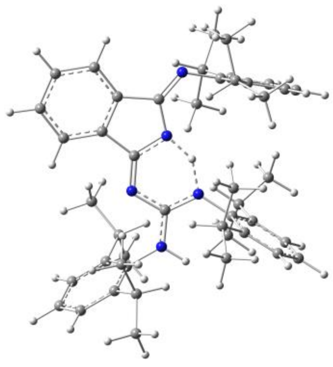

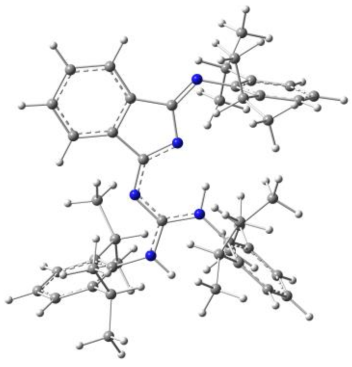

2,4-tautomer **TS** **4,5-tautomer**

Figure S7. Optimized structures and transition state for compound **5bb**.

Table S3. Relative energy for tautomers of compound **5bb**.

	2,4-tautomer	TS	4,5-tautomer
**ΔG [kcal/mol]**	0	5.36	0.29

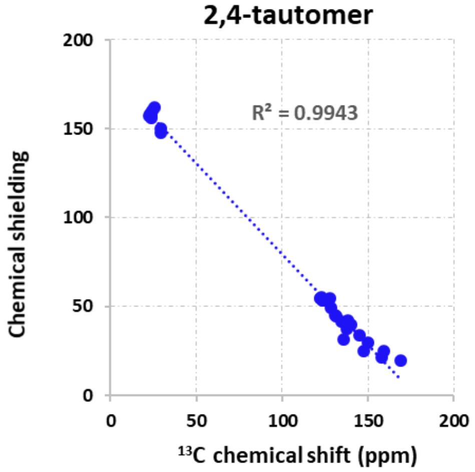

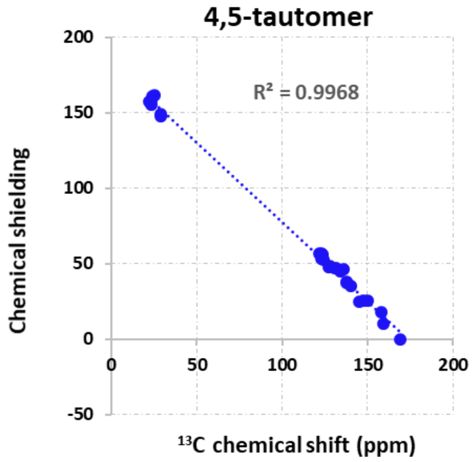

Figure S8. Correlation between experimental ^{13}C chemical shift of **5bb** with calculated shielding constants of 2,4-tautomer (left) and 4,5-tautomer (right).

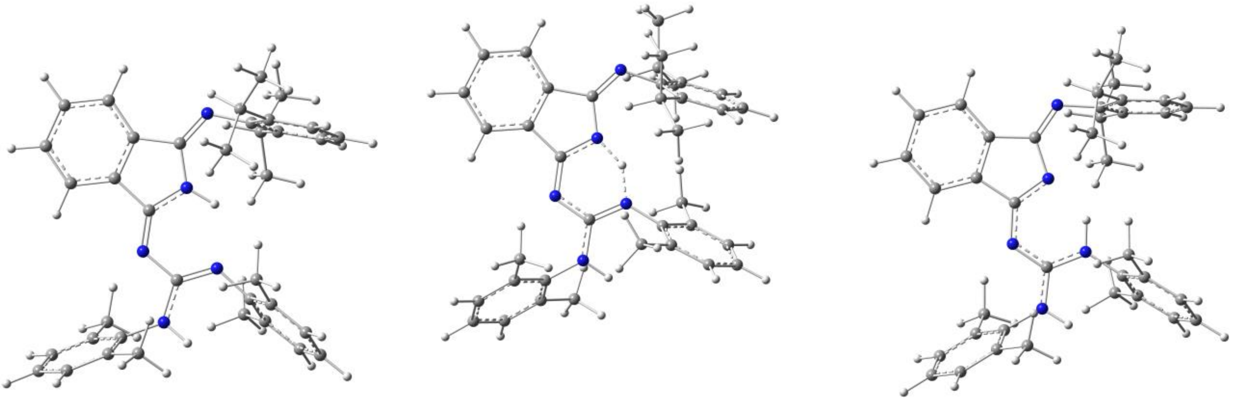

2,4-tautomer **TS** **4,5-tautomer**

Figure S9. Optimized structures and transition state for compound **5ba**.

Table S4. Relative energy for tautomers of compound **5ba**.

	**2,4-tautomer**	**TS**	**4,5-tautomer**
**ΔG [kcal/mol]**	0	4.46	0.56

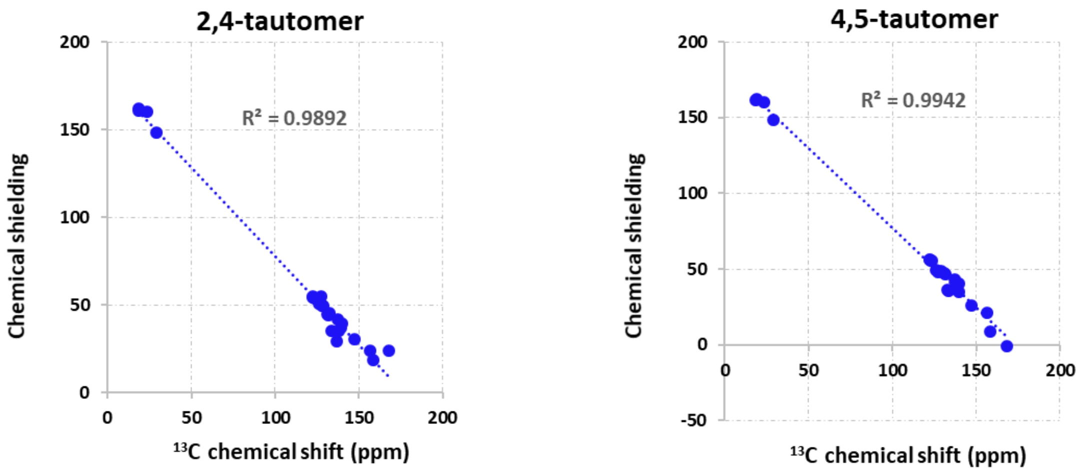

Figure S10. Correlation between experimental ^{13}C chemical shift of **5ba** with calculated shielding constants of 2,4-tautomer (left) and 4,5-tautomer (right).

3.2 Investigation of tautomeric equilibria for compounds 5aa – 5bb by ^{15}N NMR combined with DFT calculations

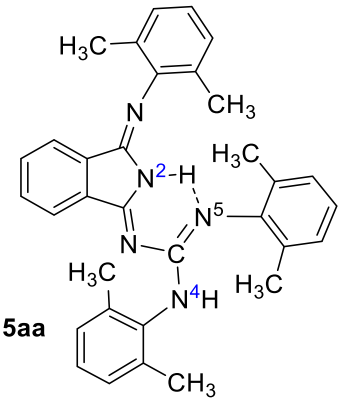

5aa

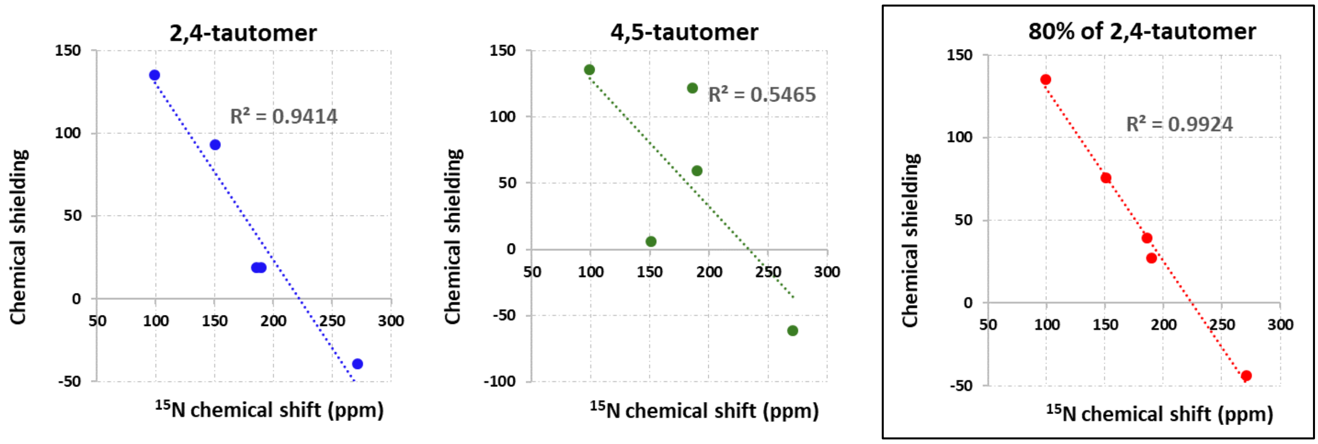

Figure S11. Correlation between experimental ^{15}N chemical shift of **5aa** with calculated shielding constants of 2,4-tautomer (left), 4,5-tautomer (middle) and averaged values based on Boltzmann distribution (right).

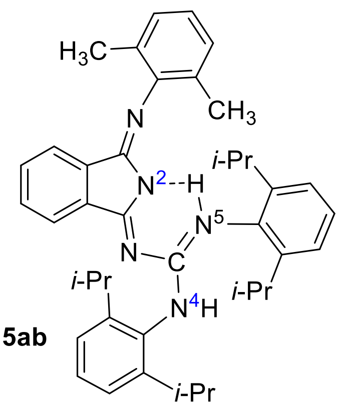

5ab

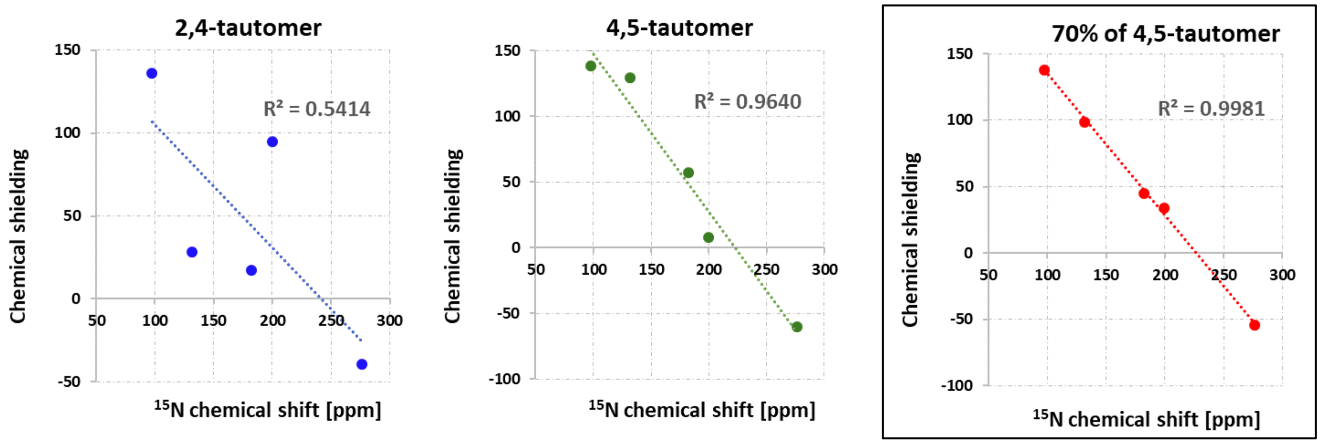

Figure S12. Correlation between experimental ^{15}N chemical shift of **5ab** with calculated shielding constants of 2,4-tautomer (left), 4,5-tautomer (middle) and averaged values based on Boltzmann distribution (right).

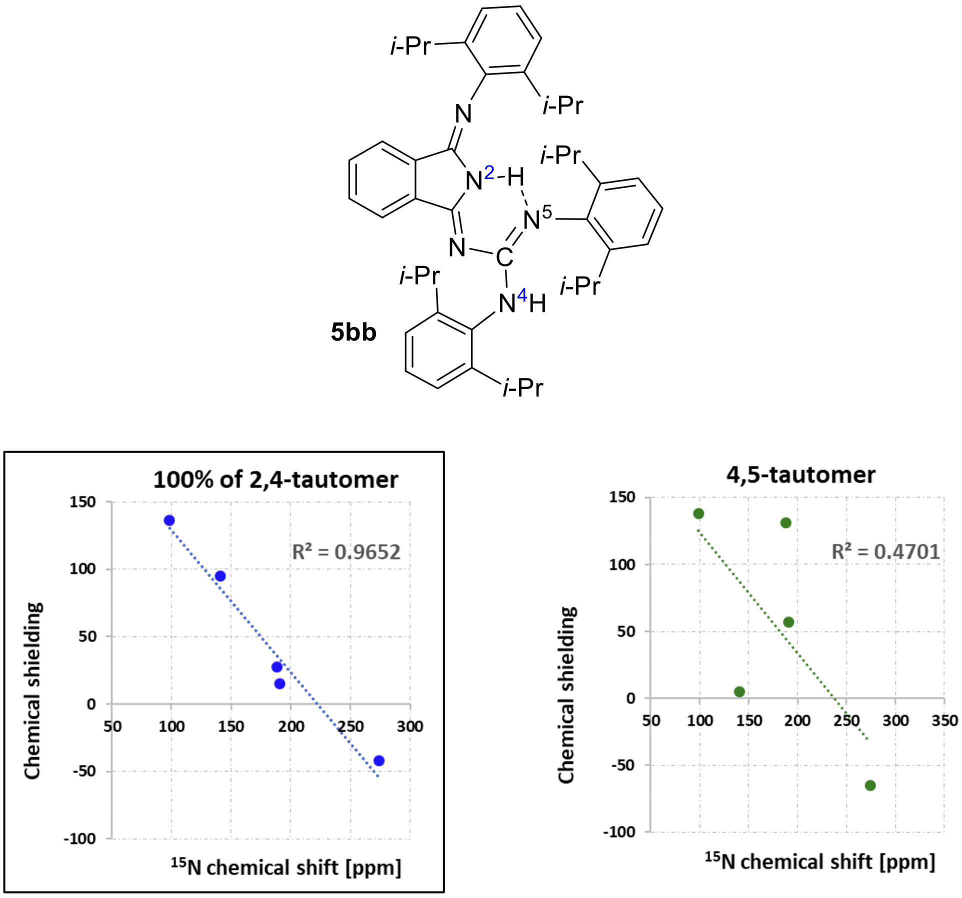

Figure S13. Correlation between experimental ^{15}N chemical shift of **5bb** with calculated shielding constants of 2,4-tautomer (left) and 4,5-tautomer (right).

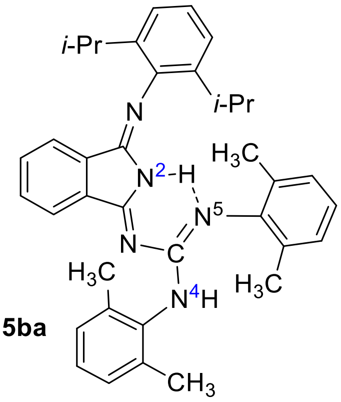

5ba

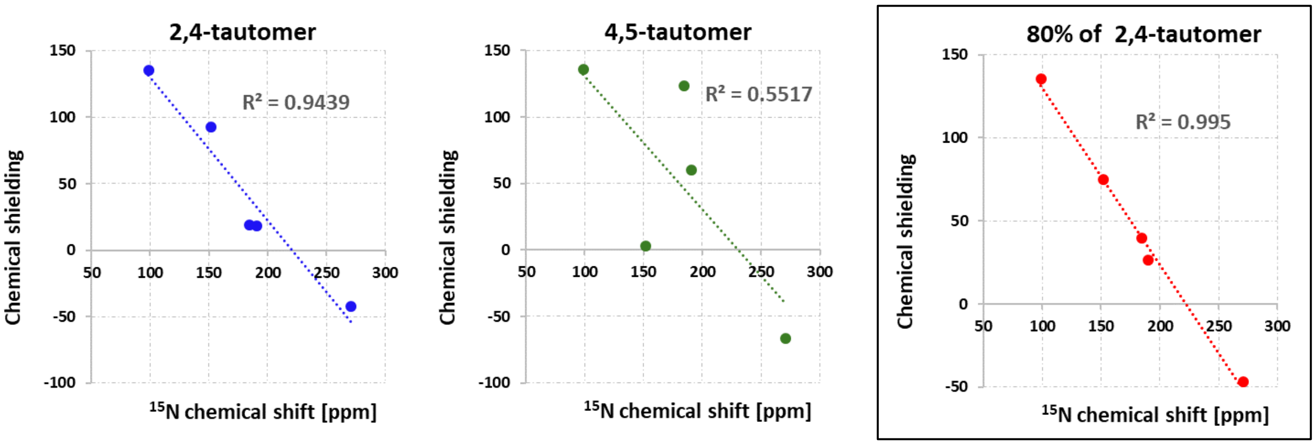

Figure S14. Correlation between experimental ^{15}N chemical shift of **5ba** with calculated shielding constants of 2,4-tautomer (left), 4,5-tautomer (middle) and averaged values based on Boltzmann distribution (right).

3.3 Investigation of isomer equilibria for compounds 6 – 9 by ^{13}C NMR and DFT data correlations

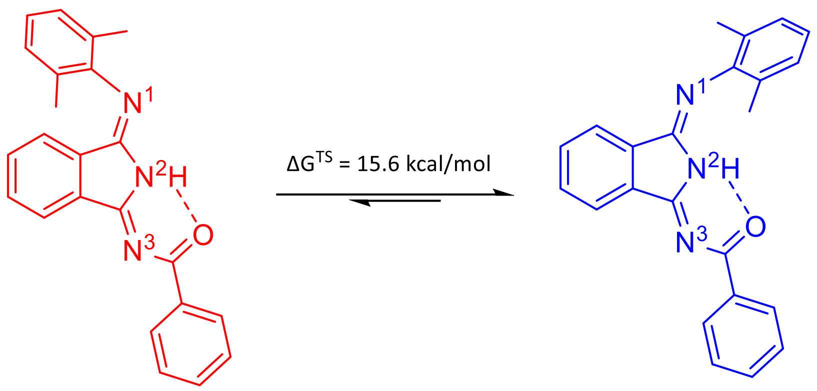

Table S5. Relative energy for isomers of compound **6** (*E* in red, *Z* in blue).

isomer	*Z*-N2-isomer	*E*-N2-isomer	*Z*-N1-isomer	*Z*-N3-isomer	*E*-OH-isomer
ΔG [kcal/mol]	0	1.21	9.55	10.76	11.65

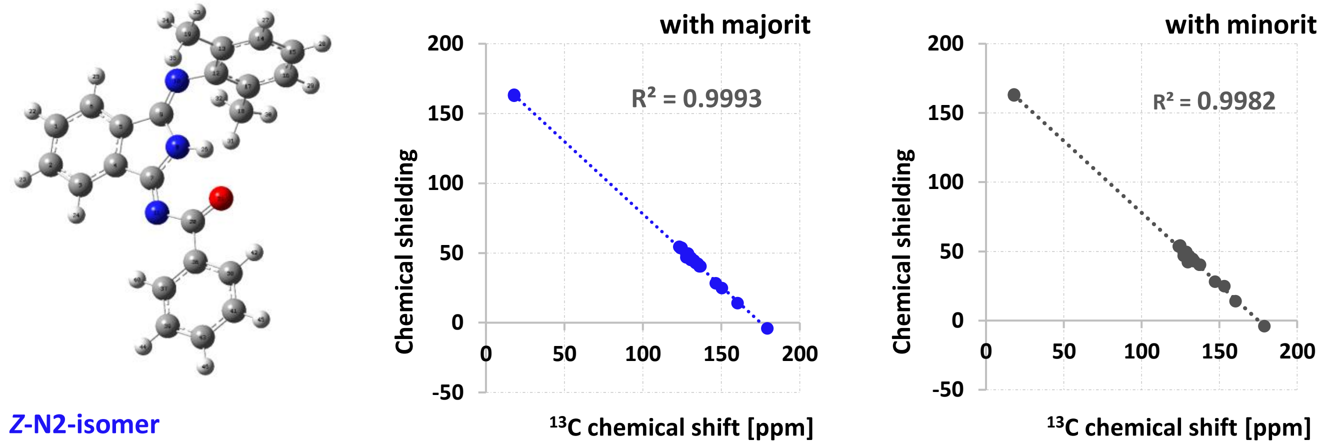

Figure S15. Correlation between experimental ^{13}C chemical shift of **6** (major data set) and (minor data set) with calculated shielding constants of *Z*-N2-isomer.

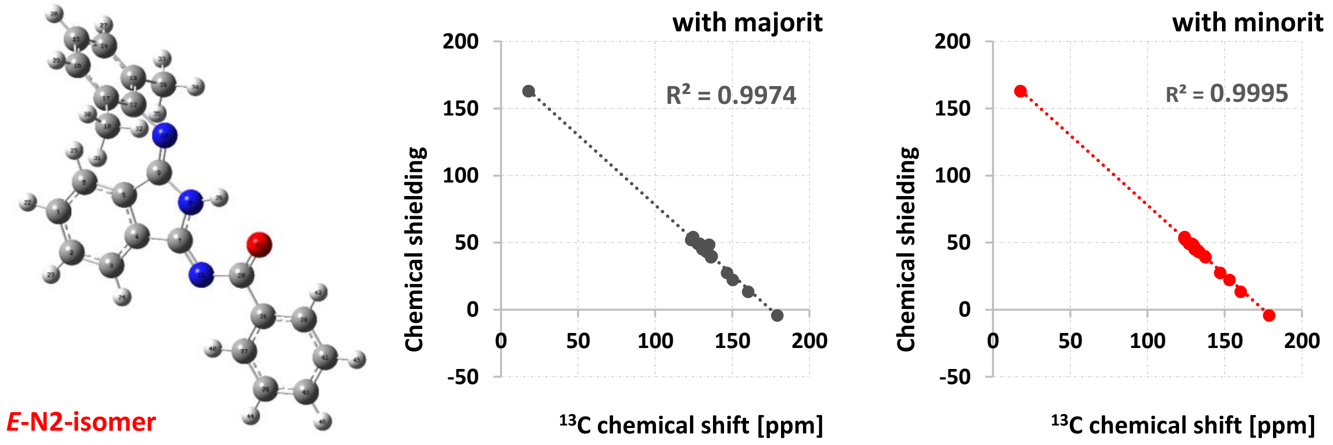

Figure S16. Correlation between experimental ^{13}C chemical shift of **6** (major data set) and (minor data set) with calculated shielding constants of *E*-N2-isomer.

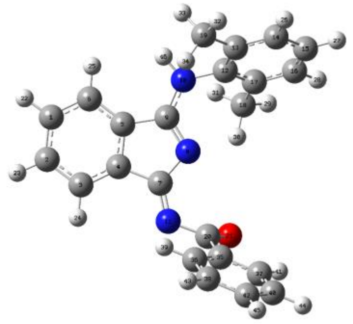

Z-N1-isomer

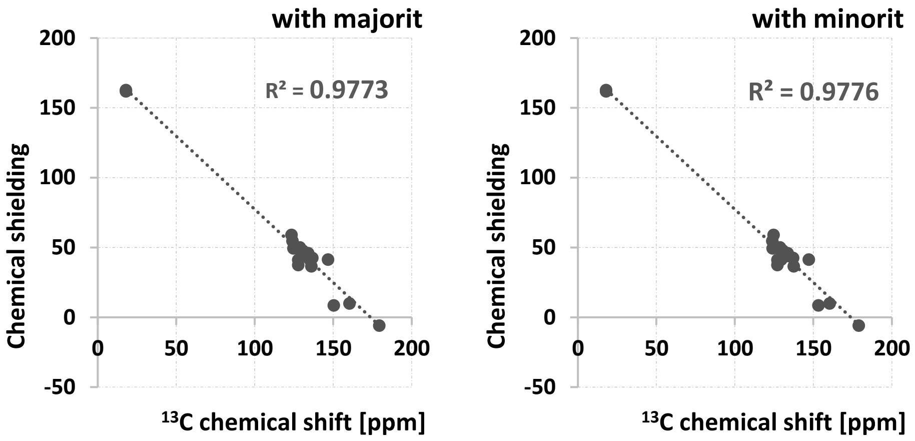

Figure S17. Correlation between experimental ^{13}C chemical shift of **6** (major data set) and (minor data set) with calculated shielding constants of *Z*-N1-isomer.

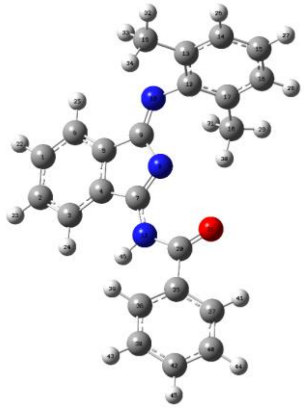

Z-N3-isomer

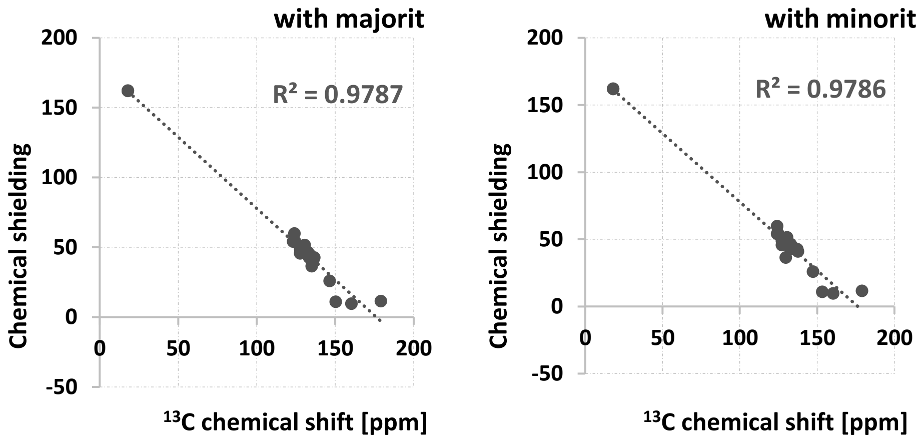

Figure S18. Correlation between experimental ^{13}C chemical shift of **6** (major data set) and (minor data set) with calculated shielding constants of *Z*-N3-isomer.

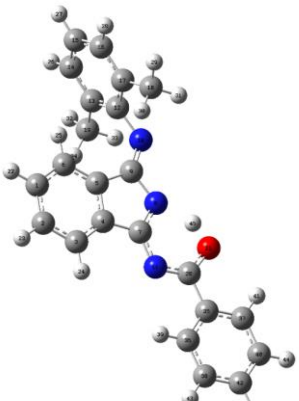

E-OH-isomer

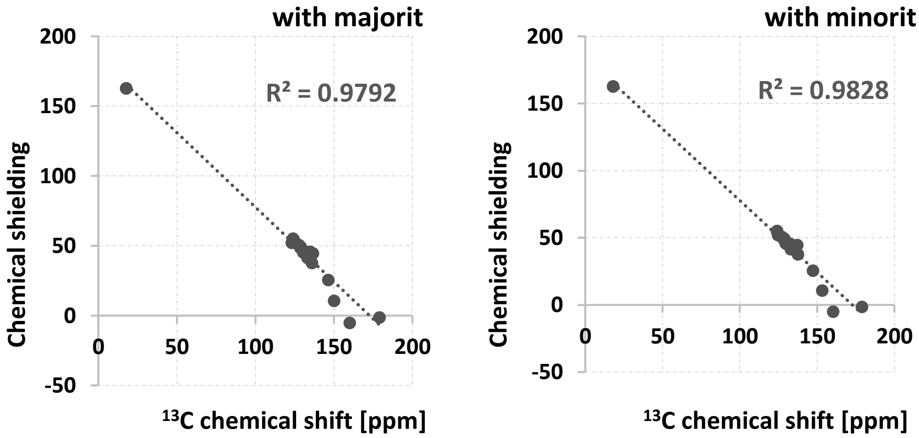

Figure S19. Correlation between experimental ^{13}C chemical shift of **6** (major data set) and (minor data set) with calculated shielding constants of *E*-OH-isomer.

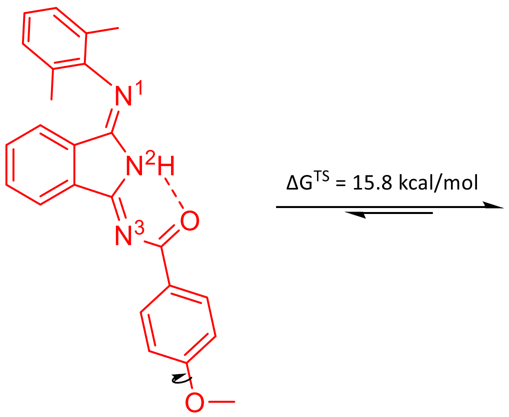

Table S6. Relative energy for isomers of compound **7** (*E* in red, *Z* in blue).

isomer	*Z*-N2-isomer (R)	*Z*-N2-isomer (L)	*E*-N2-isomer (L)	*E*-N2-isomer (R)	*Z*-N1-isomer	*Z*-N3-isomer
ΔG [kcal/mol]	0	0.29	1.52	1.70	10.24	11.96

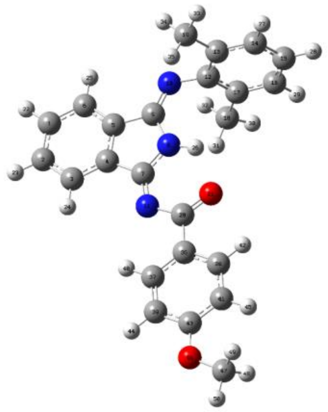

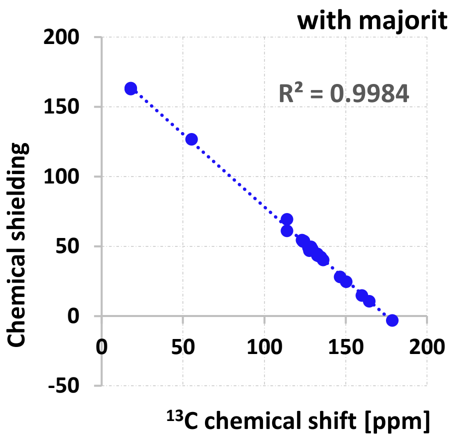

Z-N2-isomer

Figure S20. Correlation between experimental ^{13}C chemical shift of **7** (major data set) with calculated shielding constants of *Z*-N2-isomer.

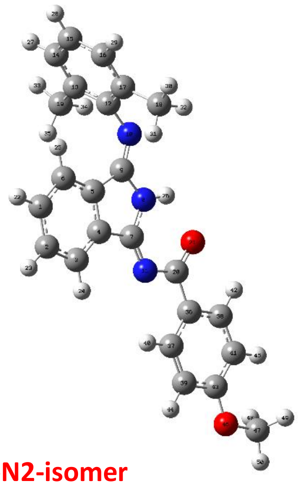

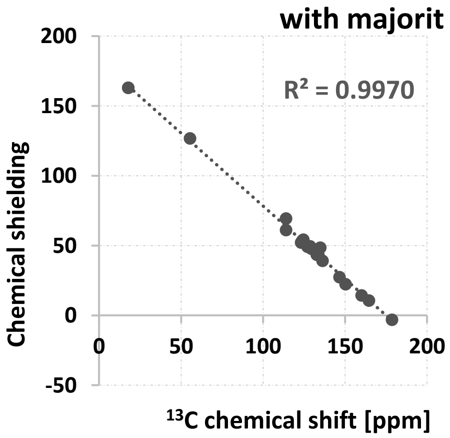

E-N2-isomer

Figure S21. Correlation between experimental ^{13}C chemical shift of **7** (major data set) with calculated shielding constants of *E*-N2-isomer.

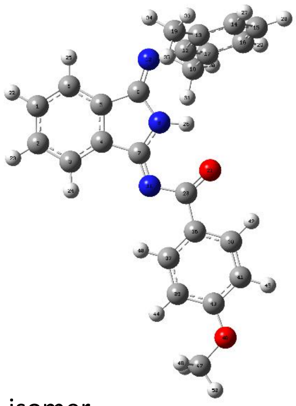

Z-N2-isomer

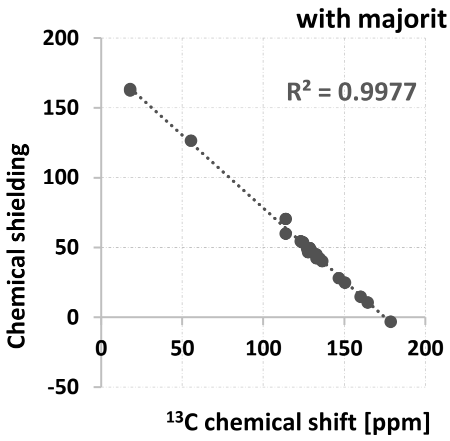

Figure S22. Correlation between experimental ^{13}C chemical shift of **7** (major data set) with calculated shielding constants of *Z*-N2-isomer.

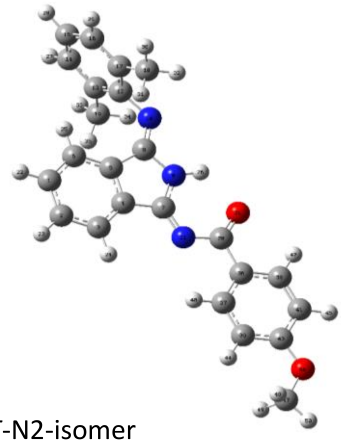

E-N2-isomer

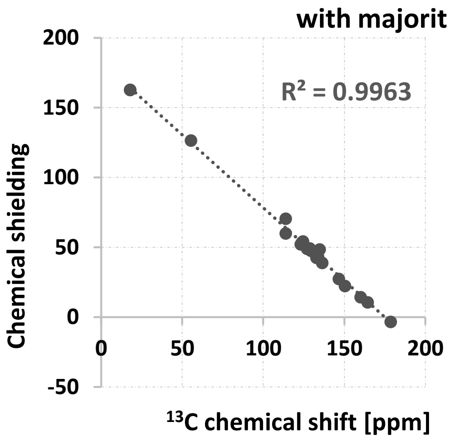

Figure S23. Correlation between experimental ^{13}C chemical shift of **7** (major data set) with calculated shielding constants of *E*-N2-isomer.

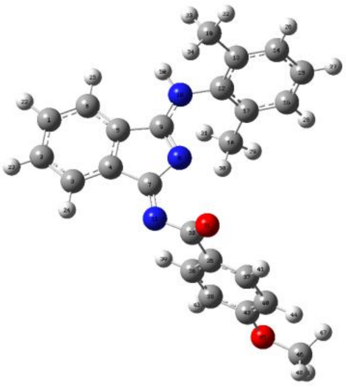

Z-N1-isomer

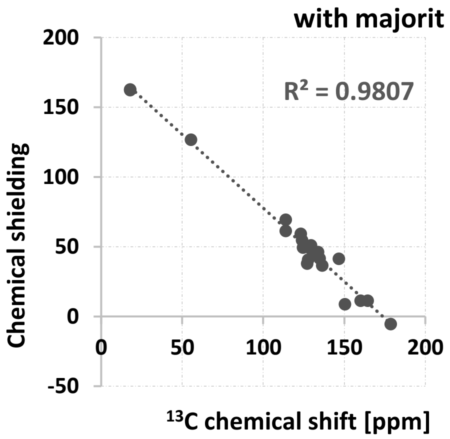

Figure S24. Correlation between experimental ^{13}C chemical shift of **7** (major data set) with calculated shielding constants of *Z*-N1-isomer.

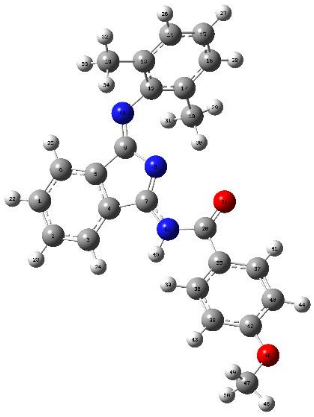

Z-N3-isomer

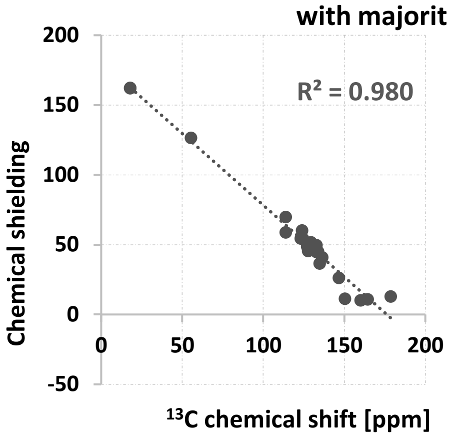

Figure S25. Correlation between experimental ^{13}C chemical shift of **7** (major data set) with calculated shielding constants of *Z*-N3-isomer.

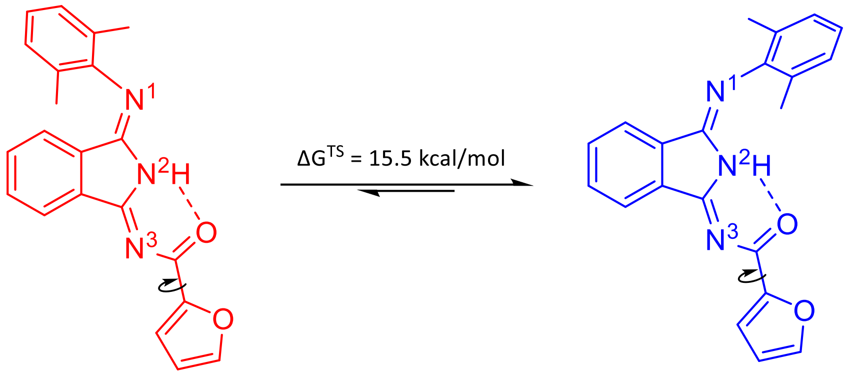

Table S7. Relative energy for isomers of compound **8** (*E* in red, *Z* in blue).

isomer	*Z*-N2-isomer (R)	*Z*-N2-isomer (L)	*E*-N2-isomer (R)	*E*-N2-isomer (L)	*E*-N1-isomer	*Z*-N3-isomer	*E*-N3-isomer
**ΔG [kcal/mol]**	0	0.62	1.62	1.93	9.72	11.60	14.80

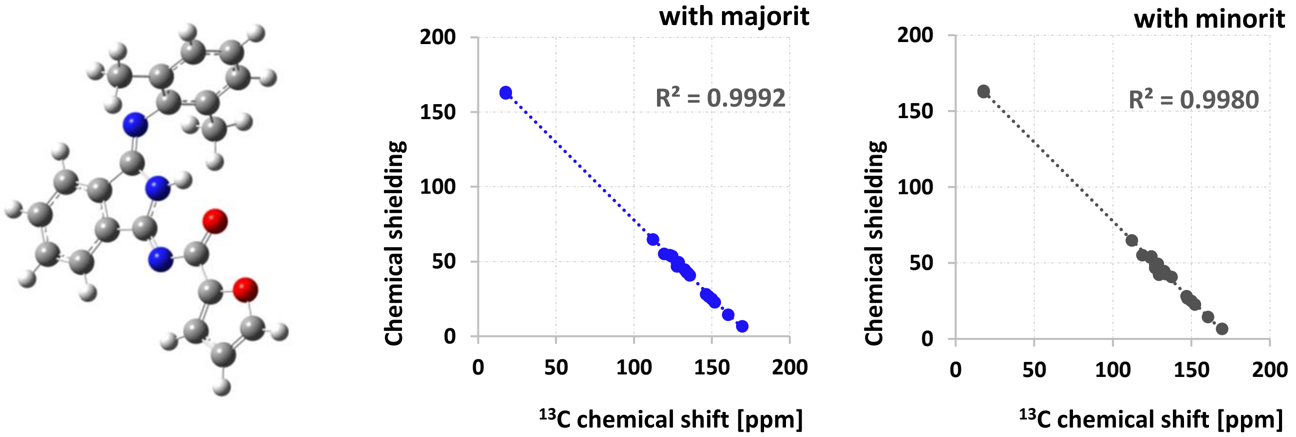

***Z*-N2-isomer**

Figure S26. Correlation between experimental ^{13}C chemical shift of **8** (major data set) and (minor data set) with calculated shielding constants of *Z*-N2-isomer.

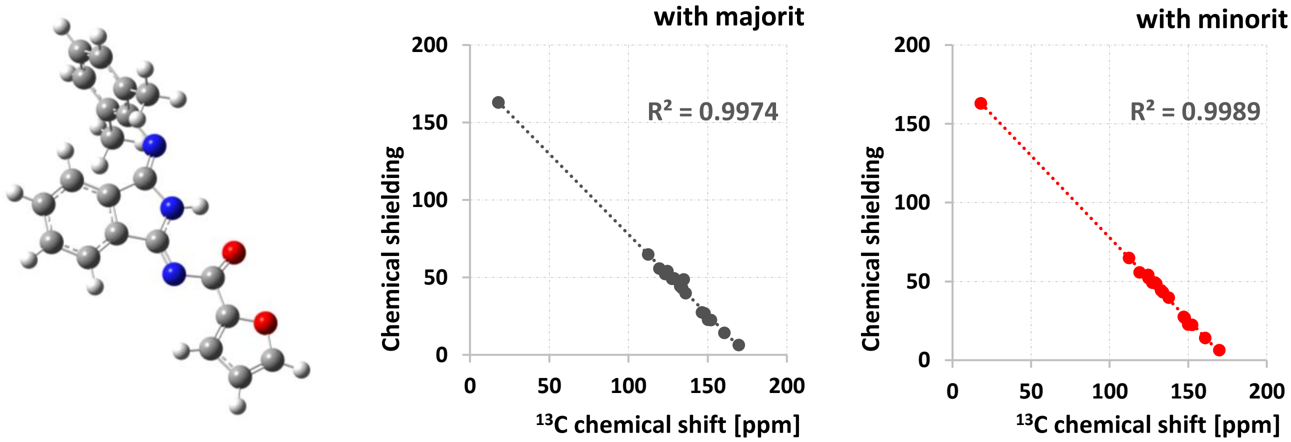

***E*-N2-isomer**

Figure S27. Correlation between experimental ^{13}C chemical shift of **8** (major data set) and (minor data set) with calculated shielding constants of *E*-N2-isomer.

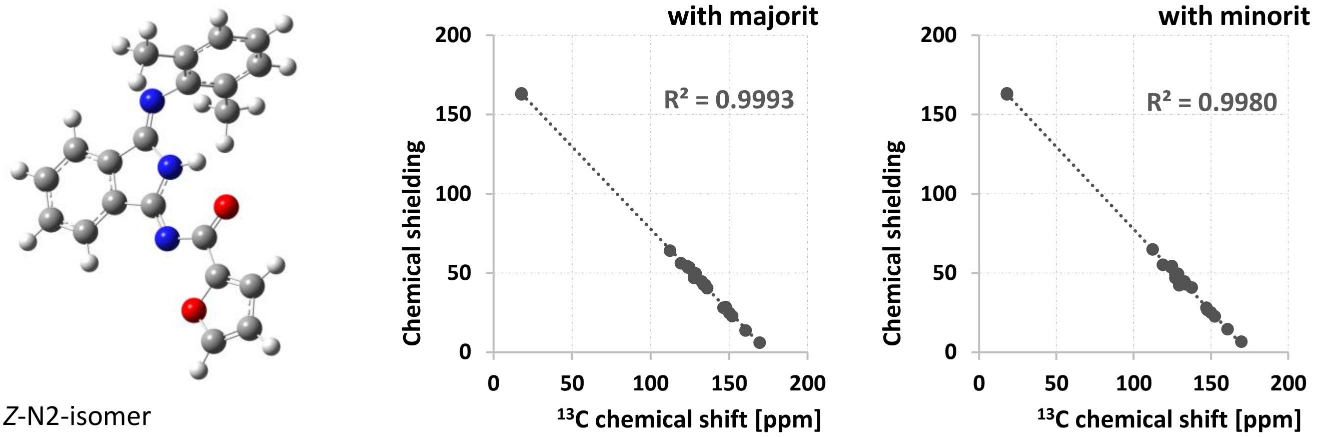

Figure S28. Correlation between experimental ^{13}C chemical shift of **8** (major data set) and (minor data set) with calculated shielding constants of *Z*-N2-isomer.

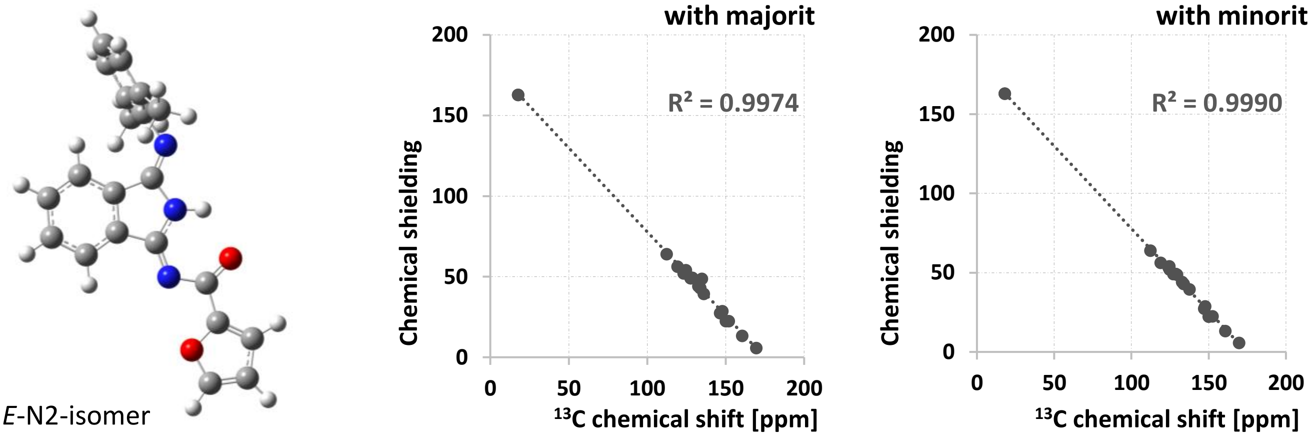

Figure S29. Correlation between experimental ^{13}C chemical shift of **8** (major data set) and (minor data set) with calculated shielding constants of *E*-N2-isomer.

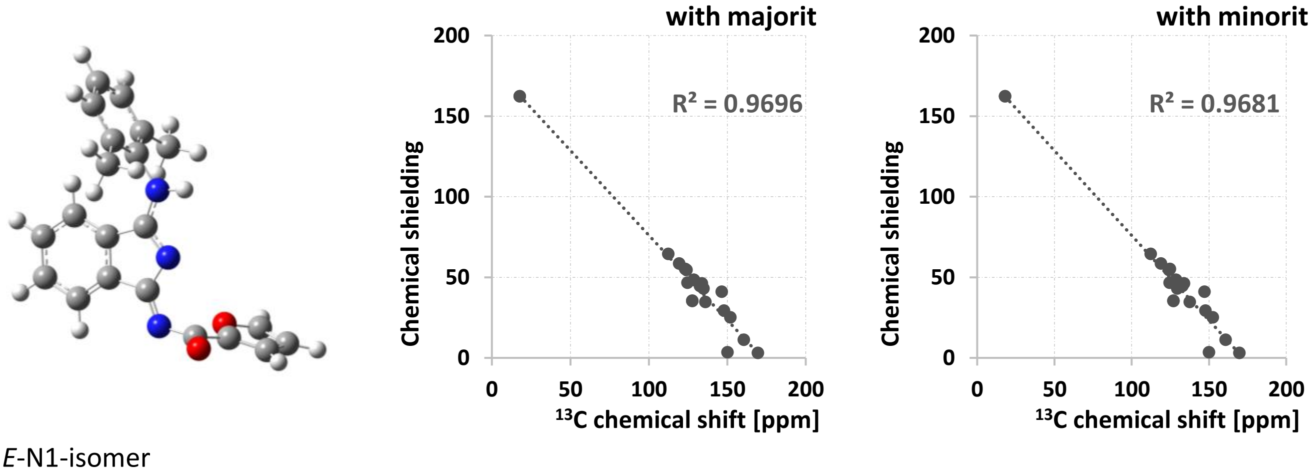

Figure S30. Correlation between experimental ^{13}C chemical shift of **8** (major data set) and (minor data set) with calculated shielding constants of *E*-N1-isomer.

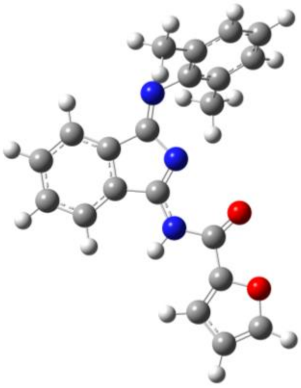

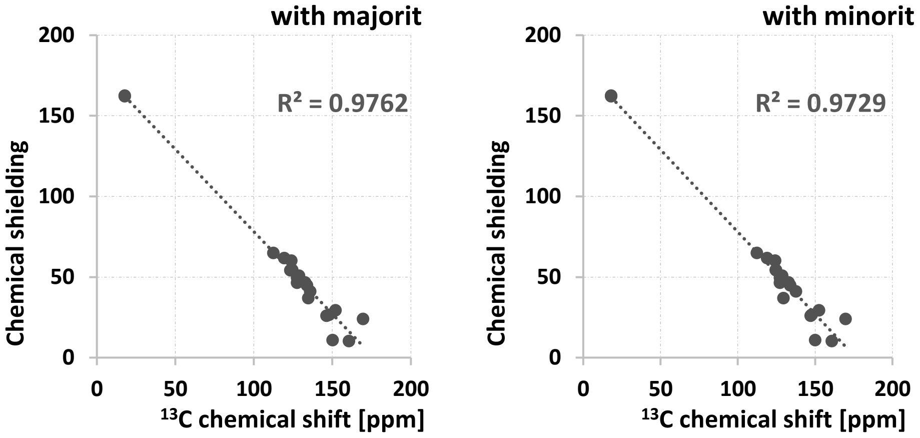

Z-N3-isomer

Figure S31. Correlation between experimental ^{13}C chemical shift of **8** (major data set) and (minor data set) with calculated shielding constants of *Z*-N3-isomer.

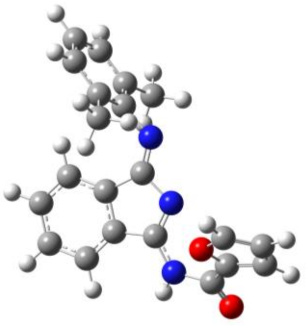

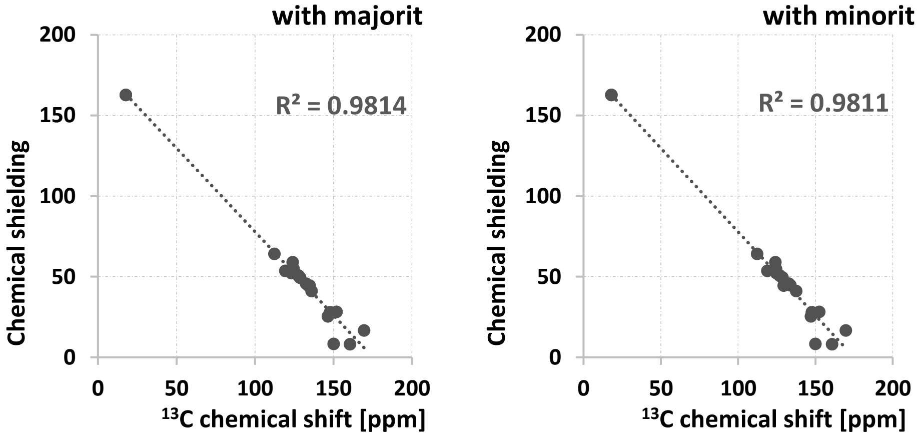

E-N3-isomer

Figure S32. Correlation between experimental ^{13}C chemical shift of **8** (major data set) and (minor data set) with calculated shielding constants of *E*-N3-isomer.

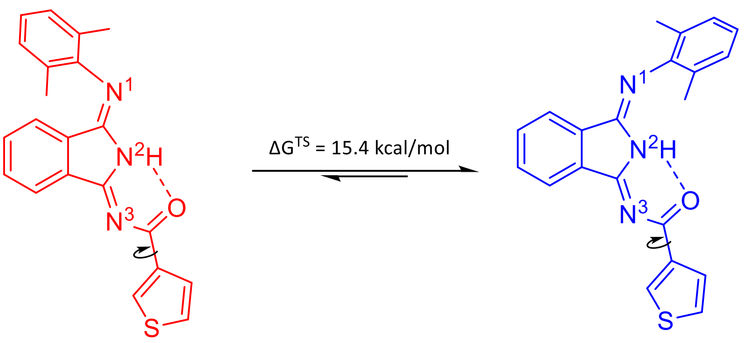

Table S8. Relative energy for isomers of compound **9** (*E* in red, *Z* in blue).

isomer	*Z*-N2-isomer (L)	*Z*-N2-isomer (R)	*E*-N2-isomer (L)	*E*-N2-isomer (R)	*Z*-N1-isomer	*Z*-N3-isomer
ΔG [kcal/mol]	0	0.43	1.40	2.01	9.62	11.30

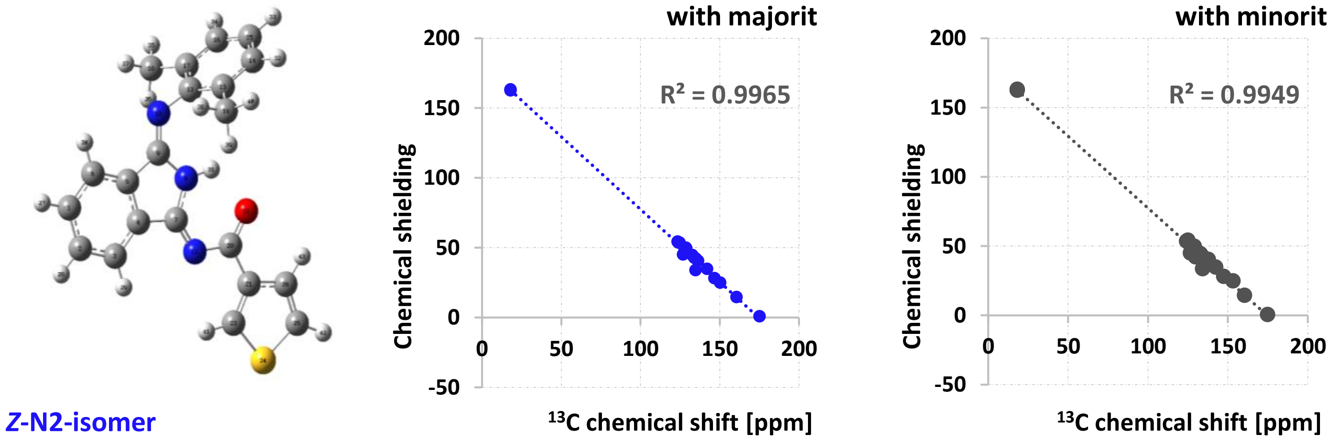

Figure S33. Correlation between experimental ^{13}C chemical shift of **9** (major data set) and (minor data set) with calculated shielding constants of *Z*-N2-isomer.

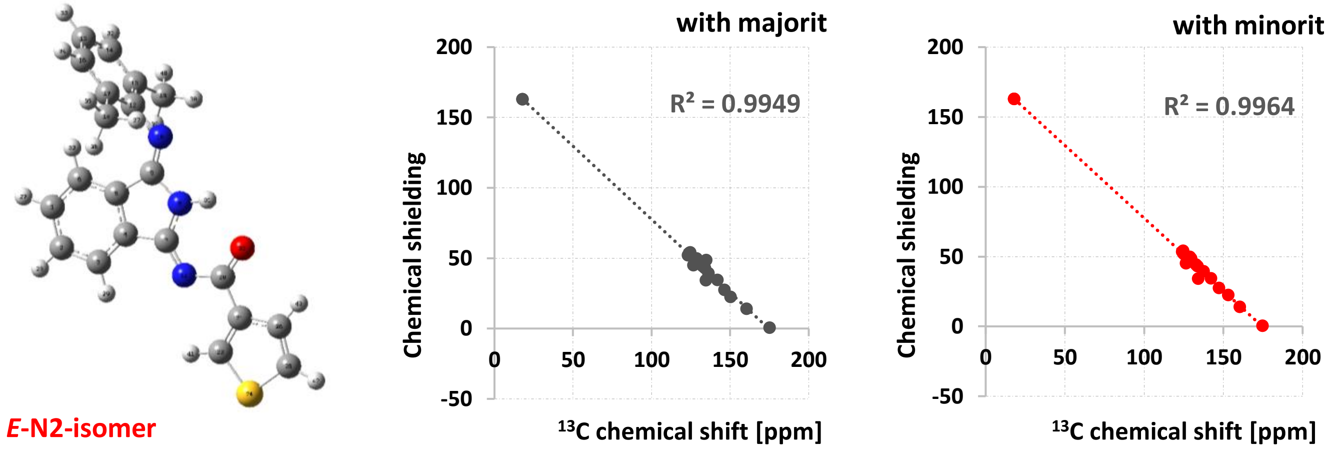

Figure S34. Correlation between experimental ^{13}C chemical shift of **9** (major data set) and (minor data set) with calculated shielding constants of *E*-N2-isomer.

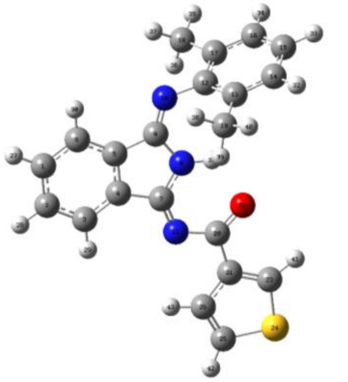

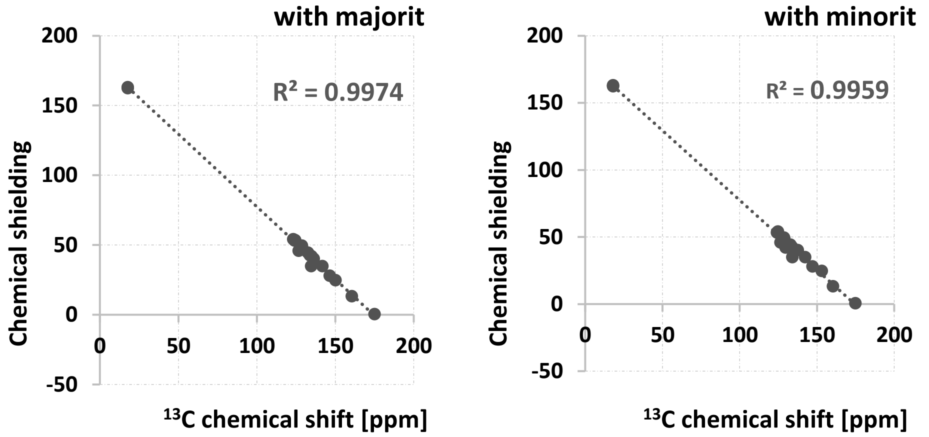

Z-N2-isomer

Figure S35. Correlation between experimental ^{13}C chemical shift of **9** (major data set) and (minor data set) with calculated shielding constants of *Z*-N2-isomer.

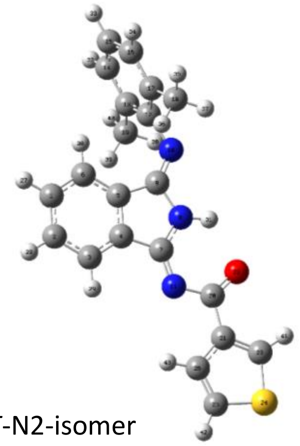

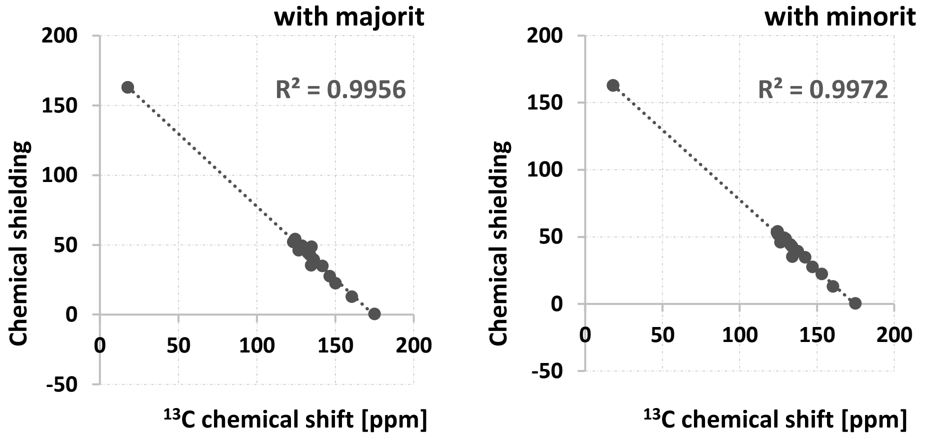

E-N2-isomer

Figure S36. Correlation between experimental ^{13}C chemical shift of **9** (major data set) and (minor data set) with calculated shielding constants of *E*-N2-isomer.

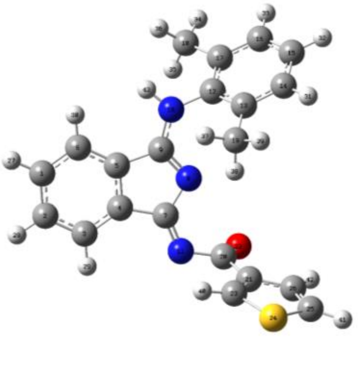

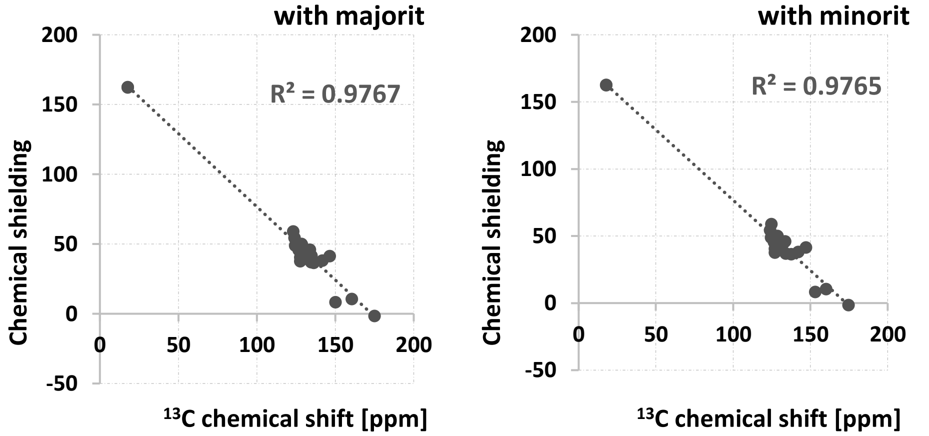

Z-N1-isomer

Figure S37. Correlation between experimental ^{13}C chemical shift of **9** (major data set) and (minor data set) with calculated shielding constants of *Z*-N1-isomer.

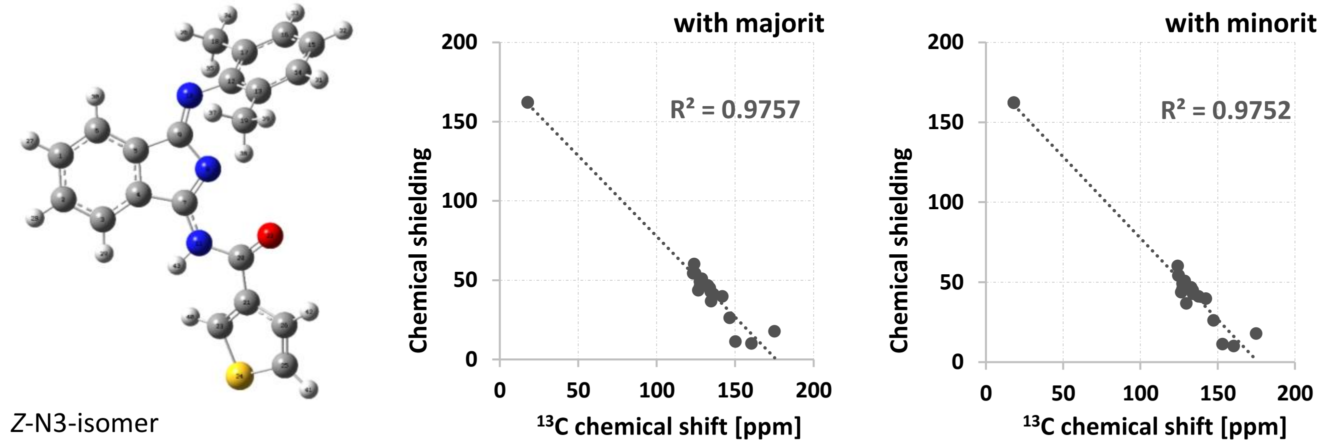

Figure S38. Correlation between experimental ^{13}C chemical shift of **9** (major data set) and (minor data set) with calculated shielding constants of *Z*-N3-isomer.

4 NMR spectroscopy

4.1 E/Z-isomerisation of complex Li6' promoted by water addition

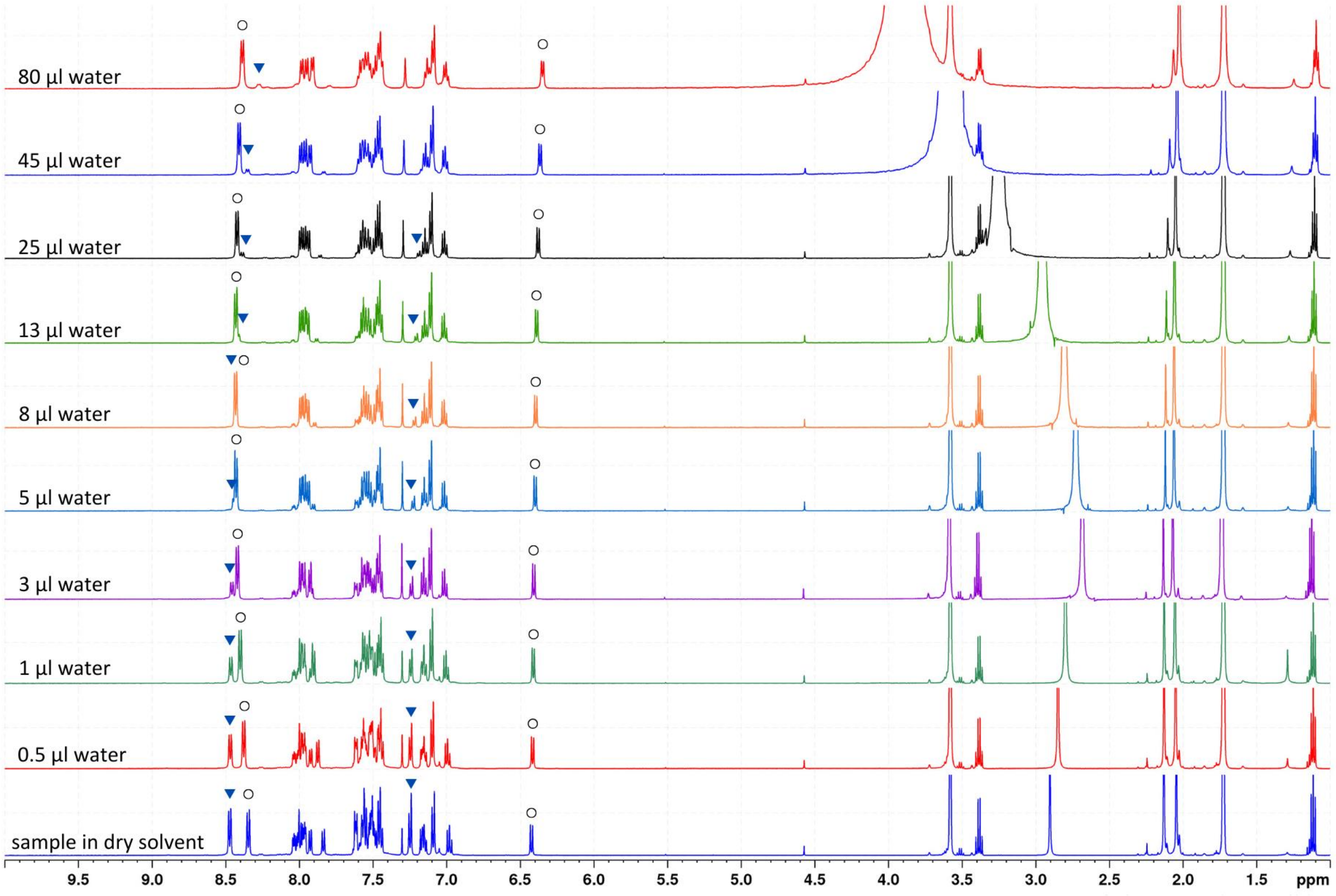

Figure S39. *E*/*Z*-isomerisation (*E* circle, *Z* triangle) of Li**6'** in THF-d_8 promoted by water addition - monitored by ^{1}H NMR spectroscopy at room temperature.

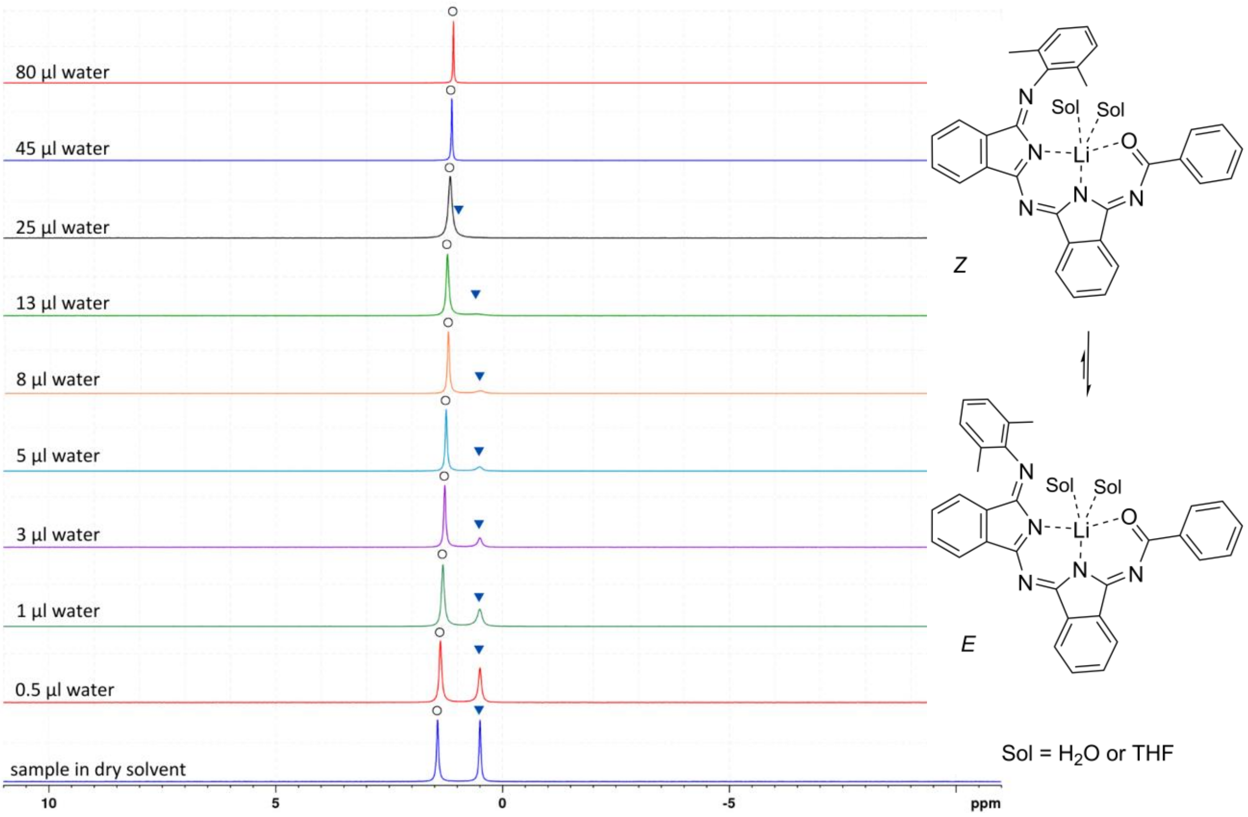

Figure S40. *E*/*Z*-isomerisation (*E* circle, *Z* triangle) of Li**6'** in THF-d_8 promoted by water addition - monitored by ^{7}Li NMR.

4.2 DOSY NMR spectroscopy

In order to describe higher structure (monomers or dimers) of the studied compounds in solution, we employed diffusion-ordered spectroscopy (DOSY) which can distinguish the chemical species using their diffusion coefficient.[15] As the DOSY spectrum is usually presented as 2D, where the diffusion coefficient is correlated with ^{1}H spectrum, it enables also the analysis of mixted samples. Usually, a standard analyte with known structure (or diffusion coefficient) is used as a reference.

Table S9. Diffusion coefficients obtained by DOSY NMR spectroscopy

	**Compound 1a**	**Compound 5ab**	**Compound *E-Li6'* THF**	**Compound *Z-*Li6' THF**	**Compound *E-Li6'* DCM**
Diffusion coefficient	$1.37 \cdot 10^{-9}$ m^2/s	$1.01 \cdot 10^{-9}$ m^2/s	$1.17 \cdot 10^{-9}$ m^2/s	$1.15 \cdot 10^{-9}$ m^2/s	$1.13 \cdot 10^{-9}$ m^2/s

Compound **5ab** was used as a reference molecule with a 611.88 g/mol molecular weight and monomeric structure. Significant differences in diffusion coefficients of compounds **1a** and **Li6'** (Table S9) compared to the reference molecule **5ab** indicate the lower molecular weight and, thus, monomeric structure of compounds **1a** and Li**6'** in solution.

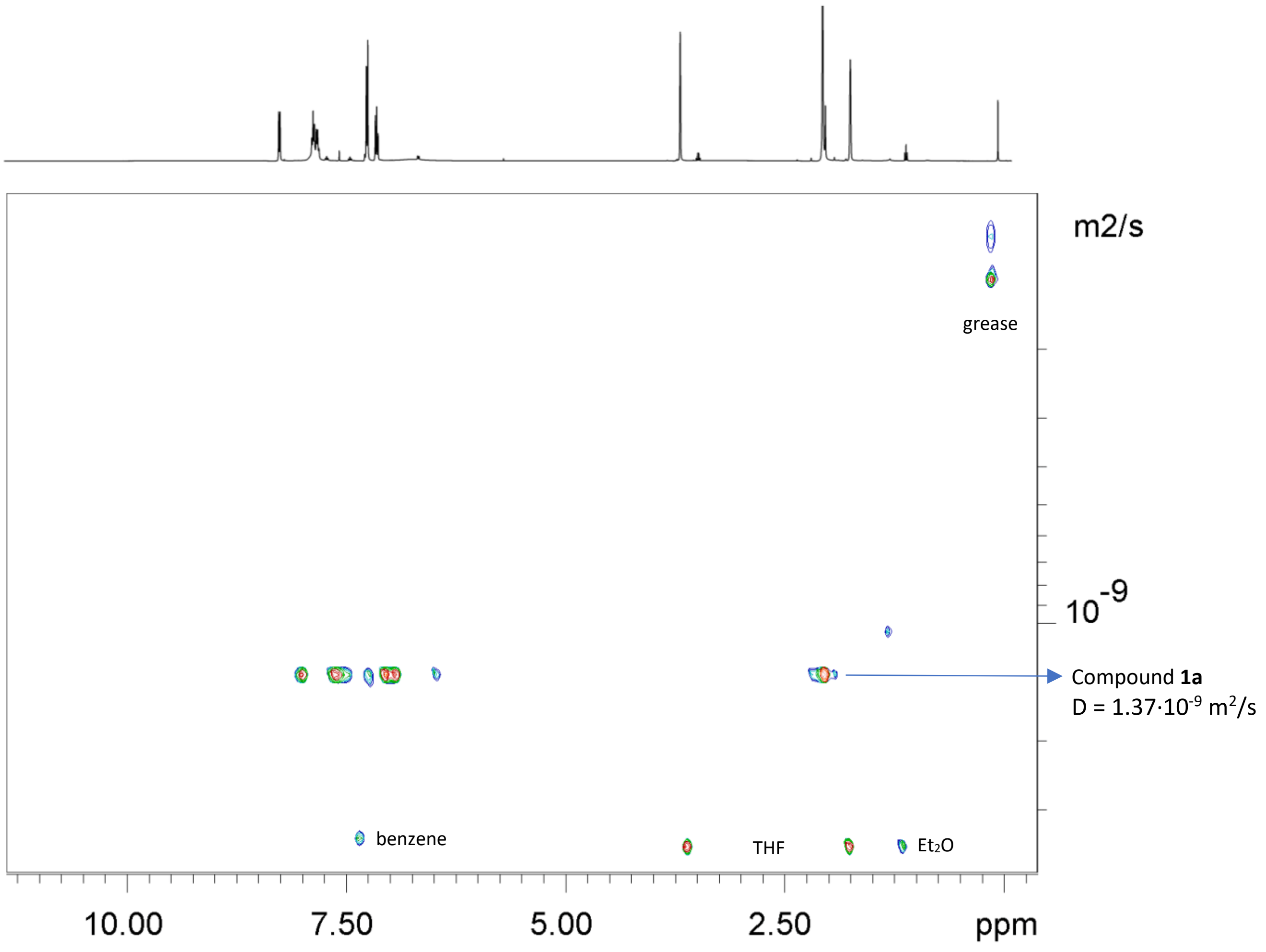

Figure S41. DOSY NMR spectrum for compound **1a** in THF-d_8 at room temperature.

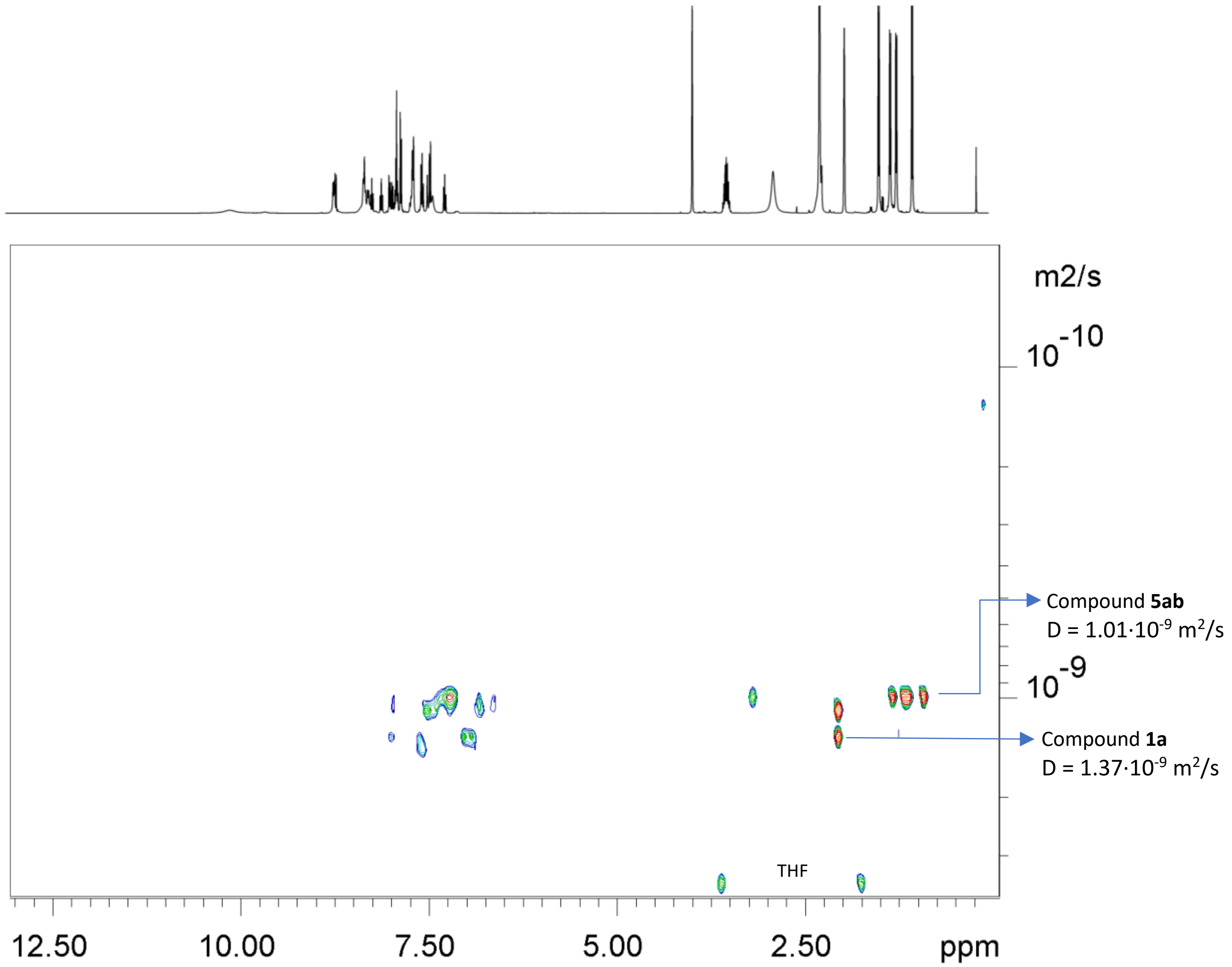

Figure S42. DOSY NMR spectrum of a mixture of compounds **1a** and **5ab** which is not able to make a dimer, in THF-d_8 at room temperature.

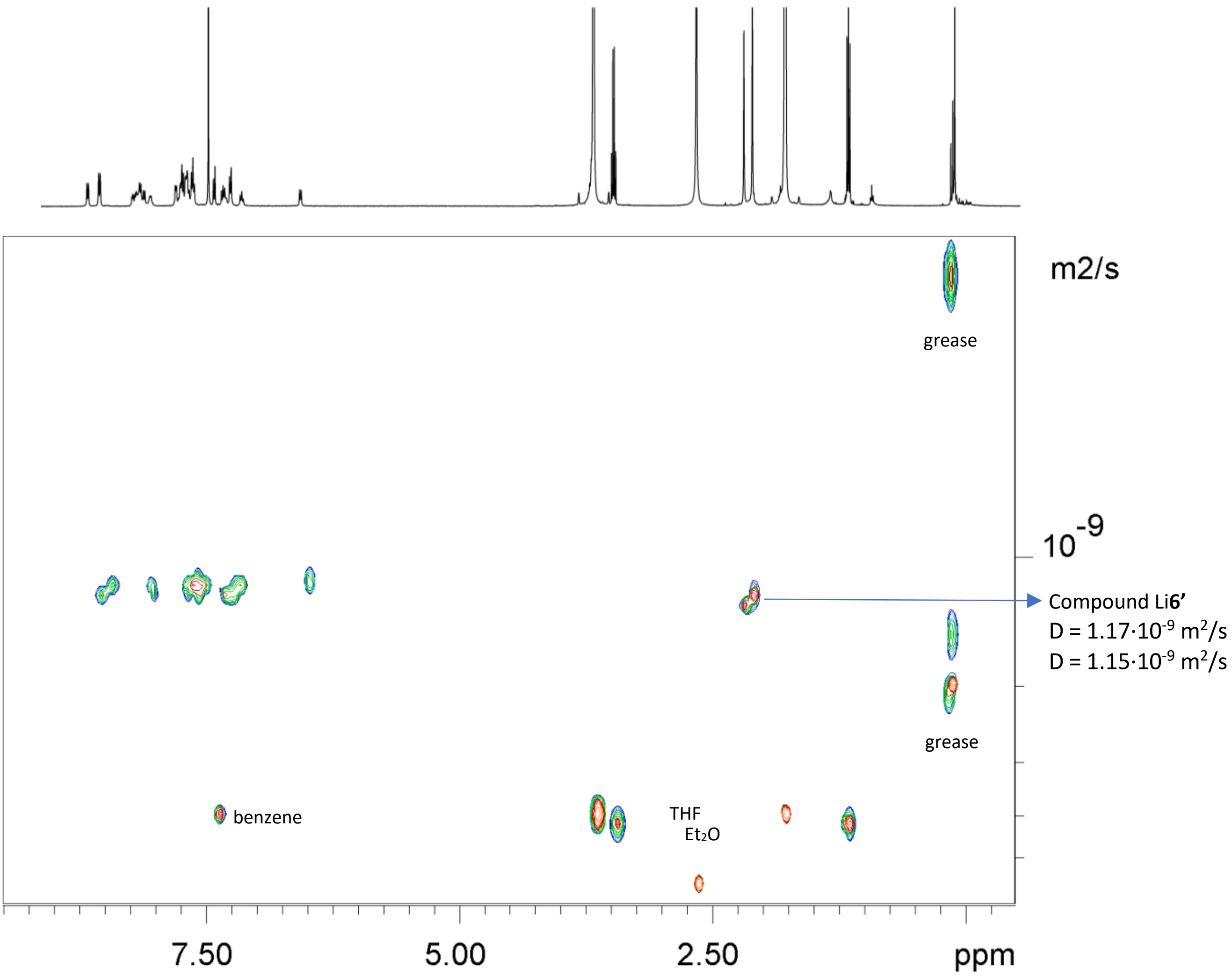

Figure S43. DOSY NMR spectrum for compound Li**6'** in THF-d_8 (*E*/*Z*-isomers in ratio 1:1) at room temperature, indicating monomeric structure of such compound.

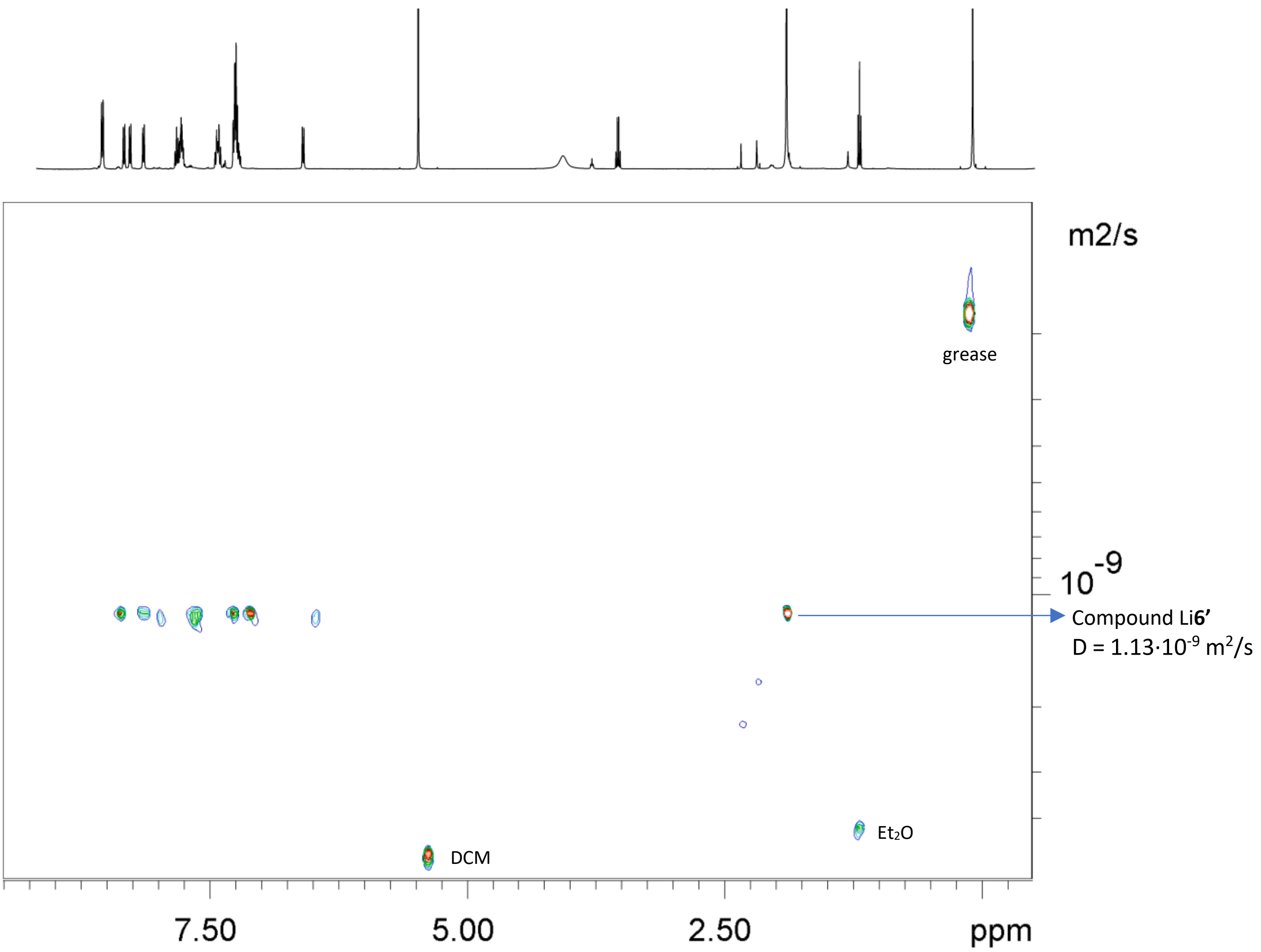

Figure S44. DOSY NMR spectrum for compound Li**6'** in CD_2Cl_2 (only *E*-isomer is present) at room temperature, indicating monomeric structure of such compound.

4.3 ^{1}H,^{15}N-HSQC NMR spectroscopy

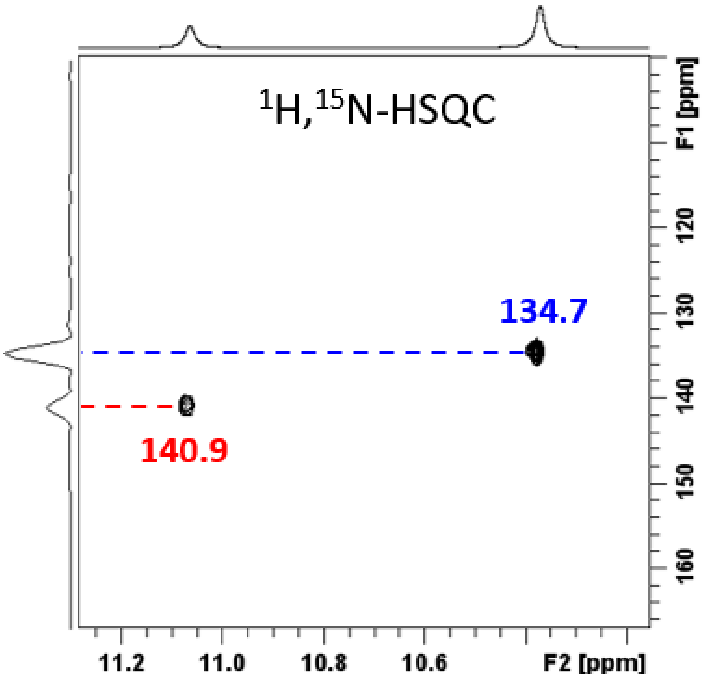

Figure S45. ^{1}H,^{15}N-HSQC NMR spectrum for compound **6.** Chemical shift of nitrogen atom N2 differing of more than 6 ppm for the two forms indicates sterically hindered rotation at C1-N1 bond leading to *E*/*Z* isomers.

5 Solid state structure determination

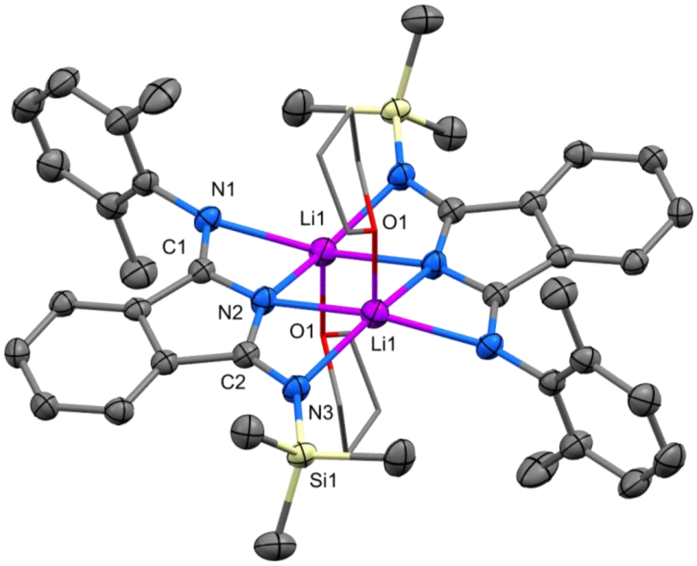

Figure S46. The ORTEP view 50% probability level of molecular structure of Li**1a**. Hydrogen atoms are omitted for clarity. Selected bond lengths (Å) and angles (°): N1–C9 1.418(3), N1–C1 1.289(3), C1–N2 1.372(3), N2–C2 1.363(3), C2–N3 1.296(3), N3–Si1 1.713(2), N1–Li1 2.209(4), N2–Li1 2.127(4), N3–Li1 2.100(4), C1–N1–C9 120.79(18), N1–C1–C4 132.14(19), N1–Li1–N2 63.7(1), N2–Li1–N3 64.1(1).

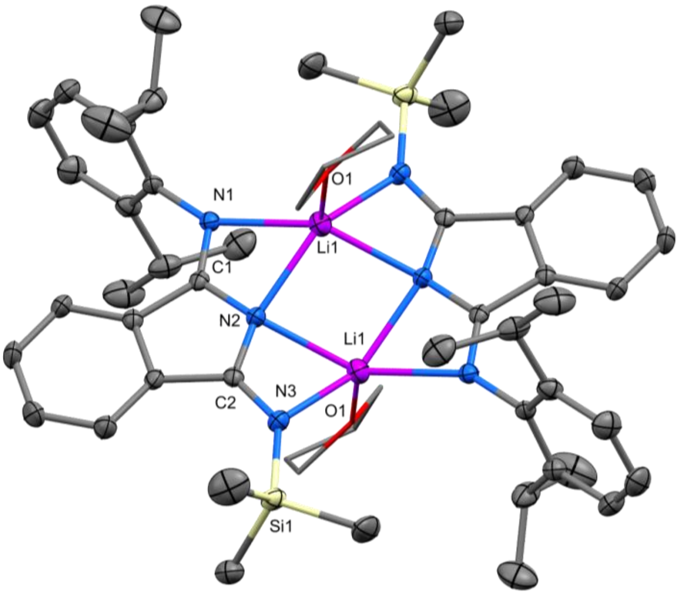

Figure S47. The ORTEP view 50% probability level of molecular structure of Li**1b**. Hydrogen atoms and THF molecule are omitted for clarity. Selected bond lengths (Å) and angles (°): N1–C9 1.420(3), N1–C1 1.294(3), C1–N2 1.371(3), N2–C2 1.368(3), C2–N3 1.297(3), N3–Si1 1.725(2), N1–Li1 2.177(5), N2–Li1 2.193(5), N3–Li1 2.143(5), C1–N1–C9 120.4(2), N1–C1–C4 131.9(2), N1–Li1–N2 63.20(14), N2–Li1–N3 64.53(15).

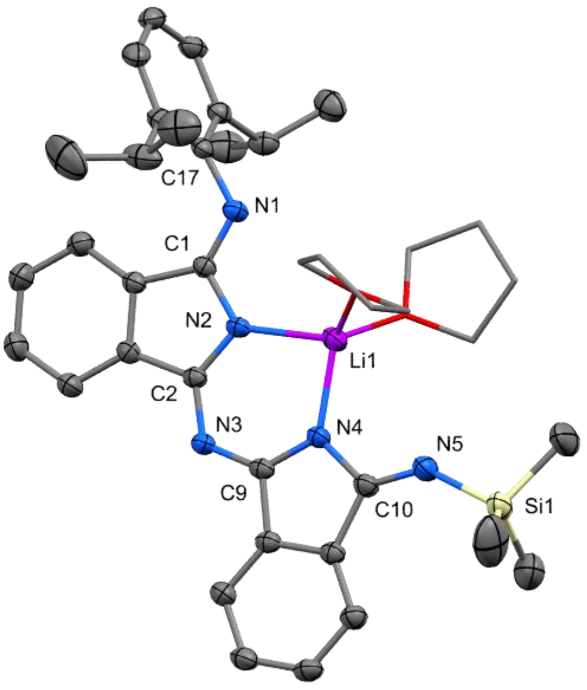

Figure S48. The ORTEP view 50% probability level of molecular structure of Li**3b**. Hydrogen atoms are omitted for clarity. Selected bond lengths (Å) and angles (°): N1–C17 1.427(4), N1–C1 1.273(3), C1–N2 1.414(4), N2–C2 1.332(3), C2–N3 1.343(3), N3–C9 1.349(4), N4–C9 1.330(3), N4–C10 1.422(4), N5–C10 1.270(3), N5–Si1 1.732(3), N2–Li1 2.019(5), N4–Li1 1.985(5), C1–N1–C17 121.8(2), N1–C1–C4 131.1(2), N2–Li1–N4 90.0(2), C10–N5–Si1 138.7(2).

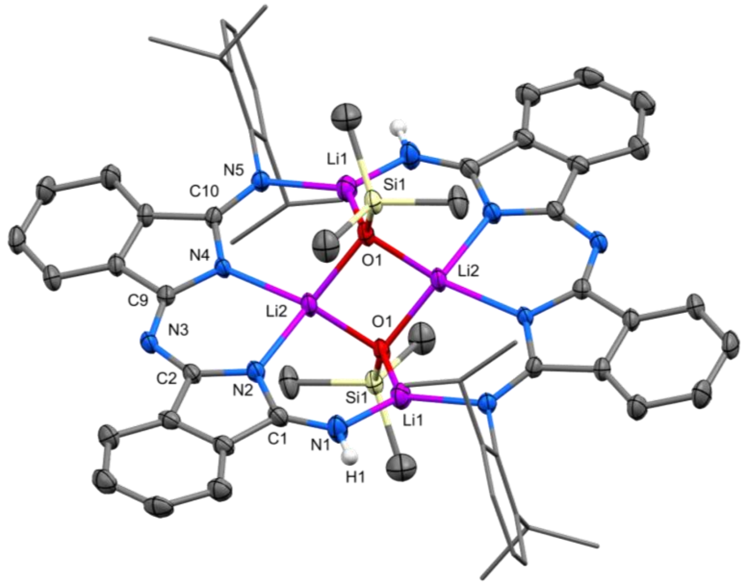

Figure S49. The ORTEP view 50% probability level of molecular structure of Li**3b'**. Hydrogen atoms are omitted for clarity. Selected bond lengths (Å) and angles (°): N1–C1 1.277(4), C1–N2 1.387(4), N2–C2 1.338(4), C2–N3 1.328(3), N3–C9 1.331(4), N4–C9 1.334(4), N4–C10 1.400(3), N5–C10 1.278(4), N5–C17 1.435(3), N1–Li1 1.985(7), N2–Li2 2.048(6), N4–Li2 2.090(4), N5–Li1 2.009(6), O1–Li1 1.837(6), O1–Li2 1.971(6), O1–Si1 1.601(2), C10–N5–C17 119.0(2), C9–N3–C2 121.7(2), N2–Li2–N4 86.9(2), Li1–O1–Li2 92.8(2).

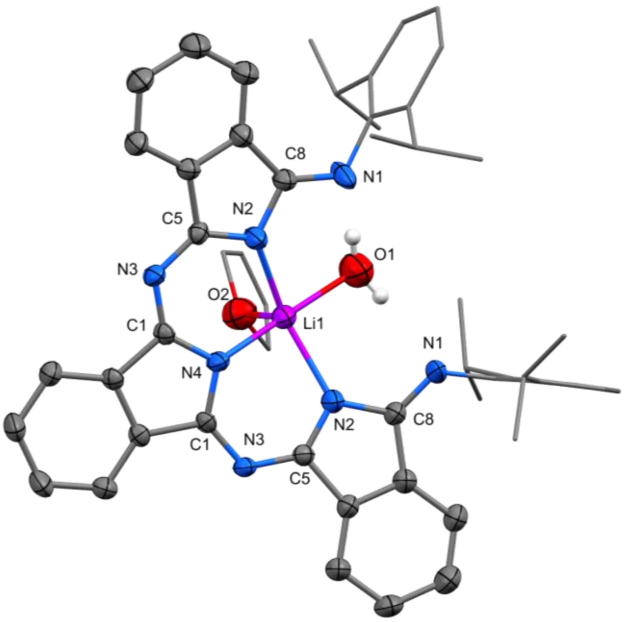

Figure S50. The ORTEP view 50% probability level of molecular structure of Li**3b''**. Hydrogen atoms and molecule of THF are omitted for clarity. Selected bond lengths (Å) and angles (°): N1–C8 1.280(19), C8–N2 1.41(4), N2–C5 1.33(3), C5–N3 1.334(16), N3–C1 1.320(4), N4–C1 1.356(4), N4–Li1 2.007(13), N2–Li1 2.11(4), Li1–O1 2.10(2), Li1–O2 2.04(2), C8–N1–C14 120.4(14), C1–N3–C5 121.3(7), N2–Li1–N4 85.5(10), N4-Li1-O1 176.3(12), N4-Li1-O2 97.6(8).

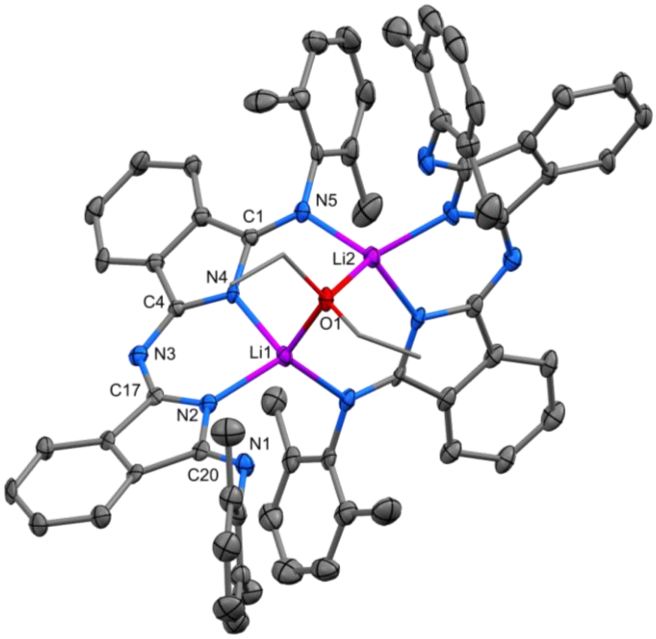

Figure S51. The ORTEP view 50% probability level of molecular structure of Li**2a**. Hydrogen atoms, hexane and diethyl ether molecule are omitted for clarity. Selected bond lengths (Å) and angles (°): N1–C20 1.258(7), N2–C20 1.405(6), N2–C17 1.313(7), C17–N3 1.347(6), N3–C4 1.347(6), N4–C4 1.331(6), N4–C1 1.398(6), N5–C1 1.292(6), N5–Li2 2.119(10), N4–Li1 2.010(9), N2–Li1 1.993(8), Li1-O1 2.094(9), Li2-O1 2.052(8), C1–N5–C9 117.5(4), C20–N1–C25 120.3(4), N2–Li1–N4 90.0(4), N4–Li1–O1 100.8(4).

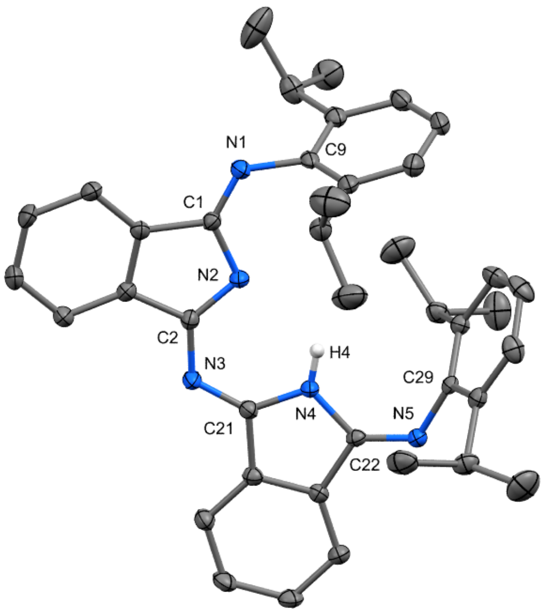

Figure S52. The ORTEP view 50% probability level of molecular structure of **2b**. Hydrogen atoms and hexane molecule are omitted for clarity. Selected bond lengths (Å) and angles (°): N1–C9 1.4240(17), N1–C1 1.2699(17), C1–N2 1.4269(16), N2–C2 1.3314(17), C2–N3 1.3534(17), N3–C21 1.3156(17), N4–C21 1.3574(17), N4–C22 1.4158(16), N5–C22 1.2664(17), N5–C29 1.4224(17), C1–N1–C9 124.77(12), N1–C1–C4 122.93(12), C2–N3–C21 120.57(12), C22–N5–C29 122.01(11).

D—H···*A*	*D*—H	H···*A*	*D*···*A*	*D*—H···*A*
N4—H4···N2	0.84(2)	2.20(2)	2.761(2)	124(2)

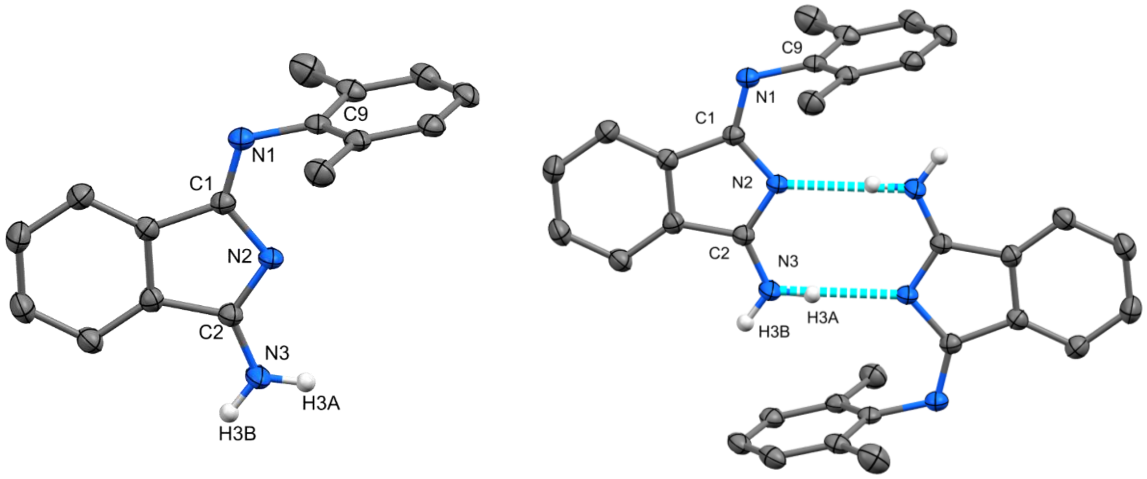

Figure S53. The ORTEP view 50% probability level of molecular structure of **1a**. Hydrogen atoms and water molecule are omitted for clarity. Selected bond lengths (Å) and angles (°): N1–C9 1.4268(16), N1–C1 1.2826(16), C1–N2 1.3917(15), N2–C2 1.3270(16), C2–N3 1.3097(16), C1–N1–C9 116.17(10), N1–C1–C4 124.74(11).

Hydrogen-bond geometry (Å, °)

D—H···*A*	*D*—H	H···*A*	*D*···*A*	*D*—H···*A*
N3—H3A···N2	0.88	1.969(2)	2.846(2)	174.57

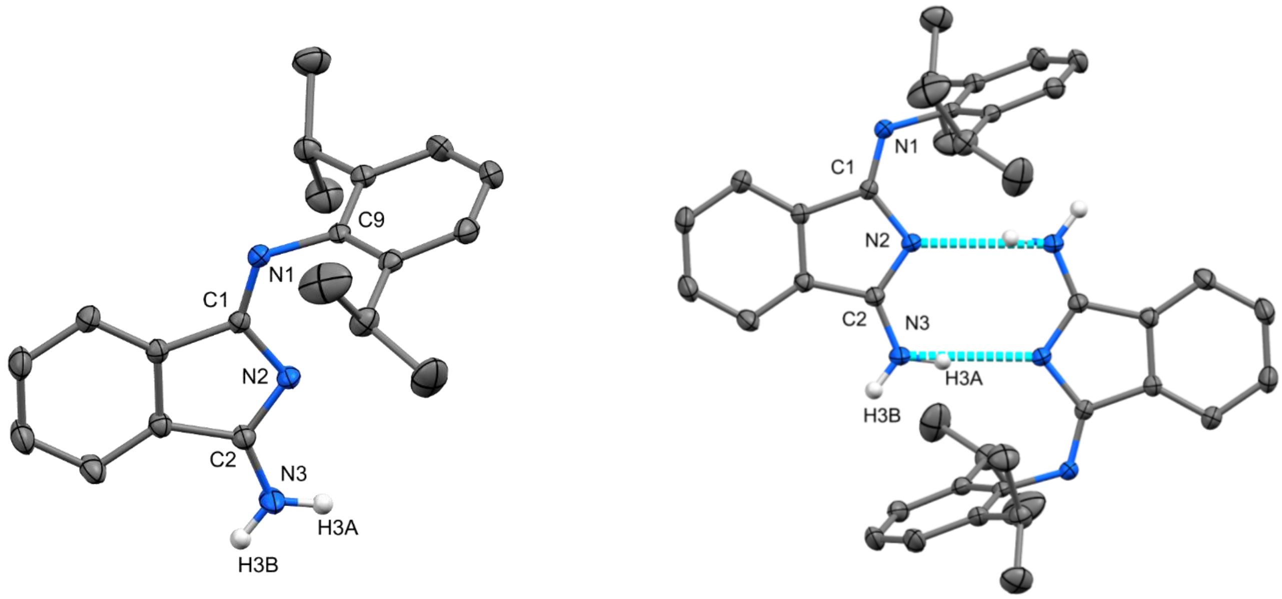

Figure S54. The ORTEP view 50% probability level of molecular structure of **1b**. Hydrogen atoms are omitted for clarity. Selected bond lengths (Å) and angles (°): N1–C9 1.4241(13), N1–C1 1.2790(13), C1–N2 1.4052(13), N2–C2 1.3309(13), C2–N3 1.3253(14), C1–N1–C9 119.70(9), N1–C1–C4 123.60(9).

Hydrogen-bond geometry (Å, °)

D—H···*A*	*D*—H	H···*A*	*D*···*A*	*D*—H···*A*
N3—H3A···N2	0.88	2.033(1)	2.905(1)	170.73

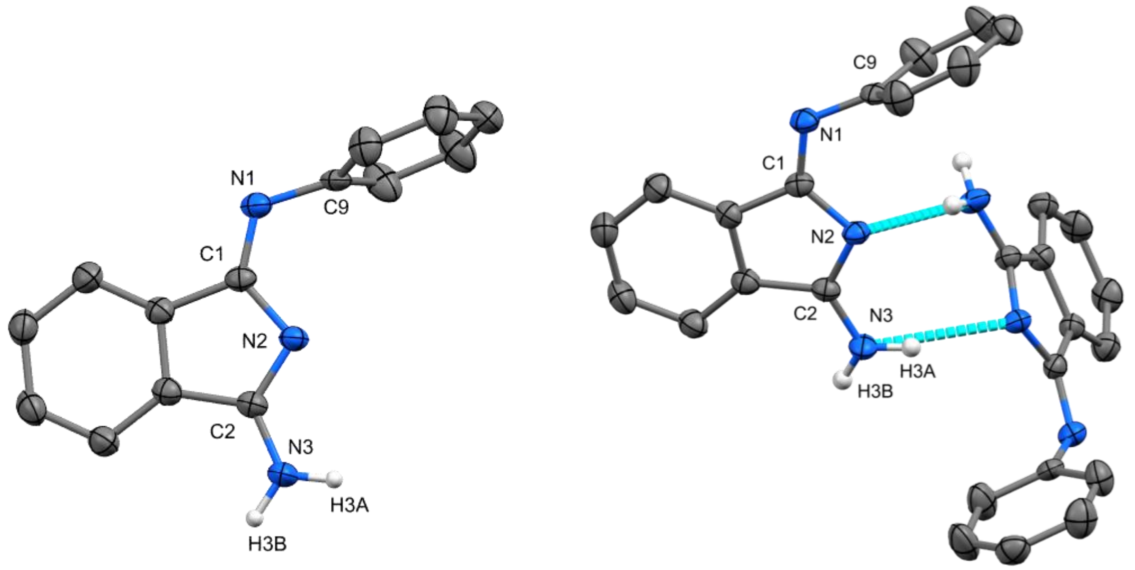

Figure S55. The ORTEP view 50% probability level of molecular structure of **1c**. Hydrogen atoms and water molecule are omitted for clarity. Selected bond lengths (Å) and angles (°): N1–C9 1.423(3), N1–C1 1.287(3), C1–N2 1.387(3), N2–C2 1.331(3), C2–N3 1.317(3), C1–N1–C9 119.3(2), N1–C1–C4 122.7(2).

Hydrogen-bond geometry (Å, °)

D—H···*A*	*D*—H	H···*A*	*D*···*A*	*D*—H···*A*
N3—H3A···N2	0.88(4)	2.16(3)	2.948(3)	149(3)
N3—H3B···N1	0.90(4)	2.03(4)	2.916(3)	168(3)

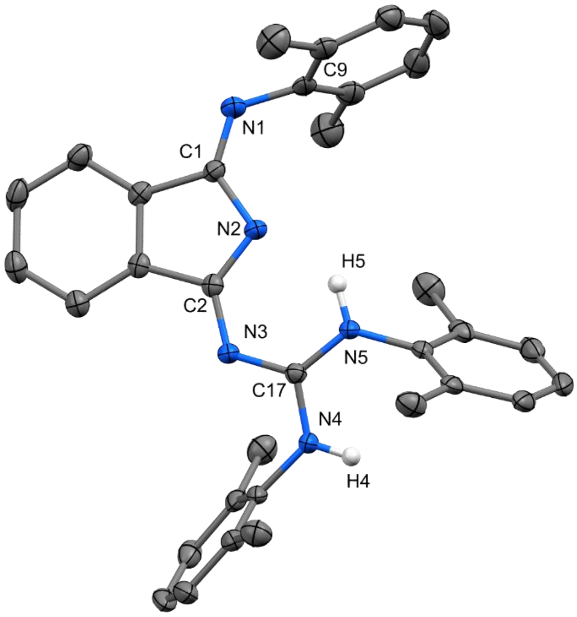

Figure S56. The ORTEP view 50% probability level of molecular structure of **5aa**. Hydrogen atoms are omitted for clarity. Selected bond lengths (Å) and angles (°): N1–C9 1.416(2), N1–C1 1.274(2), C1–N2 1.4078(19), N2–C2 1.339(2), C2–N3 1.3398(19), N3–C17 1.342(2), N4–C17 1.341(2), N5–C17 1.339(2), C1–N1–C9 122.27(14), N1–C1–C4 122.05(14), C2–N3–C17 120.23(13), N4–C17–N5 118.68(14).

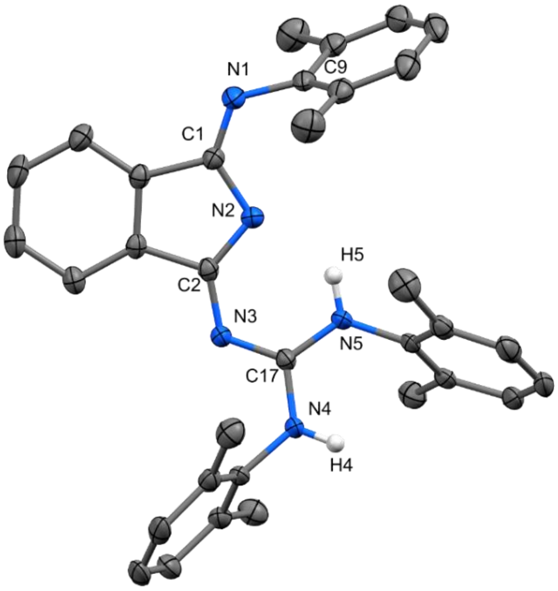

Figure S57. The ORTEP view 50% probability level of molecular structure of **5aa’**. Hydrogen atoms are omitted for clarity. Selected bond lengths (Å) and angles (°): N1–C9 1.420(3), N1–C1 1.270(2), C1–N2 1.409(2), N2–C2 1.343(2), C2–N3 1.336(2), N3–C17 1.343(2), N4–C17 1.344(2), N5–C17 1.345(2), C1–N1–C9 122.15(16), N1–C1–C4 121.97(17), C2–N3–C17 120.20(16), N4–C17–N5 118.32(16).

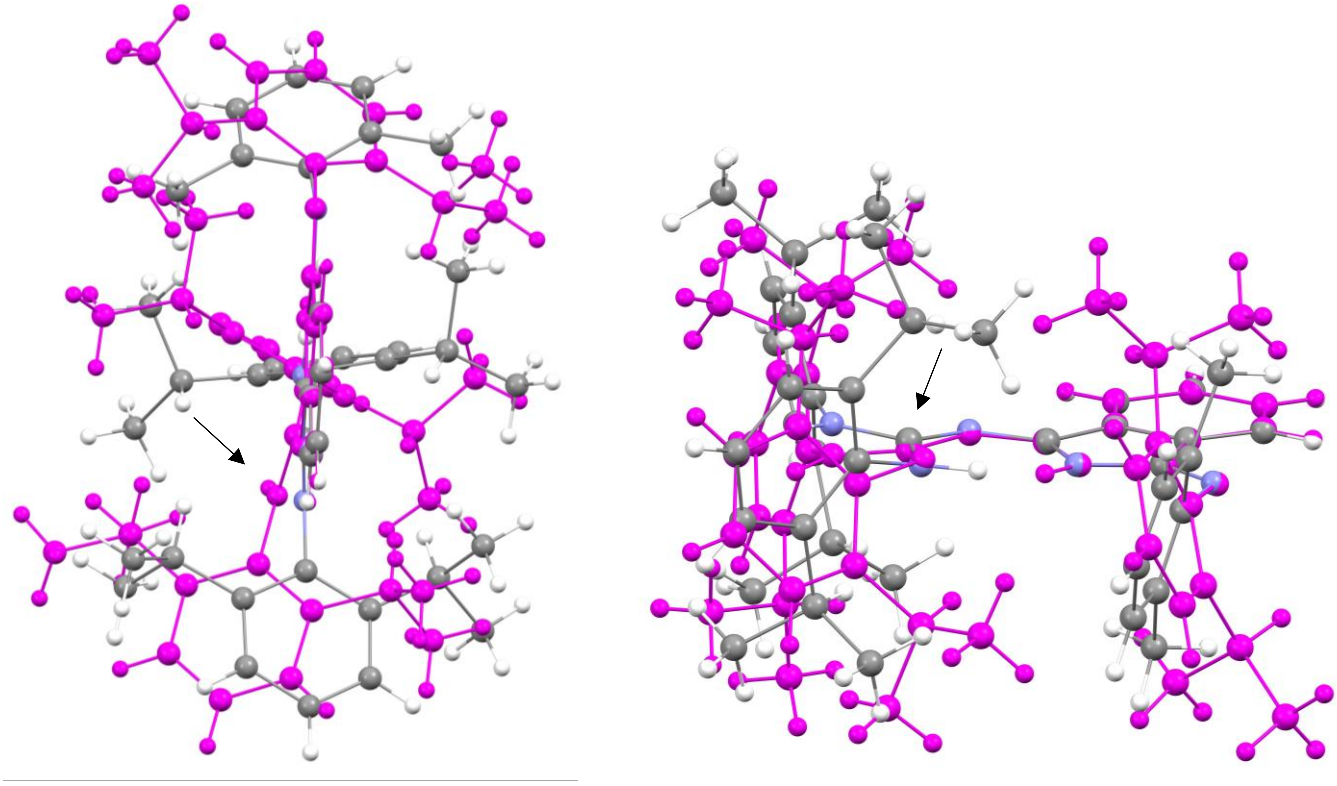

Figure S58. Structure overlay views of **5ab** (colours by elements – 4,5-tautomer) and **5bb** (magenta – 2,4-tautomer) with associated DII units, showing deviations of “guanidine“ part of **5bb** from planar arrangement.

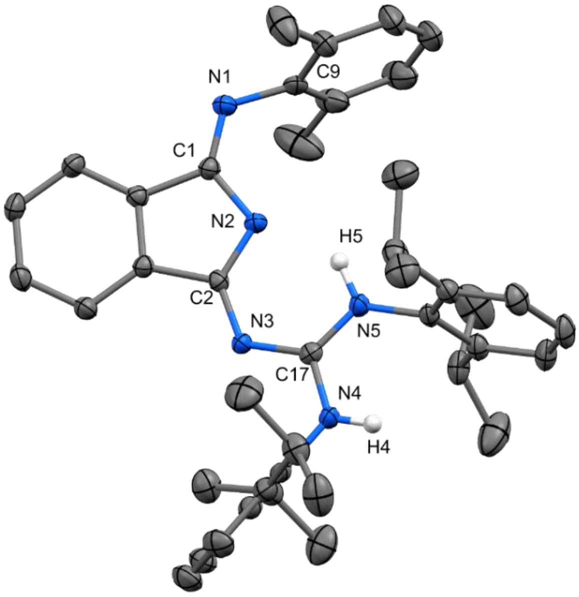

Figure S59. The ORTEP view 50% probability level of molecular structure of **5ab**. Hydrogen atoms are omitted for clarity. Selected bond lengths (Å) and angles (°): N1–C9 1.4216(17), N1–C1 1.2753(16), C1–N2 1.4055(15), N2–C2 1.3467(15), C2–N3 1.3249(15), N3–C17 1.3573(15), N4–C17 1.3556(15), N5–C17 1.3278(16), C1–N1–C9 119.95(11), N1–C1–C4 124.69(11), C2–N3–C17 119.42(10), N4–C17–N5 119.96(11).

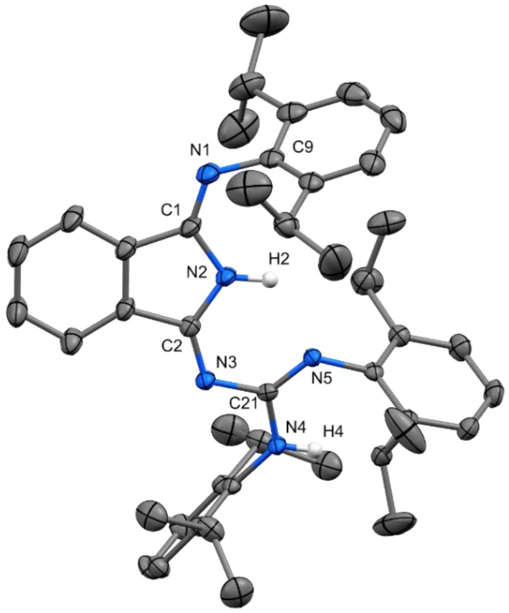

Figure S60. The ORTEP view 50% probability level of molecular structure of **5bb**. Hydrogen atoms and toluene molecule are omitted for clarity. Selected bond lengths (Å) and angles (°): N1–C9 1.419(2), N1–C1 1.271(2), C1–N2 1.4085(18), N2–C2 1.361(2), C2–N3 1.3013(18), N3–C21 1.3573(15), N4–C21 1.3880(18), N5–C21 1.3005(19), C1–N1–C9 121.07(13), N1–C1–C4 125.30(13), C2–N3–C21 118.88(13), N4–C21–N5 122.16(12).

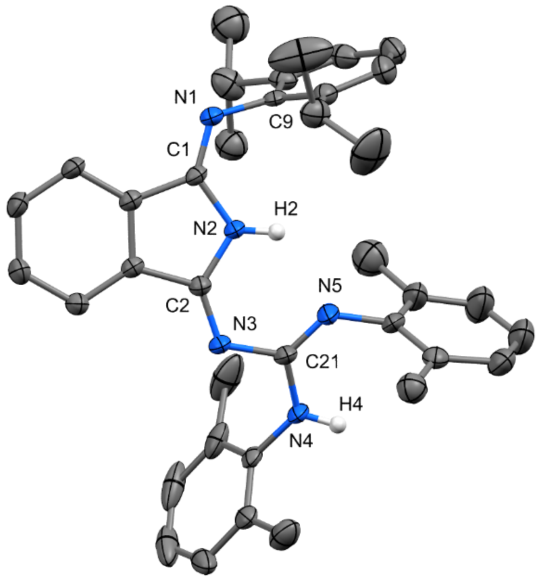

Figure S61. The ORTEP view 50% probability level of molecular structure of **5ba**. Hydrogen atoms are omitted for clarity. Selected bond lengths (Å) and angles (°): N1–C9 1.426(3), N1–C1 1.268(3), C1–N2 1.396(2), N2–C2 1.376(3), C2–N3 1.295(2), N3–C21 1.400(3), N4–C21 1.368(3), N5–C21 1.254(2), C1–N1–C9 120.48(16), N1–C1–C4 125.92(17), C2–N3–C21 118.49(17), N4–C21–N5 123.1(19).

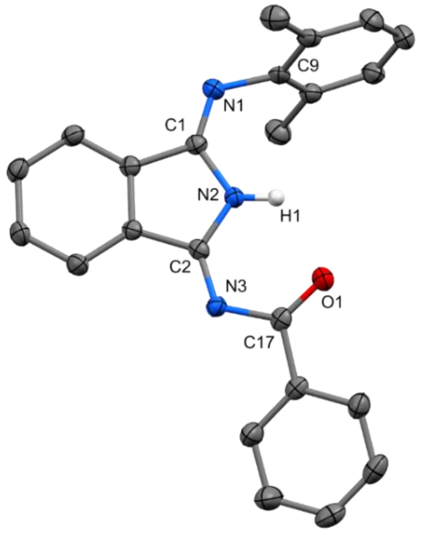

Figure S62. The ORTEP view 50% probability level of molecular structure of **6**. Hydrogen atoms are omitted for clarity. Selected bond lengths (Å) and angles (°): N1–C9 1.427(2), N1–C1 1.267(2), C1–N2 1.407(2), N2–C2 1.368(2), C2–N3 1.296(2), N3–C17 1.392(2), O1–C17 1.227(2), C1–N1–C9 120.28(16), N1–C1–C4 126.08(17), C2–N3–C17 118.77(15), N3–C17–O1 125.08(17).

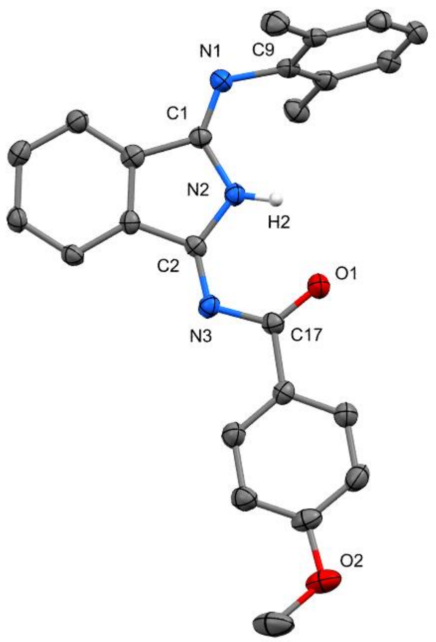

Figure S63. The ORTEP view 50% probability level of molecular structure of **7**. Hydrogen atoms are omitted for clarity. Selected bond lengths (Å) and angles (°): N1–C9 1.428(4), N1–C1 1.268(4), C1–N2 1.408(4), N2–C2 1.373(4), C2–N3 1.302(4), N3–C17 1.393(4), O1–C17 1.238(4), C1–N1–C9 120.9(3), N1–C1–C4 126.1(3), C2–N3–C17 118.4(3), N3–C17–O1 125.1(3).

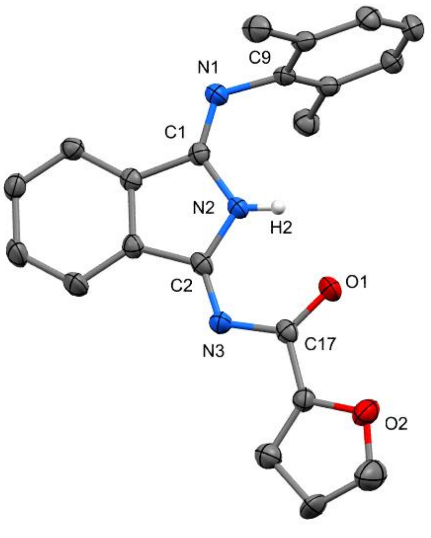

Figure S64. The ORTEP view 50% probability level of molecular structure of **8**. Hydrogen atoms are omitted for clarity. Selected bond lengths (Å) and angles (°): N1–C9 1.430(3), N1–C1 1.270(3), C1–N2 1.399(4), N2–C2 1.367(3), C2–N3 1.300(4), N3–C17 1.388(4), O1–C17 1.233(3), C1–N1–C9 120.5(2), N1–C1–C4 125.9(3), C2–N3–C17 117.7(2), N3–C17–O1 126.4(3).

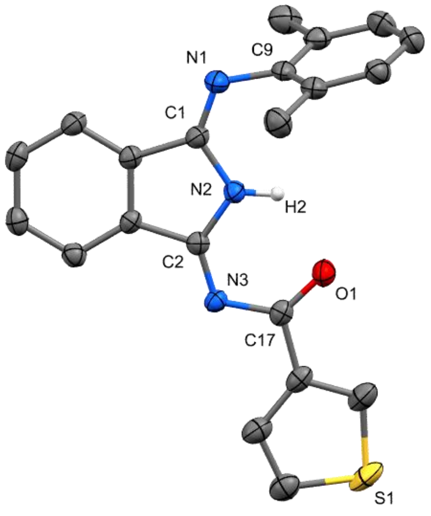

Figure S65. The ORTEP view 50% probability level of molecular structure of **9**. Hydrogen atoms are omitted for clarity. Selected bond lengths (Å) and angles (°): N1–C9 1.427(2), N1–C1 1.268(2), C1–N2 1.405(2), N2–C2 1.365(2), C2–N3 1.303(2), N3–C17 1.393(2), O1–C17 1.233(2), C1–N1–C9 119.67(12), N1–C1–C4 127.16(13), C2–N3–C17 117.14(11), N3–C17–O1 125.38(13).

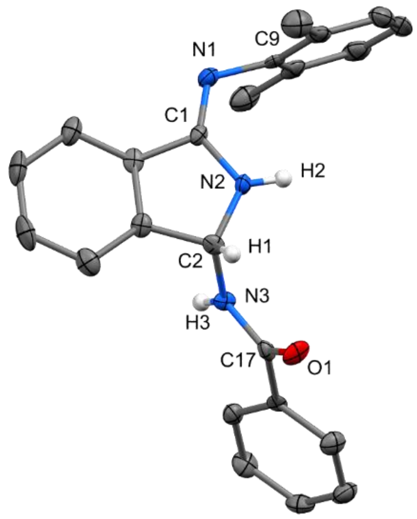

Figure S66. The ORTEP view 50% probability level of molecular structure of **6r**. Hydrogen atoms and diethyl ether molecule are omitted for clarity. Selected bond lengths (Å) and angles (°): N1–C9 1.421(6), N1–C1 1.280(6), C1–N2 1.359(6), N2–C2 1.455(6), C2–N3 1.450(6), N3–C17 1.346(6), O1–C17 1.237(5), C1–N1–C9 117.0(4), N1–C1–C4 124.4(4), C2–N3–C17 122.7(4), N3–C17–O1 121.9(4).

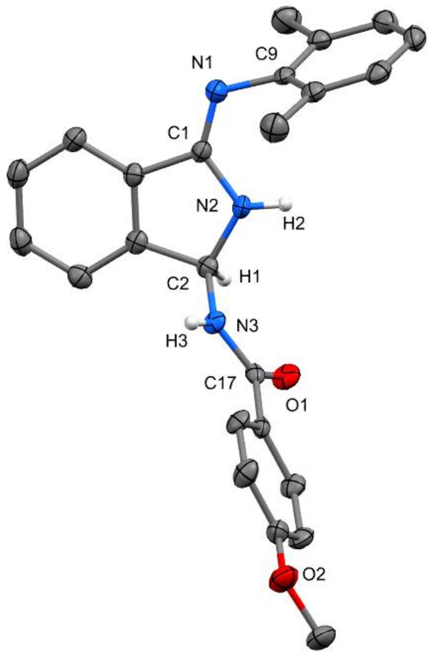

Figure S67. The ORTEP view 50% probability level of molecular structure of **7r**. Hydrogen atoms are omitted for clarity. Selected bond lengths (Å) and angles (°): N1–C9 1.427(2), N1–C1 1.276(2), C1–N2 1.380(2), N2–C2 1.466(2), C2–N3 1.448(2), N3–C17 1.350(2), O1–C17 1.237(2), C1–N1–C9 118.50(16), N1–C1–C4 124.77(17), C2–N3–C17 121.87(15), N3–C17–O1 121.15(17).

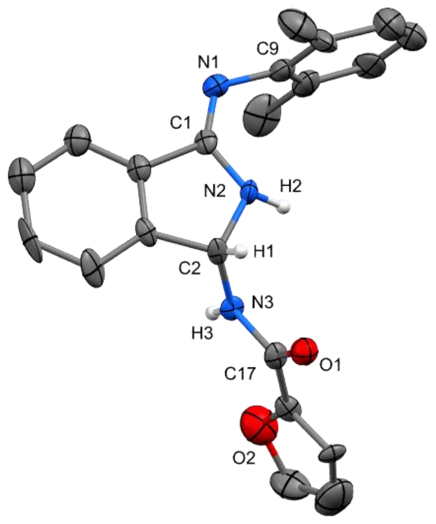

Figure S68. The ORTEP view 50% probability level of molecular structure of **8r**. Hydrogen atoms are omitted for clarity. Selected bond lengths (Å) and angles (°): N1–C9 1.425(18), N1–C1 1.266(17), C1–N2 1.372(18), N2–C2 1.462(16), C2–N3 1.416(18), N3–C17 1.374(15), O1–C17 1.236(16), C1–N1–C9 117.2(12), N1–C1–C4 124.7(13), C2–N3–C17 122.0(10), N3–C17–O1 121.5(12).

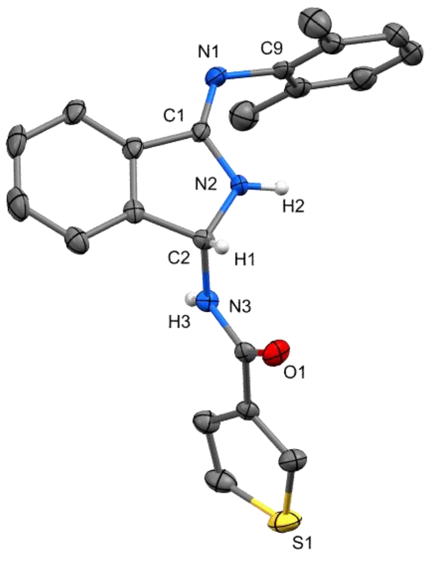

Figure S69. The ORTEP view 50% probability level of molecular structure of **9r**. Hydrogen atoms and molecule of diethyl ether are omitted for clarity. Selected bond lengths (Å) and angles (°): N1–C9 1.426(3), N1–C1 1.283(3), C1–N2 1.366(3), N2–C2 1.459(3), C2–N3 1.456(3), N3–C17 1.347(3), O1–C17 1.227(3), C1–N1–C9 117.25(19), N1–C1–C4 124.4(2), C2–N3–C17 121.81(18), N3–C17–O1 122.7(2).

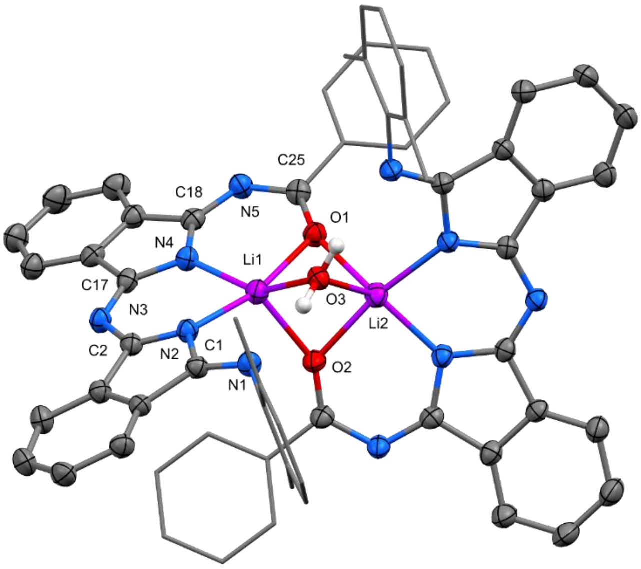

Figure S70. The ORTEP view 50% probability level of molecular structure of (Li**6'**)·H_2O. Hydrogen atoms and dichloromethane molecule are omitted for clarity. Selected bond lengths (Å) and angles (°): N1–C1 1.278(3), C1–N2 1.418(3), N2–C2 1.318(2), C2–N3 1.363(3), N3–C17 1.312(3), N4–C17 1.367(3), N4–C18 1.368(3), N5–C18 1.307(3), N5–C25 1.378(3), O1–C25 1.241(3), N2–Li1 2.109(4), N4–Li1 1.998(4), O1–Li1 2.106(4), O3–Li1 1.994(4), O2–Li1 2.049(4), C1–N1–C9 120.79(17), N2–Li1–N4 86.05(15), Li1–O1–Li2 77.57(15), Li1–O3–Li2 79.16(15).

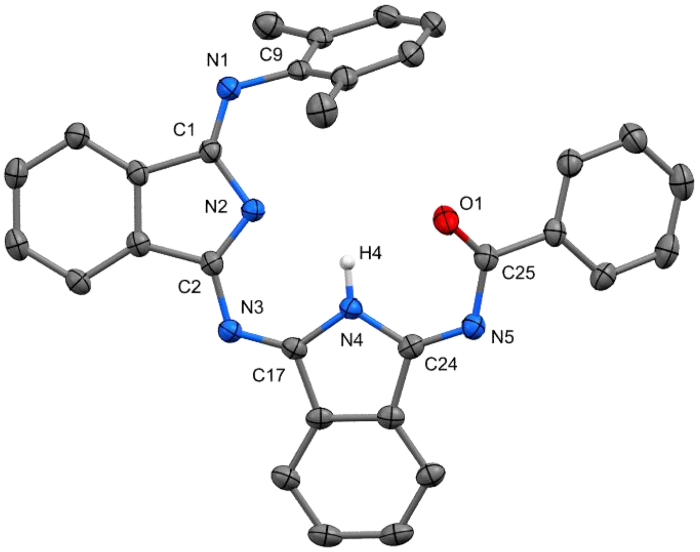

Figure S71. The ORTEP view 50% probability level of molecular structure of **6'**. Hydrogen atoms are omitted for clarity. Selected bond lengths (Å) and angles (°): N1–C9 1.4211(18), N1–C1 1.272(2), C1–N2 1.4219(19), N2–C2 1.3168(18), C2–N3 1.373(2), N3–C17 1.2985(19), N4–C17 1.3810(17), N4–C24 1.3908(19), N5–C24 1.2863(18), N5–C25 1.399(2), O1–C25 1.2237(18), C1–N1–C9 121.13(13), N1–C1–C4 123.26(14), C2–N3–C17 119.97(12), C24–N5–C25 119.15(13).

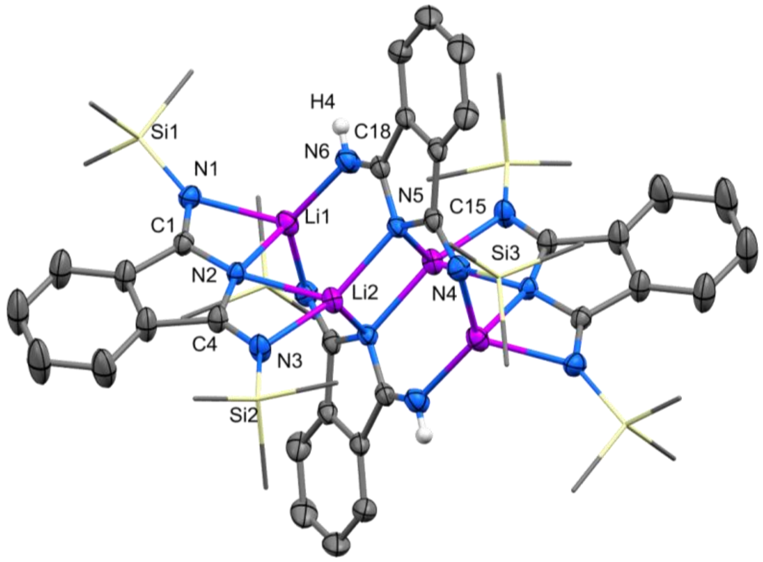

Figure S72. The ORTEP view 50% probability level of molecular structure of (Li**1e**)$_2$**1e**$_2$Li$_2$. Hydrogen atoms are omitted for clarity. Selected bond lengths (Å) and angles (°): N1–C1 1.289(4), C1–N2 1.376(3), N2–C4 1.371(3), C4–N3 1.295(3), N3–Si2 1.727(2), N4–C15 1.292(4), N5–C15 1.387(3), N5–C18 1.374(3), N6–C18 1.295(4), N1–Li1 2.108(5), N2-Li1 2.134(5), N2-Li2 2.168(5), N3-Li2 2.069(5), N5-Li2 2.128(5), N6-Li1 2.019(5), C1–N1–Si1 132.9(2), N1–Li1–N2 66.09(16), N2–Li2–N3 65.93(15), N2–Li1–N6 108.3(2), N2-Li2-N5 117.1(2). Symmetry code: -x, -y, -z.

6 General methods, preparation and characterization of compounds

*6.1 General procedure for synthesis of compounds Li**1a**, Li**1b**, Li**1c**, Li**1d** and Li**1e***

Substituted aniline was dissolved in hexane, and *n*-butyllithium was added at 0 °C. The yellow solution was stirred for another hour, concentrated in vacuo, and the yellow crystalline intermediate was isolated. Intermediate was dissolved in THF and a solution of 1,2-dicyanobenzene in THF was added while cooling to -50°C. A dark green solution was formed, which was left to stir for 12 hours and subsequently, at a low temperature (-20°C), the product was recrystallized from THF or a mixture of THF and petroleum ether. The product was filtered off, washed twice with hexane, and dried in vacuo. Reactions were performed under an argon atmosphere using standard Schlenk techniques.

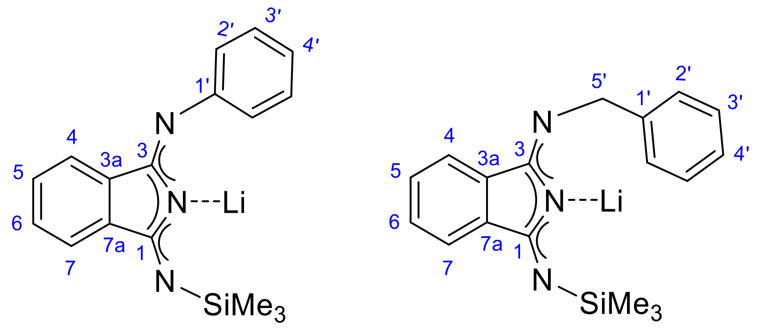

Figure S73. Atom numbering for compounds Li**1c**, Li**1d** and Li**1e**.

Compound Li**1c**

The reaction was performed using N-(trimethylsilyl)aniline (1.842 g; 11.14 mmol), *n*-butyllithium (7.31 mL; 1.6 M solution) and 1,2-dicyanobenzene (1.428 g; 11.14 mmol) to obtain the product as a white powder (2.852 g, 69%). **^{1}H NMR** (500 MHz, THF-d_8, 25 °C): δ 7.80 (m, 1H, H4), 7.61 (m, 1H, H7), 7.40–7.36 (m, 2H, H5, H6), 7.23-7.05 (m, 4H, H2', H3'), 6.81 (m, 1H, H4'), 0.25 (s, 9H, -Si(C**H**$_3$)$_3$) ppm.**^{13}C NMR** (125 MHz, THF-d_8, 25 °C): δ 177.9 (C1), 165.1 (C3), 154.7 (C1'), 145.0 (C7a), 137.3 (C3a), 129.4 (C5), 128.8 (C6), 128.1 (C2'), 124.4 (C3'), 122.0 (C4), 121.6 (C7), 121.2 (C4'), 2.6 (-Si(**C**H$_3$)$_3$) ppm. **^{7}Li NMR** (194 MHz, THF-d_8, 25 °C): δ - 0.85 ppm.

Compound Li**1d**

The reaction was performed using N-(trimethylsilyl)benzylamine (2.188 g; 12.20 mmol), *n*-butyllithium (8.01 mL; 1.6 M solution) and 1,2-dicyanobenzene (1.614 g; 12.20 mmol) to obtain the product as a dark powder (3.024 g, 64%). **^{1}H NMR** (500 MHz, THF-d_8, 25 °C): δ 7.80 (m, 1H, H4), 7.58 (m, 1H, H7), 7.30 (m, 4H, H5, H6, H2'), 7.12 (m, 2H, H3'), 7.03 (m, 1H, H4'), 4.81 (s, 1H, H5'), 0.22 (s, 9H, -Si(C**H**$_3$)$_3$) ppm.**^{13}C NMR** (125 MHz, THF-d_8, 25 °C): δ 176.8 (C1), 167.6 (C3), 144.5 (C1'), 144.2 (C7a), 138.1 (C3a), 129.2 (C5), 128.4 (C6), 128.1 (4C, C(2'),C(3')), 125.7 (C4'), 122.0 (C4), 121.3 (C7), 54.1 (C5'), 2.9 (-Si(**C**H$_3$)$_3$) ppm. **^{7}Li NMR** (194 MHz, THF-d_8, 25 °C): δ 1.95 ppm.

Compound Li**1e**

The reaction was performed using bis(trimethylsilyl)amine (5.330 g; 33.06 mmol), *n*-butyllithium (13.2 mL; 2.5 M solution) and 1,2-dicyanobenzene (4.231 g; 33.06 mmol) to obtain the product as a greenish powder (5.958 g, 61%). **^{1}H NMR** (500 MHz, THF-d_8, 25 °C): δ 7.58 (m, 2H, H7, H4)); 7.33 (m, 2H, H5, H6)); 0.22 (s, 18H, -Si(C**H**$_3$)$_3$) ppm. **^{13}C NMR** (125 MHz, THF-d_8, 25 °C): δ 175.3 (C1, C3); 141.6 (C7a, C3a); 129.5 (C5, C6); 121.8 (C7, C4)); 2.5 (-Si(**C**H$_3$)$_3$) ppm. **^{7}Li NMR** (194 MHz, THF-d_8, 25 °C): δ 1.07 ppm. **^{29}Si NMR** (99 MHz, THF-d_8, 25 °C): δ -8.03 (-**Si**(CH$_3$)$_3$) ppm.

Compound Li**1a**

The reaction was performed using *N*-(trimethylsilyl)-2,6-dimethylaniline (7.052 g; 36.47 mmol), *n*-butyllithium (22.8 mL; 1.6 M solution) and 1,2-dicyanobenzene (4.673 g; 36.47 mmol) to obtain the product as a yellow powder (6.090 g, 51%). ^{1}**H NMR** (500 MHz, THF-d_8, 25 °C): δ 7.86 (m, 1H, H4), 7.61 (m, 1H, H7), 7.38 (m, 2H, H5, H6), 6.87 (m, 2H, H3ˈ), 6.66 (m, 1H, H4ˈ), 2.04 (s, 6H, 2ˈ-CH_3), 0.24 (s, 9H, -Si(C$\mathbf{H_3}$)$_3$) ppm.^{13}**C NMR** (125 MHz, THF-d_8, 25 °C): δ 177.2 (C1), 163.4 (C3), 152.9 (C1ˈ), 144.0 (C7a), 137.8 (C3a), 129.5 (C5), 128.8 (C6), 128.5 (C2ˈ), 127.2 (C3ˈ), 122.0 (C4), 121.6 (C7), 120.8 (C4ˈ), 18.8 (2ˈ-**C**H_3), 2.5 (-Si(**C**H_3)$_3$) ppm. ^{7}**Li NMR** (194 MHz, THF-d_8, 25 °C): δ 0.77 ppm.

Compound Li**1b**

The reaction was performed using *N*-(trimethylsilyl)-2,6-diisopropylaniline (7.060 g; 28.30 mmol), *n*-butyllithium (17.7 mL; 1.6 M solution) and 1,2-dicyanobenzene (3.627 g; 28.30 mmol) to obtain the product as a yellow powder (5.101 g, 47%). ^{1}**H NMR** (500 MHz, THF-d_8, 25 °C): δ 7.84 (m, 1H, H4), 7.58 (m, 1H, H7), 7.47-7.26 (m, 2H, H5, H6), 6.93 (m, 2H, H3ˈ), 6.77 (m, 1H, H4ˈ), 3.01 (m, 2H, 2ˈ - C**H**(CH_3)$_2$), 1.16, 1.09 (d, 6H, 2ˈ - CH(C$\mathbf{H_3}$)$_2$), 0.20 (s, 9H, -Si(C$\mathbf{H_3}$)$_3$) ppm.^{13}**C NMR** (125 MHz, THF-d_8, 25 °C): δ 177.5 (C1), 163.4 (C3), 150.7 (C1ˈ), 144.1 (C7a), 138.4 (C2ˈ), 137.7 (C3a), 129.5 (C5), 128.8 (C6), 122.0 (C4), 121.9 (C3ˈ), 121.6 (C7), 121.3 (C4ˈ), 29.0 (2ˈ - **C**H(CH_3)$_2$), 23.3, 22.09 (2ˈ - CH(**C**H_3)$_2$), 2.7 (-Si(**C**H_3)$_3$) ppm**.** ^{7}**Li NMR** (194 MHz, THF-d_8, 25 °C): δ 1.68 ppm.

6.2 General procedure for synthesis of compounds 1a, 1b and 1c

Lithium complex was dissolved in methanol on air, and two molar equivalents of water were added. The solution was left to stir for 12 hours and then the solvent was evaporated under vacuo. The product was extracted by dichloromethane, filtrated, dried in vacuo and isolated in the form of a yellow powder.

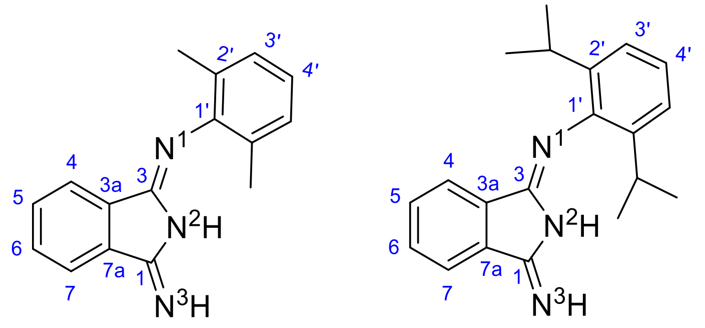

3 **4**

Figure S74. Atom numbering for compounds **1a** and **1b**.

Compound 1a

The reaction was performed using lithium complex **1** (2.451 g; 7.49 mmol) and water (0.135 mL; 14.98 mmol) to obtain the product as a yellow powder (1.865 g, 84%), m.p. 204–206°C. **^{1}H NMR** (500 MHz, DMSO-*d*$_6$, 25 °C): δ 8.59 (s, 1H, NH), 8.35 (s, 1H, NH), 7.91–7.86 (m, 2H, H4, H7), 7.63–7.57 (m, 2H, H5, H6), 6.95 (m, 2H, H3'), 6.79 (m, 1H, H4'), 1.95 (s, 6H, 2'-C**H$_3$**) ppm. **^{13}C NMR** (125 MHz, DMSO-*d*$_6$, 25 °C): δ 170.93, 164.36 (C1, C3), 149.95 (C1'), 139.60, 135.83 (C3a, C7a), 131.14, 130.35 (C5, C6), 127.22 (C3'), 126.66 (C2'), 121.50 (C4'), 121.68, 120.89 (C7, C4), 18.46 (2' -**C**H$_3$) ppm.

HRMS (ESI): m/z calculated for $C_{16}H_{16}N_3$+ ([M+H]$^+$): 250.1344, observed: 250.1340.

Compound 1b

The reaction was performed using lithium complex **2** (2.931 g; 7.64 mmol) and water (0.275 mL; 15.28 mmol) to obtain the product as a yellow powder (2.031 g, 87%), m.p. 291–293°C. **^{1}H NMR** (500 MHz, DMSO-*d*$_6$, 25 °C): δ 8.60 (s, 1H, NH), 8.30 (s, 1H, NH), 7.93–7.85 (m, 2H, H4, H7), 7.64–7.57 (m, 2H, H5, H6), 7.02 (m, 2H, H3'), 6.9 (m, 1H, H4'), 2.81 (m, 2H, 2'-C**H**(CH$_3$)$_2$), 1.13–1.01 (m, 12H, 2' - CH(C**H$_3$**)$_2$) ppm. **^{13}CNMR** (125 MHz, DMSO-*d*$_6$, 25 °C): δ 170.81, 165.12 (C1, C3), 147.70 (C1'), 139.59, 135.94 (C3a, C7a), 136.91 (C2'), 131.06, 130.30 (C5, C6), 122.44 (C4'), 122.07 (C3'), 121.60, 120.86 (C7, C4), 28.05 (2' - **C**H(CH$_3$)$_2$), 23.33, 23.04 (2' - CH(**C**H$_3$)$_2$) ppm. **^{15}N NMR** (50 MHz, THF-*d*$_8$, 25 °C): δ 274.4 (N1) ppm, (N2), (N3) were not detected due to NH signal broadening even at lower temperature (–100 °C)

HRMS (ESI): m/z calculated for $C_{20}H_{23}N_3$+ ([M+H]$^+$): 306.1970, observed: 306.1963.

Compound 1c

The reaction was performed using lithium complex prepared by general procedure for synthesis of compounds **1** and **2** using *N*-(trimethylsilyl)-aniline (1.820 g; 11.01 mmol), *n*-butyllithium (7.23 mL; 1.6 M solution) and 1,2-dicyanobenzene (1.440 g; 11.01 mmol) to obtain a greenish powder (1.813 g, 55%) and then water (0.218 mL; 12.11 mmol) for hydrolysis to obtain the product as a pale yellow powder (1.112 g, 83%).

6.3 General procedure for the synthesis of compounds 5aa – 5bb

The corresponding isoindole was dissolved in 30 mL of toluene at 50 °C. Subsequently, one molar equivalent of carbodiimide was added to the solution. The reaction mixture was stirred for 48 hours at 100 °C, then cooled to room temperature and concentrated in vacuo. The product was further washed with hexane, dried and isolated as a yellow powder.

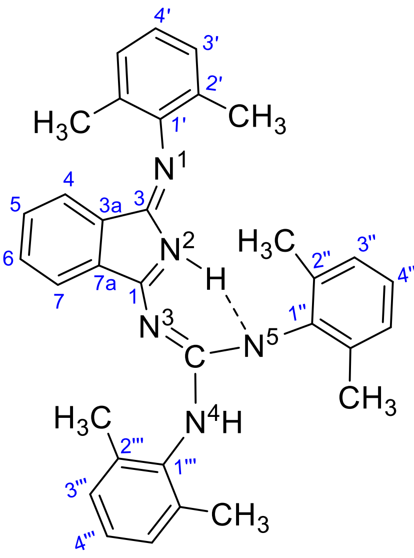

5aa

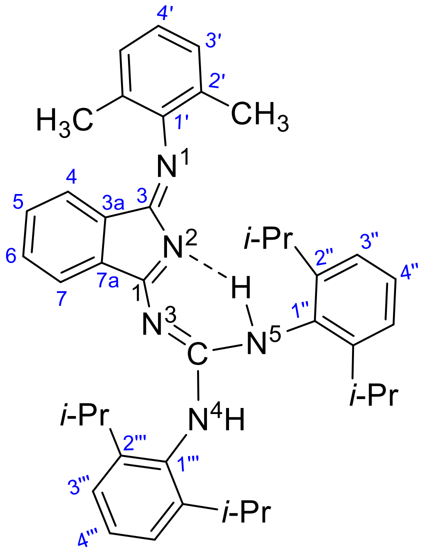

5ab

Figure S75. Atom numbering for compounds **5aa** and **5ab**.

Compound 5aa

The reaction was performed using isoindole **1a** (0.490 g; 1.97 mmol) and 1,6-bis(dimethylphenyl)carbodiimide (0.92 g; 1.97 mmol) in toluene (30 mL) to obtain the final product as a yellow powder (0.934 g, 95%), m.p. 207–209°C. **^{1}H NMR** (600 MHz, THF-d_8, 25 °C): δ 12.03 (s, 1H, N2H), 7.94 (dm, 1H, H4, $^{3}J_{4\text{-}5}$ = 7.4 Hz), 7.53 (ddd, 1H, H5, $^{3}J_{5\text{-}4}$ = $^{3}J_{5\text{-}6}$ = 7.4 Hz, $^{4}J_{5\text{-}7}$ = 1.0 Hz), 7.41 (ddd, 1H, H6, $^{3}J_{6\text{-}5}$ = $^{3}J_{6\text{-}7}$ = 7.4 Hz, $^{4}J_{6\text{-}4}$ = 0.9 Hz), 7.30 (dm, 1H, H7, $^{3}J_{7\text{-}6}$ = 7.4 Hz), 6.94–7.07 (m, 6H, H3'', H4'', H3''', H4'''), 6.89 (m, 2H, H3'), 6.79 (s, 1H, N4H), 6.71 (m, 1H, H4'), 2.20 (s, 6H, 2'''-CH_3), 2.15 (s, 6H, 2''-CH_3), 2.06 (s, 6H, 2'-CH_3) ppm. **^{13}C NMR** (150 MHz, THF-d_8, 25 °C): δ 168.56 (C1), 158.36 (C3), 156.84 (C-guanidine), 149.30 (C1'), 139.98 (C7a), 139.39 (C1''), 137.50 (C3a), 137.32 (C1'''), 137.06 (C2'''), 133.59 (C2''), 131.53 (C5), 131.11 (C6), 129.00 (C3''), 128.11 (C3'''), 127.98 (C3'), 127.61 (C2'), 127.16 (C4''), 126.06 (C4'''), 123.11 (C4'), 122.43 (C4), 122.12 (C7), 18.88 (2' -CH_3), 18.29 (2'', 2'''-CH_3) ppm.**^{15}N NMR** (60 MHz, THF-d_8, 25 °C): δ 271.2 (N1), 189.7 (N3), 185.7 (N5), 150.7 (N2), 99.1 (N4) ppm.
HRMS (ESI): m/z calculated for $C_{33}H_{33}N_5$+ ([M+H]$^+$): 500.2814, observed: 500.2810.

Compound 5ab

The reaction was performed using isoindole **1a** (0.479 g; 1.92 mmol) and 1,6-bis(diisopropylphenyl)carbodiimide (0,697 g; 1,92 mmol) in toluene (30 mL) to obtain the product as a yellow powder (1.106 g, 94%), m.p. 245–246°C. **^{1}H NMR** (600 MHz, THF-*d$_8$*, 25 °C): δ 12.52 (s, 1H, N5H), 7.92 (dm, 1H, H4, $^{3}J_{4\text{-}5}$ = 7.4 Hz), 7.49 (ddd, 1H, H5, $^{3}J_{5\text{-}4}$ = $^{3}J_{5\text{-}6}$ = 7.4 Hz, $^{4}J_{5\text{-}7}$ = 1.1 Hz), 7.37 (ddd, 1H, H6, $^{3}J_{6\text{-}5}$ = $^{3}J_{6\text{-}7}$ = 7.4 Hz, $^{4}J_{6\text{-}4}$ = 1.0 Hz), 7.27 (dm, 1H, H7, $^{3}J_{7\text{-}6}$ = 7.4 Hz), 7.16–7.23 (m, 4H, H4'', H3'', H4'''), 7.13 (m, 2H, H3'''), 6.76–6.79 (m, 3H, H3', N4H), 6.60 (m, 1H, H4'), 3.11–3.22 (m, 4H, 2'' -**CH**(CH_3)$_2$, 2''' -**CH**(CH_3)$_2$), 2.03 (s, 6H, 2' -CH_3), 1.30 (d, 6H, 2''-CH**(CH_3)$_2$**, $^{3}J_{CH3\text{-}CH}$ = 7.0 Hz), 1.16 (d, 6H, 2''' -CH**(CH_3)$_2$**, $^{3}J_{CH3\text{-}CH}$ = 6.9 Hz), 1.09 (d, 6H, 2'''-CH**(CH_3)$_2$**, $^{3}J_{CH3\text{-}CH}$ = 7.0 Hz), 0.89 (d, 6H, 2'' -CH**(CH_3)$_2$**, $^{3}J_{CH3\text{-}CH}$ = 6.8 Hz) ppm. **^{13}C NMR** (150 MHz, THF-*d$_8$*, 25 °C): δ 171.72 (C1), 160.46 (C3), 158.73 (C- guanidine), 149.80 (C1'), 147.31 (C2'''), 145.58 (C2''), 140.78 (C7a), 138.16 (C3a), 134.53 (C1''), 134.00 (C1'''), 131.17 (C5), 130.87 (C6), 128.27, 128.19 (C4'', C4'''), 127.78 (C3'), 127.26 (C2'), 123.97 (C3''), 123.27 (C3'''), 122.77 (C4'), 122.33 (C4), 121.89 (C7), 29.43 (2''-**CH**(CH_3)$_2$), 29.20 (2'''-**CH**(CH_3)$_2$), 25.32, 21.72 (2''-CH**(CH_3)$_2$**), 24.05, 23.59 (2'''-CH**(CH_3)$_2$**), 18.47 (2'-CH_3) ppm. **^{15}N NMR** (60 MHz, THF-*d$_8$*, 25 °C): δ 276.0 (N1), 199.7 (N2), 182.5 (N3), 131.5 (N5), 97.6 (N4) ppm.
HRMS (ESI): m/z calculated for $C_{41}H_{49}N_5$+ ([M+H]+): 612.4066, observed: 612.4059.

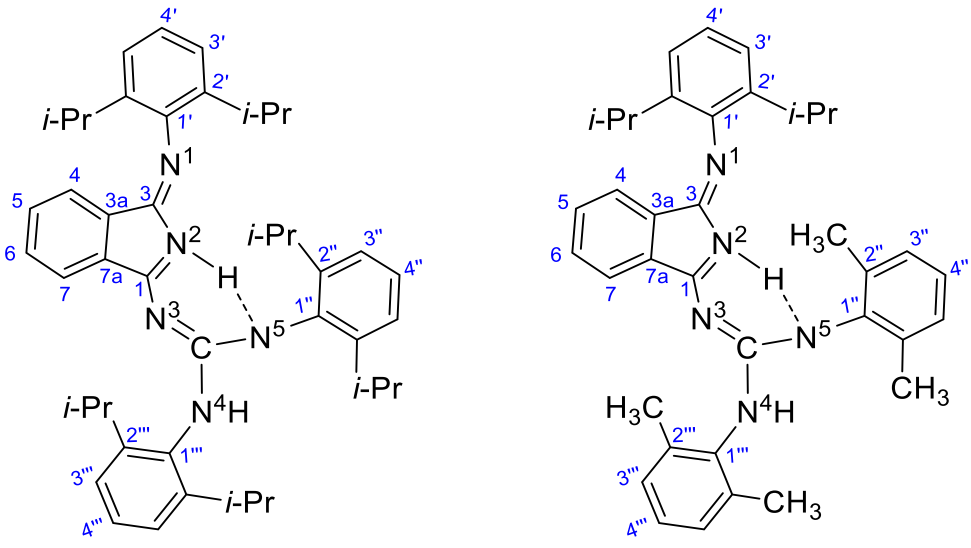

5bb **5ba**

Figure S76. Atom numbering for compounds **5bb** and **5ba**.

Compound 5bb

The reaction was performed using isoindole **1b** (0.528 g; 1.72 mmol) and 1,6-bis(diisopropylphenyl)carbodiimide (0,627 g; 1,72 mmol) in toluene (30 mL) to obtain the product as a yellow powder (1.062 g, 92%), m.p. 174–176°C. **^{1}H NMR** (600 MHz, THF-*d$_8$*, 25 °C): δ 11.44 (s, 1H, N2H), 7.94 (dm, 1H, H4, $^{3}J_{4\text{-}5}$ = 7.5 Hz), 7.51 (ddd, 1H, H5, $^{3}J_{5\text{-}4}$ = $^{3}J_{5\text{-}6}$ = 7.5 Hz, $^{4}J_{5\text{-}7}$ = 0.8 Hz), 7.39 (ddd, 1H, H6, $^{3}J_{6\text{-}5}$ = $^{3}J_{6\text{-}7}$ = 7.5 Hz, $^{4}J_{6\text{-}4}$ = 0.7 Hz), 7.28 (dm, 1H, H7, $^{3}J_{7\text{-}6}$ = 7.5 Hz), 7.23 (m, 1H, H4'''), 7.15–7.18 (m, 3H, H4'', H3''), 7.12 (m, 2H, H3'''), 6.97 (m, 2H, H3'), 6.84 (m, 1H, H4'), 6.76 (s, 1H, N4H), 3.13–3.22 (m, 4H, 2'' -**CH**(CH_3)$_2$, 2''' -**CH**(CH_3)$_2$), 2.90–2.99 (m, 2H, 2' -**CH**(CH_3)$_2$), 1.28 (d, 6H, 2'' -CH**(CH_3)$_2$**, $^{3}J_{CH3\text{-}CH}$ = 7.0 Hz), 1.07-1.16 (m, 24H, 2' ,2'', 2''' -CH**(CH_3)$_2$**), 0.82 (d, 6H, 2''' -CH**(CH_3)$_2$**, $^{3}J_{CH3\text{-}CH}$ = 6.7 Hz) ppm. **^{13}C NMR** (150 MHz, THF-*d$_8$*, 25 °C): δ 169.02 (C1), 159.10 (C3), 158.16 (C- guanidine), 147.38 (C2'''), 146.87 (C1'), 144.92 (C2''), 140.11 (C7a), 137.94 (C3a), 137.49 (C2'), 135.77 (C1''), 134.26 (C1'''), 131.43 (C5), 131.06 (C6), 128.23 (C4'''), 127.57 (C4''), 124.14 (C3''), 123.60 (C4'), 123.29 (C3'''), 123.10 (C3'), 122.37 (C4), 122.05 (C7), 29.13 (2' -**CH**(CH_3)$_2$), 29.06 (2'', 2''' -**CH**(CH_3)$_2$), 25.61, 24.14, 23.69, 23.64, 23.20, 22.59 (2', 2'', 2''' -CH**(CH_3)$_2$**) ppm. **^{15}N NMR** (60 MHz, THF-*d$_8$*, 25 °C): δ 190.5 (N3), 188.3 (N5), 141.0 (N2), 98.5 (N4) ppm. N1 was not detected.
HRMS (ESI): m/z calculated for $C_{45}H_{57}N_5$+ ([M+H]+): 668.4692, observed: 668.4688.

Compound 5ba

The reaction was performed using isoindole **1b** (0.596 g; 1.95 mmol) and 1,6-bis(dimethylphenyl)carbodiimide (0,488 g; 1,95 mmol) in toluene (30 mL) to obtain the product as a yellow powder (1.019 g, 94%), m.p. 225–227°C. **^{1}H NMR** (600 MHz, THF-*d$_8$*, 25 °C): δ 12.16 (s, 1H, N2H), 7.97 (dm, 1H, H4, $^{3}J_{4\text{-}5}$ = 7.4 Hz), 7.54 (ddd, 1H, H5, $^{3}J_{5\text{-}4}$ = $^{3}J_{5\text{-}6}$ = 7.4 Hz, $^{4}J_{5\text{-}7}$ = 1.0 Hz), 7.43 (ddd, 1H, H6, $^{3}J_{6\text{-}5}$ = $^{3}J_{6\text{-}7}$ = 7.4 Hz, $^{4}J_{6\text{-}4}$ = 1.0 Hz), 7.32 (dm, 1H, H7, $^{3}J_{7\text{-}6}$ = 7.4 Hz), 6.92–7.06 (m, 8H, H3', H3'', H3''', H4'', H4'''), 6.87 (m, 1H, H4'), 6.75 (s, 1H, N4H), 2.97 (m, 2H, 2' **-CH**(CH_3)), 2.18 (s, 6H, 2'' -CH_3), 2.08 (s, 6H, 2''' -CH_3), 1.09 (m, 12H, 2' -CH**(CH_3)$_2$**) ppm. **^{13}C NMR** (150 MHz, THF-*d$_8$*, 25 °C): δ 168.14 (C1), 158.53 (C3), 156.75 (C- guanidine), 147.13 (C1'), 139.98 (C7a), 139.46 (C1'''), 138.01 (C2'), 137.29 (C3a), 137.04 (C1''), 133.42 (C2'''), 132.60 (C2''), 131.58 (C5), 131.18 (C6), 128.92 (C3'''), 128.10 (C3''), 127.14 (C4''), 125.96 (C4'''), 123.79 (C4'), 122.98 (C3'), 122.48 (C4), 122.23 (C7), 28.98 (2' -**CH**(CH_3)$_2$), 23.49 (2'-CH**(CH_3)$_2$**), 18.87 (2'' -CH_3), 18.31 (2 '' -CH_3) ppm.**^{15}N NMR** (60 MHz, THF-*d$_8$*, 25 °C): δ 270.8 (N1), 190.4 (N3), 184.8(N4), 152.0 (N2), 98.8 (N5) ppm.

HRMS (ESI): m/z calculated for $C_{37}H_{41}N_5$+ ([M+H]+): 556.3440, observed: 556.3436.

Deprotonation attempts of compounds 5aa - 5bb

Table S10. Deprotonation conversion evaluated by ^{1}H NMR spectroscopy using N2**H** as diagnostical signal

Agent/Ligand	**5aa**	**5ab**	**5ba**	**5bb**
**LDA**	100%	100%	46%	47%
**MeLi**	95%	93%	30%	36%

6.4 ***General procedures for synthesis compounds 6 – 9***

General procedure A

The corresponding isoindole was dissolved in 30 mL of diethylether. The solution was cooled to 0 °C, and one molar equivalent of methyllithium was added. The reaction mixture was stirred for 1 hour at room temperature. Subsequently, one molar equivalent of acyl chloride was added to the solution. The solution was stirred for 4 hours with the formation of a yellow precipitate, then concentrated in vacuo to half. The product was filtrated from a solution and extracted with benzene. After evaporation of benzene in vacuo the product was dried and isolated as a yellow powder. The reactions were carried out under an argon atmosphere using standard Schlenk techniques.

General procedure B

Corresponding isoindole was dissolved in 50 mL of diethylether. Subsequently, one molar equivalent of acyl chloride and two equivalents of triethylamine were added to the solution. Solution was stirred for 12 hours and filtrated on a frit. Product was gained from solution by slow evaporation of diethyl ether. After filtration the product was dried and isolated as yellow crystals.

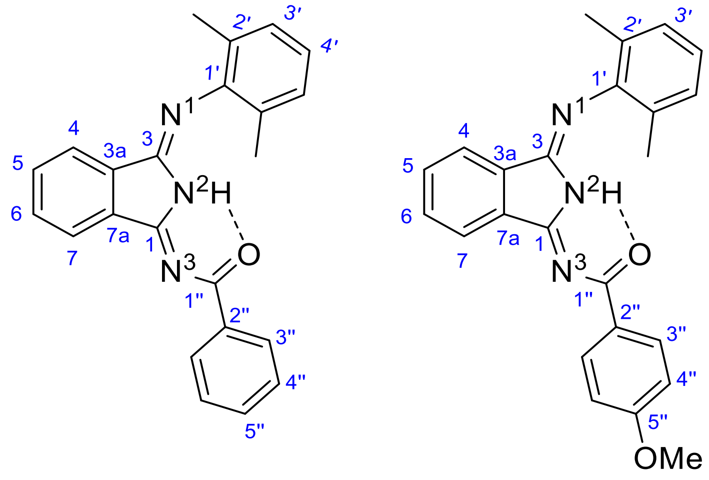

6 **7**

Figure S77. Atom numbering for compounds **6** and **7**. Only Z isomer (majority) is shown.

Compound 6

A The reaction was performed using isoindole **1a** (0.344 g; 1.38 mmol), methyllithium (0.445 mL; 3.1M in diethoxymethane) and benzoyl chloride (0.157mL; 1.38 mmol) in diethyl ether (30 mL) to obtain the product as a yellow powder (0.273 g, 56%).

B The reaction was performed using isoindole **1a** (0.402 g; 1.61 mmol), benzoyl chloride (0.187mL; 1.61 mmol) and triethylamine (0.450 mL; 3,22 mmol) in diethyl ether (50 mL) to obtain the product as yellow crystals (0.460 g, 81%), m.p. 168–169°C.

The NMR spectra contained two sets of signals at room temperature in ~2:1 ratio:

Majority signals: **^{1}H NMR** (600 MHz, THF-d_8, 25 °C): δ 10.48 (s, 1H, N2H), 8.26 (m, 2H, H3''), 8.14 (m, 1H, H4, $^{3}J_{4\text{-}5}$ = 7.5 Hz), 8.10 (dm, 1H, H7, $^{3}J_{7\text{-}6}$ = 7.5 Hz), 7.81 (ddd, 1H, H5, $^{3}J_{5\text{-}4}$ = $^{3}J_{5\text{-}6}$ = 7.5 Hz, $^{4}J_{5\text{-}7}$ = 1.0 Hz), 7.75 (ddd, 1H, H6, $^{3}J_{6\text{-}5}$ = $^{3}J_{6\text{-}7}$ = 7.5 Hz, $^{4}J_{6\text{-}4}$ = 1.0 Hz), 7.53 (m, 1H, H5''), 7.43 (m, 2H, H4''), 7.07–7.10 (m, 2H, H3'), 6.96 (m, 1H, H4'), 2.11 (s, 6H, 2'-CH_3) ppm. **^{13}C NMR** (150 MHz, THF-d_8, 25 °C): δ 179.27 (C1''), 160.31 (C1), 150.34 (C3), 146.58 (C1'), 136.77 (C2''), 136.16 (C7a), 135.03 (C3a), 133.95 (C5), 133.38 (C5''), 132.71 (C6), 130.63 (C3''), 128.75-128.83 (m, C3', C4''), 127.78 (C2'), 124.60 (C4'), 124.10 (C7), 123.34 (C4), 17.95 (2' -CH_3) ppm. **^{15}N NMR** (60 MHz, THF-d_8, 25 °C): δ 265.0 (N1), 134.6 (N2) ppm. N3 was not detected.

Minority signals: **^{1}H NMR** (600 MHz, THF-d_8, 25 °C): δ 11.16 (s, 1H, N2H), 8.39 (m, 2H, H3''), 8.11 (m, 1H, H7), 7.63 (ddd, 1H, H6, $^{3}J_{6\text{-}5}$ = $^{3}J_{6\text{-}7}$ = 7.6 Hz, $^{4}J_{5\text{-}7}$ = 1.0 Hz), 7.56 (m, 1H, H5''), 7.47 (m, 2H, H4 ''), 7.40 (ddd, 1H, H5, $^{3}J_{5\text{-}4}$ = $^{3}J_{5\text{-}6}$ =

7.6 Hz, $^4J_{5\text{-}7}$ = 0.8 Hz), 7.07–7.10 (m, 2H, H3'), 6.99 (m, 1H, H4'), 6.61 (dm, 1H, H4, $^3J_{4\text{-}5}$ = 7.6 Hz), 2.05 (s, 6H, 2' - CH_3) ppm. **^{13}C NMR** (150 MHz, THF-d_8, 25 °C): δ 179.04 (C1''), 160.48 (C1), 153.31 (C3), 147.29 (C1'), 137.71 (7a), 137.29 (C2''), 133.87 (C5), 133.15 (C5''), 132.84 (C6), 130.66 (C3''), 129.76 (C3a), 128.75-128.83 (m, C3', C4''), 127.20 (C2'), 124.86 (C4), 124.17 (C4'), 124.08 (C7), 18.04 (2' -CH_3) ppm. **^{15}N NMR** (60 MHz, THF-d_8, 25 °C): δ 281.5 (N1), 140.8 (N2) ppm. N1 and N2 were detected indirectly form N,H-HMBC and N,H-HSQC, respectively. N3 was not detected.
HRMS (ESI): m/z calculated for $C_{23}H_{19}N_3O$+ ([M+H]+): 354.1606, observed: 354.1604.

Compound 8

A The reaction was performed using isoindole **1a** (0.379 g; 1.52 mmol), methyllithium (0.490 mL; 3.1M in diethoxymethane) and 4-methoxybenzoyl chloride (0.253 g; 1.52 mmol) in diethyl ether (30 mL) to obtain the product as a yellow powder (0.309 g, 53%).
B The reaction was performed using isoindole **1a** (0.549 g; 2.20 mmol), 4-methoxybenzoyl chloride (0.380g; 2.20 mmol) and triethylamine (0.614 mL; 4.40 mmol) in diethyl ether (60 mL) to obtain the product as yellow crystals (0.642 g, 76%), m.p. 141–143°C.
The NMR spectra contained two sets of signals at room temperature in ~2:1 ratio:
Majority signals:**^{1}H NMR** (600 MHz, THF-d_8, 25 °C): δ 10.53 (s, 1H, N2H), 8.23 (m, 2H, H3''), 8.13 (dm, 1H, H4, $^3J_{4\text{-}5}$ = 7.5 Hz), 8.09 (dm, 1H, H7, $^3J_{7\text{-}6}$ = 7.3 Hz), 7.80 (ddd, 1H, H5, $^3J_{5\text{-}4}$ =7.5 Hz, $^3J_{5\text{-}6}$ = 7.3 Hz), 7.74 (ddd, 1H, H6, $^3J_{6\text{-}5}$ = 7.3 Hz, $^3J_{6\text{-}7}$ = 7.4 Hz), 7.08 (m, 2H, H3'), 7.01–6.94 (m, 3H, H4', H4''), 3.84 (s, 3H, 5'' - O – **CH_3**), 2.10 (s, 6H, 2' - CH_3) ppm. **^{13}C NMR** (150 MHz, THF-d_8, 25 °C): δ 178.76 (C1''), 164.56 (C5''), 160.15 (C1), 150.43 (C3), 146.64 (C1'), 136.40 (C7a), 134.96 (C3a), 133.83 (C5), 132.82 (C3''), 132.64 (C6), 129.53 (C2''), 128.79 (C3'), 127.77 (C2'), 124.56 (C4'), 123.98 (C7), 123.30 (C4), 114.02 (C4''),55.50 (5'' - O - **CH_3**), 17.95 (2' -CH_3) ppm. **^{15}N NMR** (60 MHz, THF-d_8, 25 °C): δ 134.4 (N2) ppm. N2 was assigned indirectly from N,H-HSQC. N1 and N3 were not detected.
HRMS (ESI): m/z calculated for $C_{24}H_{21}N_3O_2$+ ([M+H]$^+$): 384.1712, observed: 384.1708.
Due to the high signal overlap, it wasn't possible to assign the NMR signals of the minor component.

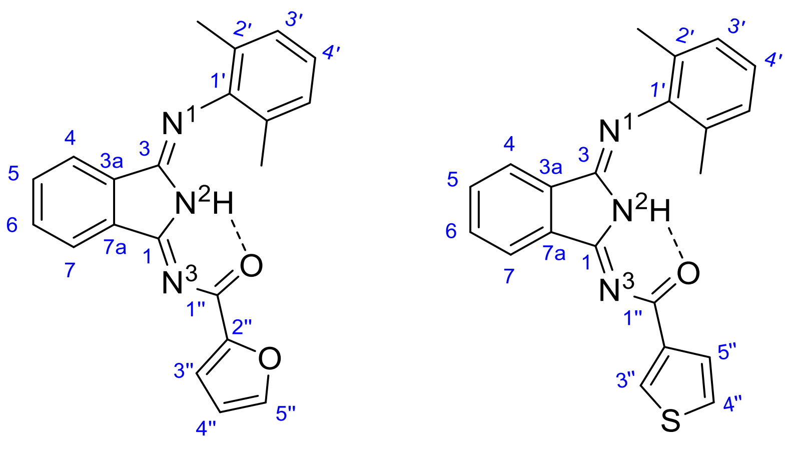

8 **9**

Figure S78. Atom numbering for compounds **8** and **9**. Only Z isomer (majority) is shown.

Compound 8

A The reaction was performed using isoindole **1a** (0.256 g; 1.03 mmol), methyllithium (0.331 mL; 3.1M in diethoxymethane) and 2-furoyl chloride (0.104mL; 1.03 mmol) in diethyl ether (30 mL) to obtain the product as a yellow powder (0.222 g, 60%).

B The reaction was performed using isoindole **1a** (0.452 g; 1.81 mmol), 2-furoyl chloride (0.188 mL; 1.81 mmol) and triethylamine (0.505 mL; 3.62 mmol) in diethyl ether (60 mL) to obtain the product as yellow crystals (0.455 g, 73%), m.p. 162–164°C.

The NMR spectra contained two sets of signals at room temperature in ~2:1 ratio:

Majority signals:**^{1}H NMR** (600 MHz, THF-d_8, 25 °C): δ 10.44 (s, 1H, N2H), 8.15 (dm, 1H, H4, $^3J_{4\text{-}5}$ = 7.5 Hz), 8.08 (dm, 1H, H7, $^3J_{7\text{-}6}$ = 7.5 Hz), 7.81 (td, 1H, H5, $^3J_{5\text{-}4}$ = $^3J_{5\text{-}6}$ = 7.5 Hz, $^4J_{5\text{-}7}$ = 0.8 Hz), 7.77 (td, 1H, H6, $^3J_{6\text{-}5}$ = $^3J_{6\text{-}7}$ = 7.5 Hz, $^4J_{6\text{-}4}$ = 0.7 Hz), 7.76 (dd, 1H, H5'', $^3J_{5''\text{-}4''}$ = 1.4 Hz, $^4J_{5''\text{-}3''}$ = 0.8 Hz), 7.41 (dm, 1H, H3'', $^3J_{3''\text{-}4''}$ = 3.4 Hz), 7.11 (m, 2H, H3'), 6.99 (m, 1H, H4'), 6.59 (dd, 1H, H4'', $^3J_{4''\text{-}5''}$ = 1.4 Hz, $^3J_{4''\text{-}3''}$ = 3.4 Hz), 2.13 (s, 6H, 2'-CH_3) ppm. **^{13}C NMR** (150 MHz, THF-d_8, 25 °C): δ 169.84 (C1''), 160.69 (C1), 152.07 (C2''), 150.23 (C3), 148.08 (C5''), 146.53 (C1'), 136.10 (C7a), 134.86 (C3a), 133.98 (C5), 132.69 (C6), 128.81 (C3'), 127.75 (C2'), 124.64 (C4'), 124.05 (C7), 123.32 (C4), 119.53 (C3''), 112.48 (C4''), 17.93 (2' -CH_3) ppm. **^{15}N NMR** (60 MHz, THF-d_8, 25 °C): δ 265.6 (N1), 134.7 (N2) ppm. N3 was not detected.

Minority signals:**^{1}H NMR** (600 MHz, THF-d_8, 25 °C): δ 11.15 (s, 1H, N2H), 8.08 (dm, 1H, H7, $^3J_{7\text{-}6}$ = 7.5 Hz), 7.80 (m, 1H, H5''), 7.64 (t, 1H, H6, $^3J_{6\text{-}5}$ = $^3J_{6\text{-}7}$ = 7.5 Hz), 7.47 (dm, 1H, H3'', $^3J_{3''\text{-}4''}$ = 3.3 Hz), 7.42 (t, 1H, H5, $^3J_{5\text{-}4}$ = $^3J_{5\text{-}6}$ = 7.5 Hz) 7.11 (m, 2H, H3'), 7.01 (m, 1H, H4'), 6.61–6.64 (m, 2H, H4, H4''), 2.07 (s, 6H, 2'-CH_3) ppm. **^{13}C NMR** (150 MHz, THF-d_8, 25 °C): δ 169.68 (C1''), 160.82 (C1), 153.19 (C3), 152.53 (C2''), 147.71 (C5''), 147.25 (C1'), 137.60 (C7a), 133.90 (C5), 132.81 (C6), 129.60 (C3a), 128.81 (C3'), 127.18 (C2'), 124.84 (C4), 124.18 (C4'), 124.05 (C7), 119.07 (C3''), 112.37 (C4''), 18.03 (2' -CH_3) ppm. **^{15}N NMR** (60 MHz, THF-d_8, 25 °C): δ 281.4 (N1), 141.2 (N2) ppm. N3 was not detected.

HRMS (ESI): m/z calculated for $C_{21}H_{17}N_3O_2$+ ([M+H]+): 344.1399, observed: 344.1395.

Compound 9

A The reaction was performed using isoindole **1a** (0.222 g; 0.89 mmol), methyllithium (0.288 mL; 3.1M in diethoxymethane) and thiophene-3-carbonyl chloride (0.135 g; 0.89 mmol) in diethyl ether (30 mL) to obtain the product as a yellow powder (0.163 g, 51%).

B The reaction was performed using isoindole **1a** (0.471 g; 1.89 mmol), thiophene-3-carbonyl chloride (0.286g; 1.89 mmol) and triethylamine (0.527 mL; 3.78 mmol) in diethyl ether (50 mL) to obtain the product as yellow crystals (0.550 g, 81%), m.p. 148–150°C.

The NMR spectra contained two sets of signals at room temperature in ~2:1 ratio:

Majority signals:

^{1}H NMR (600 MHz, THF-d_8, 25 °C): δ 10.46 (s, 1H, N2H), 8.42 (dd, 1H, H3'', $^4J_{3''\text{-}4''}$ = 3.0 Hz, $^4J_{3''\text{-}5''}$ = 1.1 Hz), 8.13 (dm, 1H, H4, $^3J_{4\text{-}5}$ = 7.6 Hz), 8.08 (dm, 1H, H7, $^3J_{7\text{-}6}$ = 7.6 Hz), 7.81 (td, 1H, H5, $^3J_{5\text{-}4}$ = $^3J_{5\text{-}6}$ = 7.6 Hz, $^4J_{5\text{-}7}$ = 1.0 Hz), 7.74 (td, 1H, H6, $^3J_{6\text{-}5}$ = $^3J_{6\text{-}7}$ = 7.6 Hz, $^4J_{6\text{-}4}$ = 1.0 Hz), 7.59 (dd, 1H, H5'', $^3J_{5''\text{-}4''}$ = 5.1 Hz, $^4J_{5''\text{-}3''}$ = 1.1 Hz), 7.40 (dd, 1H, H4'', $^3J_{4''\text{-}5''}$ = 5.1 Hz, $^4J_{4''\text{-}3''}$ = 3.0 Hz), 7.08 (m, 2H, H3'), 6.96 (m, 1H, H4'), 2.10 (s, 6H, 2'-CH_3) ppm. **^{13}C NMR** (150 MHz, THF-d_8, 25 °C): δ 175.11 (C1''), 160.54 (C1), 150.34 (C3), 146.57 (C1'), 141.91 (C2''), 136.27 (C7a), 134.88 (C3a), 134.64 (C3''), 133.95 (C5), 132.69 (C6), 128.80 (C3'), 128.70 (C5''), 127.76 (C2'), 126.71 (C4''), 124.63 (C4'), 124.03 (C7), 123.33 (C4), 17.93 (2' -CH_3) ppm. **^{15}N NMR** (60 MHz, THF-d_8, 25 °C): δ 265.5 (N1), 134.5 (N2) ppm. N3 was not detected.

Minority signals:

^{1}H NMR (600 MHz, THF-d_8, 25 °C): δ 11.10 (s, 1H, N2H), 8.48 (dd, 1H, H3'', $^4J_{3''\text{-}4''}$ = 3.0 Hz, $^4J_{3''\text{-}5''}$ = 0.9 Hz), 8.08 (dm, 1H, H7), 7.72 (m, 1H, H5''), 7.61 (t, 1H, H6, $^3J_{6\text{-}5}$ = $^3J_{6\text{-}7}$ = 7.4 Hz), 7.44 (dd, 1H, H4'', $^3J_{4''\text{-}5''}$ = 5.1 Hz, $^4J_{4''\text{-}3''}$ = 3.1 Hz), 7.39 (t, 1H, H5, $^3J_{5\text{-}4}$ = $^3J_{5\text{-}6}$ = 7.4 Hz), 7.08 (m, 2H, H3'), 6.98 (m, 1H, H4'), 6.60 (d, 1H, H4), 2.04 (s, 6H, 2' - CH_3) ppm. **^{13}C NMR** (150 MHz, THF-d_8, 25 °C): δ 174.85 (C1''), 160.37 (C1), 153.21 (C3), 147.28 (C1'), 142.38 (C2''), 137.72 (C7a), 134.18 (C3''), 133.83 (C5), 132.79 (C6), 129.70 (C3a), 128.91 (C5''), 127.20 (C2'), 126.46 (C4''), 124.84 (C4), 124.63 (C4'), 124.15 (C7), 18.04 (2' -CH_3) ppm. **^{15}N NMR** (60 MHz, THF-d_8, 25 °C): δ 281.3 (N1) ppm. N2, N3 were not detected.

HRMS (ESI): m/z calculated for $C_{21}H_{17}N_3OS$+ ($[M+H]^+$): 360.1170, observed: 360.1168.

6.5 General procedure for reduction compounds 6 – 9

To a mixture of isoindole and sodium tetraborohydride hexane was added, the suspension was cooled down to -30 °C and 0.250 mL of methanol were added. The solution was left to stir for 2 hours. Then the solution was filtrated rom a highly viscous by-product and the solvent was evaporated under vacuo. The product was dried in vacuo and isolated in the form of a pale-yellow powder. Reactions were carried out under an argon atmosphere using standard Schlenk techniques.

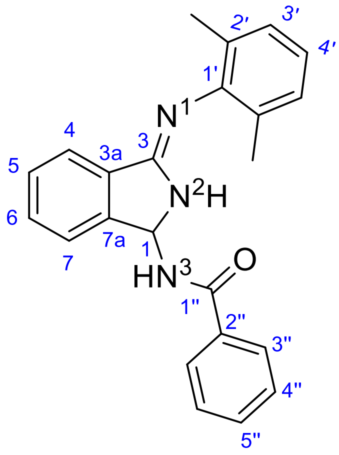

6r

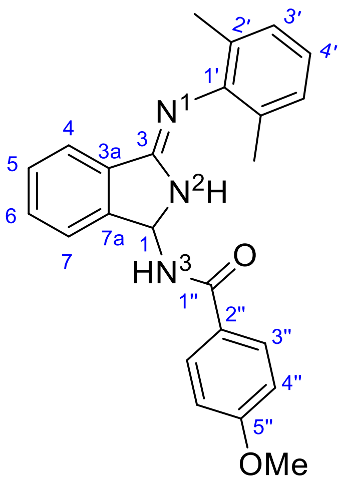

7r

Figure S79. **Atom** numbering for compounds **6r** and **7r**.

Compound 6r

The reaction was performed using isoindole **6** (0.151 g; 0.43 mmol), methanol (0.250 mL) and sodium tetraborohydride (0.016 g; 0.43 mmol) in hexane (20 mL) to obtain the product as a pale yellow powder (0.119 g, 78%) , m.p. 193–195°C. **^{1}H NMR** (600 MHz, THF-d_8, 25 °C): δ 8.19 (d, 1H, N3H, $^3J_{N3H-1}$ = 8.9 Hz), 7.95 (dm, 1H, H4, $^3J_{4-5}$ = 6.9 Hz), 7.84 (m, 2H, H3''), 7.55–7.49 (m, 3H, H5, H6, H7), 7.45 (m, 1H, H5''), 7.37 (m, 2H, H4''), 6.94 (m, 2H, H3'), 6.86 (dd, 1H, H1, $^3J_{1-N3H}$ = 8.9 Hz, $^3J_{1-N2H}$ = 1.6 Hz), 6.78–6.73 (m, 2H, N2H, H4'), 2.11 (s, 6H, 2' **-** CH_3) ppm. **^{13}C NMR** (150 MHz, THF-d_8, 25 °C): δ 167.43 (C1''), 153.77 (C3), 148.91 (C1'), 144.68 (C7a), 136.03 (C3a), 135.36 (C2''), 131.85 (C5''), 131.22 (C6), 129.41 (C5), 128.83 (C2'- broad signal), 128.70 (C4''), 128.25 (C3'- broad signal), 128.15 (C3''), 123.89 (C7), 123.24 (C4), 122.31 (C4'), 64.47 (C1), 18.18 (2' -CH_3) ppm. The signal broadening is caused by the hindered rotation of Dmp. **^{15}N NMR** (60 MHz, THF-d_8, 25 °C): δ 123.5 (N3), 104.3 (N2) ppm. N1 was not detected.
HRMS (ESI): m/z calculated for $C_{23}H_{21}N_3O$+ ([M+H]$^+$): 356.1763, observed: 356.1757.

Compound 7r

The reaction was performed using isoindole **7** (0.133 g; 0.35 mmol), methanol (0.250 mL) and sodium tetraborohydride (0.013 g; 0.35 mmol) in hexane (20 mL) to obtain the product as a pale yellow powder (0.091g, 68%), m.p. 202–204°C. **^{1}H NMR** (500 MHz, THF-d_8, 25 °C): δ 8.06 (d, 1H, N3H, $^3J_{N3H-1}$ = 8.8 Hz), 7.95 (dm, 1H, H4, $^3J_{4-5}$ = 6.5 Hz), 7.81 (m, 2H, H3''), 7.54–7.48 (m, 3H, H5, H6, H7), 6.94 (m, 2H, H3'), 6.90 (m, 2H, H4''), 6.84 (dd, 1H, H1, $^3J_{1-N3H}$ = 8.8 Hz, $^3J_{1-N2H}$ = 1.3 Hz), 6.77–6.71 (m, 2H, N2H, H4'), 3.79 (s, 3H, 5''- O –CH_3), 2.11 (bs, 6H, 2' **-** CH_3) ppm. **^{13}C NMR** (125 MHz, THF-d_8, 25 °C): δ 166.96 (C1''), 163.17 (C5''), 153.79 (C3), 148.91 (C1'), 144.80 (C7a), 136.01 (C3a), 131.18 (C6), 129.92 (C3''), 129.34 (C5), 128.89, 128.78 (C2'), 128.31, 128.19 (C3'), 127.44 (C2''), 123.89 (C7), 123.22 (C4), 122.30 (C4'), 113.85 (C4''), 64.46 (C1), 55.38 (5''- O - CH_3), 18.18 (2' - CH_3) ppm. **^{15}N NMR** (50 MHz, THF-d_8, 25 °C): δ 219.4 (N1), 121.7 (N3), 105.1 (N2) ppm.
HRMS (ESI): m/z calculated for $C_{24}H_{23}N_3O_2$+ ([M+H]$^+$): 386.1869, observed: 386.1862.

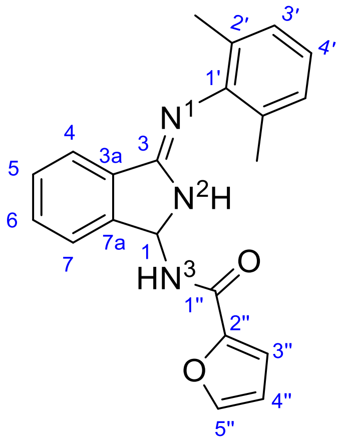

8r

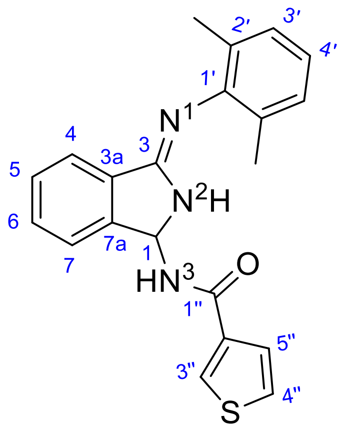

9r

Figure S80. Atom numbering for compounds **8r** and **9r**.

Compound 8r

The reaction was performed using isoindole **8** (0.076 g; 0.22 mmol), methanol (0.125 mL) and sodium tetraborohydride (0.009 g; 0.22 mmol) in hexane (10 mL) to obtain the product as a pale yellow powder (0.055 g, 72%), m.p. 180–182°C. **^{1}H NMR** (500 MHz, THF-d_8, 25 °C): δ 8.23 (d, 1H, N3H, $^{3}J_{N3H-1}$ = 8.8 Hz), 7.95 (m, 1H, H4), 7.50 (dd, 1H, H5'', $^{3}J_{5''-4''}$ = 1.8 Hz, $^{4}J_{5''-3''}$ = 0.7 Hz), 7.53–7.48 (m, 3H, H5, H6, H7), 7.06 (dd, 1H, H3'', $^{3}J_{3''-4''}$ = 3.5 Hz, $^{4}J_{3''-5''}$ = 0.7 Hz), 6.94 (bm, 2H, H3'), 6.80 (dd, 1H, H1, $^{3}J_{1-N3H}$ = 8.8 Hz, $^{3}J_{1-N2H}$ = 1.6 Hz), 6.77–6.73 (m, 2H, N2H, H4'), 6.51 (dd, 1H, H4'', $^{3}J_{4''-5''}$ = 1.8 Hz, $^{3}J_{4''-3''}$ = 3.5 Hz), 2.13 (s, 3H, 2'-CH$_3$), 2.10 (s, 3H, 2'-CH$_3$) ppm. **^{13}C NMR** (125 MHz, THF-d_8, 25 °C): δ 158.55 (C1''), 153.81 (C3), 149.24 (C2''), 148.90 (C1'), 144.99 (C5''), 144.50 (C7a), 136.00 (C3a), 131.19 (C6), 129.39 (C5), 128.85 (C2'- broad signal), 128.26, 128.20 (C3'), 123.86 (C7), 123.23 (C4), 122.31 (C4'), 114.64 (C3''), 112.34 (C4''), 63.64 (C1), 18.18 (2' -CH$_3$) ppm. **^{15}N NMR** (50 MHz, THF-d_8, 25 °C): δ 219.6 (N1), 122.0 (N3), 104.5 (N2) ppm.

HRMS (ESI): m/z calculated for $C_{21}H_{19}N_3O_2$+ ([M+H]$^+$): 346.1556, observed: 346.1550.

Compound 9r

The reaction was performed using isoindole **9** (0.083 g; 0.23 mmol), methanol (0.125 mL) and sodium tetraborohydride (0.009 g; 0.23 mmol) in hexane (10 mL) to obtain the product as a pale yellow powder (0.062 g, 74%), m.p. 194–196°C. **^{1}H NMR** (500 MHz, THF-d_8, 25 °C): δ 8.03 (d, 1H, N3H, $^{3}J_{N3H-1}$ = 8.9 Hz), 7.99 (dd, 1H, H3'', $^{4}J_{3''-4''}$ = 3.0 Hz, $^{4}J_{3''-5''}$ = 1.3 Hz), 7.95 (dm, 1H, H4, $^{3}J_{4-5}$ = 6.8 Hz), 7.54–7.49 (m, 3H, H5, H6, H7), 7.48 (dd, 1H, H5'', $^{3}J_{5''-4''}$ = 5.1 Hz, $^{4}J_{5''-3''}$ = 1.3 Hz), 7.38 (dd, 1H, H4'', $^{3}J_{4''-5''}$ = 5.1 Hz, $^{4}J_{4''-3''}$ = 3.0 Hz), 6.94 (m, 2H, H3'), 6.81 (d, 1H, H1, $^{3}J_{1-N3H}$ = 8.9 Hz), 6.78–6.73 (m, 2H, N2H, H4'), 2.12 (s, 3H, 2'-CH$_3$), 2.10 (s, 3H, 2'-CH$_3$) ppm. **^{13}C NMR** (125 MHz, THF-d_8, 25 °C): δ 162.96 (C1''), 153.72 (C3), 148.87 (C1'), 144.59 (C7a), 138.67 (C2''), 136.01 (C3a), 131.23 (C6), 129.42 (C5), 128.99 (C3''), 128.81 (C2'- broad signal), 128.26 (C3'- broad signal), 127.68 (C5''), 126.36 (C4''), 123.89 (C7), 123.24 (C4), 122.32 (C4'), 64.14 (C1), 18.21 (2'-CH$_3$) ppm. **^{15}N NMR** (50 MHz, THF-d_8, 25 °C): δ 123.6 (N3), 104.9 (N2) ppm. N1 was not detected.

HRMS (ESI): m/z calculated for $C_{21}H_{19}N_3OS$+ ([M+H]$^+$): 362.1327, observed: 362.1324.

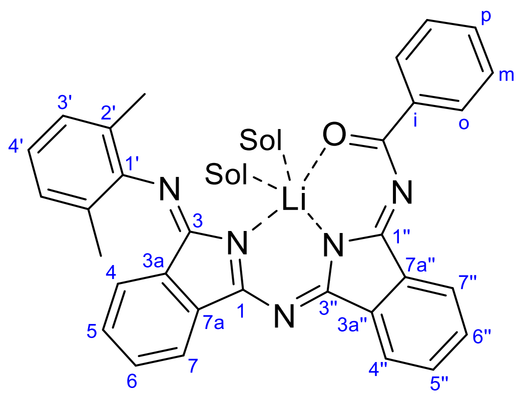

Sol = H_2O or THF

Figure S81. Atom numbering for compound Li**6'**, E-isomer is shown.

Compound Li**6'** in THF-d_8

The NMR spectra contained two sets of signals at room temperature in ~1:1 ratio:

^{1}H NMR (500 MHz, THF-d_8, 25 °C): ***Z*-isomer**: δ 8.47 (m, 2H, *ortho*), 8.05–8.00 (m, 2H, H4, H7), 7.99–7.94 (m, 1H, H4''), 7.92 (dm, 1H, H7'', $^3J_{7''-6''}$ = 7.1 Hz), 7.62–7.59 (m, 2H, H6, H5), 7.58–7.54 (m, 2H, H5'', *para*), 7.54–7.43 (m, 3H, *meta*, H6''), 7.25 (d, 2H, H3', $^3J_{3'-4'}$ = 7.5 Hz), 7.16 (m, 1H, H4'), 2.13 (s, 6H, 2'-C**H**$_3$) ppm. **^{13}C NMR** (125 MHz, THF-d_8, 25 °C): ***Z*-isomer**: δ 179.84 (C=O), 177.71 (C3''), 177.37 (C1), 177.29 (C1''), 164.91 (C3), 149.19 (C1'), 142.54 (C3a), 141.57 (C7a''), 140.02 (C3a''), 138.87 (C7a), 138.43 (C-*ipso*), 133.20 (C-*para*), 132.15 (C5''), 131.56 (C5), 131.41 (C6''), 131.30 (C6''), 131.15 (C-*ortho*), 129.51 (C3'), 128.70 (C-*meta*), 126.78 (C2'), 124.17 (C4'), 123.10 (C7''), 122.88 (C4''), 122.79 (C7), 122.73 (C4), 18.27 (C2'- **C**H$_3$) ppm. **^{7}Li NMR** (194 MHz, THF-d_8, 25 °C): δ 0.49 ppm.

^{1}H NMR (500 MHz, THF-d_8, 25 °C): ***E*-isomer**: δ 8.35 (m, 2H, *ortho*), 7.99- 7.94 (m, 2H, H7, H4''), 7.84 (dm, 1H, H7'', $^3J_{7''-6''}$ = 7.3 Hz), 7.58–7.54 (m, 1H, H5''), 7.54–7.43 (m, 5H, H6'', *para*, H6, *meta*), 7.15 (ddd, 1H, H5, $^3J_{5-6}$ = $^3J_{5-4}$ = 7.6 Hz, $^3J_{5-7}$ = 1.1 Hz), 7.09 (2, 2H, H3', $^3J_{3'-4'}$ = 7.5 Hz), 6.98 (t, 1H, H4', $^3J_{4'-3'}$ = 7.5 Hz), 6.42 (d, 1H, H4, $^3J_{4-5}$ = 7.6 Hz), 2.05 (s, 6H, 2'- C**H**$_3$) ppm. **^{13}C NMR** (125 MHz, THF-d_8, 25 °C): ***E*-isomer**: δ 179.06 (C=O), 178.38 (C3''), 176.98 (C1), 173.79 (C1''), 168.68 (C3), 149.10 (C1'), 143.51 (C3a), 142. 76 (C7a''), 141.87 (C3a''), 137.73 (C-*ipso*), 133.12 (C7a), 132.82 (C-*para*), 131.82 (C6''), 131.75 (C5''), 131.63 (C6), 131.30 (C5), 130.67 (C-*ortho*), 128.82 (C3'), 128.64 (C-*meta*), 126.76 (C2'), 124.42 (C4), 123.92 (C4'), 123.29 (C7''), 123.13 (C4''), 122.65 (C7), 18.69 (C2'- **C**H$_3$) ppm. **^{7}Li NMR** (194 MHz, THF-d_8, 25 °C): δ 1.44 ppm.

HRMS m/z calculated for $C_{31}H_{23}N_5ONa$+ ([M+Na]$^+$): 504.1800, observed: 504.1796.

Compound Li**6'** in THF-d_8 + 80µl H_2O

^{1}H NMR (500 MHz, THF-d_8, 25 °C): δ 8.40 (m, 2H, *ortho*), 8.01 (dm, 1H, H7'', $^3J_{7''-6''}$ = 7.1 Hz), 7.98 (dm, $^3J_{7-6}$ = 7.2 Hz, H7), 7.93 (m, H4''), 7.63 (dd, $^3J_{6''-7''}$ = $^3J_{6''-5''}$ = 7.1 Hz, H6''), 7.55–7.61(m, 2H, H5'', *para*), 7.46–7.54 (m, 3H, H6, *meta*), 7.16 (dd, $^3J_{5-6}$ = $^3J_{5-4}$ = 7.5 Hz, H5), 7.12 (m, 2H, H3'), 7.04 (m, H4'), 6.38 (dm, $^3J_{4-5}$ = 7.5 Hz, H4), 2.06 (s, 6H, 2' -C**H$_3$**) ppm.**^{13}C NMR** (125 MHz, THF-d_8, 25 °C): δ 180.11 (C=O), 178.13 (C1''), 177.12 (C1), 174.82 (C3''), 169.90 (C3), 147.91 (C1'), 143.10 (C7a), 142.26 (C7a''), 140.58 (C3a''), 137.51 (C-*ipso*), 132.99 (C-*para*), 132.89 (C3a), 131.99 (C6), 131.91 (C6''), 131.43 (C5''), 131.21 (C5), 130.67 (C-*ortho*), 128.63 (C3'), 128.57 (C-*meta*), 127.22 (C2'), 124.34 (C4'),

124.20 (C4), 122.75 and 122.72 (C4'' and C7''), 122.66 (C7), 18.05 (C2'- **C**H3) ppm. **^{7}Li NMR** (194 MHz, THF-d_8, 25 °C): δ 1.05 ppm.

Compound Li**6'** in CD_2Cl_2

^{1}H NMR (500 MHz, CD_2Cl_2, 25 °C): δ 8.30 (d, 2H, *ortho,* $^3J_{o\text{-}m}$ = 7.3 Hz), 8.10 (d, 1H, H7, $^3J_{7\text{-}6}$ = 7.4 Hz), 8.04 (d, 1H, H4'', $^3J_{4''\text{-}5''}$ = 7.2 Hz), 7.91 (d, 1H, H7'', $^3J_{7''\text{-}6''}$ = 7.2 Hz), 7.60 (ddd, 1H, H5'', $^3J_{5''\text{-}6''}$ = $^3J_{5''\text{-}4''}$ = 6.5 Hz, $^3J_{5''\text{-}7''}$ = 0.8 Hz), 7.24–7.18 (m, 2H, H5, *para*), 7.07–7.00 (m, 5H, *meta*, H3', H4'), 6.40 (d, H4, $^3J_{4\text{-}5}$ = 7.5 Hz), 1.84 (s, 6H, 2'-C**H3**) ppm.**^{13}C NMR** (125 MHz, CD_2Cl_2, 25 °C): δ 180.31 (C=O), 179.42 (C3''), 177.95 (C1''), 177.91 (C1), 169.76 (C3), 146.68 (C1'), 142.61 (C3a), 141.65 (C3a''), 141.19 (C7a''), 137.82 (C-*ipso*), 132.69 (C-*para*), 132.50 (C7a), 132.16 (C5), 132.07 (C5''), 131.81 (C6), 131.76 (C6''), 130.78 (C-*ortho*), 128.47 (C3'), 128.05 (C-*meta*), 127.41 (C2'), 124.75 (C4), 124.64 (C4'), 123.00 (C4''), 122.95 (C7''), 122.74 (C7), 18.1 (C2'- **C**H3) ppm. **^{7}Li NMR** (194 MHz, CD_2Cl_2, 25 °C): δ 2.49 ppm.

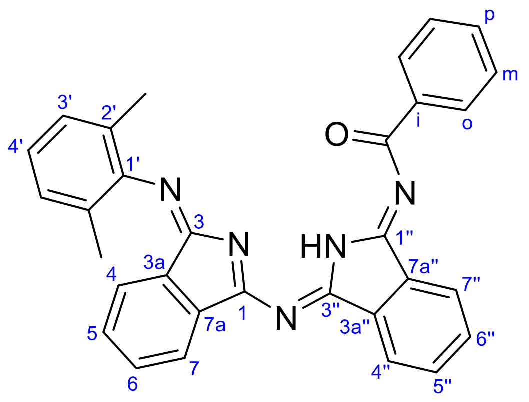

Figure S82. Atom numbering for compound **6'**.

Compound 6' in THF-d_8

The NMR spectra contained two sets of signals at room temperature in ~5:1 ratio:
Majority signals:
^{1}H NMR (500 MHz, THF-d_8, 25 °C): ***Z*-isomer:** δ 11.77 (1H, NH), 8.11 (d, 2H, *ortho,* $^3J_{o\text{-}m}$ = 7.5 Hz), 8.10–8.05 (m, 2H, H4, H7''), 8.01 (d, 1H, H4'', $^3J_{7''\text{-}6''}$ = 7.5 Hz), 7.98 (d, 1H, H7, $^3J_{7\text{-}6}$ = 7 Hz), 7.76–7.65 (m, 4H, H6'', H5'', H6, H5), 7.57 (t, 1H, *para,* $^3J_{p\text{-}m}$ = 6.6 Hz), 7.48 (m, 2H, *meta*), 7.09 (d, 2H, H3', $^3J_{3'\text{-}4'}$ = 7 Hz), 6.99 (t, 1H, H4', $^3J_{4'\text{-}3'}$ = 7 Hz), 2.09 (s, 6H, 2'-C**H3**) ppm. **^{13}C NMR** (125 MHz, THF-d_8, 25 °C): ***Z*-isomer:** δ 178.11 (C=O), 173.37 (C1), 167.72 (C1''), 159.57 (C3''), 158.54 (C3), 147.78 (C1'), 139.21 (C3a''), 138.60 (C3a), 137.51 (C7a''), 136.36 (C7a), 135.55 (C-*ipso*), 133.60 (C6''), 133.54 (C5''),133.52 (C-*para*), 132.77 (C5), 132.03 (C6), 130.75 (C-*ortho*), 128.81 (C-*meta*), 128.57 (C3'), 127.07 (C2'), 124.31 (C4'), 124.18 (C7''), 123.85 (C4''), 123.34 (C4), 122.96 (C7), 18.57 (C2'- **C**H3) ppm.

Minority signals:
^{1}H NMR (500 MHz, THF-d_8, 25 °C): ***E*-isomer:** δ 11.77 (1H, NH), 8.17 (d, 2H, *ortho,* $^3J_{o\text{-}m}$ = 7.5 Hz), 8.10–8.05 (m, 1H, H7''), 8.01 (m, 1H, H4''), 7.98 (m, 1H, H7), 7.76–7.65 (m, 2H, H6'', H5''), 7.58 (m, 1H, H6), 7.57 (m, 1H, *para*), 7.48 (m, 2H *meta*), 7.34 (ddd, 1H, H5 $^3J_{5\text{-}6}$ = $^3J_{5\text{-}4}$ = 7.4 Hz), 7.11 (m, 2H, H3'), 7.01 (m, 1H, H4'), 6.58 (d, 1H, H4, $^3J_{4\text{-}5}$ = 7.5 Hz), 2.03 (s, 6H, 2'-C**H3**) ppm. **^{13}C NMR** (125 MHz, THF-d_8, 25 °C): ***E*-isomer:** δ 178.96 (C=O), 172.87 (C1''), 169.83 (C1), 161.69 (C3''), 157.88 (C3), 147.78 (C1'), 138.99 (C3a), 137.97 (C7a), 137.51 (C3a''), 136.36 (C7a''), 135.56 (C-*ipso*), 133.50 (C5), 133.21 (C6''), 132.96 (C5''), 132.76 (C-*para*), 132.72 (C6), 130.30 (C-*ortho*), 129.07 (C-*meta*), 128.81 (C3'), 126.42 (C2'), 125.14 (C4), 124.44 (C7''), 124.42 (C4'), 123.53 (C7), 123.39 (C4''), 18.04 (C2'-Me) ppm.

HRMS m/z calculated for $C_{31}H_{24}N_5O+$ ($[M+H]^+$): 482.1980, observed: 482.1977.

Compound 6' in CD_2Cl_2

The NMR spectra contained two sets of signals at room temperature in ~2:1 ratio:
Majority signals:
^{1}H NMR (500 MHz, CD_2Cl_2, 25 °C): ***Z*-isomer:** δ 11.47 (1H, NH), 8.10 (d, 1H, H4, $^3J_{o\text{-}m}$ = 7.4 Hz), 8.07–8.01 (m, 4H, H7, H7'', *ortho*), 7.97 (d, 1H, H4'', $^3J_{4''\text{-}5''}$ = 7.2 Hz), 7.75–7.65 (m, 4H, H6, H5, H6'', H5''), 7.61–7.56 (m, 1H, *para*), 7.53–7.46 (m, 2H, *meta*), 7.07 (m, 2H, H3'), 7.05-7.02 (m, 1H, H4'), 2.05 (s, 6H, 2'-C**H**$_3$) ppm. **^{13}C NMR** (125 MHz, CD_2Cl_2, 25 °C): ***Z-isomer:*** δ 178.86 (C=O), 171.51 (C1), 170.33 (C1''), 160.18 (C3''), 156.53 (C3), 146.86 (C1'), 138.11 (C3a), 137.99 (C3a''), 136.93 (C7a), 136.16 (C7a''), 134.64 (C-*ipso*), 133.65 (C-*para*), 133.51 (C6''), 133.17 (C5''), 133.05 (C5), 132.19 (C6), 130.52 (C-*ortho*), 128.83 (C-*meta*), 128.59 (C3'), 127.24 (C2'), 124.60 (C4'), 124.10 (C4''), 123.63 (C7''), 123.33 (C4), 123.21 (C7), 18.61 (C2'- **C**H$_3$) ppm.

Minority signals:
^{1}H NMR (500 MHz, CD_2Cl_2, 25 °C): ***E*-isomer:** δ 11.47 (1H, NH), 8.22 (m, 2H, *ortho*), 8.07–8.01 (m, H, H7'', H7, H4''), 7.75–7.65 (m, 1H, H5''), 7.61–7.56 (m, 2H, *para,* H6), 7.53–7.46 (m, 3H, H6'', *meta*), 7.07 (m, 2H, H3'), 7.05-7.02 (m, 1H, H4'),7.32 (ddd, 1H, H5 $^3J_{5\text{-}6}$ = $^3J_{5\text{-}4}$ = 7.4 Hz), 7.13 (m, 2H, H3'), 7.05-7.02 (m, 1H, H4'), 6.53 (d, 1H, H4, $^3J_{4\text{-}5}$ = 7.6 Hz), 2.04 (s, 6H, 2'-C**H**$_3$) ppm. **^{13}C NMR** (125 MHz, CD_2Cl_2, 25 °C): ***E*-isomer:** δ 180.04 (C=O), 172.01 (C1''), 171.05 (C1), 161.69 (C3''), 158.86 (C3), 147.36 (C1'), 138.63 (C3a''), 138.57 (C3a), 137.99 (C7a), 135.90 (C7a''), 135.00 (C-*ipso*), 134.05 (C-*para*), 133.65 (C5), 133.35 (C6''), 133.05 (C5''), 132.57 (C6), 130.46 (C-*ortho*), 129.11 (C-*meta*), 128.79 (C3'), 126.49 (C2'), 125.29 (C4), 124.54 (C4'), 124.41 (C4''), 123.57 (C7''), 123.30 (C7), 18.43 (C2'- **C**H$_3$) ppm.

7 Crystal data and structure refinements of prepared compounds

Table S11. Crystal data and structure refinement for Li**1a**.

Crystal data	
Chemical formula	$C_{50}H_{68}Li_2N_6O_3Si_2$
M_r	871.16
Crystal system, space group	Monoclinic, *C*2/*c*
Temperature (K)	150
a, *b*, *c* (Å)	19.3302(3), 10.7540(2), 24.7234(5)
β (°)	100.382(4)
V (Å^3)	5055.28(17)
Z	4
Radiation type	Mo*K*α
μ (mm^{-1})	0.12
Crystal size (mm)	0.48 × 0.46 × 0.35
**Data collection**	
Diffractometer	Bruker Nonius KappaCCD area detector
Absorption correction	Integration, Gaussian integration (Coppens, 1970)
T_{min}, T_{max}	0.960, 0.974
No. of measured, independent and	33545, 5754, 4908
R_{int}	0.072
(sin θ/λ)$_{max}$ (Å^{-1})	0.650
**Refinement**	
$R[F^2 > 2\sigma(F^2)]$, $wR(F^2)$, S	0.064, 0.181, 1.11
No. of reflections	5754
No. of parameters	266
No. of restraints	268
H-atom treatment	H-atom parameters constrained
	$w = 1/[\sigma^2(F_o^2) + (0.0758P)^2 + 10.418P]$
$\Delta\rho_{max}$, $\Delta\rho_{min}$ (e Å^{-3})	0.62, −0.43

Computer programs: *COLLECT* (Hooft, 1998) and *DENZO* (Otwinowski & Minor, 1997), *COLLECT* and *DENZO*, *SIR92* (Altomare *et al.*, 1994), *SHELXL2013* (Sheldrick, 2013), *PLATON* (Spek, 2003), *SHELXL97* (Sheldrick, 2008).

Table S12. Crystal data and structure refinement for Li**1b**.

Crystal data	
Chemical formula	$C_{62}H_{92}Li_2N_6O_4Si_2$
M_r	1055.47
Crystal system, space group	Monoclinic, $P2_1/n$
Temperature (K)	150
a, *b*, *c* (Å)	13.0351(16), 16.7375(15), 14.264(3)
β (°)	97.930(9)
V (Å^3)	3082.3(7)
Z	2
Radiation type	Mo*K*α
μ (mm^{-1})	0.11
Crystal size (mm)	0.59 × 0.46 × 0.41
**Data collection**	
Diffractometer	Bruker D8 - Venture
Absorption correction	Multi-scan *SADABS2016*/2 - Bruker AXS area detector scaling and absorption correction
T_{min}, T_{max}	0.632, 0.746
No. of measured, independent and observed [$I > 2\sigma(I)$] reflections	74635, 6033, 5122
R_{int}	0.074
$(\sin \theta/\lambda)_{max}$ (Å^{-1})	0.617
**Refinement**	
$R[F^2 > 2\sigma(F^2)]$, $wR(F^2)$, S	0.077, 0.198, 1.07
No. of reflections	6033
No. of parameters	430
No. of restraints	125
H-atom treatment	H-atom parameters constrained
$\Delta\rho_{max}$, $\Delta\rho_{min}$ (e Å^{-3})	0.78, −0.78

Computer programs: Apex4 v2022.1-1 (Bruker, 2022), *SAINT* V8.40B (Bruker AXS LLC, 2019), *SHELXT* 2014/5 (Sheldrick, 2014), *SHELXL2018*/3 (Sheldrick, 2018), Bruker *SHELXTL*.

Table S13. Crystal data and structure refinement for Li**3b**

Crystal data	
Chemical formula	$C_{39}H_{50}LiN_5O_2Si$
M_r	655.87
Crystal system, space group	Monoclinic, $P2_1/n$
Temperature (K)	150
a, *b*, *c* (Å)	8.078(13), 29.43(5), 16.27(2)
β (°)	100.83(4)
V (Å^3)	3799(10)
Z	4
Radiation type	Mo*K*α
μ (mm^{-1})	0.10
Crystal size (mm)	0.22 × 0.16 × 0.13
**Data collection**	
Diffractometer	Bruker D8 - Venture
Absorption correction	Multi-scan<br>*SADABS2016*/2 - Bruker AXS area detector scaling and absorption correction
T_{min}, T_{max}	0.708, 0.745
No. of measured, independent and observed [$I > 2\sigma(I)$] reflections	39982, 7454, 5687
R_{int}	0.052
$(\sin \theta/\lambda)_{max}$ (Å^{-1})	0.621
**Refinement**	
$R[F^2 > 2\sigma(F^2)]$, $wR(F^2)$, S	0.060, 0.166, 1.02
No. of reflections	7454
No. of parameters	425
No. of restraints	12
H-atom treatment	H-atom parameters constrained
$(\Delta/\sigma)_{max}$	0.556
$\Delta\rho_{max}$, $\Delta\rho_{min}$ (e Å^{-3})	0.06, −0.42

Computer programs: Bruker Instrument Service vV6.2.3, *APEX3* v2016.5-0 (Bruker AXS), *SAINT* V8.37A (Bruker AXS Inc., 2015), XT, VERSION 2014/5, *SHELXL2014*/7 (Sheldrick, 2014), *PLATON* (Spek, 2009).

Table S14. Crystal data and structure refinement for Li**3b'**.

Crystal data	
Chemical formula	$C_{62}H_{70}Li_4N_{10}O_2Si_2 \cdot 3(CHCl_3)$
M_r	1429.32
Crystal system, space group	Triclinic, *P*1
Temperature (K)	150
a, *b*, *c* (Å)	11.580(1), 18.184(2), 18.2930(12)
α, β, γ (°)	74.962(7), 78.718(5), 86.917(10)
V (Å^3)	3648.2(6)
Z	2
Radiation type	Mo*K*α
μ (mm^{-1})	0.43
Crystal size (mm)	0.59 × 0.50 × 0.44
**Data collection**	
Diffractometer	Bruker Nonius KappaCCD area detector
Absorption correction	Integration<br>Gaussian integration (Coppens, 1970)
T_{min}, T_{max}	0.825, 0.901
No. of measured, independent and observed [$I > 2\sigma(I)$] reflections	57082, 16116, 11301
R_{int}	0.093
$(\sin \theta/\lambda)_{max}$ (Å^{-1})	0.645
**Refinement**	
$R[F^2 > 2\sigma(F^2)]$, $wR(F^2)$, S	0.062, 0.202, 1.14
No. of reflections	16116
No. of parameters	729
H-atom treatment	H atoms treated by a mixture of independent and constrained refinement
$\Delta\rho_{max}$, $\Delta\rho_{min}$ (e Å^{-3})	0.63, −0.41

Computer programs: *COLLECT* (Hooft, 1998) and *DENZO* (Otwinowski & Minor, 1997), *COLLECT* and *DENZO*, *SIR92* (Altomare *et al.*, 1994), *SHELXL2013* (Sheldrick, 2013), *PLATON* (Spek, 2003), *SHELXL97* (Sheldrick, 2008).

Table S15. Crystal data and structure refinement for Li**3b''**.

Crystal data	
Chemical formula	$C_{30.5}H_{34.75}LiN_4O_3$
M_r	4098.49
Crystal system, space group	Orthorhombic, *Ama*2
Temperature (K)	150
a, *b*, *c* (Å)	17.2649(10), 20.1075(11), 16.2759(11)
V (Å^3)	5650.2(6)
Z	8
Radiation type	Mo*K*α
μ (mm^{-1})	0.08
Crystal size (mm)	0.29 x 0.24 x 0.23
Data collection	
Diffractometer	Bruker D8 - Venture
Absorption correction	–
No. of measured, independent and observed [$I > 2\sigma(I)$] reflections	47352, 5744, 5461
R_{int}	0.0806
$(\sin \theta/\lambda)_{max}$ (Å^{-1})	0.626
Refinement	
$R[F^2 > 2\sigma(F^2)]$, $wR(F^2)$, S	0.079, 0.232, 0.93
No. of reflections	5744
No. of parameters	411
No. of restraints	681
H-atom treatment	H-atom parameters constrained
$\Delta\rho_{max}$, $\Delta\rho_{min}$ (e Å^{-3})	0.31, −0.34
Absolute structure	Refined as an inversion twin.
Absolute structure parameter	1 (4)

Computer programs: *SHELXL2013* (Sheldrick, 2013).

Table S16. Crystal data and structure refinement for Li**2a**

Crystal data	
Chemical formula	$C_{68}H_{62}Li_2N_{10}O \cdot 0.5(C_6H_{14}) \cdot C_4H_{10}O$
M_r	1166.36
Crystal system, space group	Triclinic, *P*-1
Temperature (K)	150
a, *b*, *c* (Å)	12.9613(12), 15.9307(14), 16.3777(16)
α, β, γ (°)	88.346(3), 75.740(3), 80.200(3)
V (Å^3)	3229.4(5)
Z	2
Radiation type	Mo*K*α
μ (mm^{-1})	0.07
Crystal size (mm)	0.42 × 0.17 × 0.11
**Data collection**	
Diffractometer	Bruker D8 - Venture
Absorption correction	Multi-scan<br>*SADABS2016*/2 - Bruker AXS area detector scaling and absorption correction
T_{min}, T_{max}	0.645, 0.746
No. of measured, independent and observed [$I > 2\sigma(I)$] reflections	39118, 10616, 7856
R_{int}	0.074
$(\sin \theta/\lambda)_{max}$ (Å^{-1})	0.595
**Refinement**	
$R[F^2 > 2\sigma(F^2)]$, $wR(F^2)$, S	0.117, 0.291, 1.13
No. of reflections	10616
No. of parameters	833
No. of restraints	853
H-atom treatment	H-atom parameters constrained<br>$w = 1/[\sigma^2(F_o^2) + (0.1091P)^2 + 13.4457P]$ where $P = (F_o^2 + 2F_c^2)/3$
$\Delta\rho_{max}$, $\Delta\rho_{min}$ (e Å^{-3})	0.54, −0.72

Computer programs: *COLLECT* (Hooft, 1998) and *DENZO* (Otwinowski & Minor, 1997), *COLLECT* and *DENZO*, *SIR92* (Altomare *et al.*, 1994), *SHELXL2013* (Sheldrick, 2013), *PLATON* (Spek, 2003), *SHELXL97* (Sheldrick, 2008).

Table S17. Crystal data and structure refinement for **2b**

Crystal data	
Chemical formula	$C_{46}H_{57}N_5$
M_r	679.96
Crystal system, space group	Monoclinic, $P2_1/n$
Temperature (K)	150
a, *b*, *c* (Å)	8.3012(4), 22.0347(10), 21.6890(9)
β (°)	95.919(2)
V (Å^3)	3946.1(3)
Z	4
Radiation type	Mo*K*α
μ (mm^{-1})	0.07
Crystal size (mm)	0.59 × 0.57 × 0.55
**Data collection**	
Diffractometer	Bruker D8 - Venture
Absorption correction	Multi-scan *SADABS2016*/2 - Bruker AXS area detector scaling and absorption correction
T_{min}, T_{max}	0.717, 0.746
No. of measured, independent and observed [$I > 2\sigma(I)$] reflections	111690, 9084, 7251
R_{int}	0.058
$(\sin \theta/\lambda)_{max}$ (Å^{-1})	0.651
**Refinement**	
$R[F^2 > 2\sigma(F^2)]$, $wR(F^2)$, S	0.051, 0.128, 1.05
No. of reflections	9084
No. of parameters	474
H-atom treatment	H atoms treated by a mixture of independent and constrained refinement
$\Delta\rho_{max}$, $\Delta\rho_{min}$ (e Å^{-3})	0.49, −0.28

Computer programs: Bruker Instrument Service vV6.2.3, *APEX3* v2018.1-0 (Bruker AXS), *SAINT* V8.38A (Bruker AXS Inc., 2016), *SHELXT* 2014/5 (Sheldrick, 2014), *SHELXL2018*/3 (Sheldrick, 2018), Bruker *SHELXTL*.

Table S18. Crystal data and structure refinement for **1a**.

Crystal data	
Chemical formula	$C_{16}H_{16}N_3O_{0.50}$
M_r	258.32
Crystal system, space group	Monoclinic, *C*2/*c*
Temperature (K)	150
a, *b*, *c* (Å)	24.489(9), 8.624(3), 13.616(5)
β (°)	109.812(9)
V (Å^3)	2705.4(17)
Z	8
Radiation type	Mo*K*α
μ (mm^{-1})	0.08
Crystal size (mm)	0.43 × 0.34 × 0.20
**Data collection**	
Diffractometer	Bruker D8 - Venture
Absorption correction	Multi-scan *SADABS2016*/2 - Bruker AXS area detector scaling and absorption correction
T_{min}, T_{max}	0.670, 0.746
No. of measured, independent and observed [$I > 2\sigma(I)$] reflections	10315, 3101, 2538
R_{int}	0.030
$(\sin \theta/\lambda)_{max}$ (Å^{-1})	0.652
**Refinement**	
$R[F^2 > 2\sigma(F^2)]$, $wR(F^2)$, S	0.040, 0.101, 1.05
No. of reflections	3101
No. of parameters	186
No. of restraints	2
H-atom treatment	H atoms treated by a mixture of independent and constrained refinement
$\Delta\rho_{max}$, $\Delta\rho_{min}$ (e Å^{-3})	0.26, −0.22

Computer programs: Bruker Instrument Service vV6.2.3, APEX3 v2018.1-0 (Bruker AXS), SAINT V8.38A (Bruker AXS Inc., 2016), SHELXT 2014/5 (Sheldrick, 2014), SHELXL2018/3 (Sheldrick, 2018), Bruker SHELXTL.

Table S19. Crystal data and structure refinement for **1b**.

Crystal data	
Chemical formula	$C_{20}H_{23}N_3$
M_r	305.41
Crystal system, space group	Triclinic, *P*-1
Temperature (K)	150
a, *b*, *c* (Å)	8.8034(8), 9.7521(10), 11.1081(15)
α, β, γ (°)	87.990(7), 70.502(6), 79.753(6)
V (Å^3)	884.32(17)
Z	2
Radiation type	Mo*K*α
μ (mm^{-1})	0.07
Crystal size (mm)	0.59 × 0.36 × 0.26
Data collection	
Diffractometer	Bruker D8 - Venture
Absorption correction	Multi-scan *SADABS2016*/2 - Bruker AXS area detector scaling and absorption correction
T_{min}, T_{max}	0.685, 0.746
No. of measured, independent and observed [$I > 2\sigma(I)$] reflections	27082, 4035, 3639
R_{int}	0.026
$(\sin \theta/\lambda)_{max}$ (Å^{-1})	0.650
Refinement	
$R[F^2 > 2\sigma(F^2)]$, $wR(F^2)$, S	0.044, 0.115, 1.01
No. of reflections	4035
No. of parameters	212
H-atom treatment	H-atom parameters constrained
$\Delta\rho_{max}$, $\Delta\rho_{min}$ (e Å^{-3})	0.43, −0.33

Computer programs: Bruker Instrument Service vV6.2.3, *APEX3* v2018.1-0 (Bruker AXS), *SAINT* V8.38A (Bruker AXS Inc., 2016), *SHELXT* 2014/5 (Sheldrick, 2014), *SHELXL2018*/3 (Sheldrick, 2018), Bruker *SHELXTL*.

Table S20. Crystal data and structure refinement for **1c**.

Crystal data	
Chemical formula	$C_{14}H_{11}N_3$
M_r	221.26
Crystal system, space group	Tetragonal, $P4_32_12$
Temperature (K)	150
a, *c* (Å)	9.4560(7), 25.8130(17)
V (Å^3)	2308.1(4)
Z	8
Radiation type	Mo*K*α
μ (mm^{-1})	0.08
Crystal size (mm)	0.48 × 0.29 × 0.25
Data collection	
Diffractometer	Bruker Nonius KappaCCD area detector
Absorption correction	Integration Gaussian integration (Coppens, 1970)
T_{min}, T_{max}	0.980, 0.988
No. of measured, independent and observed [$I > 2\sigma(I)$] reflections	11145, 2530, 2140
R_{int}	0.041
$(\sin \theta/\lambda)_{max}$ (Å^{-1})	0.649
Refinement	
$R[F^2 > 2\sigma(F^2)]$, $wR(F^2)$, S	0.044, 0.107, 1.06
No. of reflections	2530
No. of parameters	162
H-atom treatment	H atoms treated by a mixture of independent and constrained refinement
$\Delta\rho_{max}$, $\Delta\rho_{min}$ (e Å^{-3})	0.16−0.21
Absolute structure	Refined as a perfect inversion twin.
Absolute structure parameter	0.5

Computer programs: *COLLECT* (Hooft, 1998) and *DENZO* (Otwinowski & Minor, 1997), *COLLECT* and *DENZO*, *SIR92* (Altomare *et al.*, 1994), *SHELXL2013* (Sheldrick, 2013), *PLATON* (Spek, 2003).

Table S21. Crystal data and structure refinement for **5aa**.

Crystal data	
Chemical formula	$C_{33}H_{33}N_5$
M_r	499.64
Crystal system, space group	Monoclinic, $P2_1/n$
Temperature (K)	150
a, *b*, *c* (Å)	13.3167(5), 8.0497(3), 25.9533(9)
β (°)	100.536(1)
V (Å^3)	2735.17(17)
Z	4
Radiation type	Mo*K*α
μ (mm^{-1})	0.07
Crystal size (mm)	0.54 × 0.35 × 0.08
**Data collection**	
Diffractometer	Bruker D8 - Venture
Absorption correction	Multi-scan *SADABS2016*/2 - Bruker AXS area detector scaling and absorption correction
T_{min}, T_{max}	0.687, 0.746
No. of measured, independent and observed [$I > 2\sigma(I)$] reflections	64566, 6308, 4257
R_{int}	0.134
$(\sin \theta/\lambda)_{max}$ (Å^{-1})	0.651
**Refinement**	
$R[F^2 > 2\sigma(F^2)]$, $wR(F^2)$, S	0.053, 0.114, 1.04
No. of reflections	6308
No. of parameters	357
H-atom treatment	H atoms treated by a mixture of independent and constrained refinement
$\Delta\rho_{max}$, $\Delta\rho_{min}$ (e Å^{-3})	0.19, −0.23

Computer programs: Bruker Instrument Service vV6.2.3, *APEX3* v2018.1-0 (Bruker AXS), *SAINT* V8.38A (Bruker AXS Inc., 2016), *SHELXT* 2014/5 (Sheldrick, 2014), *SHELXL2018*/3 (Sheldrick, 2018), Bruker *SHELXTL*.

D—H···*A*	*D*—H	H···*A*	*D*···*A*	*D*—H···*A*
N5—H5···N2	0.90(2)	1.92(2)	2.666(2)	139(2)

Table S22. Crystal data and structure refinement for **5aa'**.

Crystal data	
Chemical formula	$C_{33}H_{33}N_5$
M_r	499.64
Crystal system, space group	Monoclinic, $P2_1/n$
Temperature (K)	150
a, *b*, *c* (Å)	13.344(2), 8.0548(9), 25.986(5)
β (°)	100.538(8)
V (Å^3)	2745.9(7)
Z	4
Radiation type	Mo*K*α
μ (mm^{-1})	0.07
Crystal size (mm)	0.26 × 0.24 × 0.19
**Data collection**	
Diffractometer	Bruker D8 - Venture
Absorption correction	Multi-scan *SADABS2016*/2 - Bruker AXS area detector scaling and absorption correction
T_{min}, T_{max}	0.575, 0.746
No. of measured, independent and observed [$I > 2σ(I)$] reflections	72415, 8474, 5898
R_{int}	0.089
$(\sin θ/λ)_{max}$ (Å^{-1})	0.730
**Refinement**	
$R[F^2 > 2σ(F^2)]$, $wR(F^2)$, S	0.081, 0.177, 1.05
No. of reflections	8474
No. of parameters	357
H-atom treatment	H atoms treated by a mixture of independent and constrained refinement
$Δρ_{max}$, $Δρ_{min}$ (e Å^{-3})	0.40, −0.35

Computer programs: Bruker Instrument Service vV6.2.3, *APEX3* v2018.1-0 (Bruker AXS), *SAINT* V8.38A (Bruker AXS Inc., 2016), *SHELXT* 2014/5 (Sheldrick, 2014), *SHELXL2018*/3 (Sheldrick, 2018), Bruker *SHELXTL*.

D—H···*A*	*D*—H	H···*A*	*D*···*A*	*D*—H···*A*
N5—H5···N2	0.88(2)	1.96(2)	2.673(2)	137(2)

Table S23. Crystal data and structure refinement for **5ab**.

Crystal data	
Chemical formula	$C_{41}H_{49}N_5$
M_r	611.85
Crystal system, space group	Monoclinic, $P2_1/n$
Temperature (K)	150
a, *b*, *c* (Å)	11.7970(7), 22.6581(14), 14.5460(7)
β (°)	112.585(2)
V (Å^3)	3589.9(4)
Z	4
Radiation type	Mo*K*α
μ (mm^{-1})	0.07
Crystal size (mm)	0.59 × 0.56 × 0.26
**Data collection**	
Diffractometer	Bruker D8 - Venture
Absorption correction	Multi-scan<br>*SADABS2016*/2 - Bruker AXS area detector scaling and absorption correction
T_{min}, T_{max}	0.686, 0.746
No. of measured, independent and observed [$I > 2\sigma(I)$] reflections	158518, 8272, 6777
R_{int}	0.068
(sin θ/λ)$_{max}$ (Å^{-1})	0.651
**Refinement**	
$R[F^2 > 2\sigma(F^2)]$, $wR(F^2)$, S	0.049, 0.133, 1.13
No. of reflections	8272
No. of parameters	434
H-atom treatment	H atoms treated by a mixture of independent and constrained refinement
$\Delta\rho_{max}$, $\Delta\rho_{min}$ (e Å^{-3})	0.42, −0.39

Computer programs: Bruker Instrument Service vV6.2.3, *APEX3* v2018.1-0 (Bruker AXS), *SAINT* V8.38A (Bruker AXS Inc., 2016), *SHELXT* 2014/5 (Sheldrick, 2014), *SHELXL2018*/3 (Sheldrick, 2018), Bruker *SHELXTL*.

D—H···*A*	*D*—H	H···*A*	*D*···*A*	*D*—H···*A*
N5—H5···N2	0.95 (3)	1.81(3)	2.612(1)	141(2)

Table S24. Crystal data and structure refinement for **5bb**.

Crystal data	
Chemical formula	$C_{52}H_{65}N_5$
M_r	760.09
Crystal system, space group	Triclinic, *P*-1
Temperature (K)	150
a, *b*, *c* (Å)	10.4798(4), 10.7451(4), 22.0979(8)
α, β, γ (°)	80.4101(16), 78.0026(16), 70.3112(16)
V (Å^3)	2279.08(15)
Z	2
Radiation type	Mo*K*α
μ (mm^{-1})	0.07
Crystal size (mm)	0.22 × 0.21 × 0.20
**Data collection**	
Diffractometer	Bruker D8 - Venture
Absorption correction	Multi-scan *SADABS2016*/2 - Bruker AXS area detector scaling and absorption correction
T_{min}, T_{max}	0.619, 0.746
No. of measured, independent and observed [$I > 2\sigma(I)$] reflections	37660, 10275, 7505
R_{int}	0.045
$(\sin \theta/\lambda)_{max}$ (Å^{-1})	0.661
**Refinement**	
$R[F^2 > 2\sigma(F^2)]$, $wR(F^2)$, S	0.059, 0.151, 1.06
No. of reflections	10275
No. of parameters	535
H-atom treatment	H atoms treated by a mixture of independent and constrained refinement
$\Delta\rho_{max}$, $\Delta\rho_{min}$ (e Å^{-3})	0.36, −0.34

Computer programs: Bruker Instrument Service vV6.2.3, *APEX3* v2018.1-0 (Bruker AXS), *SAINT* V8.38A (Bruker AXS Inc., 2016), *SHELXT* 2014/5 (Sheldrick, 2014), *SHELXL2018*/3 (Sheldrick, 2018), Bruker *SHELXTL*.

D—H···*A*	*D*—H	H···*A*	*D*···*A*	*D*—H···*A*
N2—H2···N5	0.95(3)	2.01(3)	2.637(2)	122(2)

Table S25. Crystal data and structure refinement for **5ba**.

Crystal data	
Chemical formula	$C_{37}H_{41}N_5$
M_r	555.75
Crystal system, space group	Monoclinic, $P2_1/n$
Temperature (K)	150
a, *b*, *c* (Å)	17.5395(7), 10.8508(5), 18.9592(11)
β (°)	117.317(1)
V (Å^3)	3205.9(3)
Z	4
Radiation type	Mo*K*α
μ (mm^{-1})	0.07
Crystal size (mm)	0.31 × 0.25 × 0.14
**Data collection**	
Diffractometer	Bruker D8 - Venture
Absorption correction	Multi-scan *SADABS2016*/2 - Bruker AXS area detector scaling and absorption correction
T_{min}, T_{max}	0.667, 0.746
No. of measured, independent and observed [$I > 2\sigma(I)$] reflections	32072, 6184, 5413
R_{int}	0.034
$(\sin \theta/\lambda)_{max}$ (Å^{-1})	0.617
**Refinement**	
$R[F^2 > 2\sigma(F^2)]$, $wR(F^2)$, S	0.077, 0.175, 1.03
No. of reflections	6184
No. of parameters	443
No. of restraints	134
H-atom treatment	H atoms treated by a mixture of independent and constrained refinement
$\Delta\rho_{max}$, $\Delta\rho_{min}$ (e Å^{-3})	0.71, −0.41

Computer programs: Bruker Instrument Service vV6.2.3, APEX3 v2018.1-0 (Bruker AXS), SAINT V8.38A (Bruker AXS Inc., 2016), SHELXT 2014/5 (Sheldrick, 2014), SHELXL2018/3 (Sheldrick, 2018), Bruker SHELXTL.

D—H···*A*	*D*—H	H···*A*	*D*···*A*	*D*—H···*A*
N2—H2···N5	0.87(2)	2.01(6)	2.63(4)	127(2)

Table S26. Crystal data and structure refinement for **6**.

Crystal data	
Chemical formula	$C_{23}H_{19}N_3O$
M_r	353.41
Crystal system, space group	Monoclinic, $P2_1/c$
Temperature (K)	150
a, *b*, *c* (Å)	24.125(3), 5.2560(7), 14.841(2)
β (°)	106.785(5)
V (Å^3)	1801.7(4)
Z	4
Radiation type	Mo*K*α
μ (mm^{-1})	0.08
Crystal size (mm)	0.59 × 0.20 × 0.09
**Data collection**	
Diffractometer	Bruker D8 - Venture
Absorption correction	Multi-scan *SADABS2016*/2 - Bruker AXS area detector scaling and absorption correction
T_{min}, T_{max}	0.708, 0.746
No. of measured, independent and observed [$I > 2\sigma(I)$] reflections	37521, 4131, 3100
R_{int}	0.089
$(\sin \theta/\lambda)_{max}$ (Å^{-1})	0.651
**Refinement**	
$R[F^2 > 2\sigma(F^2)]$, $wR(F^2)$, S	0.057, 0.128, 1.11
No. of reflections	4131
No. of parameters	250
H-atom treatment	H atoms treated by a mixture of independent and constrained refinement
$\Delta\rho_{max}$, $\Delta\rho_{min}$ (e Å^{-3})	0.22, −0.24

Computer programs: Bruker Instrument Service vV6.2.3, *APEX3* v2018.1-0 (Bruker AXS), *SAINT* V8.38A (Bruker AXS Inc., 2016), *SHELXT* 2014/5 (Sheldrick, 2014), *SHELXL2018*/3 (Sheldrick, 2018), Bruker *SHELXTL*.

D—H···*A*	*D*—H	H···*A*	*D*···*A*	*D*—H···*A*
N2—H1···O1	0.89(2)	2.10(2)	2.661(2)	120(2)

Table S27. Crystal data and structure refinement for **7**.

Chemical formula	$C_{24}H_{21}N_3O_2$
M_r	383.44
Crystal system, space group	Orthorhombic, $Pca2_1$
Temperature (K)	150
a, *b*, *c* (Å)	15.187(3), 4.997(2), 51.139(10)
V (Å³)	3880.8(19)
Z	8
Radiation type	Mo*K*α
μ (mm⁻¹)	0.09
Crystal size (mm)	0.59 × 0.21 × 0.16
Data collection	
Diffractometer	Bruker D8 - Venture
Absorption correction	Multi-scan<br>*SADABS2016*/2 - Bruker AXS area detector scaling and absorption correction
T_{min}, T_{max}	0.598, 0.746
No. of measured, independent and observed [$I > 2\sigma(I)$] reflections	16360, 6410, 5354
R_{int}	0.036
$(\sin \theta/\lambda)_{max}$ (Å⁻¹)	0.649
Refinement	
$R[F^2 > 2\sigma(F^2)]$, $wR(F^2)$, S	0.047, 0.108, 1.05
No. of reflections	6410
No. of parameters	537
No. of restraints	1
H-atom treatment	H atoms treated by a mixture of independent and constrained refinement
$\Delta\rho_{max}$, $\Delta\rho_{min}$ (e Å⁻³)	0.20, −0.24
Absolute structure	Flack x determined using 1418 quotients [(I+)-(I-)]/[(I+)+(I-)] (Parsons, Flack and Wagner, Acta Cryst. B69 (2013) 249-259).

Computer programs: Bruker Instrument Service vV6.2.3, *APEX3* v2018.1-0 (Bruker AXS), *SAINT* V8.38A (Bruker AXS Inc., 2016), *SHELXT* 2014/5 (Sheldrick, 2014), *SHELXL2018*/3 (Sheldrick, 2018), Bruker *SHELXTL*.

D—H···*A*	*D*—H	H···*A*	*D*···*A*	*D*—H···*A*
N2—H1···O1	0.88(5)	2.04(4)	2.631(3)	125(4)

Table S28. Crystal data and structure refinement for **8**.

Crystal data	
Chemical formula	$C_{21}H_{17}N_3O_2$
M_r	343.38
Crystal system, space group	Monoclinic, $P2_1/c$
Temperature (K)	150
a, *b*, *c* (Å)	21.8088(15), 5.2435(4), 14.8310(11)
β (°)	96.820(3)
V (Å^3)	1684.0(2)
Z	4
Radiation type	Mo*K*α
μ (mm^{-1})	0.09
Crystal size (mm)	0.51 × 0.39 × 0.17
**Data collection**	
Diffractometer	Bruker D8 – Venture
Absorption correction	Multi-scan<br>*SADABS2016*/2 – Bruker AXS area detector scaling and absorption correction
T_{min}, T_{max}	0.632, 0.745
No. Of measured, independent and observed [$I > 2\sigma(I)$] reflections	31280, 3275, 2964
R_{int}	0.060
$(\sin \theta/\lambda)_{max}$ (Å^{-1})	0.617
**Refinement**	
$R[F^2 > 2\sigma(F^2)]$, $wR(F^2)$, S	0.082, 0.153, 1.33
No. Of reflections	3275
No. Of parameters	272
No. Of restraints	54
H-atom treatment	H atoms treated by a mixture of independent and constrained refinement
$\Delta\rho_{max}$, $\Delta\rho_{min}$ (e Å^{-3})	0.28, −0.24

Computer programs: Bruker Instrument Service vV6.2.3, *APEX3* v2018.1-0 (Bruker AXS), *SAINT* V8.38A (Bruker AXS Inc., 2016), *SHELXT* 2014/5 (Sheldrick, 2014), *SHELXL2018*/3 (Sheldrick, 2018), Bruker *SHELXTL*.

D—H···*A*	*D*—H	H···*A*	*D*···*A*	*D*—H···*A*
N2—H1···O1	0.82(3)	2.08(3)	2.624(3)	123(3)

Table S29. Crystal data and structure refinement for **9**.

Crystal data	
Chemical formula	$C_{21}H_{17}N_3OS$
M_r	359.43
Crystal system, space group	Monoclinic, $P2_1/c$
Temperature (K)	150
a, *b*, *c* (Å)	21.8615(8), 5.3836(2), 14.9093(5)
β (°)	95.639(1)
V (Å^3)	1746.24(11)
Z	4
Radiation type	Mo*K*α
μ (mm^{-1})	0.20
Crystal size (mm)	0.59 × 0.32 × 0.20
**Data collection**	
Diffractometer	Bruker D8 - Venture
Absorption correction	Multi-scan<br>*SADABS2016*/2 - Bruker AXS area detector scaling and absorption correction
T_{min}, T_{max}	0.673, 0.746
No. of measured, independent and observed [$I > 2\sigma(I)$] reflections	37108, 3990, 3161
R_{int}	0.049
$(\sin \theta/\lambda)_{max}$ (Å^{-1})	0.651
**Refinement**	
$R[F^2 > 2\sigma(F^2)]$, $wR(F^2)$, S	0.040, 0.104, 1.05
No. of reflections	3990
No. of parameters	254
No. of restraints	3
H-atom treatment	H atoms treated by a mixture of independent and constrained refinement
$\Delta\rho_{max}$, $\Delta\rho_{min}$ (e Å^{-3})	0.23, −0.31

Computer programs: Bruker Instrument Service vV6.2.3, *APEX3* v2018.1-0 (Bruker AXS), *SAINT* V8.38A (Bruker AXS Inc., 2016), *SHELXT* 2014/5 (Sheldrick, 2014), *SHELXL2018*/3 (Sheldrick, 2018), Bruker *SHELXTL*.

D—H···*A*	*D*—H	H···*A*	*D*···*A*	*D*—H···*A*
N2—H1···O1	0.89(2)	1.987(18)	2.577(2)	122(1)

Table S30. Crystal data and structure refinement for **6r**.

Crystal data	
Chemical formula	$C_{27}H_{31}N_3O_2$
M_r	429.55
Crystal system, space group	Orthorhombic, $Pna2_1$
Temperature (K)	150
a, *b*, *c* (Å)	10.0689(10), 13.8063(15), 17.1437(17)
V (Å^3)	2383.2(4)
Z	4
Radiation type	Mo*K*α
μ (mm^{-1})	0.08
Crystal size (mm)	0.59 × 0.09 × 0.06
**Data collection**	
Diffractometer	Bruker D8 - Venture
Absorption correction	Multi-scan *SADABS2016*/2 - Bruker AXS area detector scaling and absorption correction
T_{min}, T_{max}	0.531, 0.746
No. of measured, independent and observed [$I > 2\sigma(I)$] reflections	11687, 5213, 3965
R_{int}	0.088
$(\sin \theta/\lambda)_{max}$ (Å^{-1})	0.650
**Refinement**	
$R[F^2 > 2\sigma(F^2)]$, $wR(F^2)$, S	0.076, 0.157, 1.13
No. of reflections	5213
No. of parameters	301
No. of restraints	1
H-atom treatment	H atoms treated by a mixture of independent and constrained refinement
$\Delta\rho_{max}$, $\Delta\rho_{min}$ (e Å^{-3})	0.29, −0.34

Computer programs: Bruker Instrument Service vV6.2.3, *APEX3* v2018.1-0 (Bruker AXS), *SAINT* V8.38A (Bruker AXS Inc., 2016), *SHELXT* 2014/5 (Sheldrick, 2014), *SHELXL2018*/3 (Sheldrick, 2018), Bruker *SHELXTL*.

D—H···*A*	*D*—H	H···*A*	*D*···*A*	*D*—H···*A*
N3—H3···O1’	0.88(7)	2.06(7)	2.879(5)	155(6)

Intramolecular

Table S31. Crystal data and structure refinement for **7r**.

Crystal data	
Chemical formula	$C_{24}H_{23}N_3O_2$
M_r	385.45
Crystal system, space group	Monoclinic, $P2_1/c$
Temperature (K)	150
a, *b*, *c* (Å)	13.9453(10), 15.1347(11), 9.9594(8)
β (°)	109.551(2)
V (Å^3)	1980.8(3)
Z	4
Radiation type	Mo*K*α
μ (mm^{-1})	0.08
Crystal size (mm)	0.59 × 0.06 × 0.03
**Data collection**	
Diffractometer	Bruker D8 - Venture
Absorption correction	Multi-scan<br>*SADABS2016*/2 - Bruker AXS area detector scaling and absorption correction
T_{min}, T_{max}	0.463, 0.746
No. of measured, independent and observed [$I > 2\sigma(I)$] reflections	40829, 3669, 2901
R_{int}	0.107
$(\sin \theta/\lambda)_{max}$ (Å^{-1})	0.606
**Refinement**	
$R[F^2 > 2\sigma(F^2)]$, $wR(F^2)$, S	0.052, 0.112, 1.05
No. of reflections	3669
No. of parameters	269
H-atom treatment	H atoms treated by a mixture of independent and constrained refinement
$\Delta\rho_{max}$, $\Delta\rho_{min}$ (e Å^{-3})	0.20, −0.28

Computer programs: Bruker Instrument Service vV6.2.3, *APEX3* v2018.1-0 (Bruker AXS), *SAINT* V8.38A (Bruker AXS Inc., 2016), *SHELXT* 2014/5 (Sheldrick, 2014), *SHELXL2018*/3 (Sheldrick, 2018), Bruker *SHELXTL*.

D—H···*A*	*D*—H	H···*A*	*D*···*A*	*D*—H···*A*
N3—H3···O1′	0.88	2.086(2)	2.933(2)	161.2

Intramolecular

Table S32. Crystal data and structure refinement for **8r**.

Crystal data	
Chemical formula	$C_{25}H_{29}N_3O_3$
M_r	419.51
Crystal system, space group	Orthorhombic, $Pna2_1$
Temperature (K)	150
a, *b*, *c* (Å)	9.9966(10), 13.9343(14), 16.9378(16)
V (Å^3)	2359.4(4)
Z	4
Radiation type	Mo*K*α
μ (mm^{-1})	0.08
Crystal size (mm)	0.23 × 0.11 × 0.07
Data collection	
Diffractometer	Bruker D8 - Venture
Absorption correction	Multi-scan *SADABS2016*/2 - Bruker AXS area detector scaling and absorption correction
T_{min}, T_{max}	0.618, 0.746
No. of measured, independent and observed [$I > 2\sigma(I)$] reflections	9679, 3725, 3582
R_{int}	0.054
$(\sin \theta/\lambda)_{max}$ (Å^{-1})	0.595
Refinement	
$R[F^2 > 2\sigma(F^2)]$, $wR(F^2)$, S	0.161, 0.359, 1.13
No. of reflections	3725
No. of parameters	291
No. of restraints	347
H-atom treatment	H atoms treated by a mixture of independent and constrained refinement<br>$w = 1/[\sigma^2(F_o^2) + (0.0003P)^2 + 31.8788P]$ where $P = (F_o^2 + 2F_c^2)/3$
$\Delta\rho_{max}$, $\Delta\rho_{min}$ (e Å^{-3})	0.71, −0.61
Absolute structure	Flack x determined using 1309 quotients [(I+)-(I-)]/[(I+)+(I-)] (Parsons, Flack and Wagner, Acta Cryst. B69 (2013) 249-259).
Absolute structure parameter	-1(8)

Computer programs: Bruker Instrument Service vV6.2.3, *APEX3* v2018.1-0 (Bruker AXS), *SAINT* V8.38A (Bruker AXS Inc., 2016), *SHELXT* 2014/5 (Sheldrick, 2014), *SHELXL2018*/3 (Sheldrick, 2018), Bruker *SHELXTL*.

D—H···*A*	*D*—H	H···*A*	*D*···*A*	*D*—H···*A*
N3—H3···O1′	0.88	1.982(2)	2.82(1)	159.9

Intramolecular

Table S33. Crystal data and structure refinement for **9r**.

Crystal data	
Chemical formula	$C_{25}H_{29}N_3O_2S$
M_r	435.57
Crystal system, space group	Orthorhombic, $Pna2_1$
Temperature (K)	150
a, *b*, *c* (Å)	10.0126(4), 13.8835(4), 17.0785(6)
V (Å^3)	2374.08(14)
Z	4
Radiation type	Mo*K*α
μ (mm^{-1})	0.16
Crystal size (mm)	0.59 × 0.15 × 0.05
**Data collection**	
Diffractometer	Bruker D8 – Venture
Absorption correction	Multi-scan *SADABS2016*/2 - Bruker AXS area detector scaling and absorption correction
T_{min}, T_{max}	0.613, 0.746
No. of measured, independent and observed [$I > 2\sigma(I)$] reflections	21250, 5385, 4971
R_{int}	0.043
$(\sin \theta/\lambda)_{max}$ (Å^{-1})	0.650
**Refinement**	
$R[F^2 > 2\sigma(F^2)]$, $wR(F^2)$, S	0.039, 0.085, 1.07
No. of reflections	5385
No. of parameters	291
No. of restraints	1
H-atom treatment	H atoms treated by a mixture of independent and constrained refinement
$\Delta\rho_{max}$, $\Delta\rho_{min}$ (e Å^{-3})	0.16, −0.26
Absolute structure	Flack x determined using 2108 quotients [(I+)-(I-)]/[(I+)+(I-)] (Parsons, Flack and Wagner, Acta Cryst. B69 (2013) 249-259).
Absolute structure parameter	0.05(2)

Computer programs: Apex4 v2022.1-1 (Bruker, 2022), *SAINT* V8.40B (Bruker AXS LLC, 2019), *SHELXT* 2014/5 (Sheldrick, 2014), *SHELXL2018*/3 (Sheldrick, 2018), Bruker *SHELXTL*.

D—H···*A*	*D*—H	H···*A*	*D*···*A*	*D*—H···*A*
N3—H3···O1′	0.83(3)	2.12(3)	2.873(3)	167(3)

Intramolecular

Table S34. Crystal data and structure refinement for (Li**6'**)$_2$·H_2O.

Crystal data	
Chemical formula	$C_{32}H_{25.50}LiN_5O_{1.75}$
M_r	515.01
Crystal system, space group	Triclinic, *P*-1
Temperature (K)	150
a, *b*, *c* (Å)	15.7049(5), 18.6016(6), 20.5934(6)
α, β, γ (°)	106.632(1), 107.823(1), 90.111(2)
V (Å^3)	5461.2(3)
Z	8
Radiation type	Mo*K*α
μ (mm^{-1})	0.08
Crystal size (mm)	0.08 × 0.10 × 0.32
Data collection	
Diffractometer	Bruker D8 – Venture
Absorption correction	Multi-scan *SADABS2016*/2 - Bruker AXS area detector scaling and absorption correction
No. of measured, independent and observed [$I > 2\sigma(I)$] reflections	243701, 25040, 15747
R_{int}	0.137
$(\sin \theta/\lambda)_{max}$ (Å^{-1})	0.650
Refinement	
$R[F^2 > 2\sigma(F^2)]$, $wR(F^2)$, S	0.055, 0.163, 1.02
No. of reflections	25040
No. of parameters	1454
No. of restraints	1516
H-atom treatment	H atoms treated by a mixture of independent and constrained refinement
$\Delta\rho_{max}$, $\Delta\rho_{min}$ (e Å^{-3})	0.98, −0.71

Computer programs: *SHELXL2019*/1 (Sheldrick, 2019).

Table S35. Crystal data and structure refinement for **6’**.

Crystal data	
Chemical formula	$C_{31}H_{23}N_5O$
M_r	481.54
Crystal system, space group	Monoclinic, *P*2/*c*
Temperature (K)	150
a, *b*, *c* (Å)	13.1758(4), 9.9218(4), 19.0493(8)
β (°)	91.595(1)
V (Å^3)	2489.31(16)
Z	4
Radiation type	Mo*K*α
μ (mm^{-1})	0.08
Crystal size (mm)	0.53 × 0.28 × 0.13
**Data collection**	
Diffractometer	Bruker D8 – Venture
Absorption correction	Multi-scan *SADABS2016*/2 - Bruker AXS area detector scaling and absorption correction
T_{min}, T_{max}	0.620, 0.746
No. of measured, independent and observed [$I > 2\sigma(I)$] reflections	34715, 5556, 4248
R_{int}	0.082
$(\sin \theta/\lambda)_{max}$ (Å^{-1})	0.651
**Refinement**	
$R[F^2 > 2\sigma(F^2)]$, $wR(F^2)$, S	0.049, 0.129, 1.04
No. of reflections	5556
No. of parameters	340
H-atom treatment	H atoms treated by a mixture of independent and constrained refinement
$\Delta\rho_{max}$, $\Delta\rho_{min}$ (e Å^{-3})	0.27, −0.38

Computer programs: Bruker Instrument Service vV6.2.3, *APEX3* v2018.1-0 (Bruker AXS), *SAINT* V8.38A (Bruker AXS Inc., 2016), *SHELXT* 2014/5 (Sheldrick, 2014), *SHELXL2018*/3 (Sheldrick, 2018), Bruker *SHELXTL*.

D—H···*A*	*D*—H	H···*A*	*D*···*A*	*D*—H···*A*
N4—H4···O1	0.90(2)	2.286(15)	2.738(2)	111(1)
N4—H4···N2	0.90(2)	2.117(18)	2.707(2)	122(1)

Table S36. Crystal data and structure refinement for (Li**1e**)$_2$**1e**$_2$Li$_2$.

Crystal data	
Chemical formula	$C_{50}H_{72}Li_4N_{12}Si_6$
M_r	1037.49
Crystal system, space group	Triclinic, *P*-1
Temperature (K)	150
a, *b*, *c* (Å)	9.9822(17), 12.8466(18), 13.402(3)
α, β, γ (°)	104.469(9), 94.613(10), 100.490(6)
V (Å^3)	1622.0(5)
Z	1
Radiation type	Mo*K*α
μ (mm^{-1})	0.17
Crystal size (mm)	0.26 × 0.22 × 0.20
Data collection	
Diffractometer	Bruker D8 – Venture
Absorption correction	Multi-scan *SADABS2016*/2 - Bruker AXS area detector scaling and absorption correction
No. of measured, independent and observed [$I > 2\sigma(I)$] reflections	48163, 7464, 5296
R_{int}	0.076
$(\sin \theta/\lambda)_{max}$ (Å^{-1})	0.652
Refinement	
$R[F^2 > 2\sigma(F^2)]$, $wR(F^2)$, S	0.065, 0.206, 0.99
No. of reflections	7464
No. of parameters	351
No. of restraints	367
H-atom treatment	H atoms treated by a mixture of independent and constrained refinement
$(\Delta/\sigma)_{max}$	0.140
$\Delta\rho_{max}$, $\Delta\rho_{min}$ (e Å^{-3})	0.58, −0.70

Computer programs: *SHELXL2013* (Sheldrick, 2013).

8 NMR spectra of prepared compounds

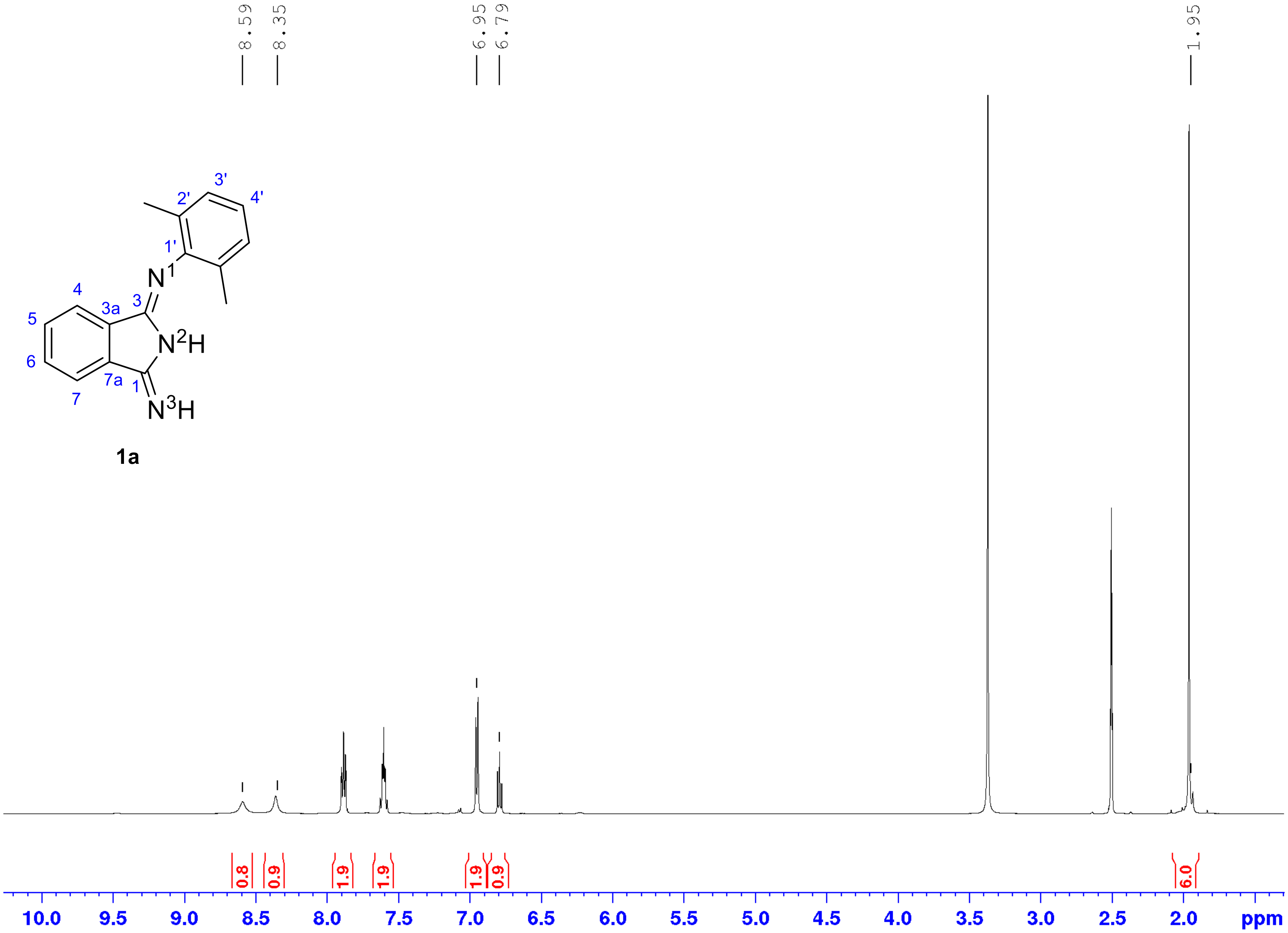

Figure S83. ^{1}H NMR spectrum of compound **1a** in DMSO-d_6 at room temperature.

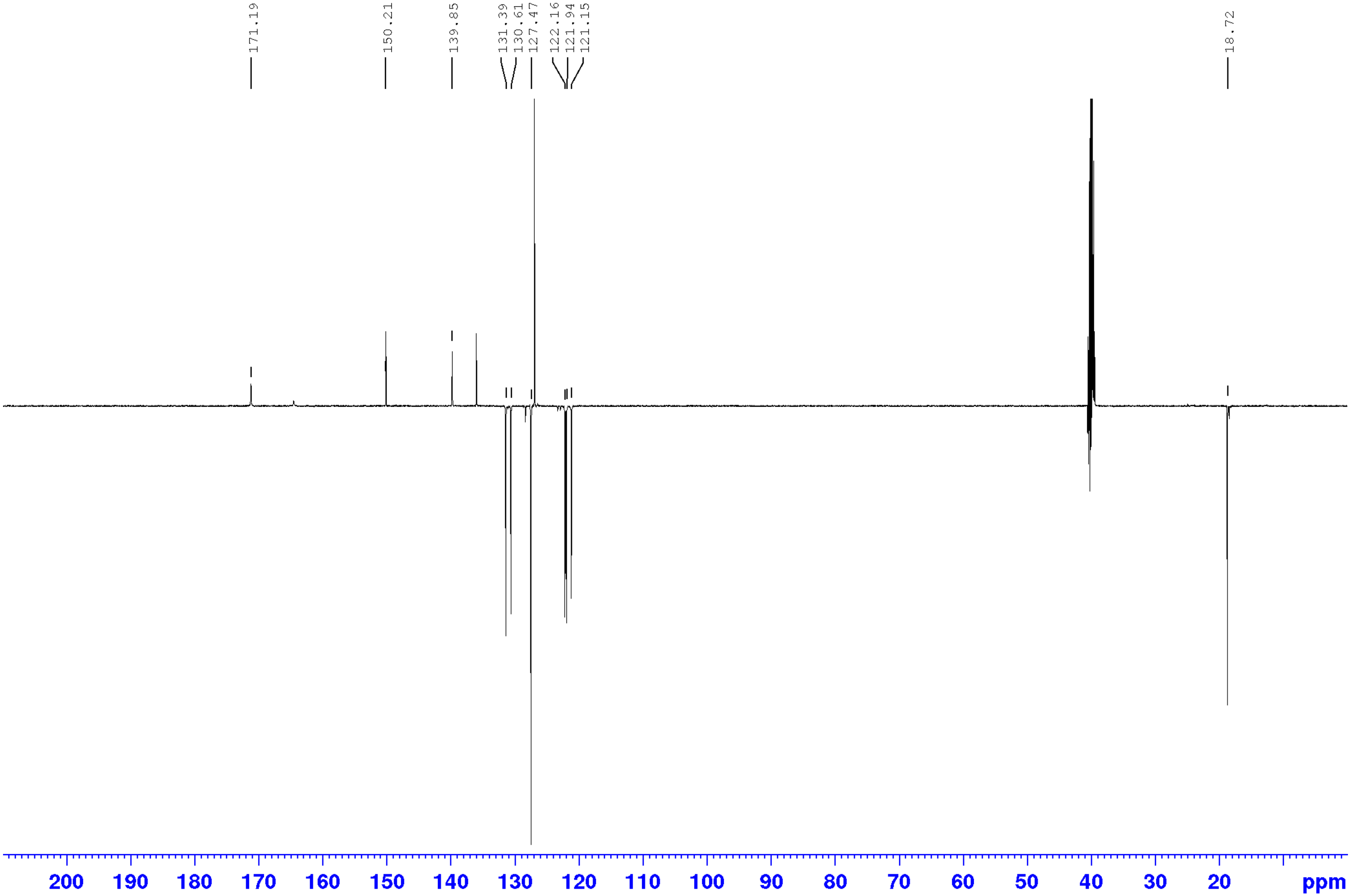

Figure S84. ^{13}C NMR spectrum for compound **1a** in DMSO-d_6 at room temperature.

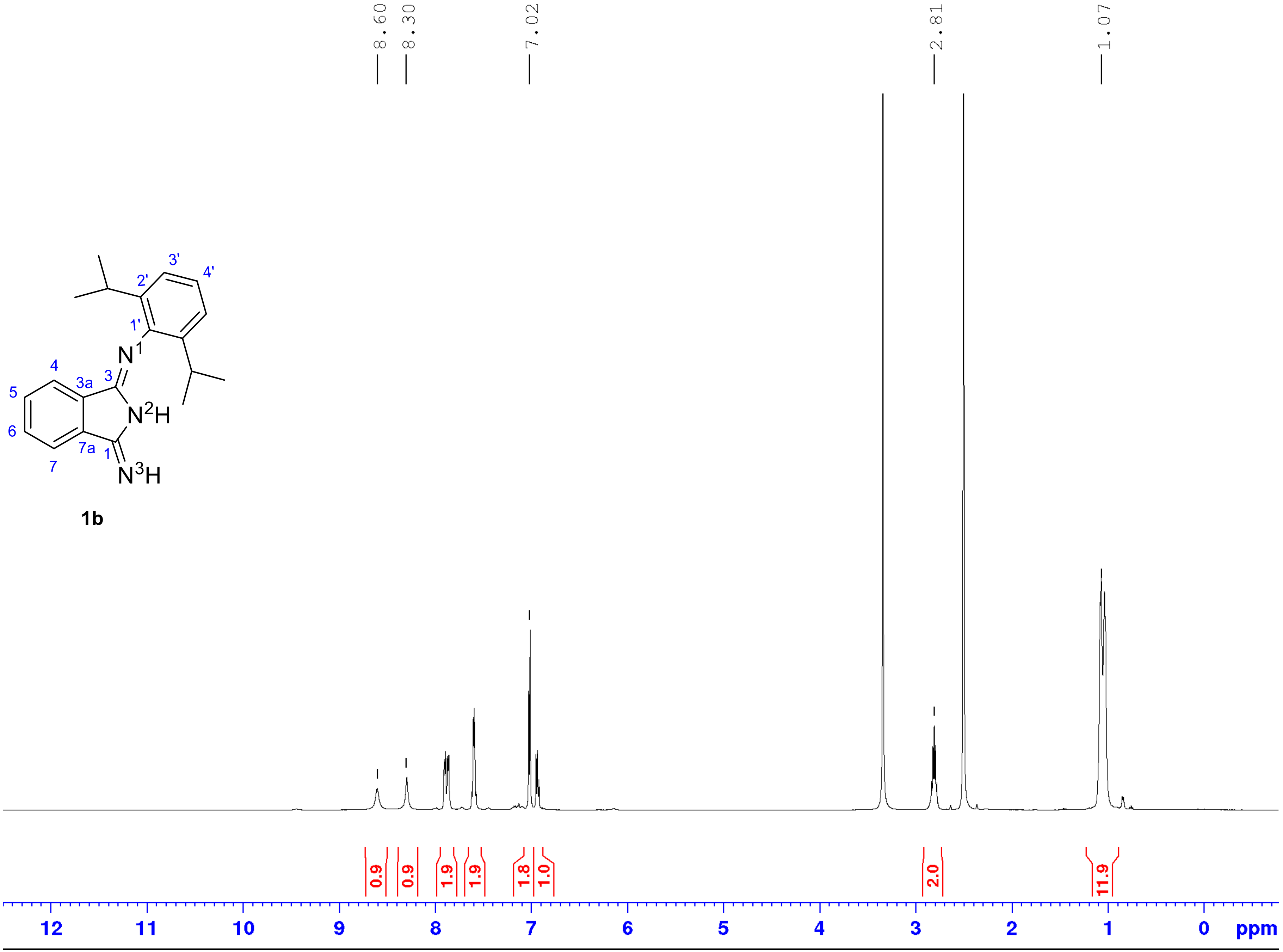

1b

Figure S85. ^{1}H NMR spectrum for compound **1b** in DMSO-d_6 at room temperature.

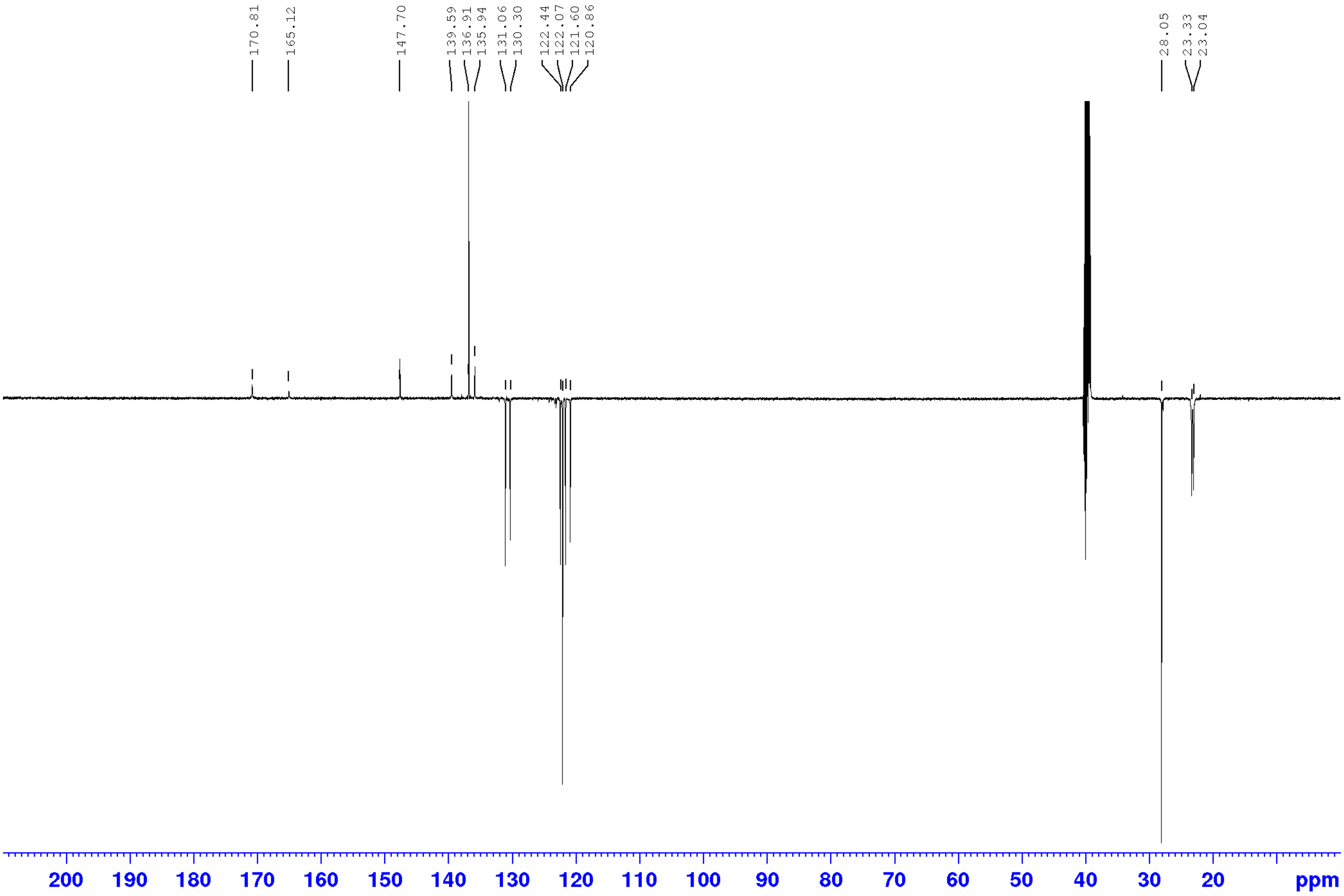

Figure S86. ^{13}C NMR spectrum for compound **1b** in DMSO-d_6 at room temperature.

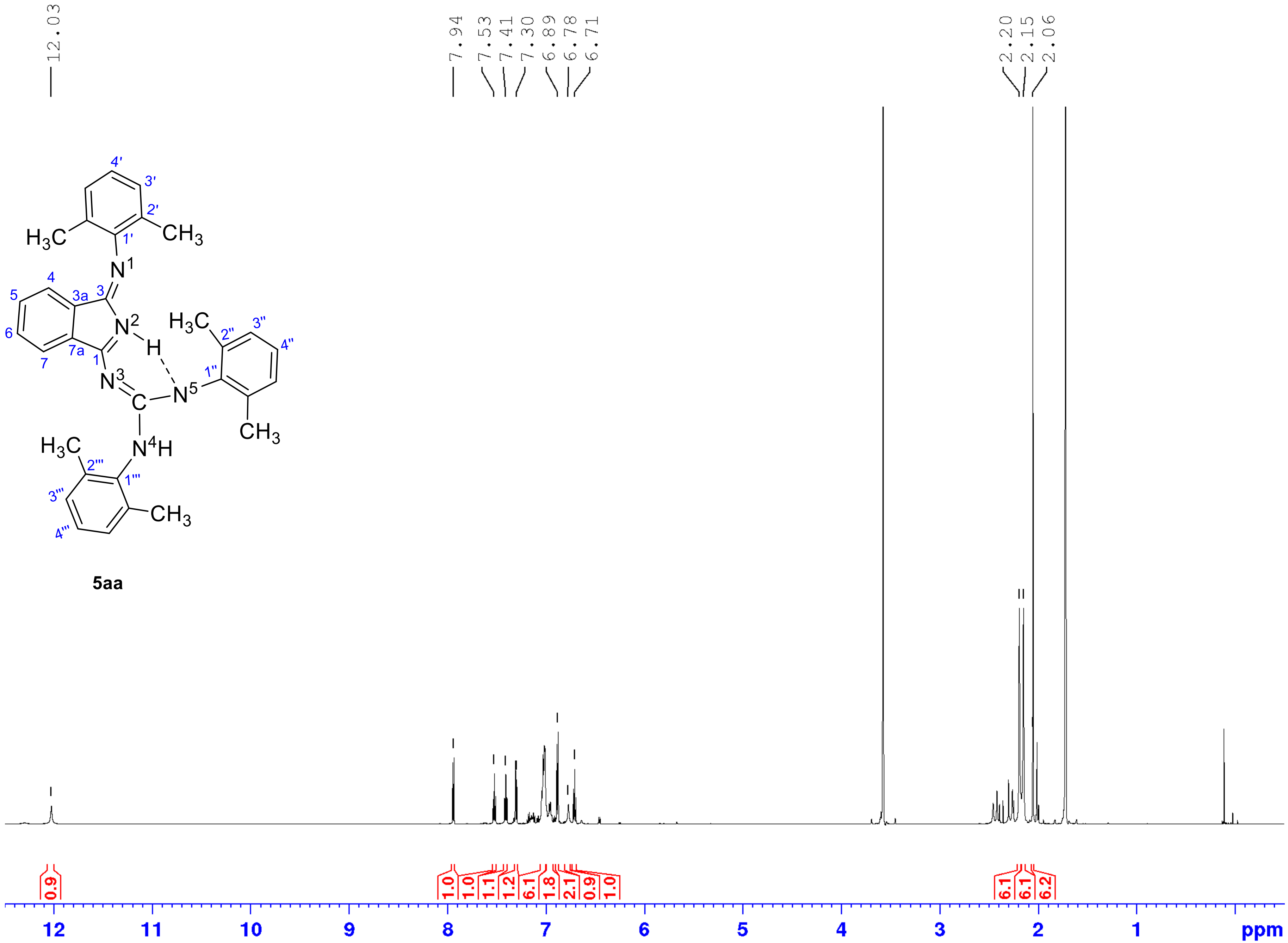

Figure S87. ^{1}H NMR spectrum of compound **5aa** in THF-d_8 at room temperature.

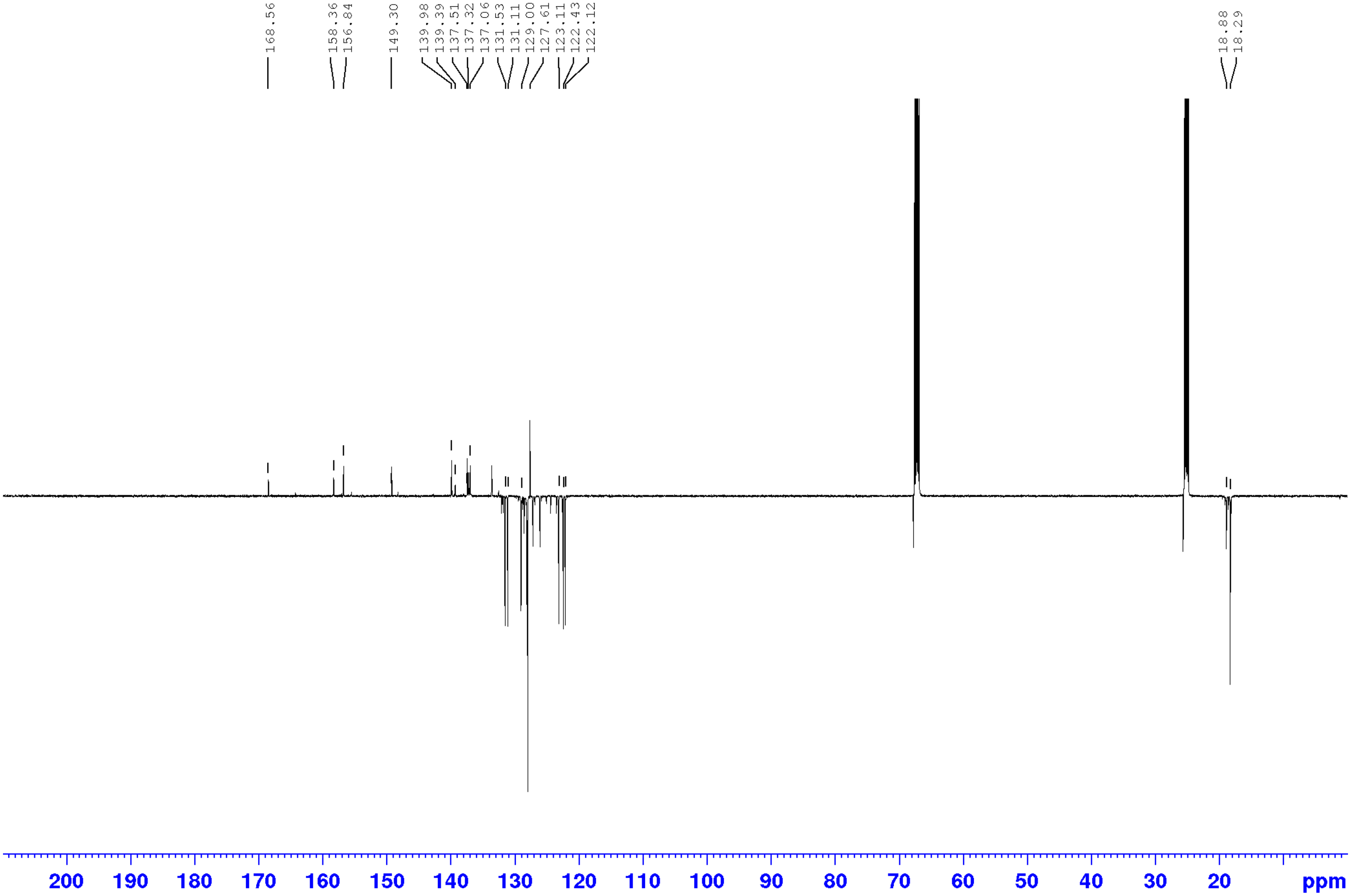

Figure S88. ^{13}C NMR spectrum for compound **5aa** in THF-d_8 at room temperature.

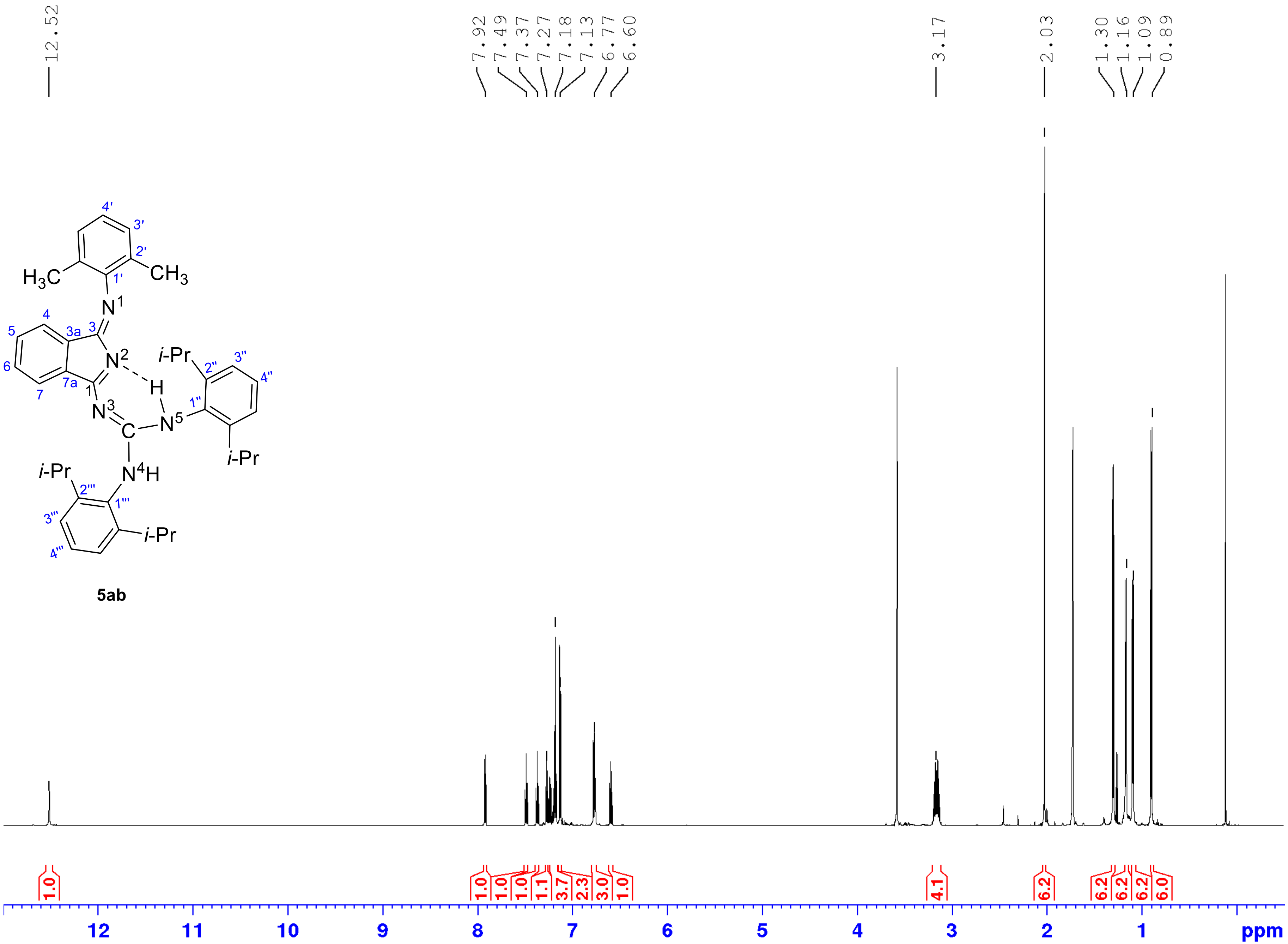

Figure S89. ^{1}H NMR spectrum for compound **5ab** in THF-d_8 at room temperature.

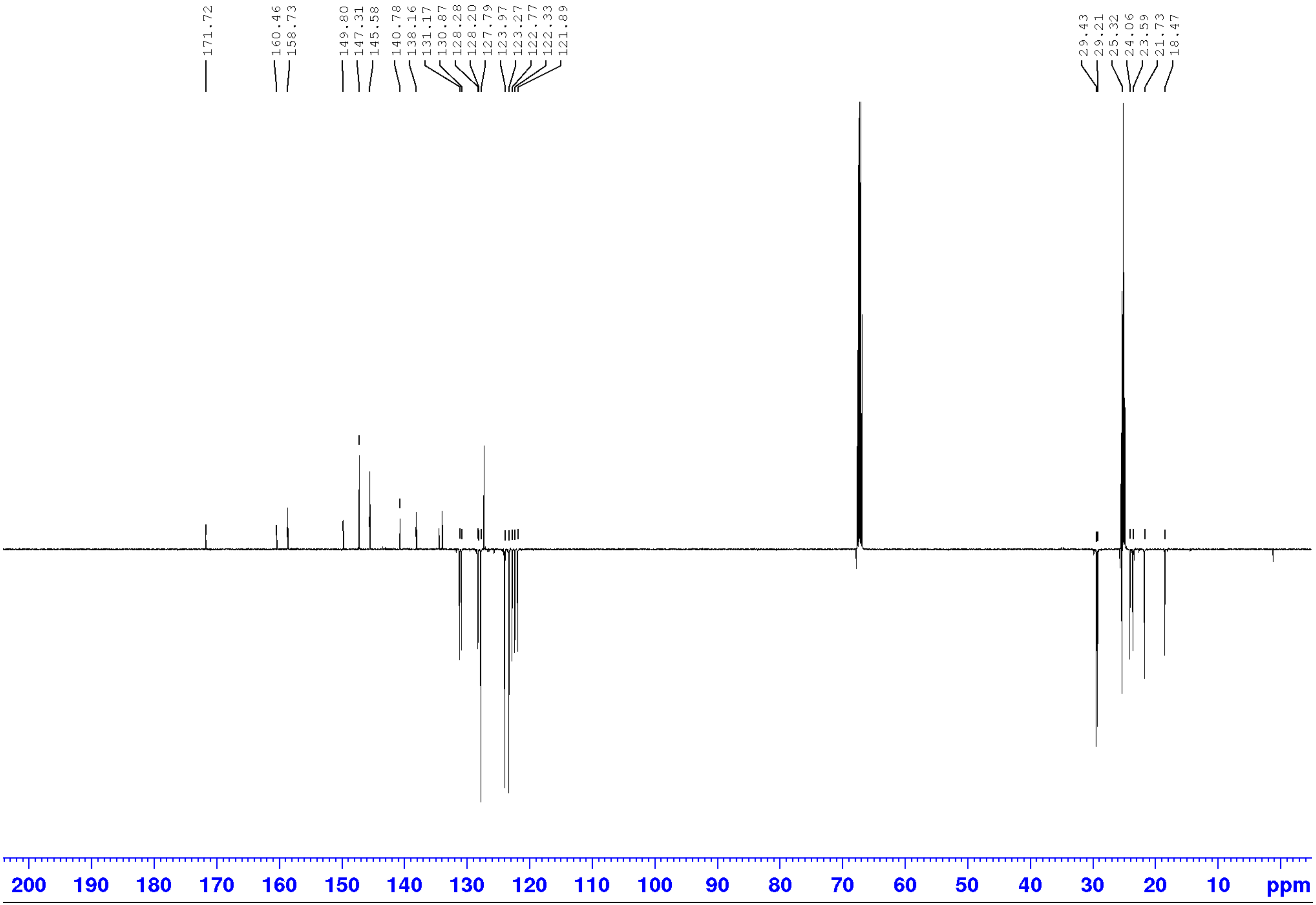

Figure S90. ^{13}C NMR spectrum for compound **5ab** in THF-d_8 at room temperature.

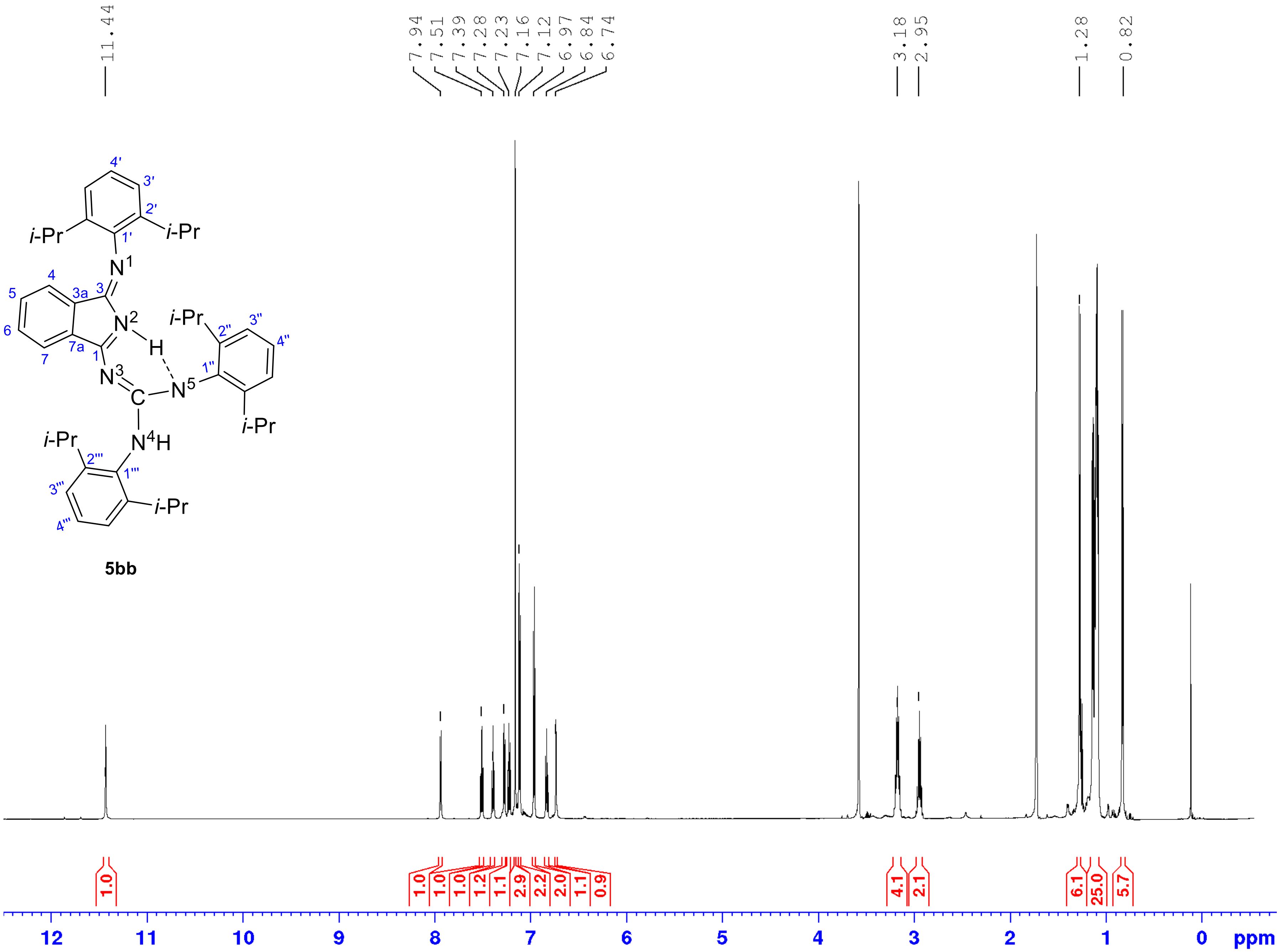

Figure S91. ^{1}H NMR spectrum for compound **5bb** in THF-d_8 at room temperature.

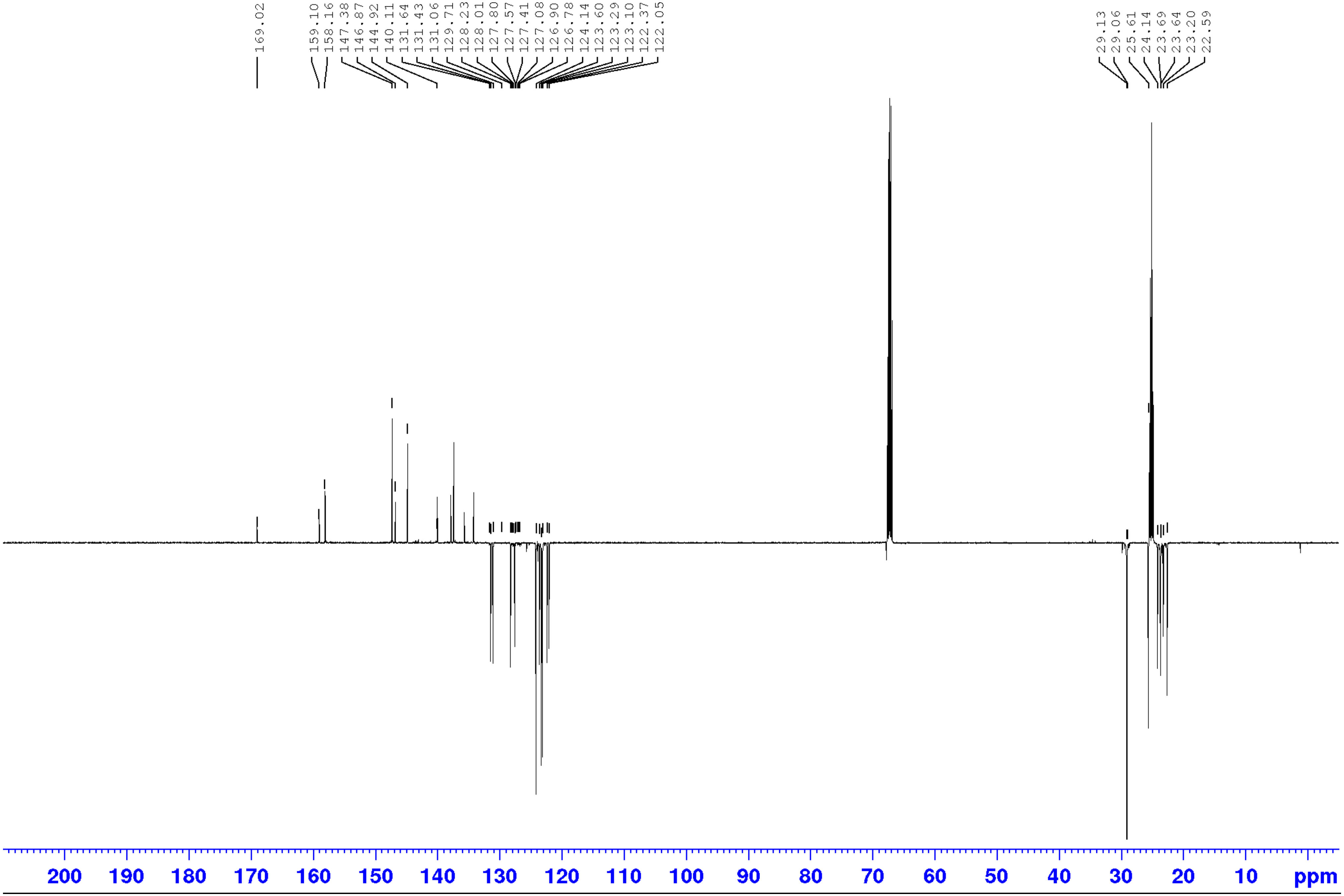

Figure S92. ^{13}C NMR spectrum for compound **5bb** in THF-d_8 at room temperature.

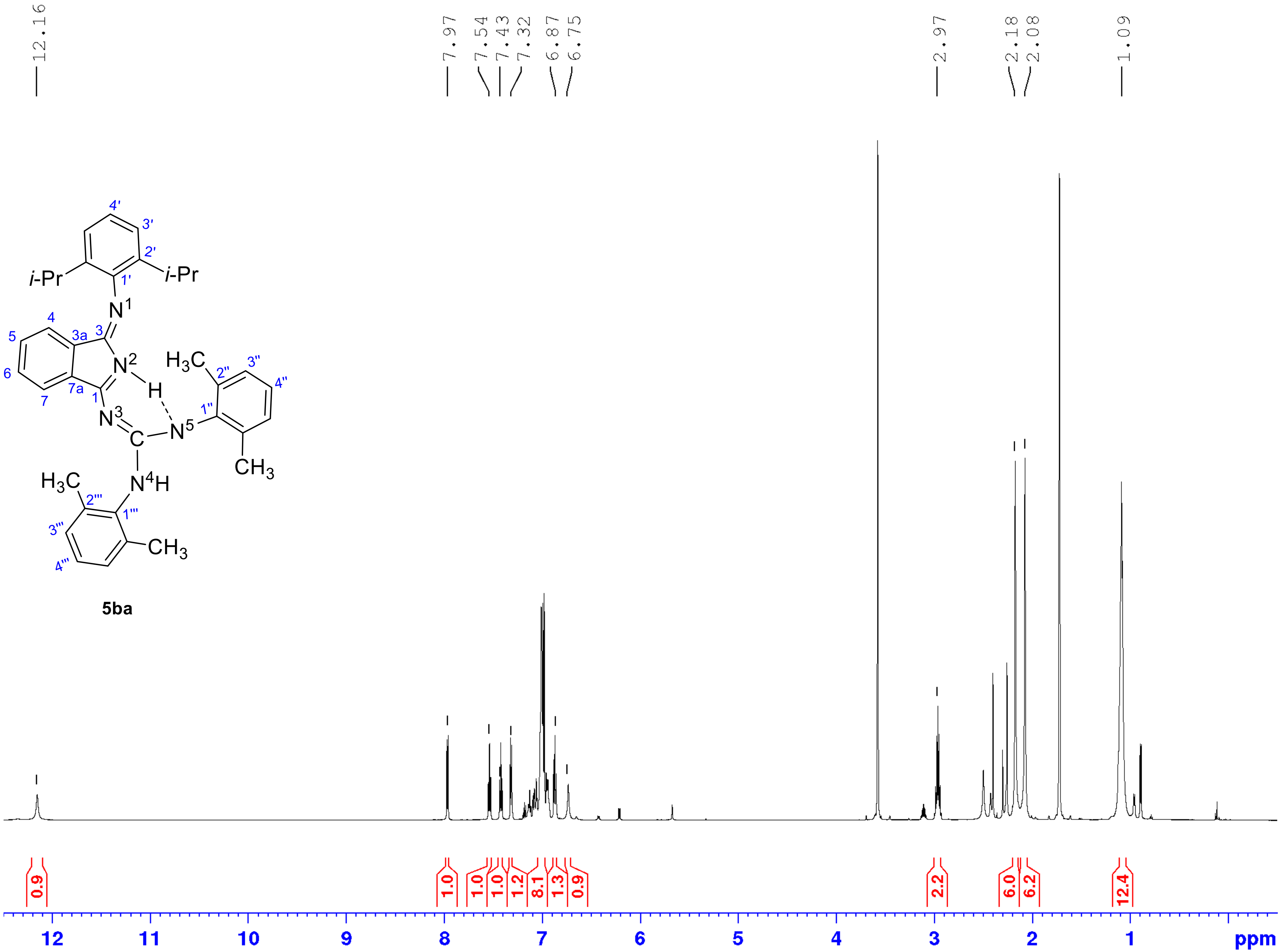

Figure S93. ^{1}H NMR spectrum for compound **5ba** in THF-d_8 at room temperature.

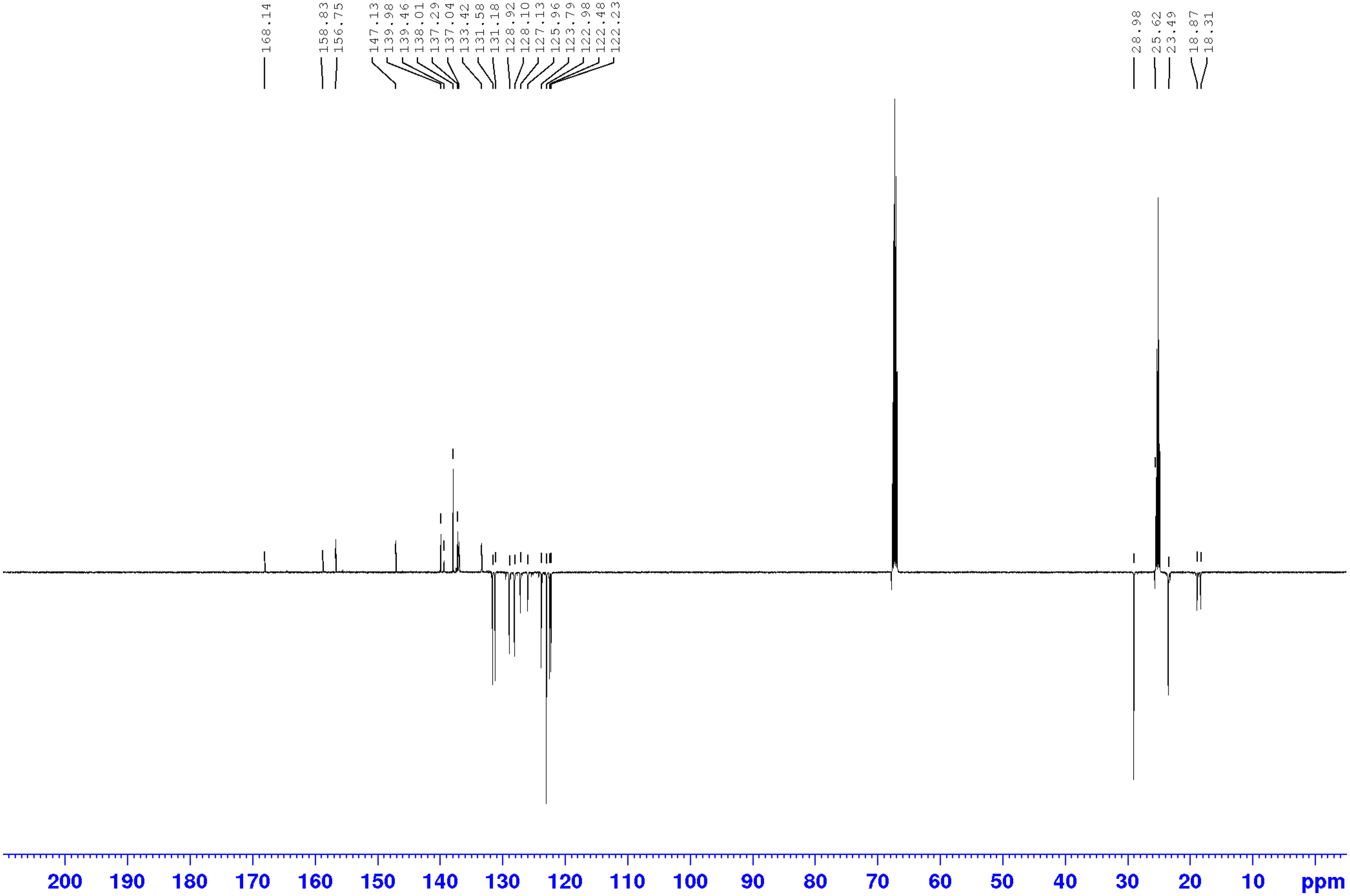

Figure S94. ^{13}C NMR spectrum for compound **5ba** in THF-d_8 at room temperature.

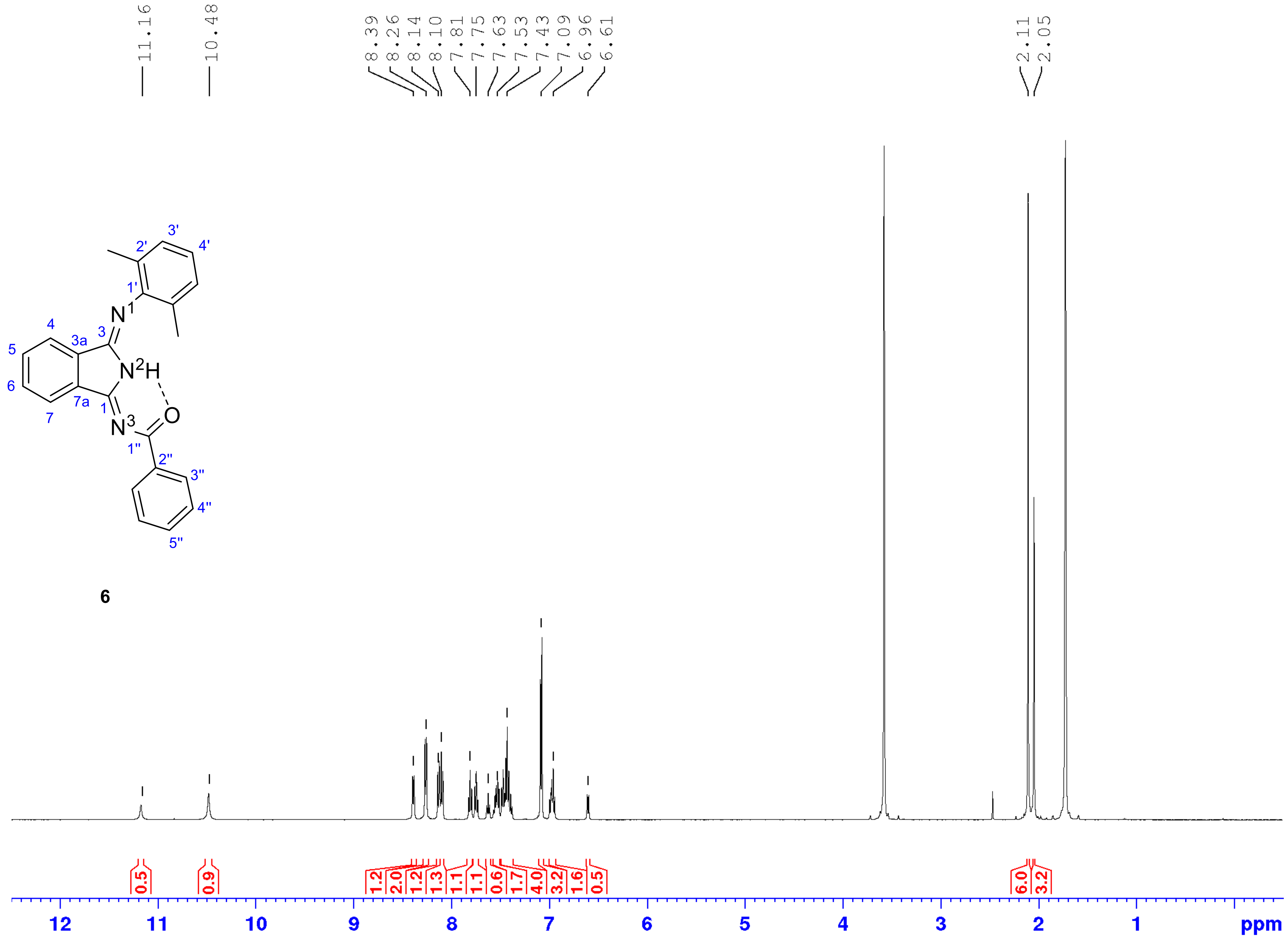

Figure S95. ^{1}H NMR spectrum for compound **6** in THF-d_8 at room temperature.

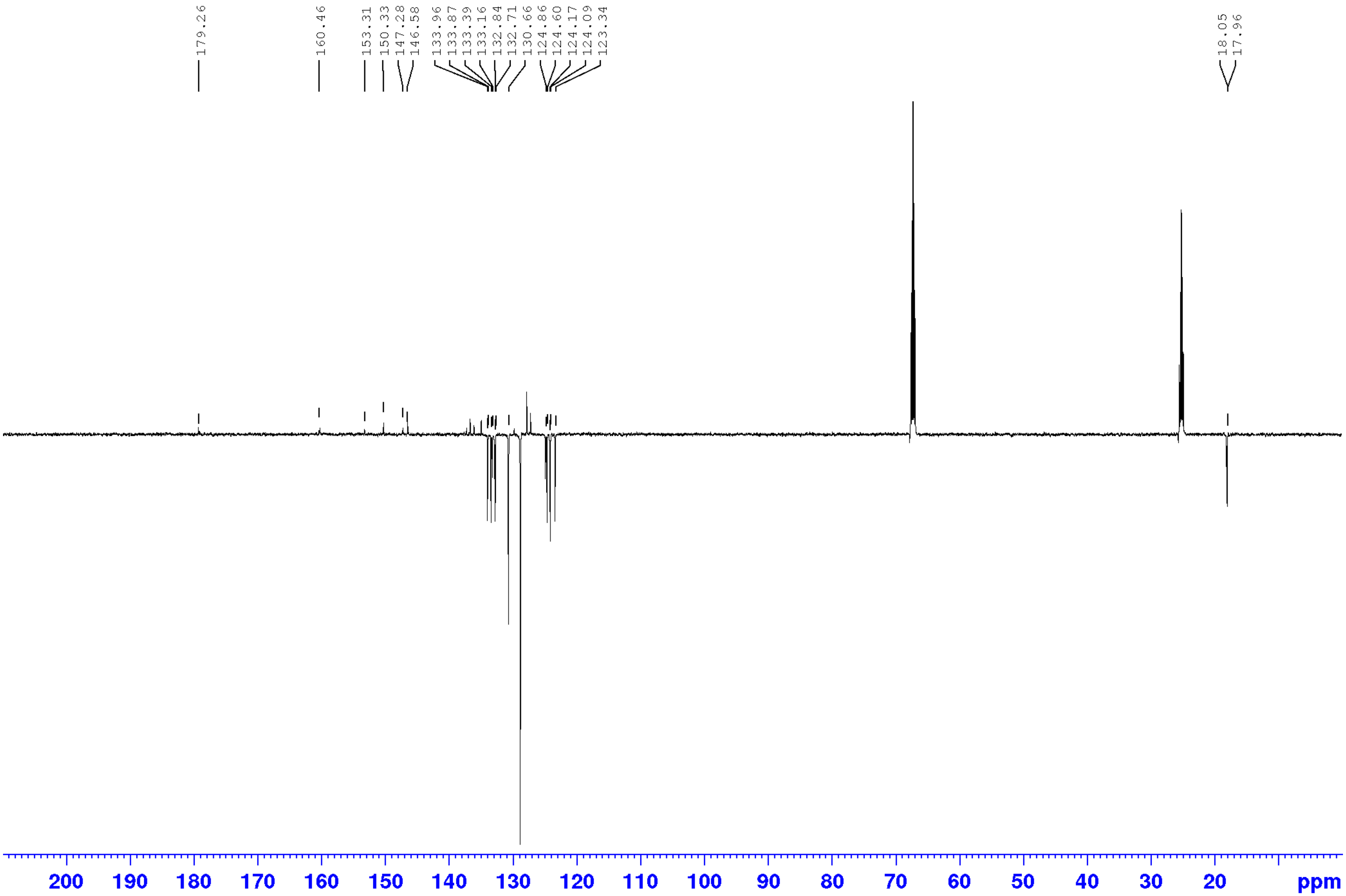

Figure S96. ^{13}C NMR spectrum for compound **6** in THF-d_8 at room temperature.

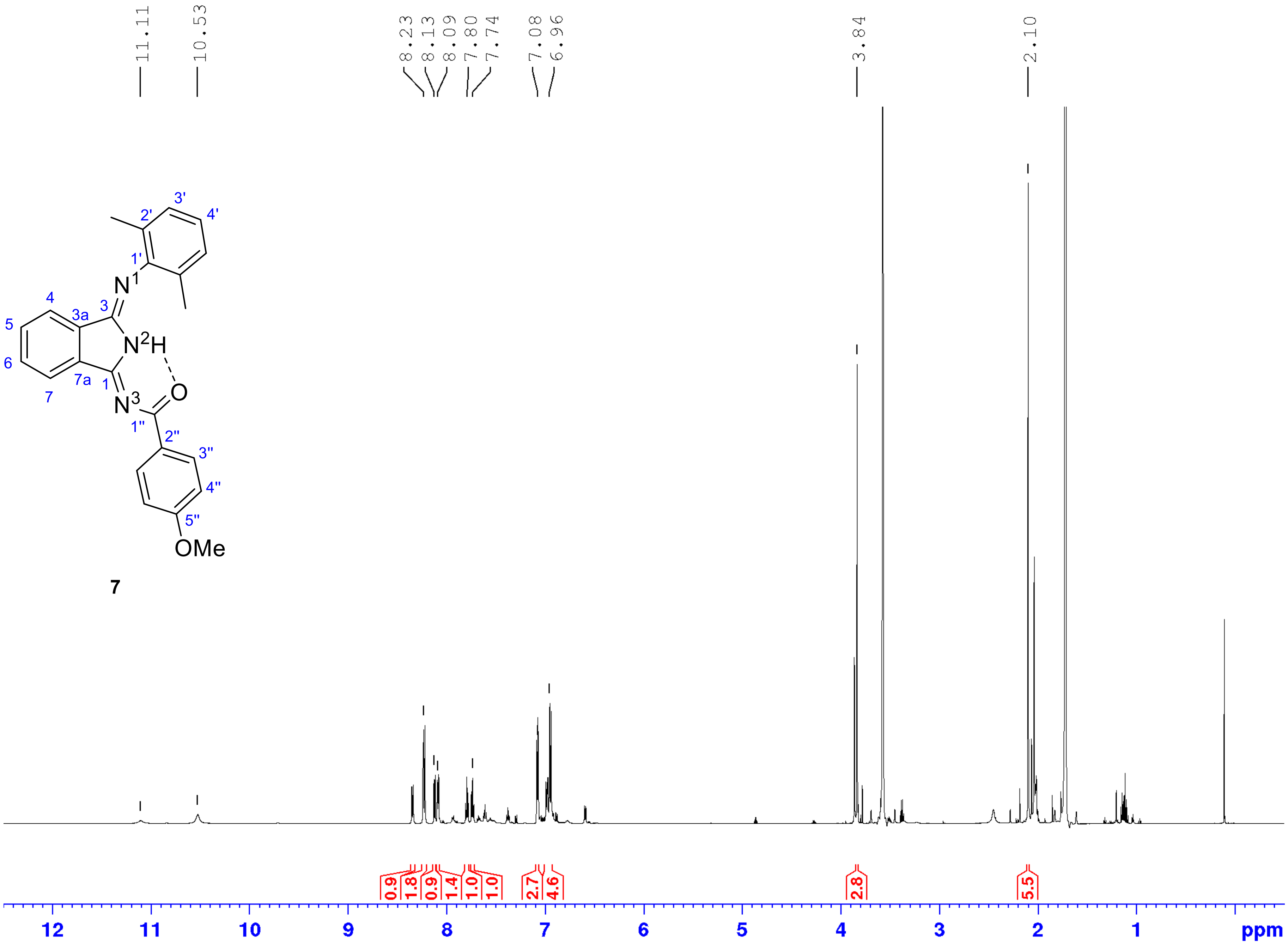

Figure S97. ^{1}H NMR spectrum for compound **7** in THF-d_8 at room temperature.

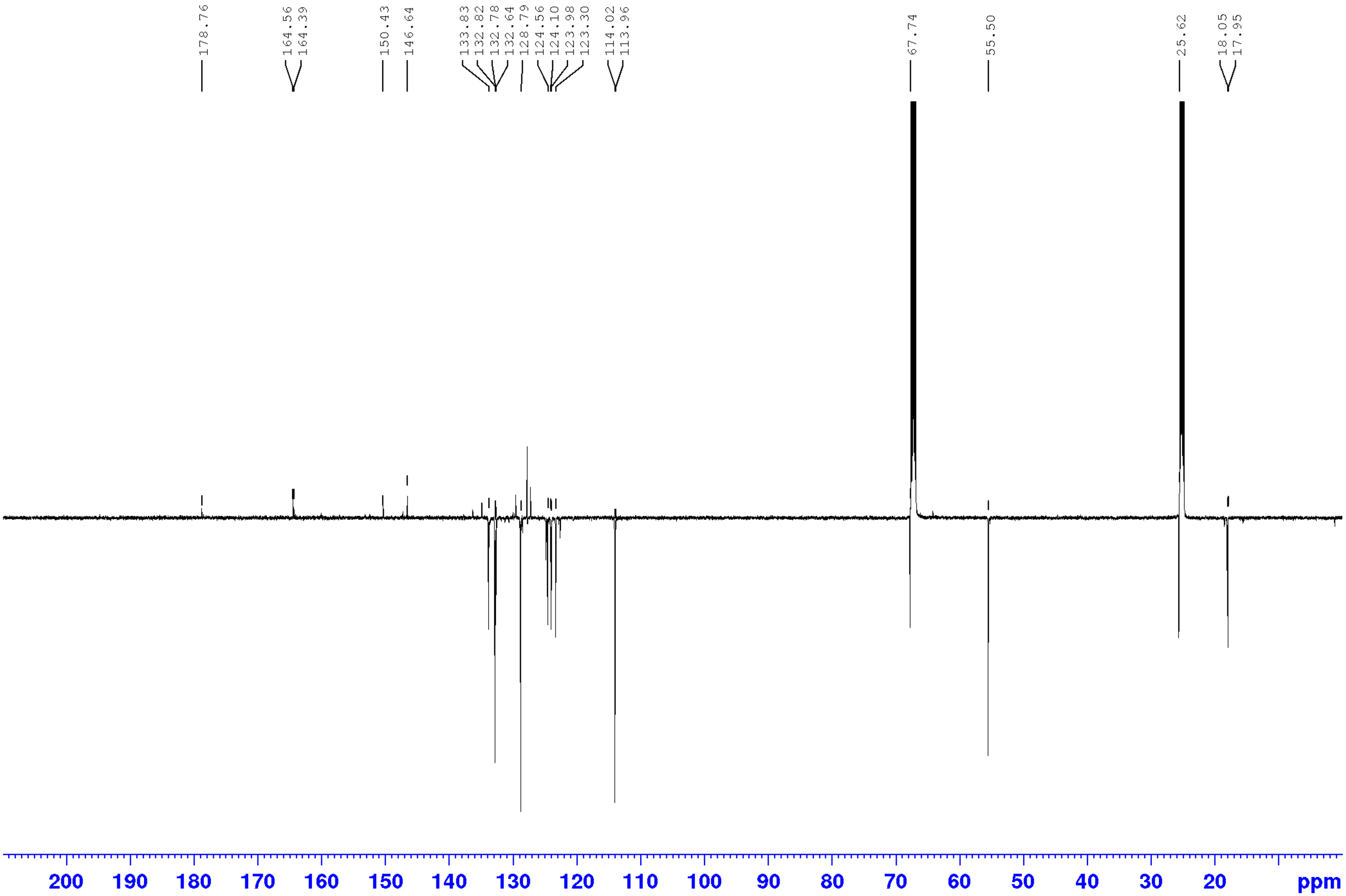

Figure S98. ^{13}C NMR spectrum for compound **7** in THF-d_8 at room temperature.

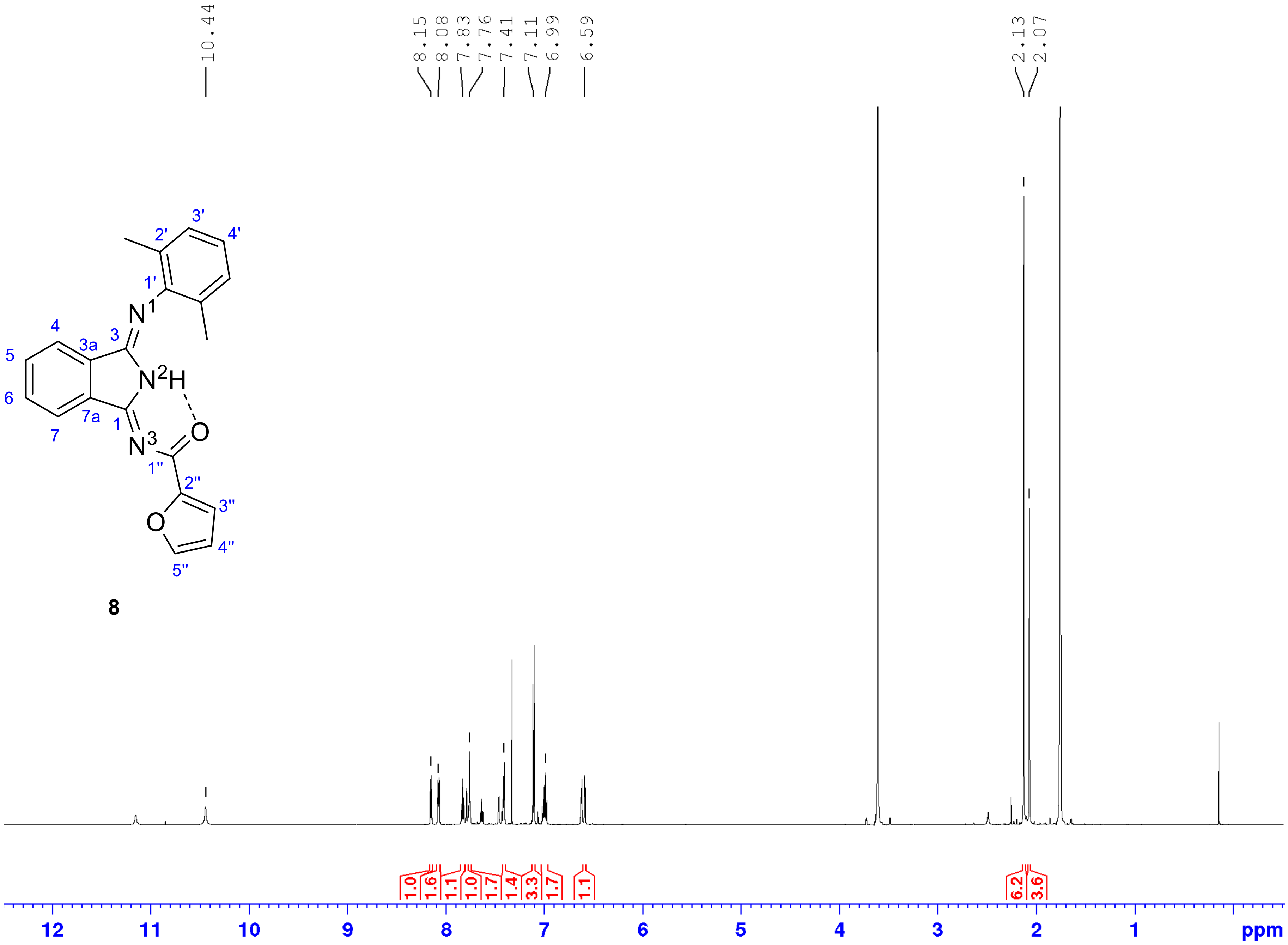

8

Figure S99. ^{1}H NMR spectrum for compound **8** in THF-d_8 at room temperature.

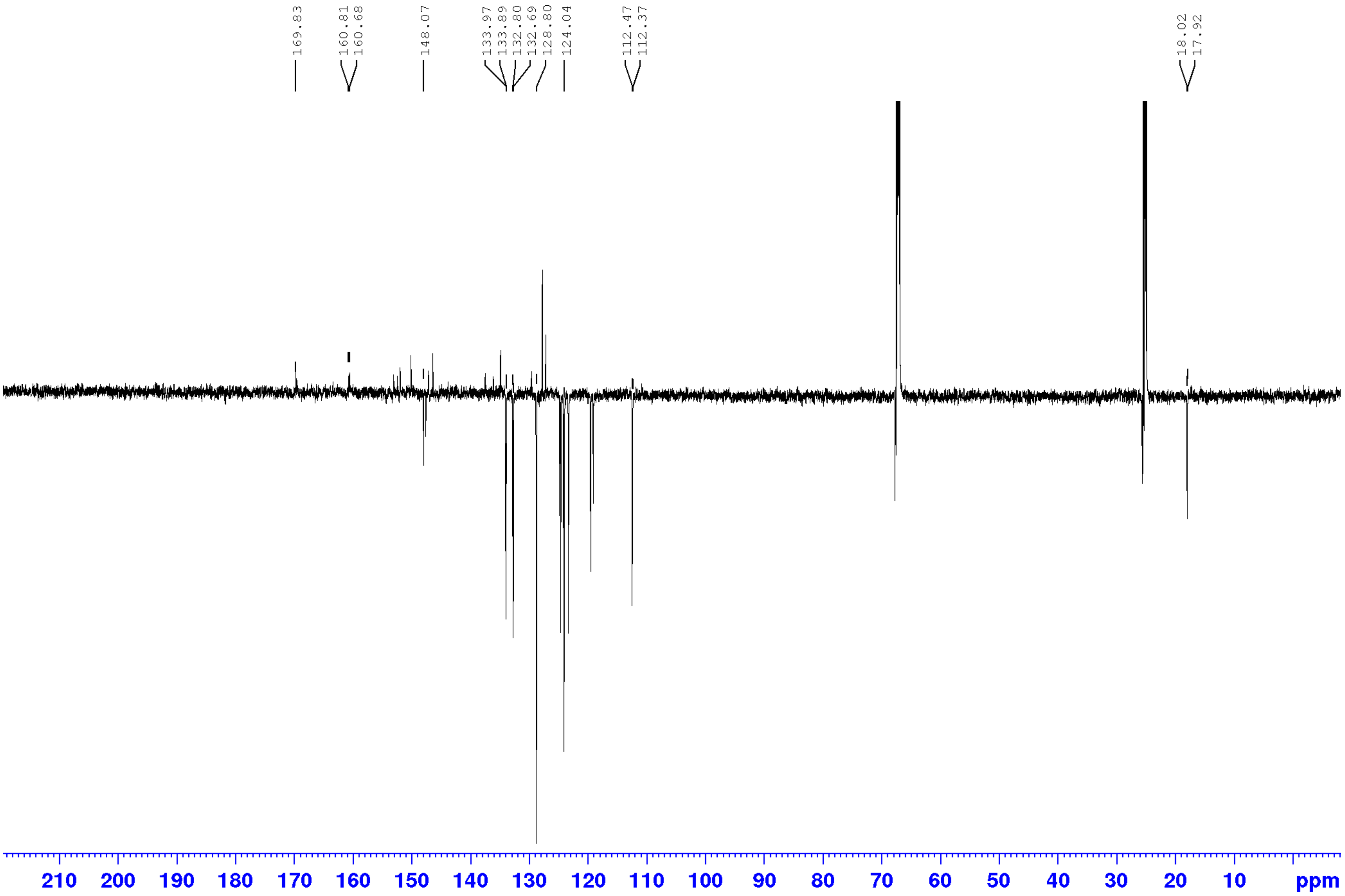

Figure S100. ^{13}C NMR spectrum for compound **8** in THF-d_8 at room temperature.

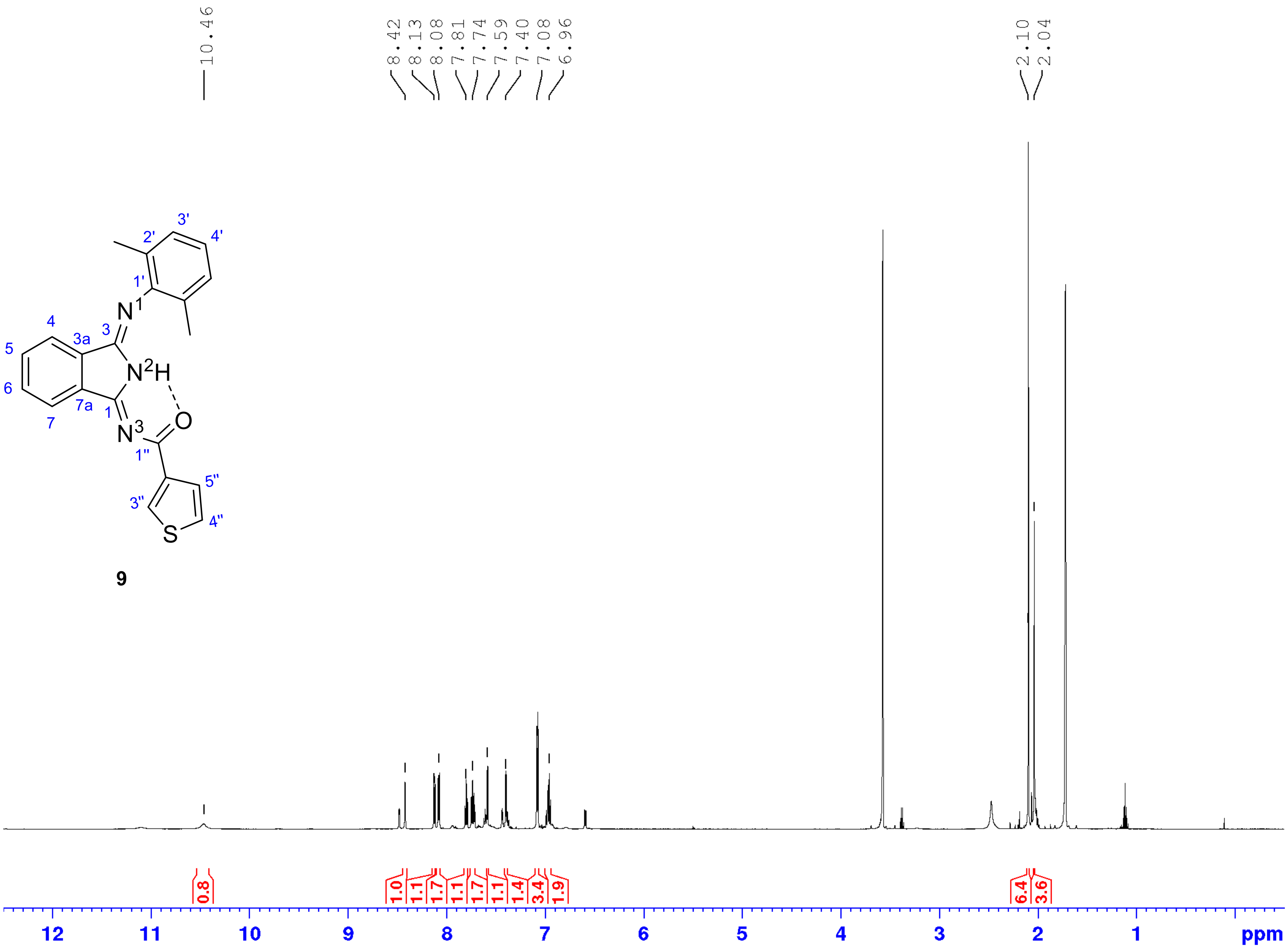

Figure S101. ^{1}H NMR spectrum for compound **9** in THF-d_8 at room temperature.

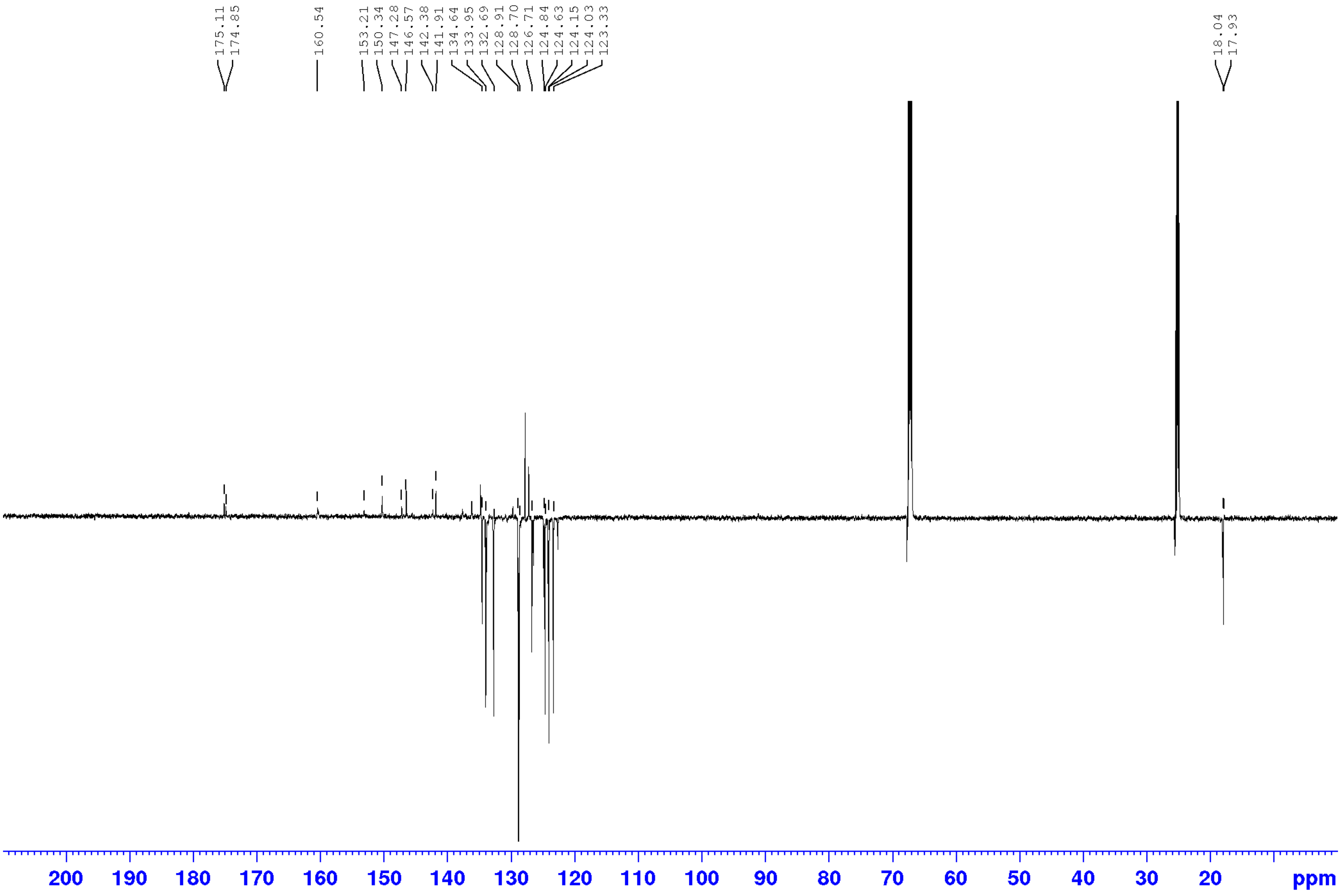

Figure S102. ^{13}C NMR spectrum for compound **9** in THF-d_8 at room temperature.

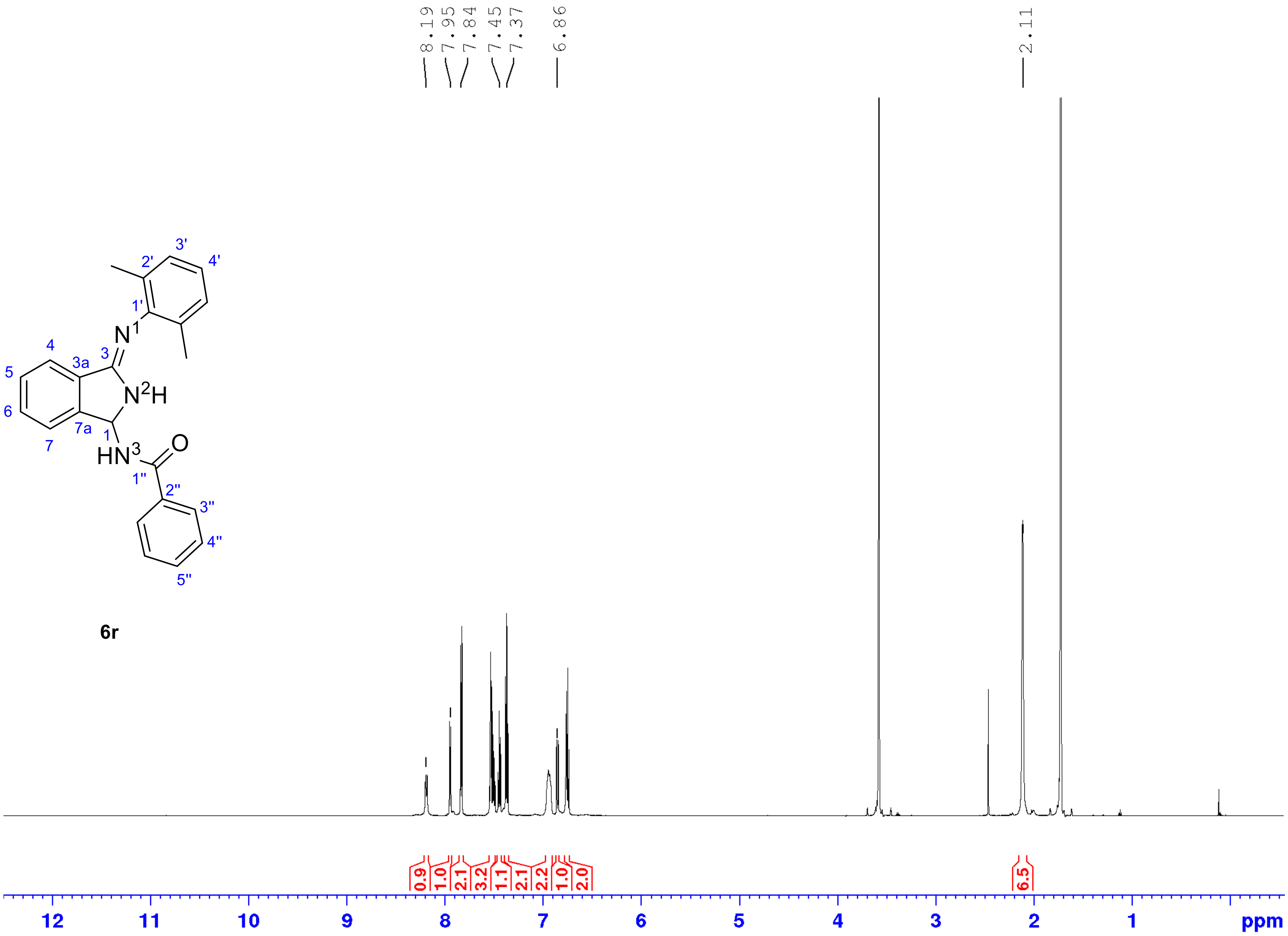

Figure S103. ^{1}H NMR spectrum for compound **6r** in THF-d_8 at room temperature.

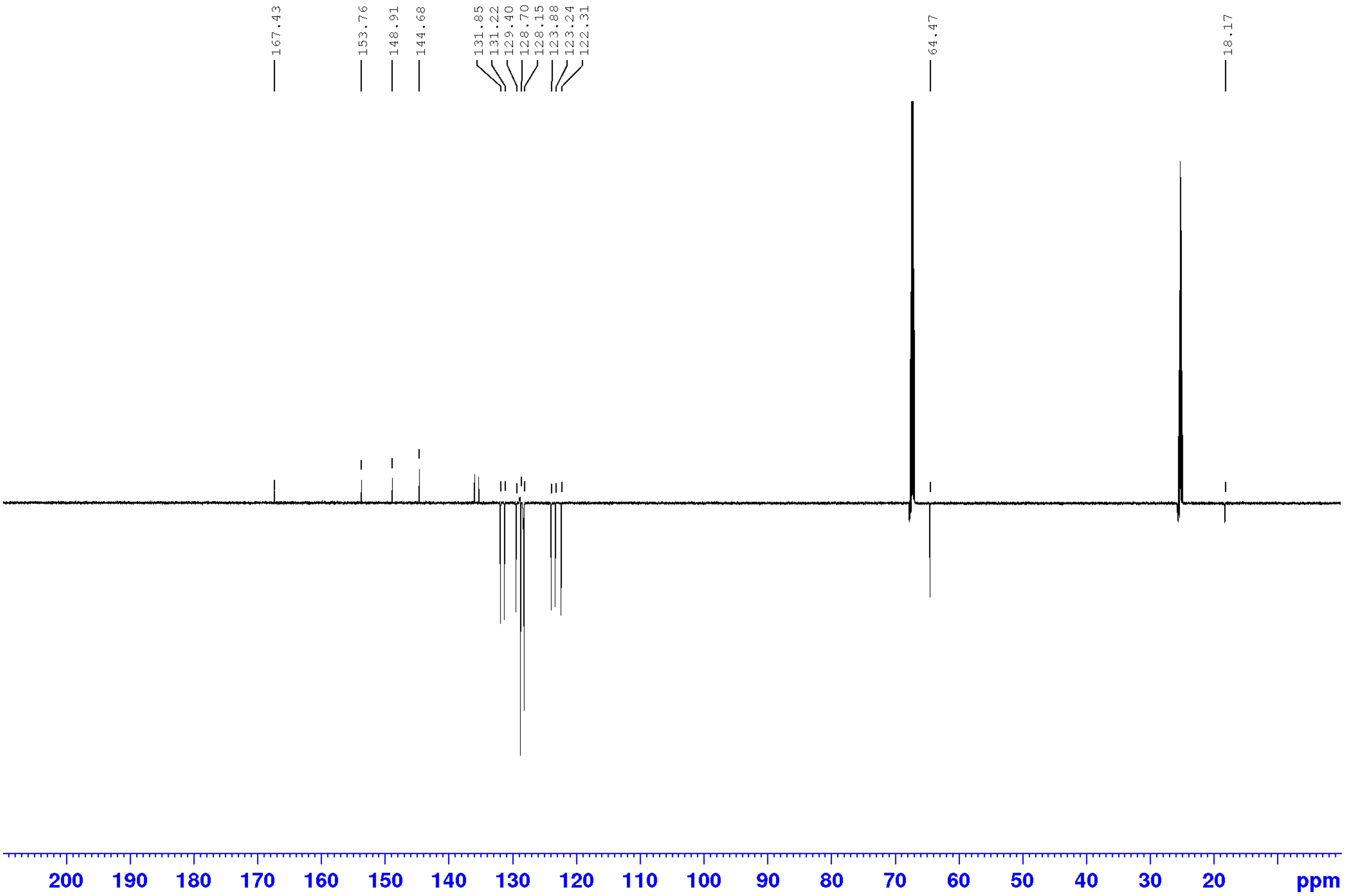

Figure S104. ^{13}C NMR spectrum for compound **6r** in THF-d_8 at room temperature.

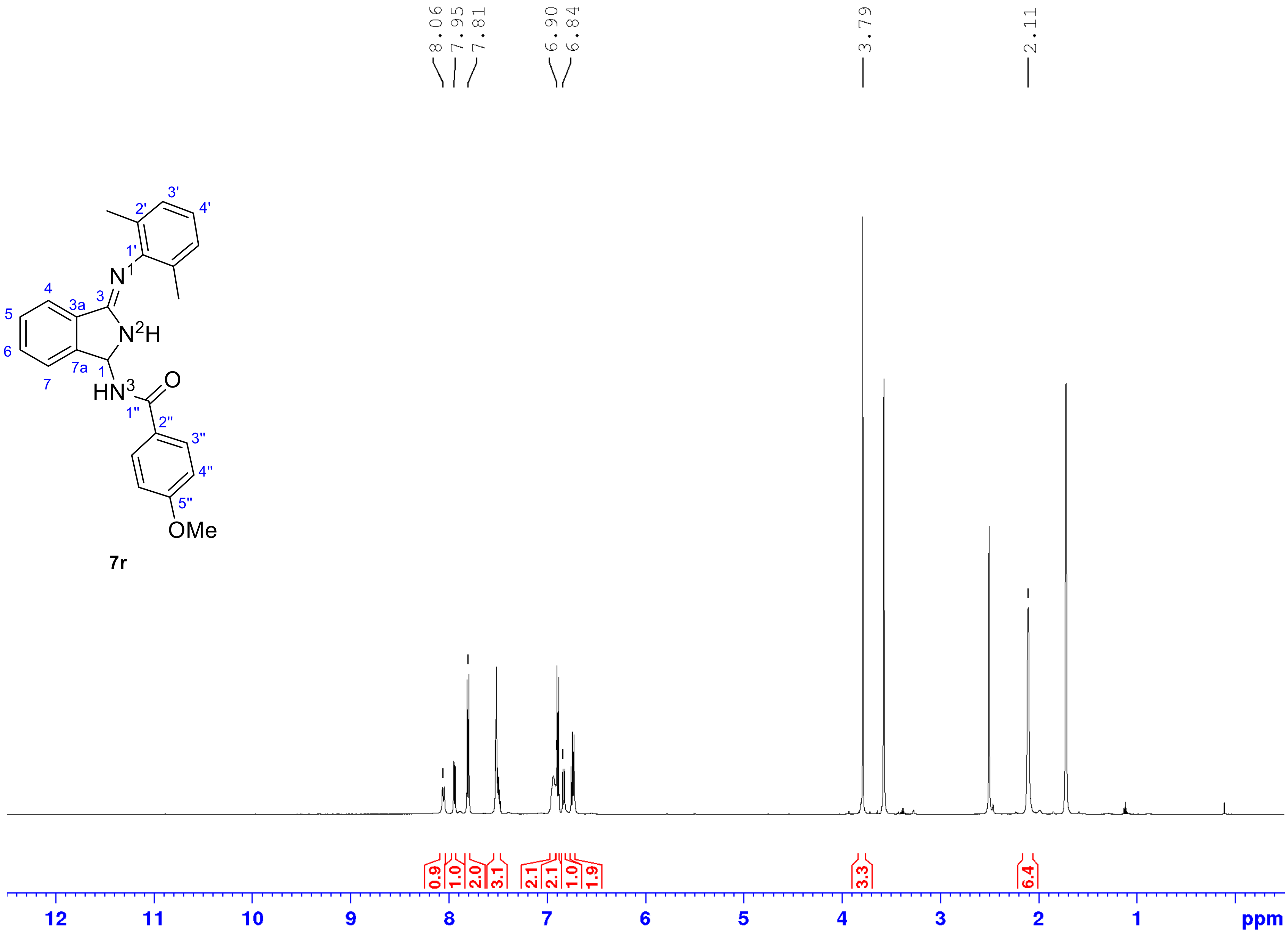

7r

Figure S105. ^{1}H NMR spectrum for compound **7r** in THF-d_8 at room temperature.

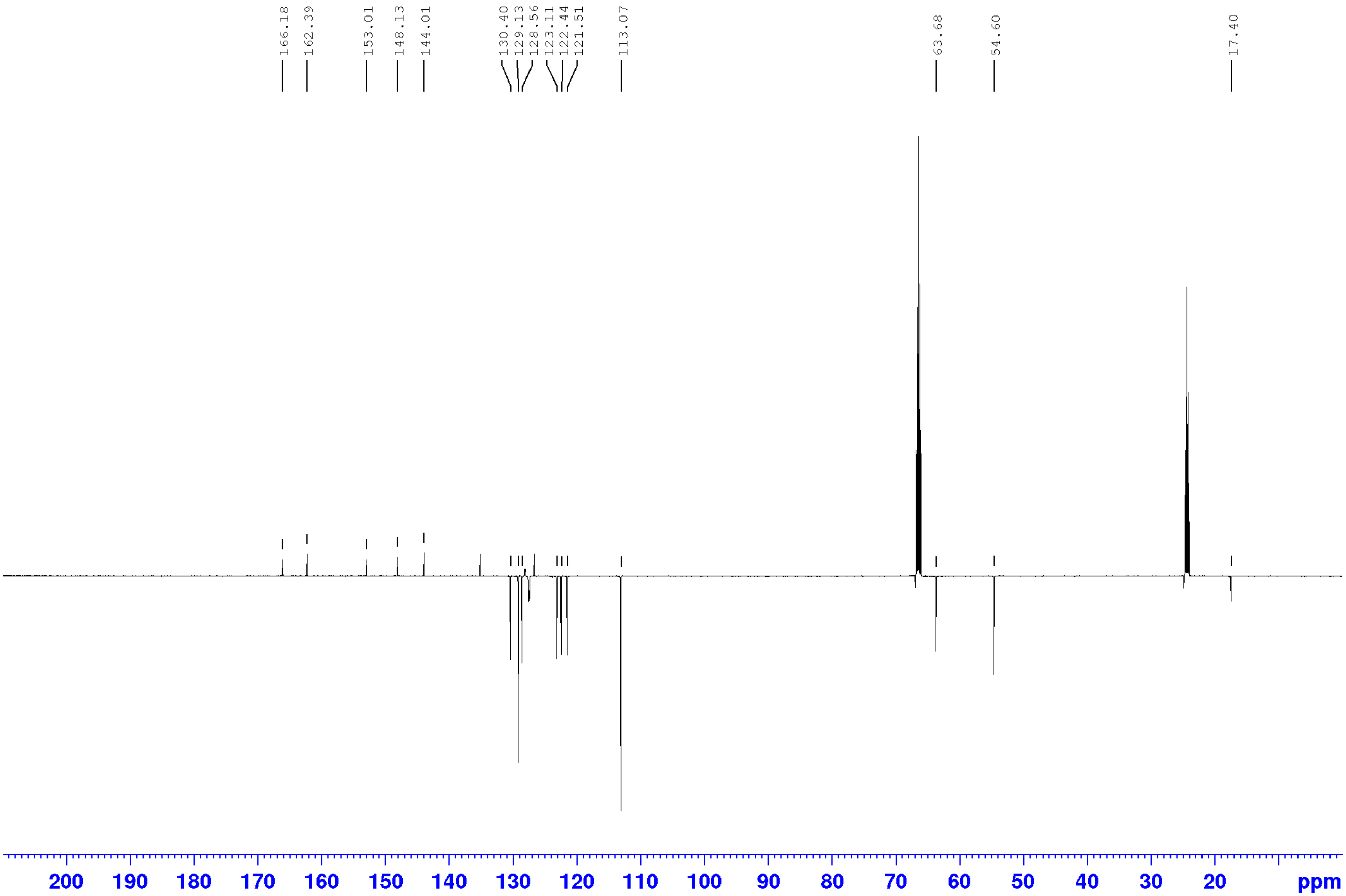

Figure S106. ^{13}C NMR spectrum for compound **7r** in THF-d_8 at room temperature.

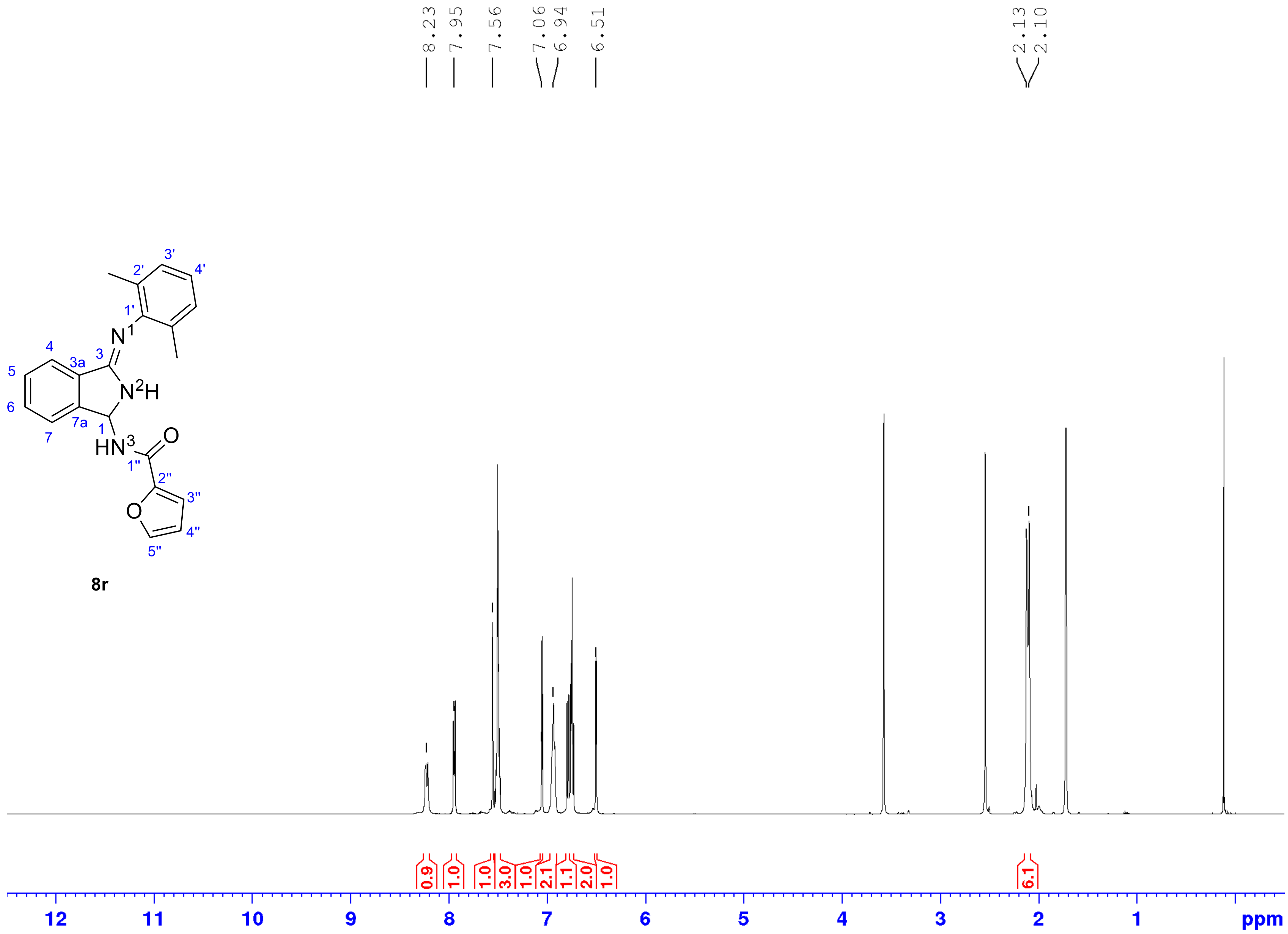

8r

Figure S107. ^{1}H NMR spectrum for compound **8r** in THF-d_8 at room temperature.

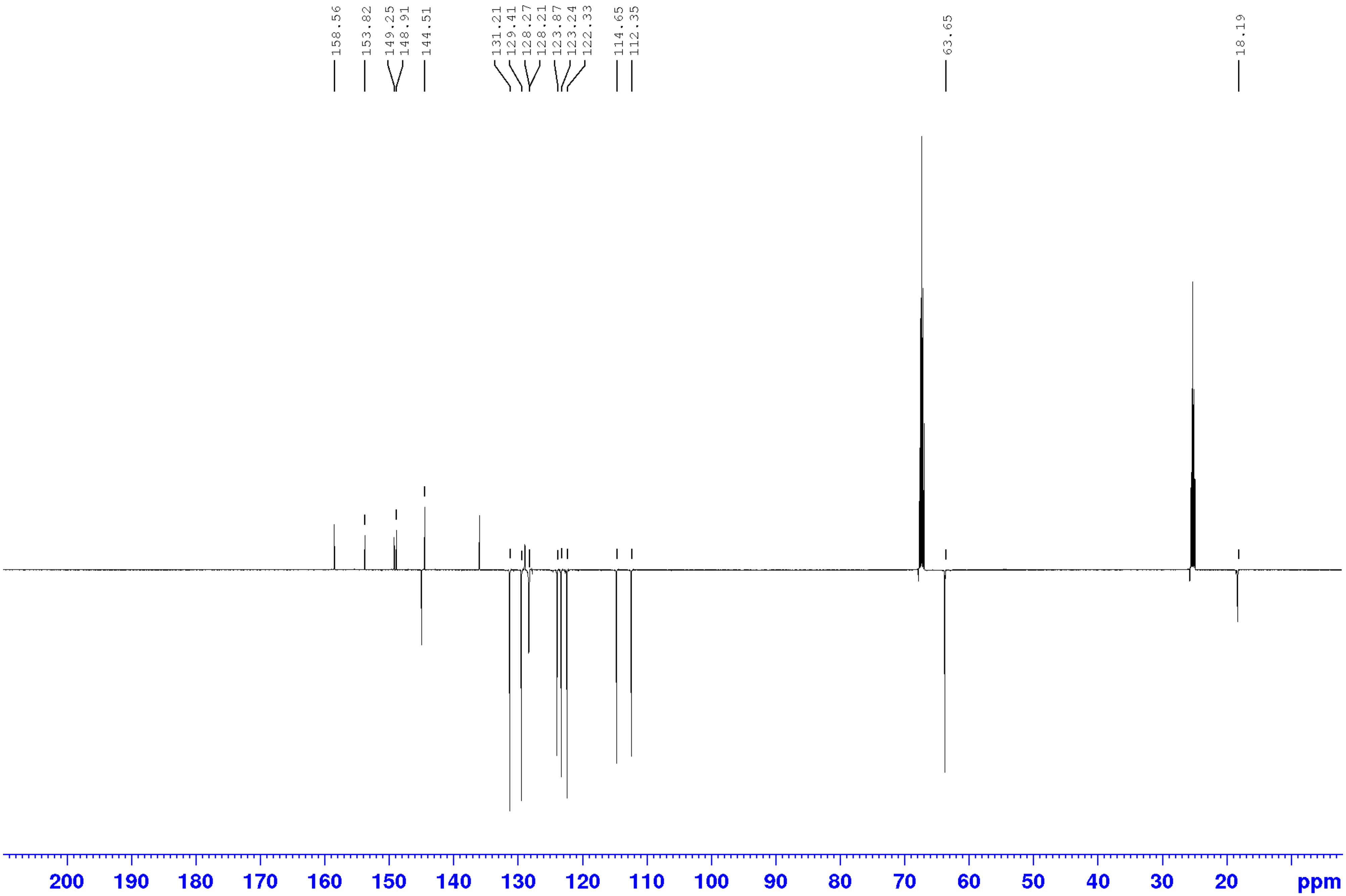

Figure S108. ^{13}C NMR spectrum for compound **8r** in THF-d_8 at room temperature.

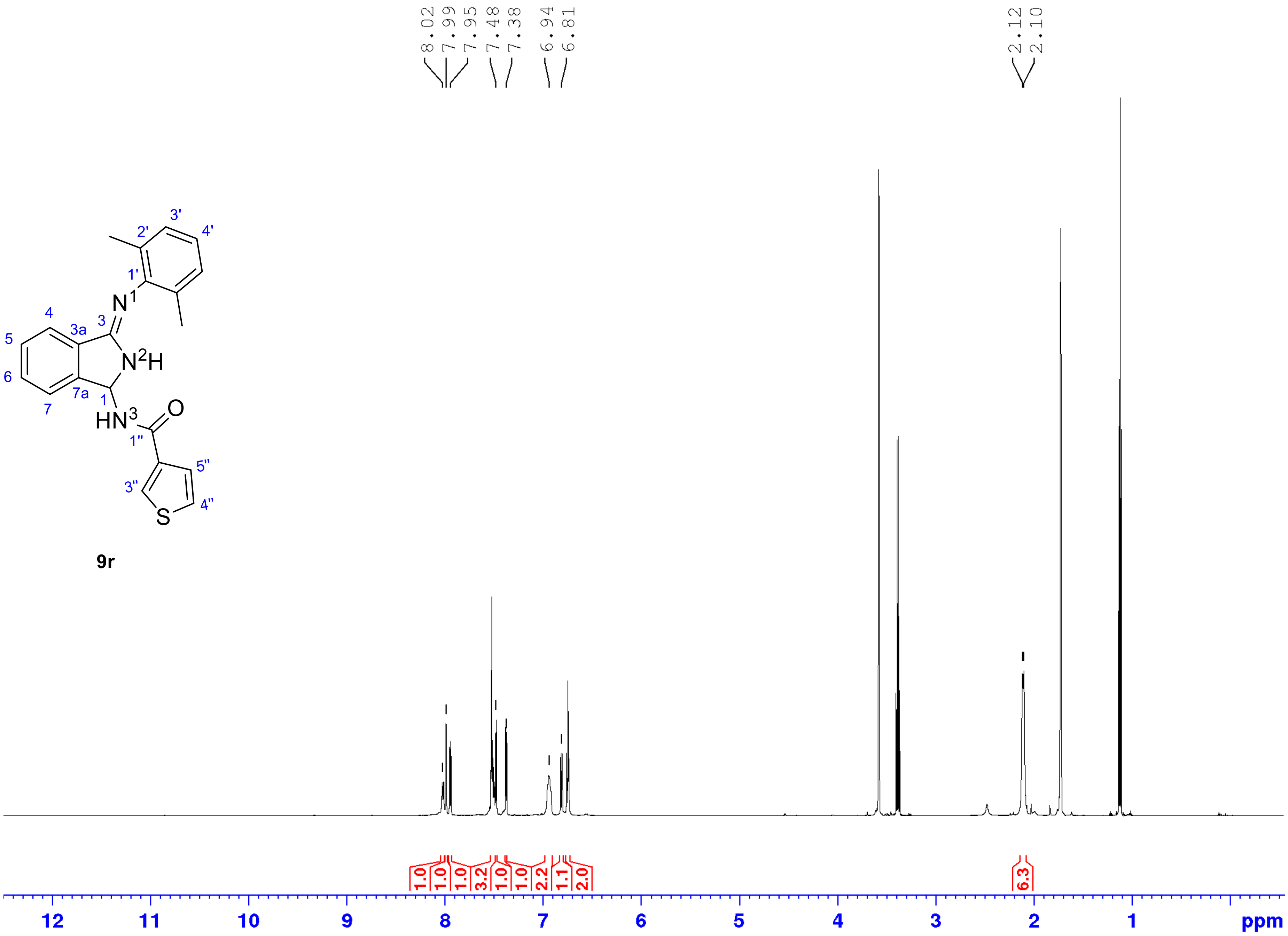

Figure S109. ^{1}H NMR spectrum for compound **9r** in THF-d_8 at room temperature.

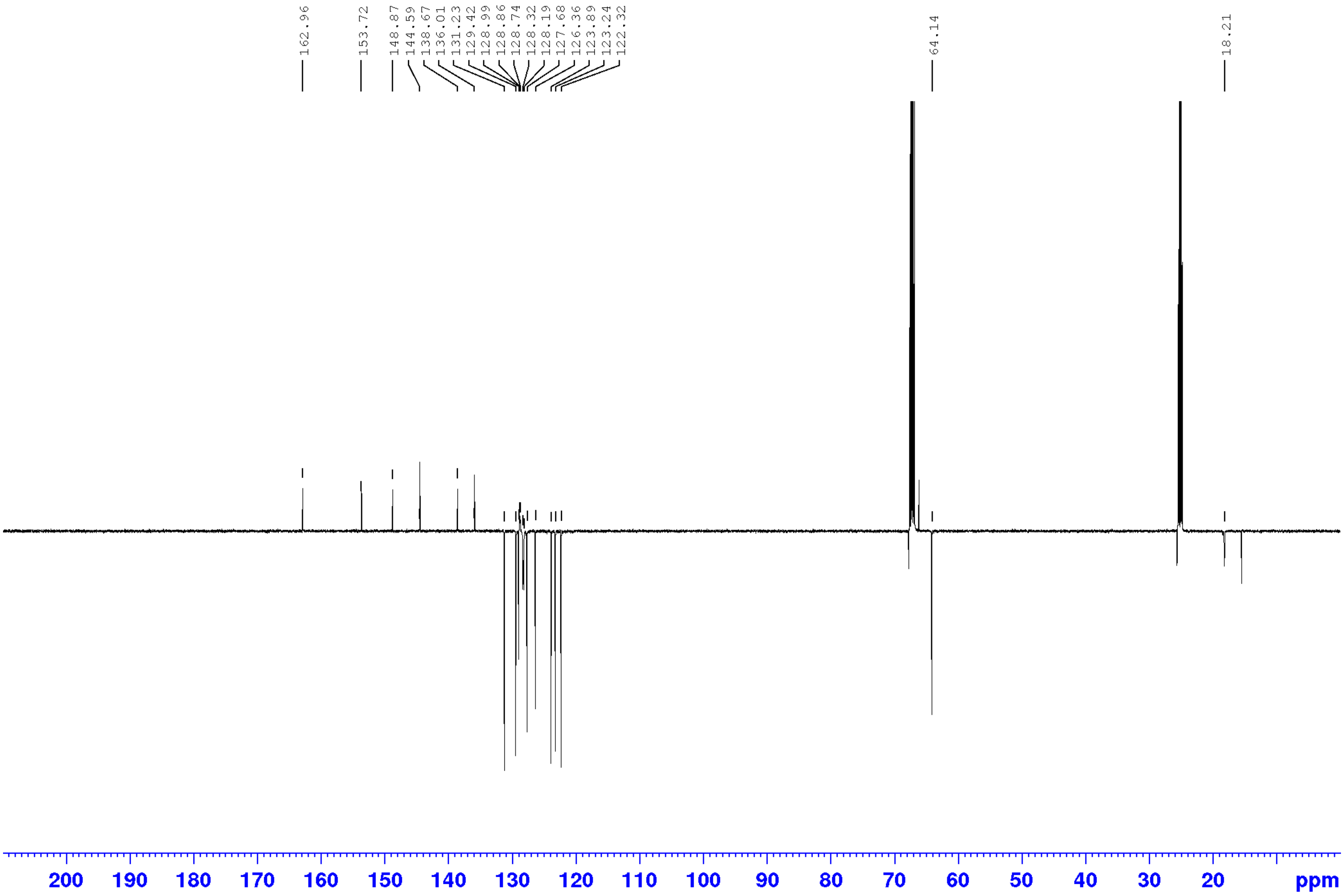

Figure S110. ^{13}C NMR spectrum for compound **9r** in THF-d_8 at room temperature.

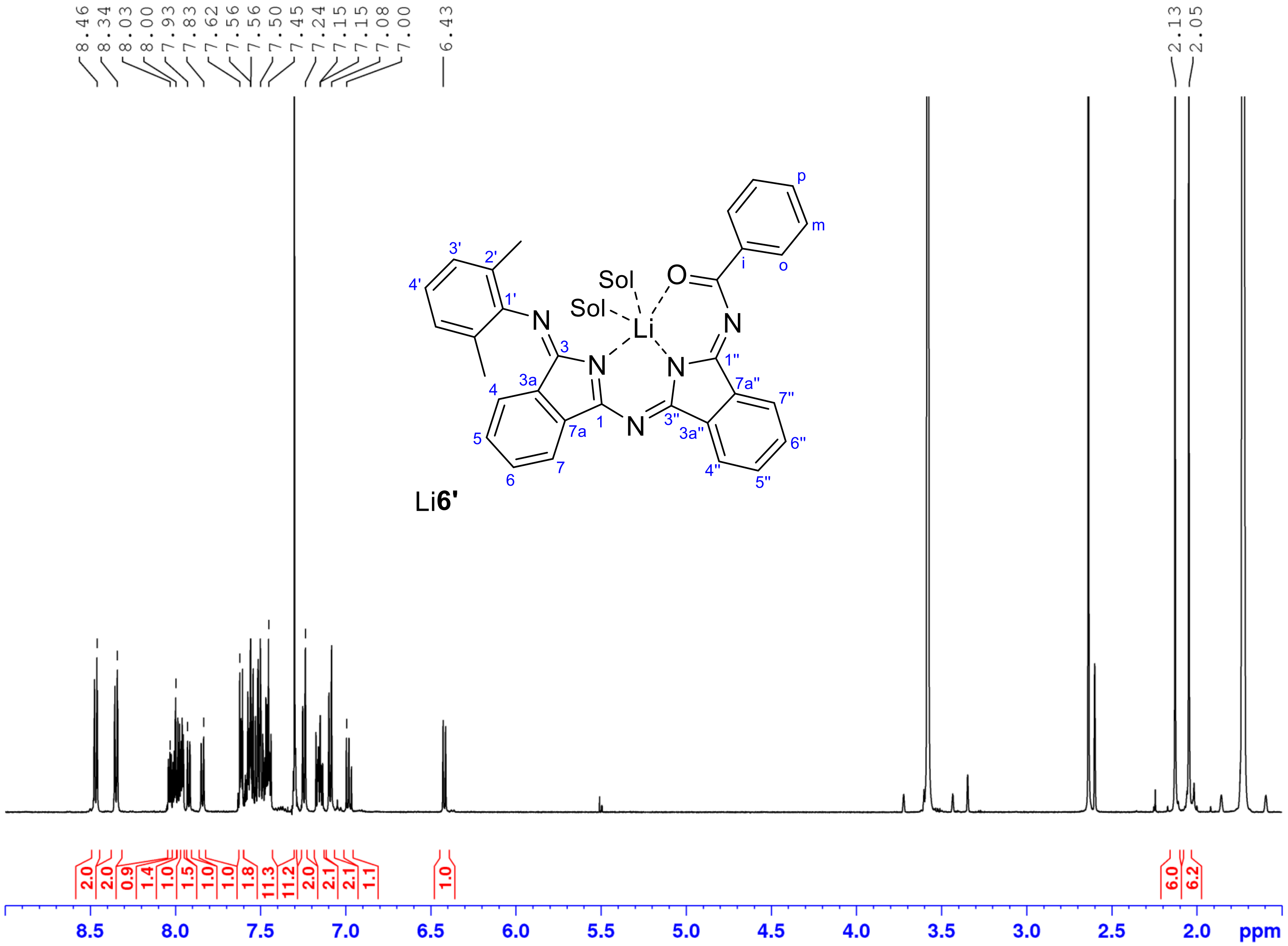

Figure S111. ^{1}H NMR spectrum for compound Li**6'** in THF-d_8 at room temperature.

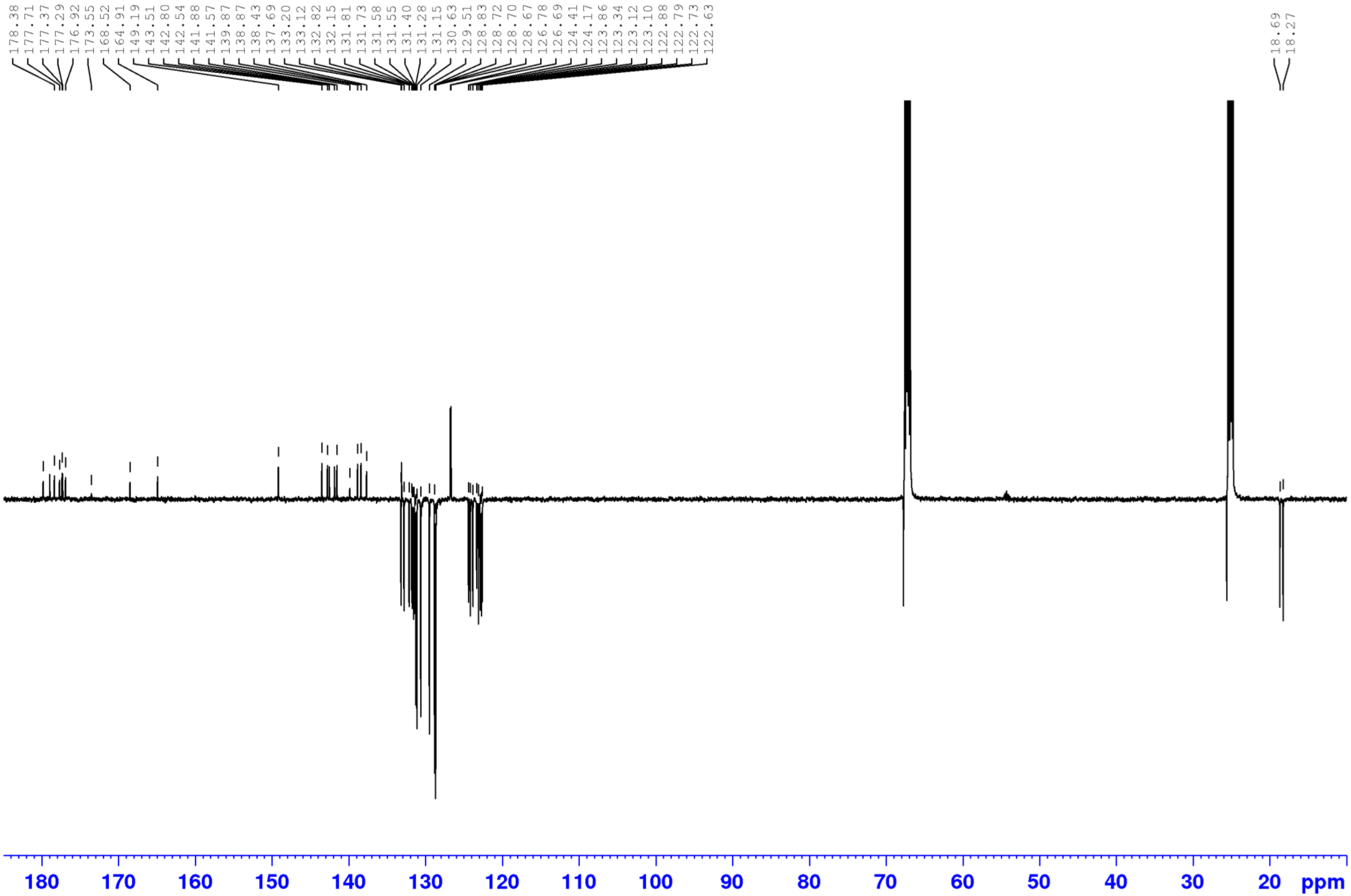

Figure S112. ^{13}C NMR spectrum for compound Li**6'** in THF-d_8 at room temperature.

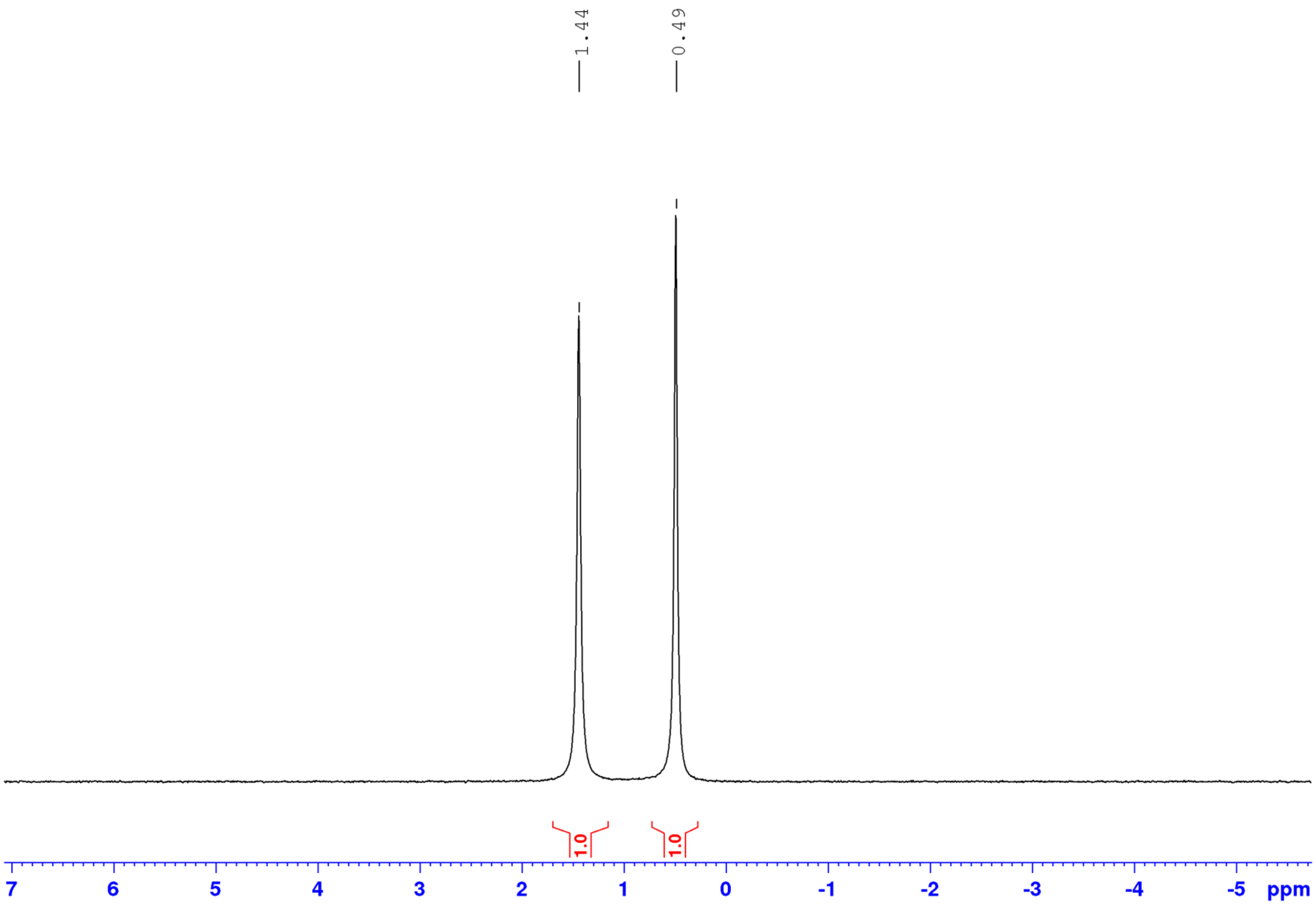

Figure S113. ^{7}Li NMR spectrum for compound Li**6'** in THF-d_8 at room temperature.

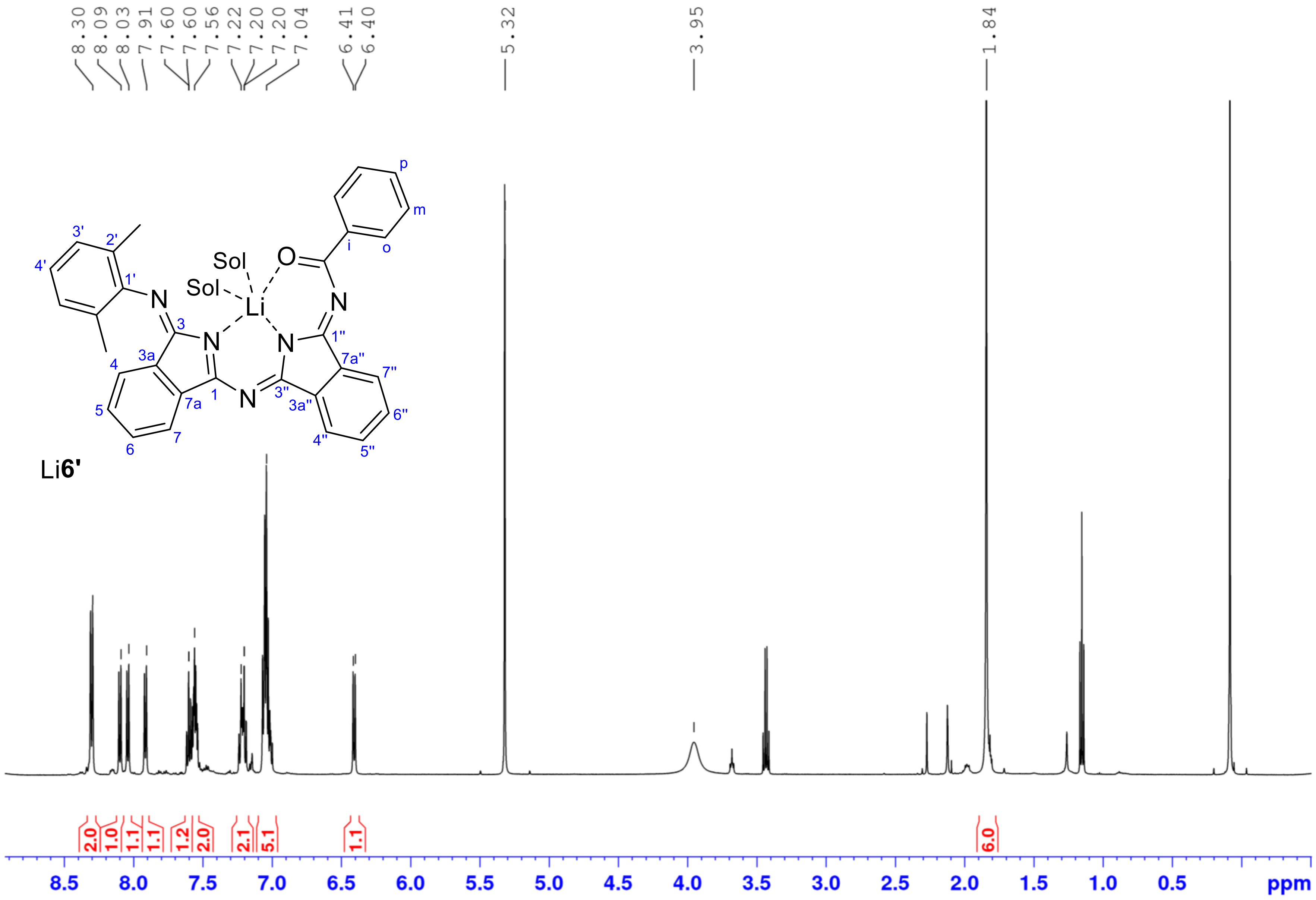

Figure S114. ^{1}H NMR spectrum for compound Li**6'** in CD_2Cl_2 at room temperature.

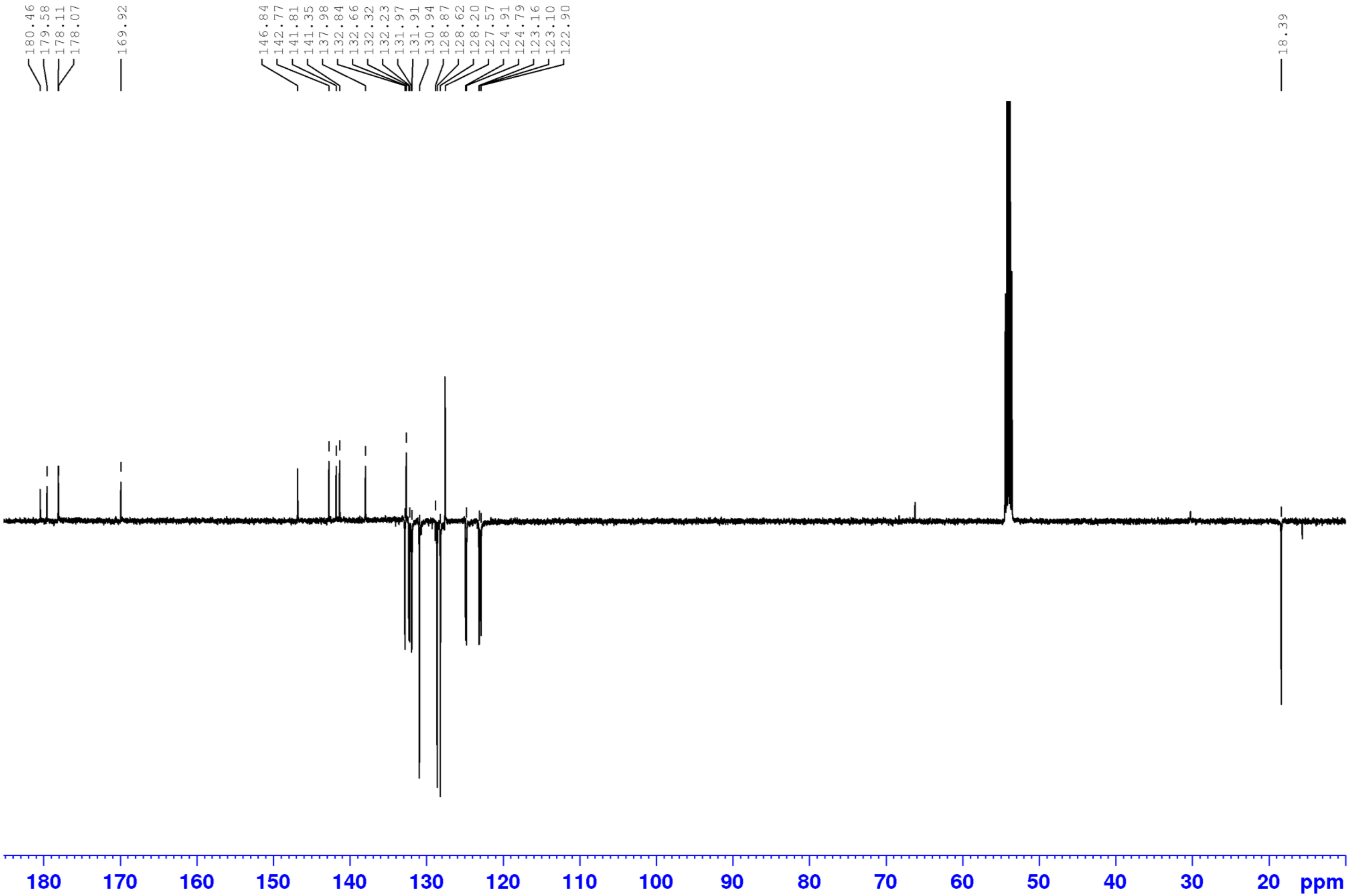

Figure S115. ^{13}C NMR spectrum for compound Li**6'** in CD_2Cl_2 at room temperature.

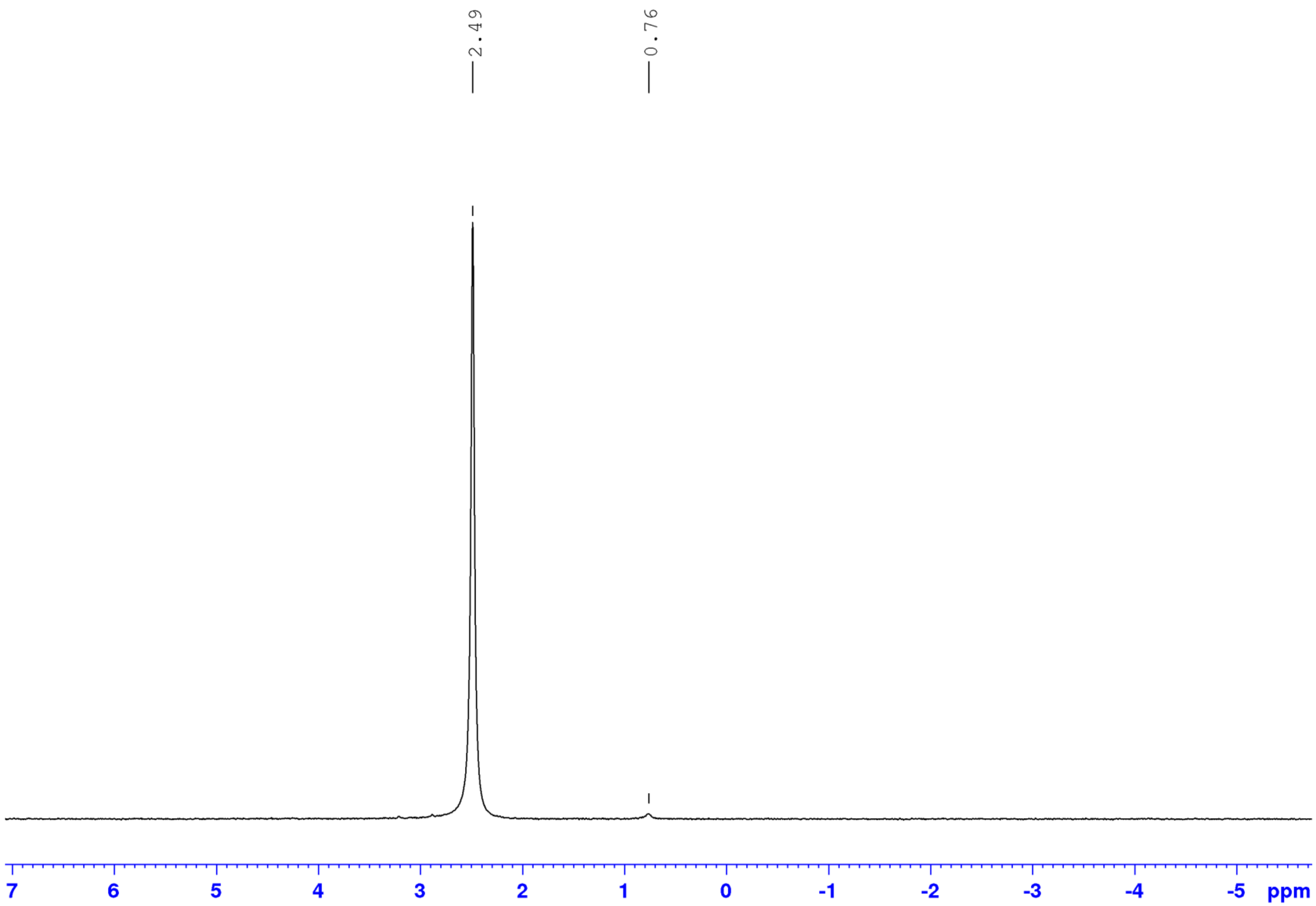

Figure S116. ^{7}Li NMR spectrum for compound Li**6'** in CD_2Cl_2 at room temperature.

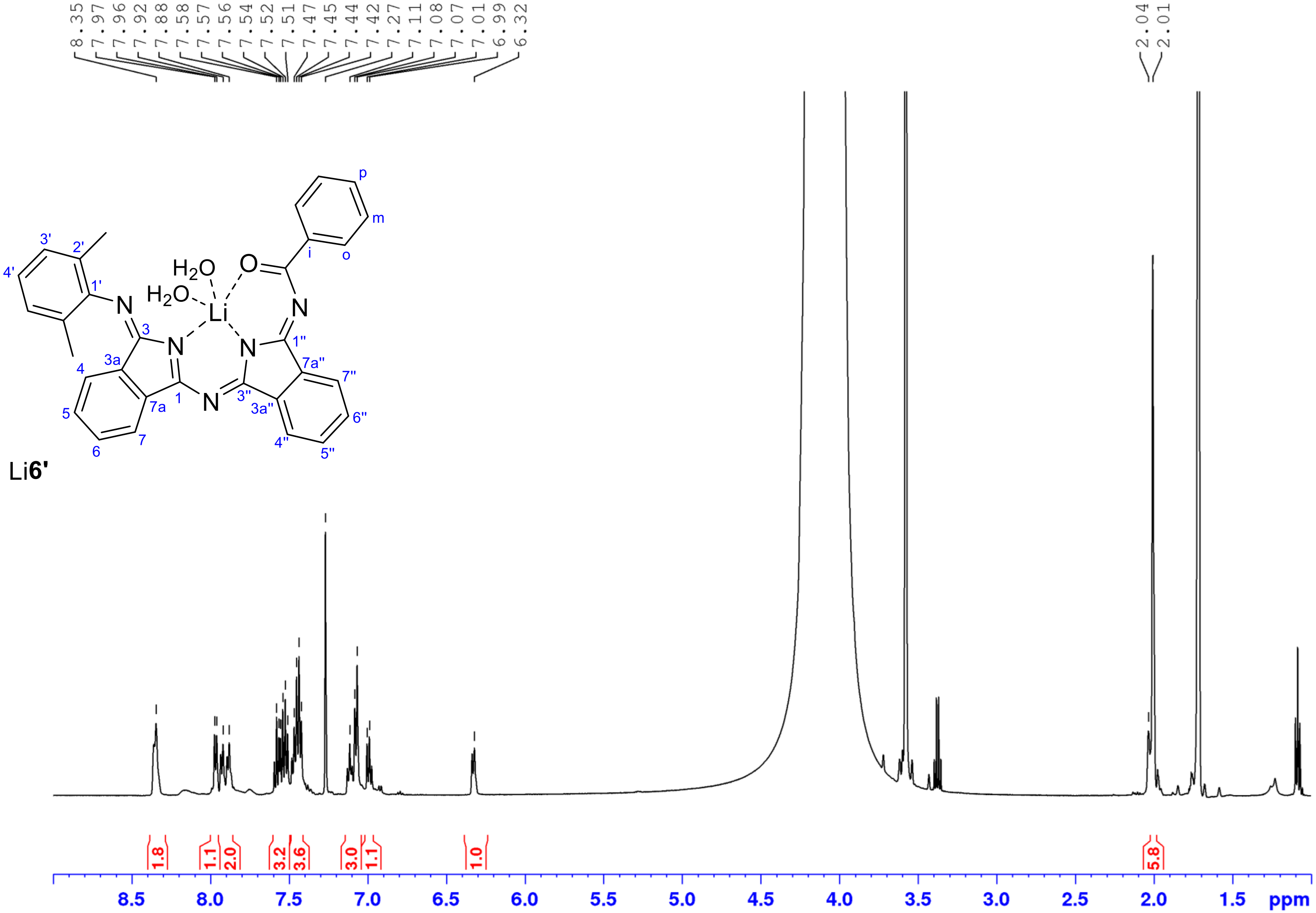

Figure S117. ^{1}H NMR spectrum for compound **Li6'** + 80 µl H_2O in THF-d_8 at room temperature.

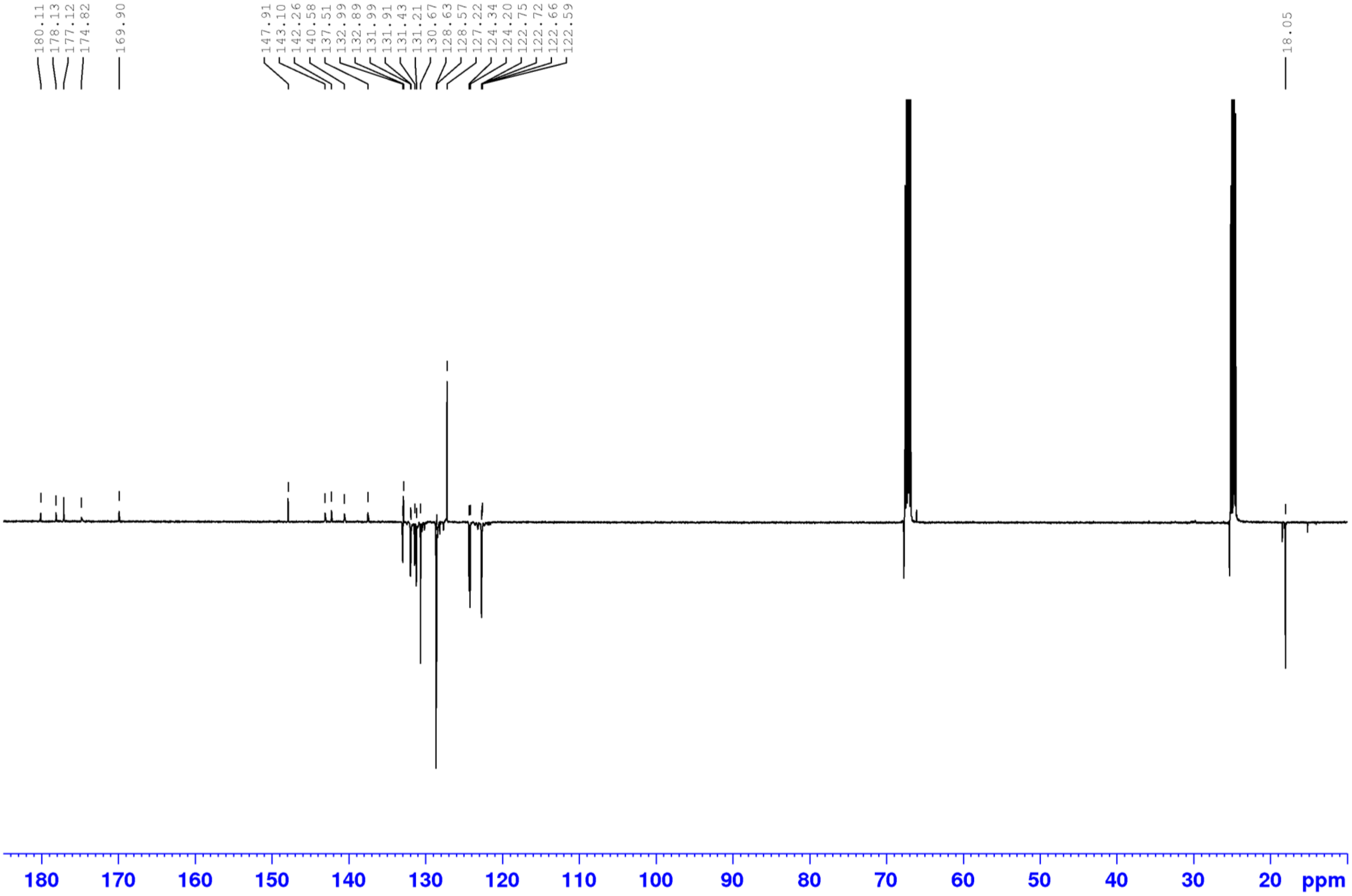

Figure S118. ^{13}C NMR spectrum for compound Li**6‘** + 80 µl H_2O in THF-d_8 at room temperature.

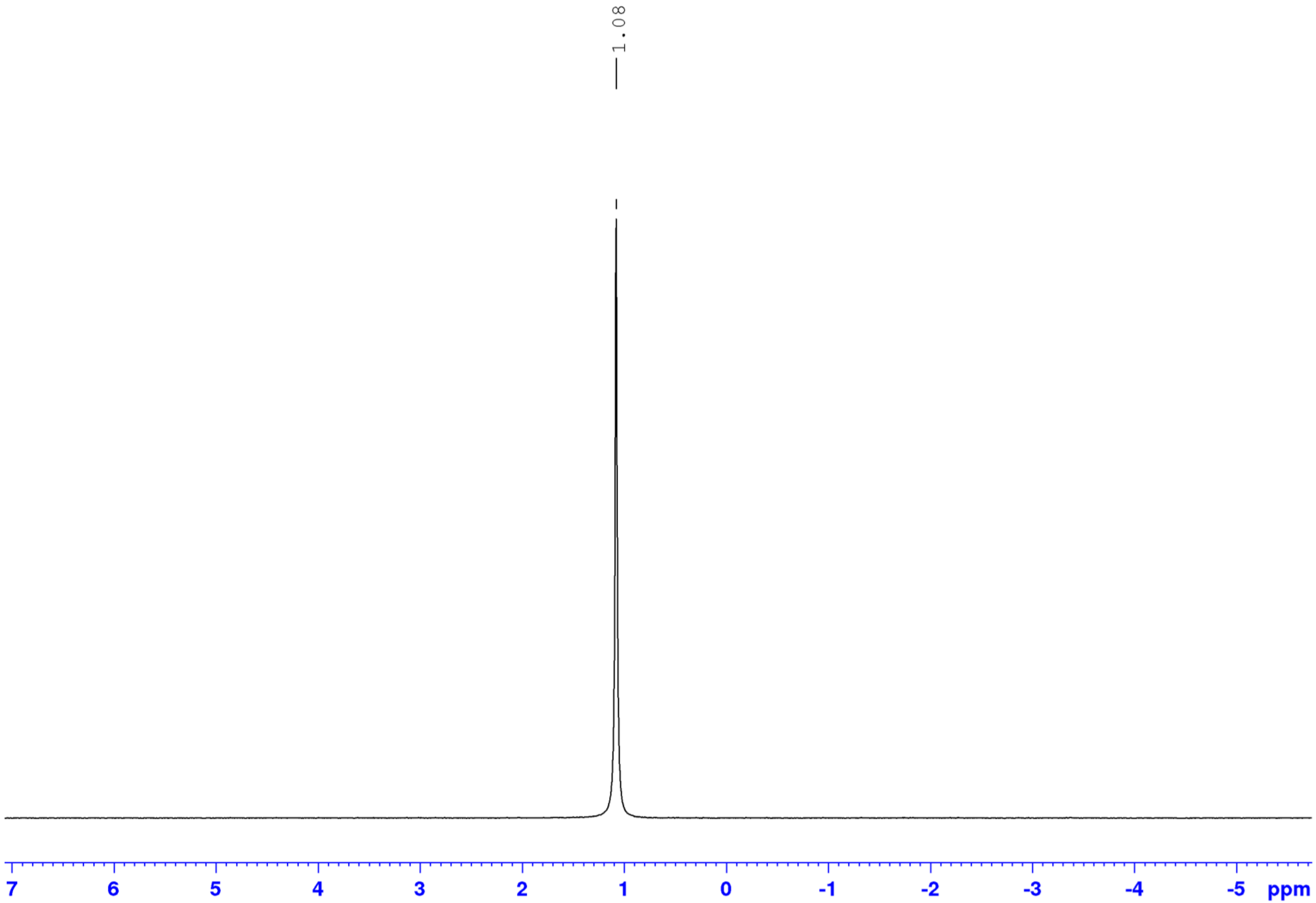

Figure S119. ^{7}Li NMR spectrum for compound Li**6'** + 80 µl H_2O in THF-d_8 at room temperature.

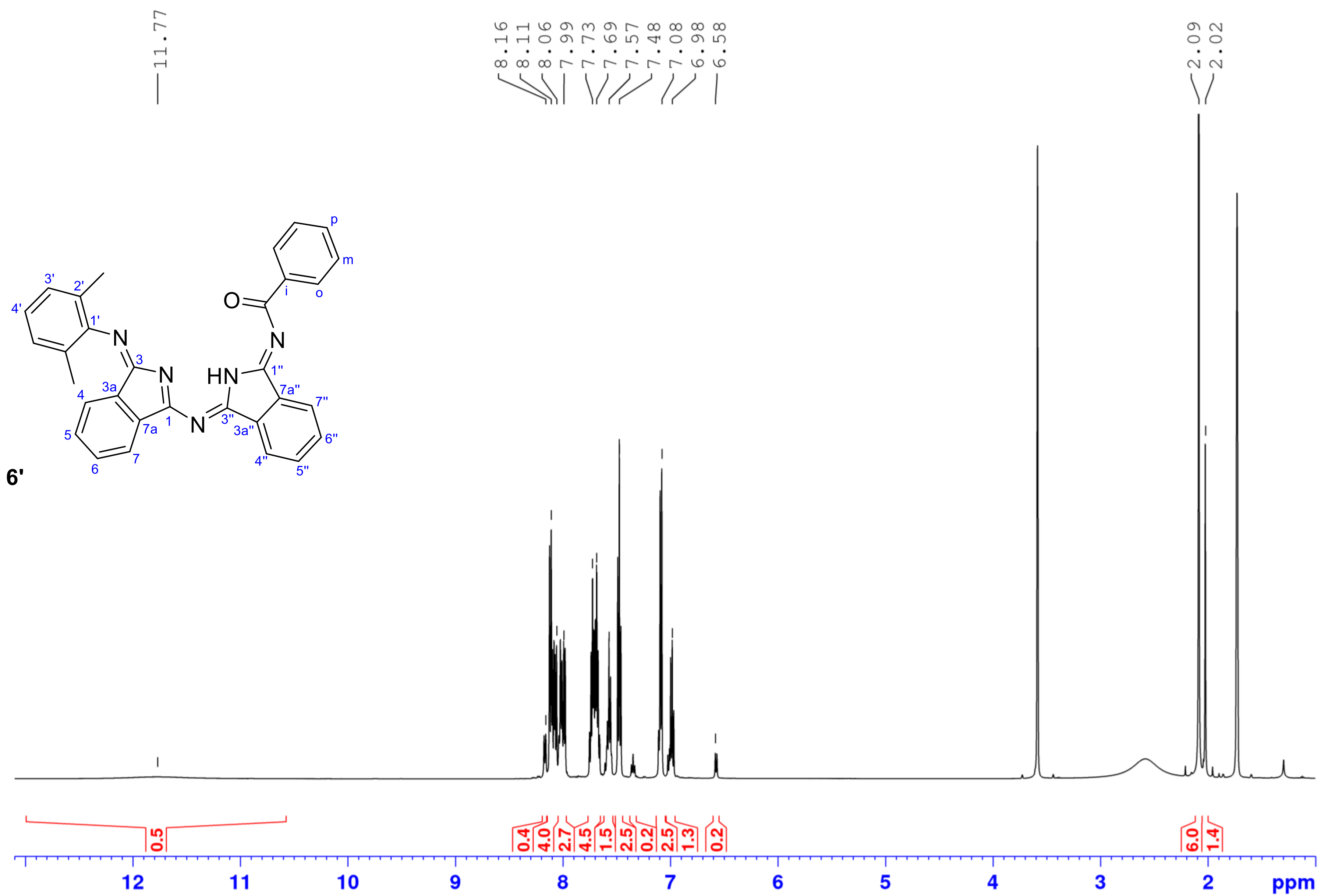

Figure S120. ^{1}H NMR spectrum for compound **6'** in THF-d_8 at room temperature.

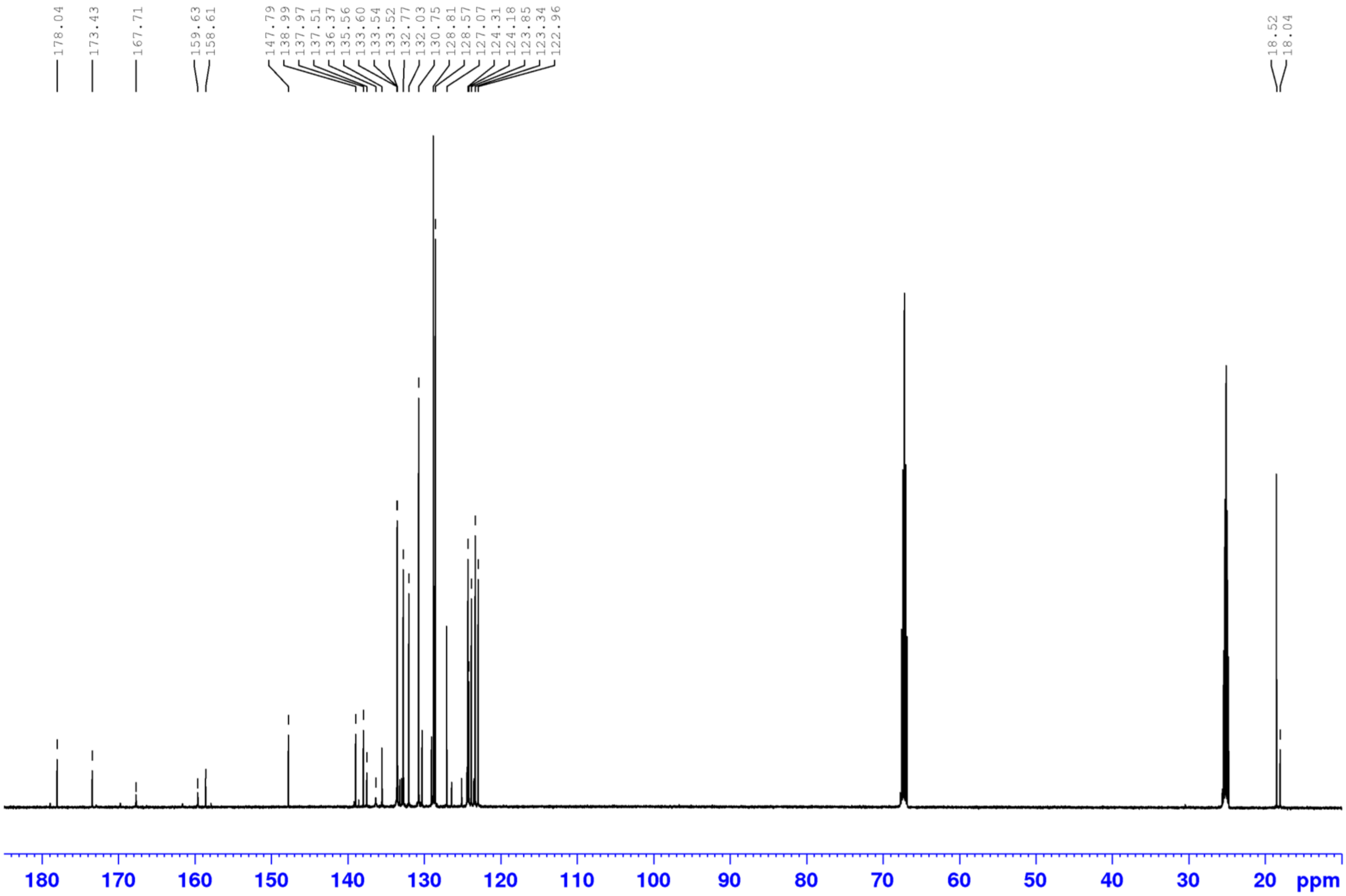

Figure S121. ^{13}C NMR spectrum for compound **6'** in THF-d_8 at room temperature.

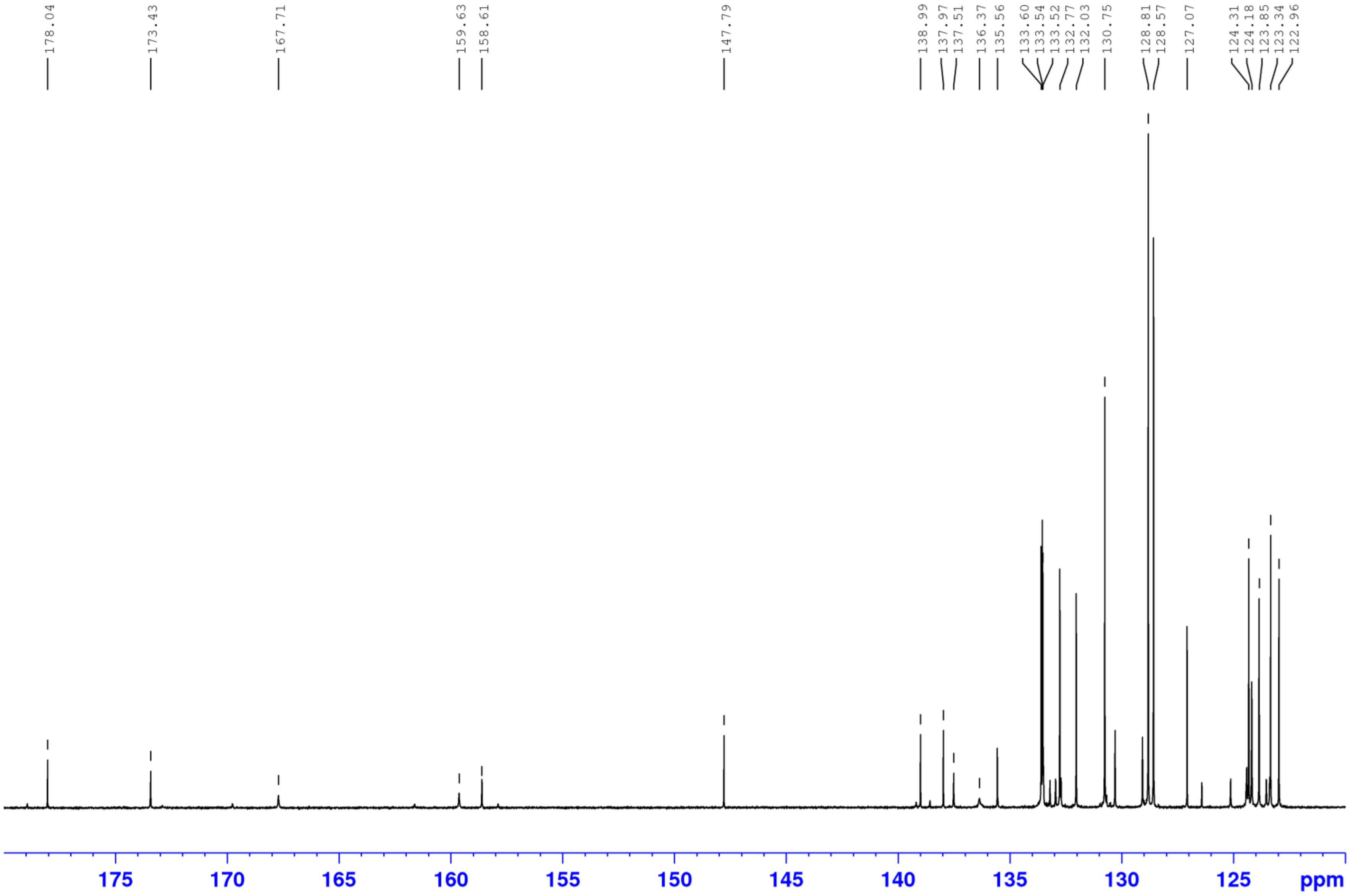

Figure S122. ^{13}C NMR spectrum for compound **6'** in THF-d_8 at room temperature (detail).

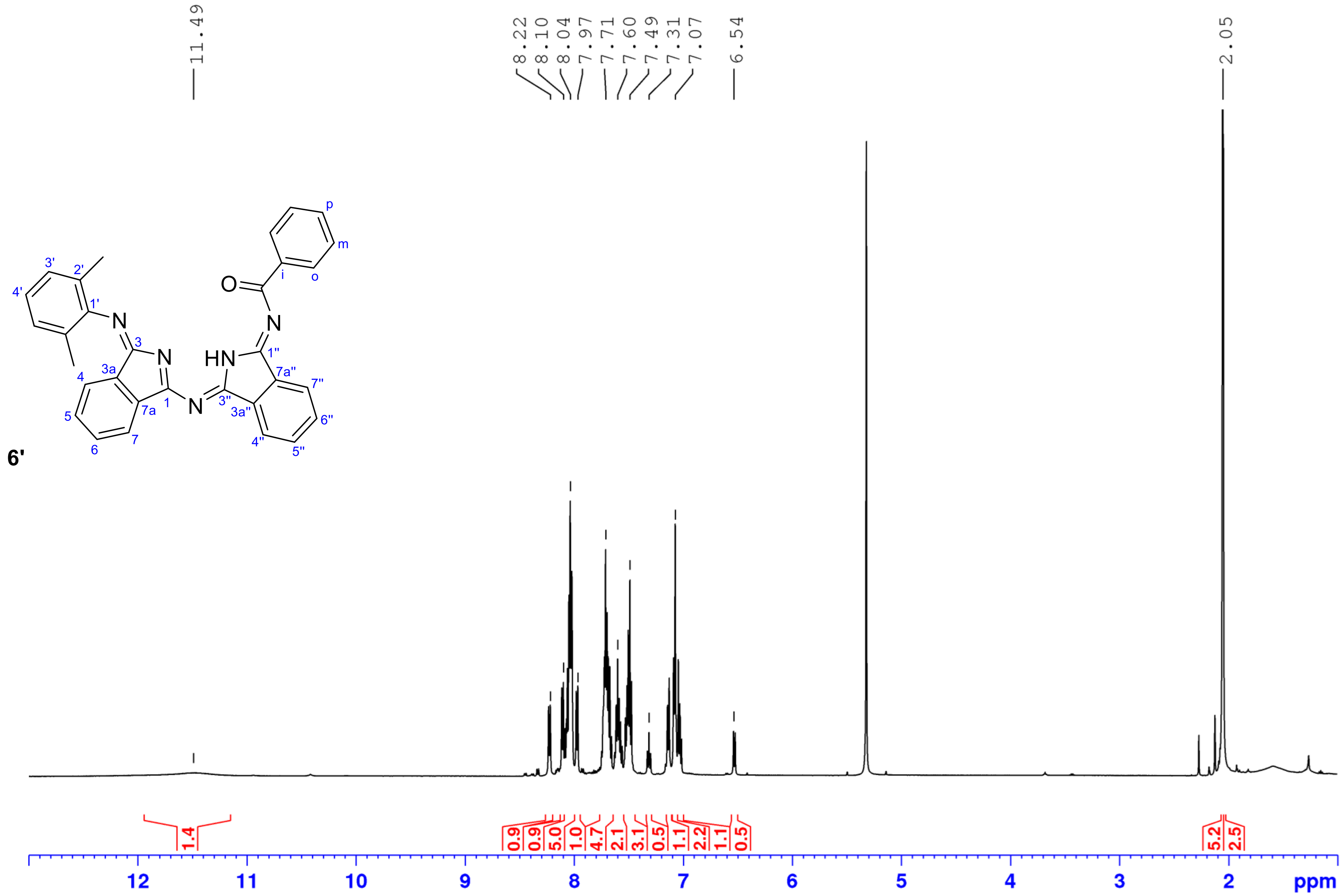

Figure S123. ^{1}H NMR spectrum for compound **6'** in CD_2Cl_2 at room temperature.

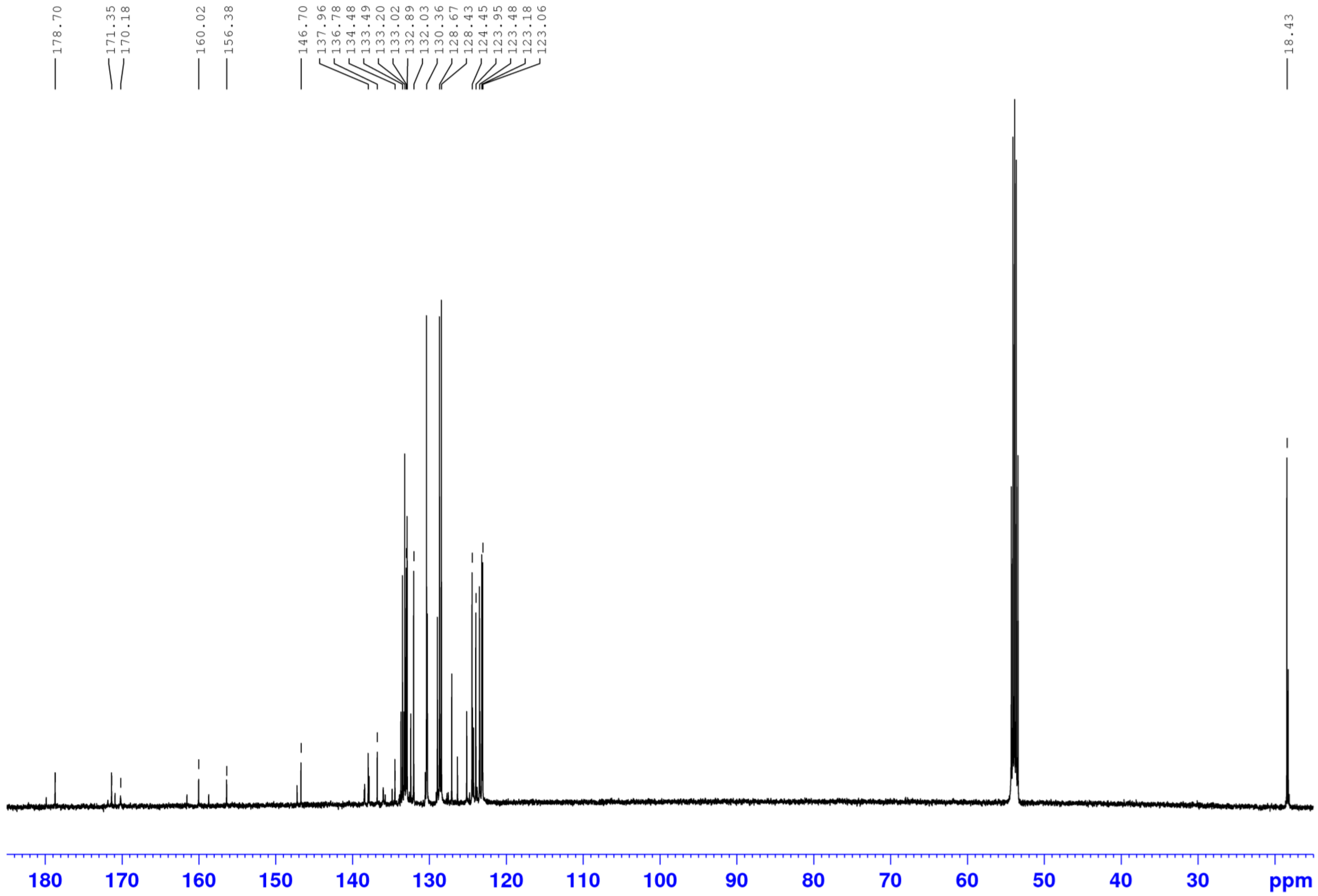

Figure S124. ^{13}C NMR spectrum for compound **6'** in CD_2Cl_2 at room temperature.

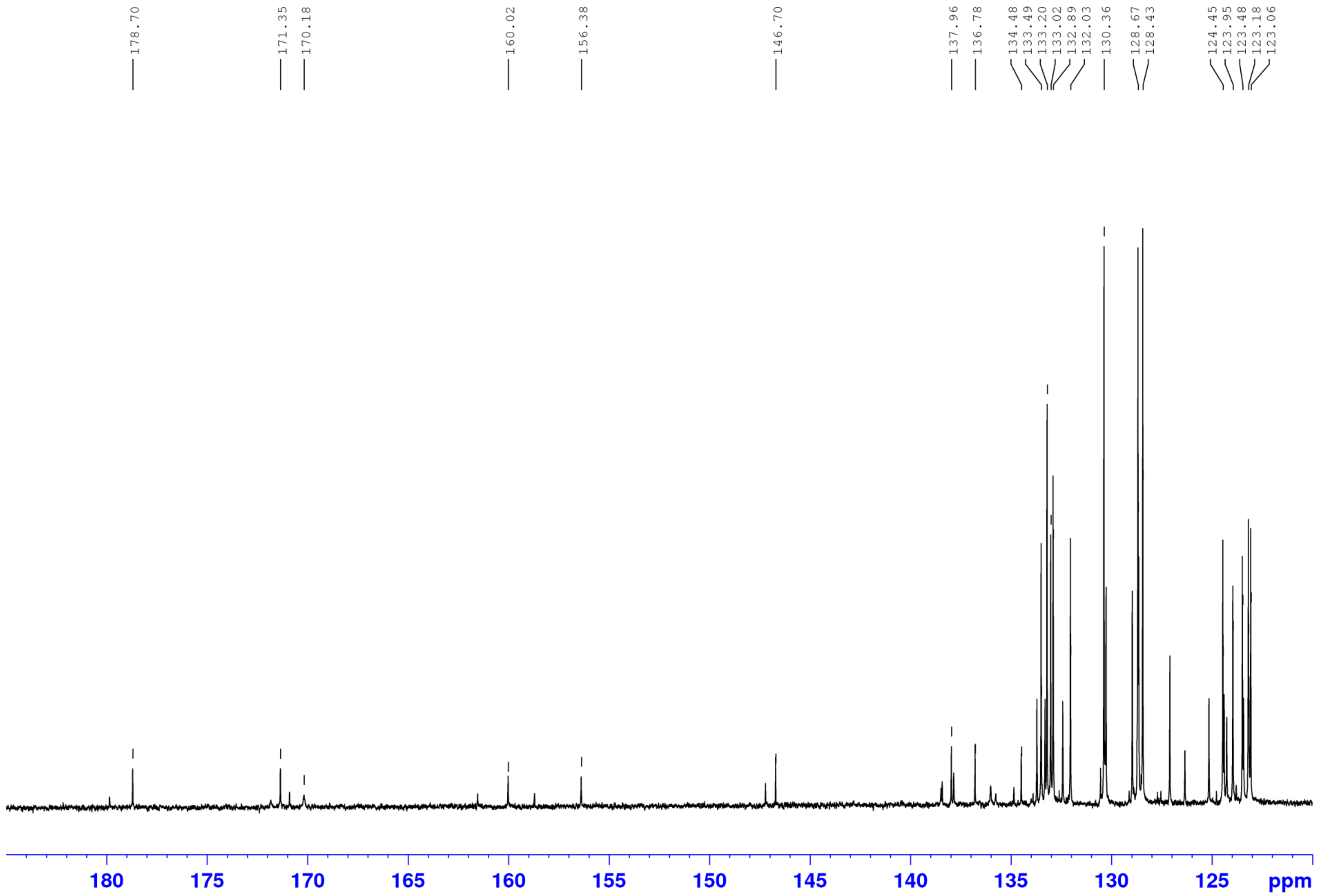

Figure S125. ^{13}C NMR spectrum for compound **6‘** in CD_2Cl_2 at room temperature (detail).

9 IR spectra of prepared compounds

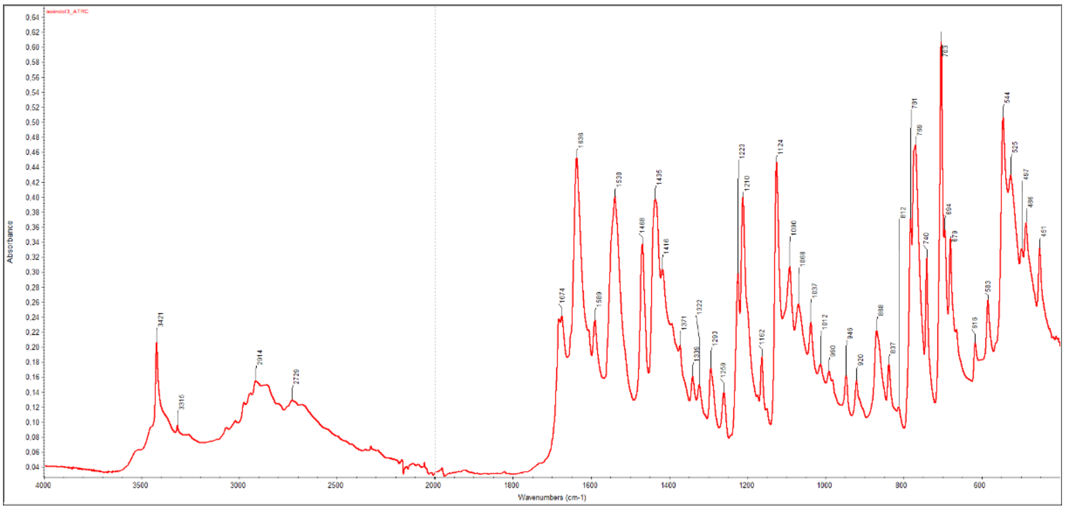

Figure S126. IR spectrum for compound **1a** measured in solid state**.**

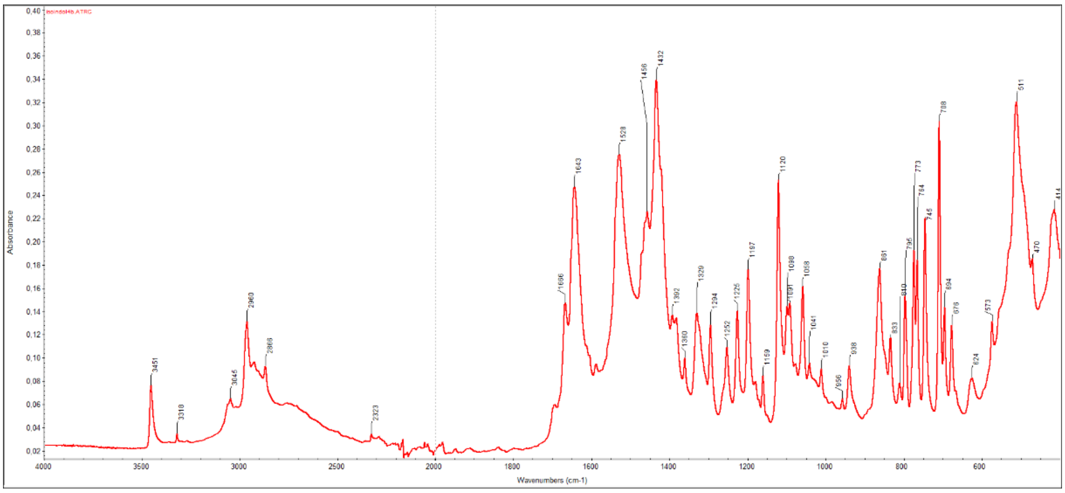

Figure S127. IR spectrum for compound **1b** measured in solid state**.**

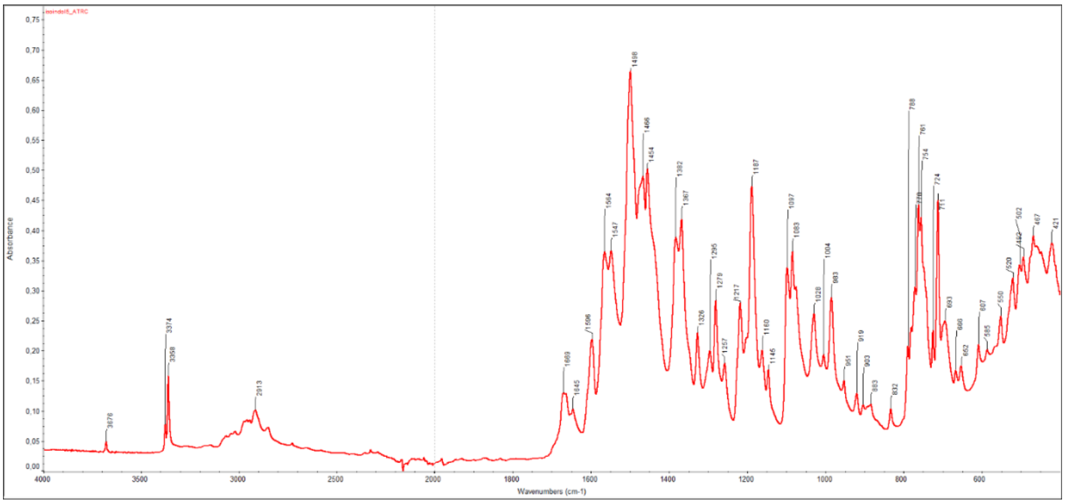

Figure S128. IR spectrum for compound **5aa** measured in solid state**.**

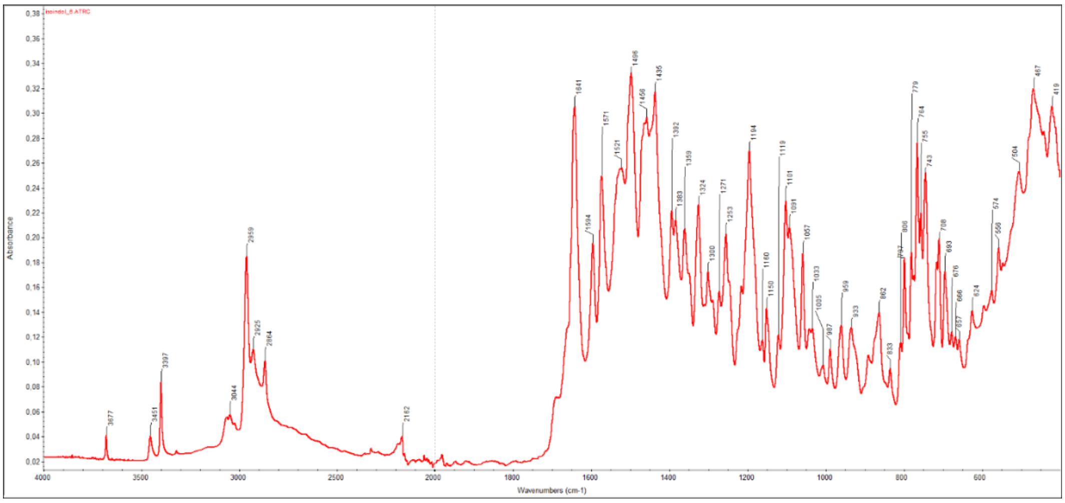

Figure S129. IR spectrum for compound **5ab** measured in solid state**.**

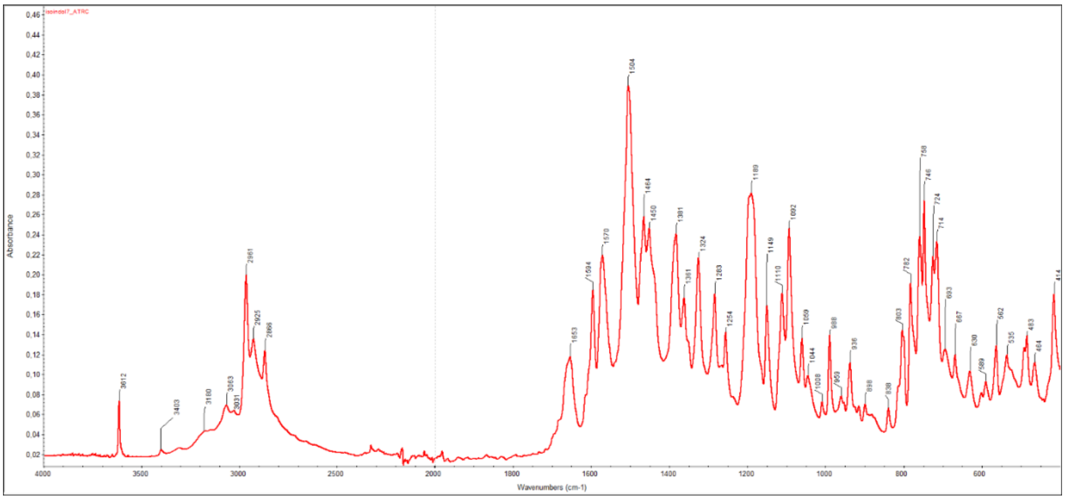

Figure S130. IR spectrum for compound **5bb** measured in solid state**.**

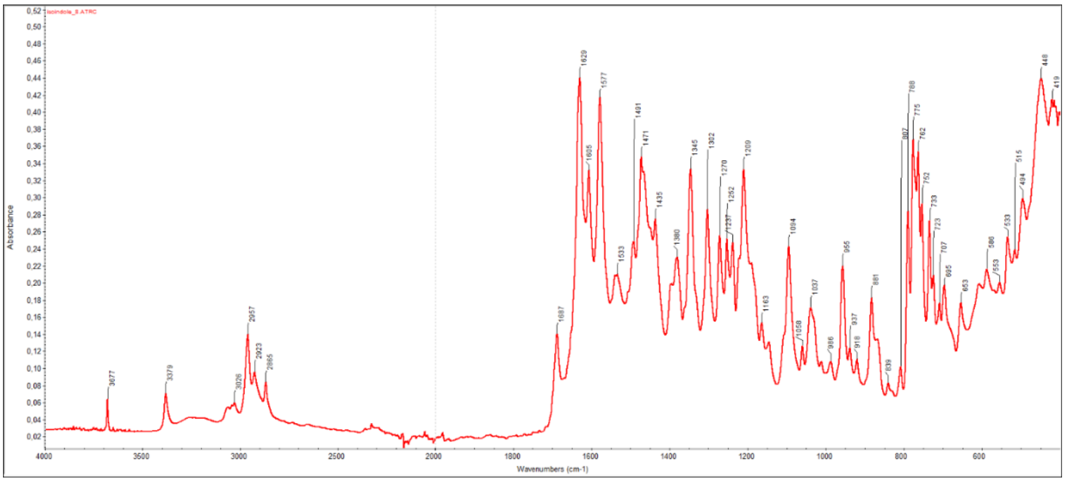

Figure S131. IR spectrum for compound **5ba** measured in solid state**.**

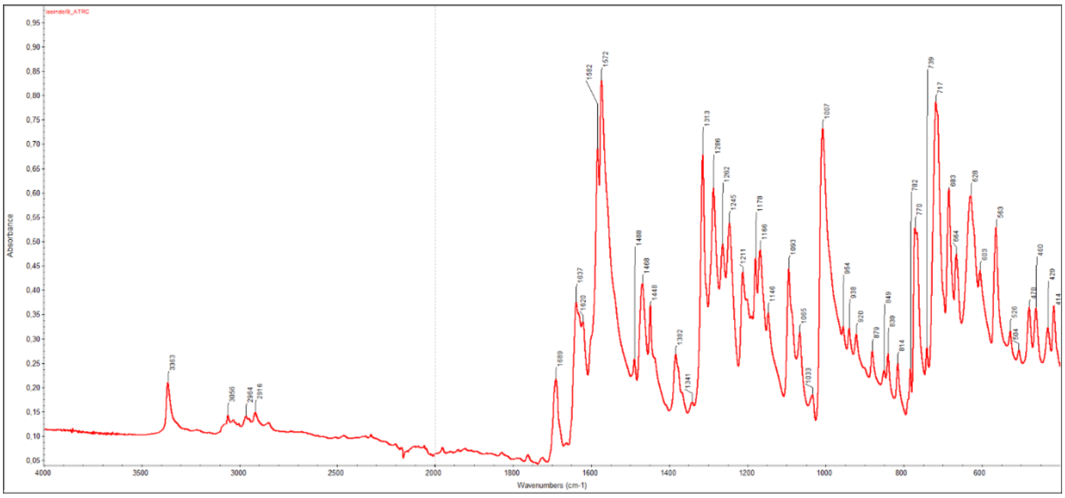

Figure S132. IR spectrum for compound **6** measured in solid state**.**

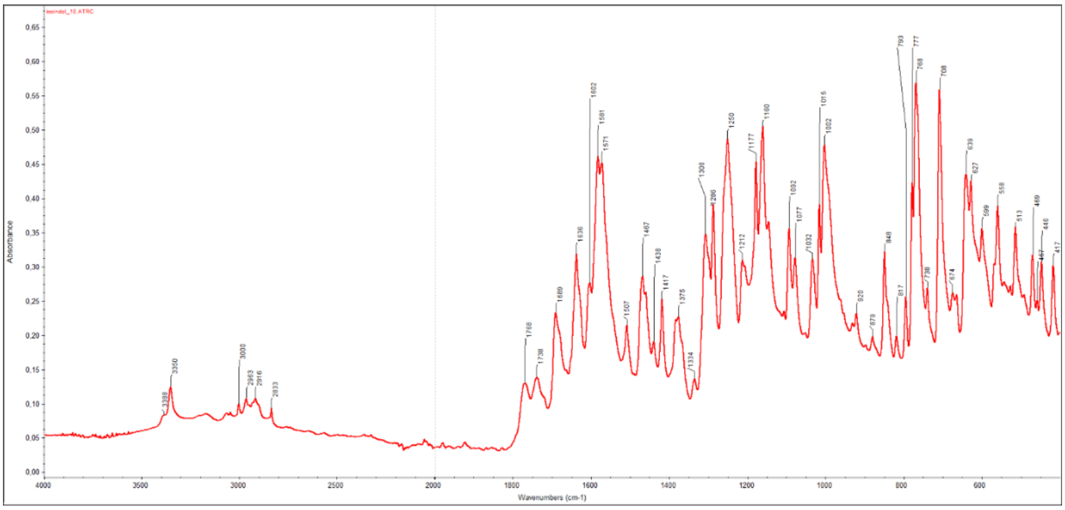

Figure S133. IR spectrum for compound **7** measured in solid state**.**

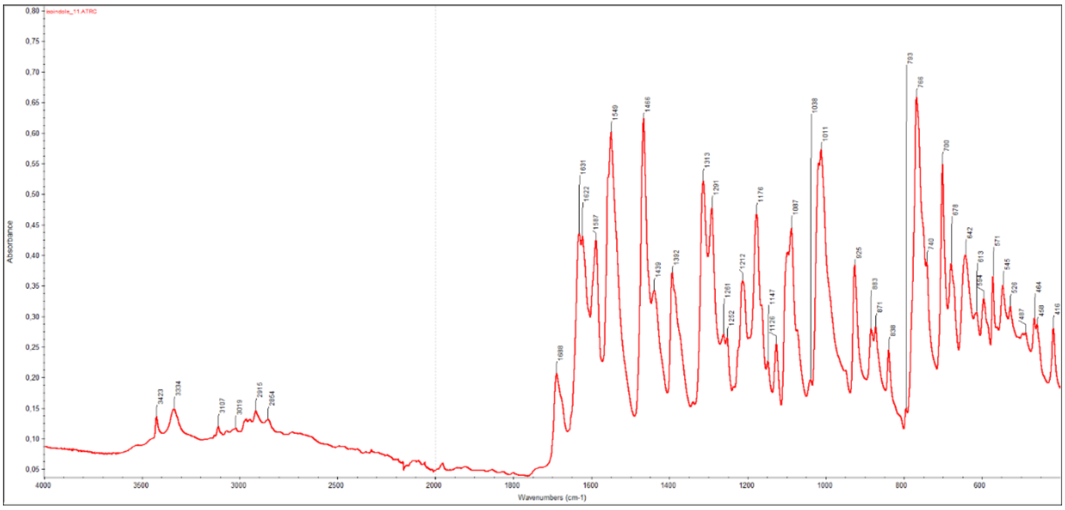

Figure S134. IR spectrum for compound **8** measured in solid state**.**

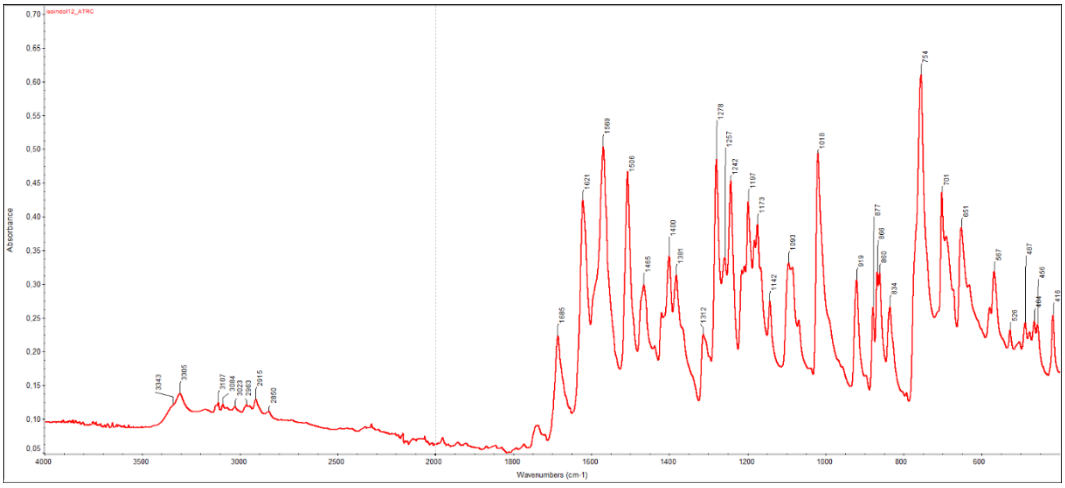

Figure S135. IR spectrum for compound **9** measured in solid state.

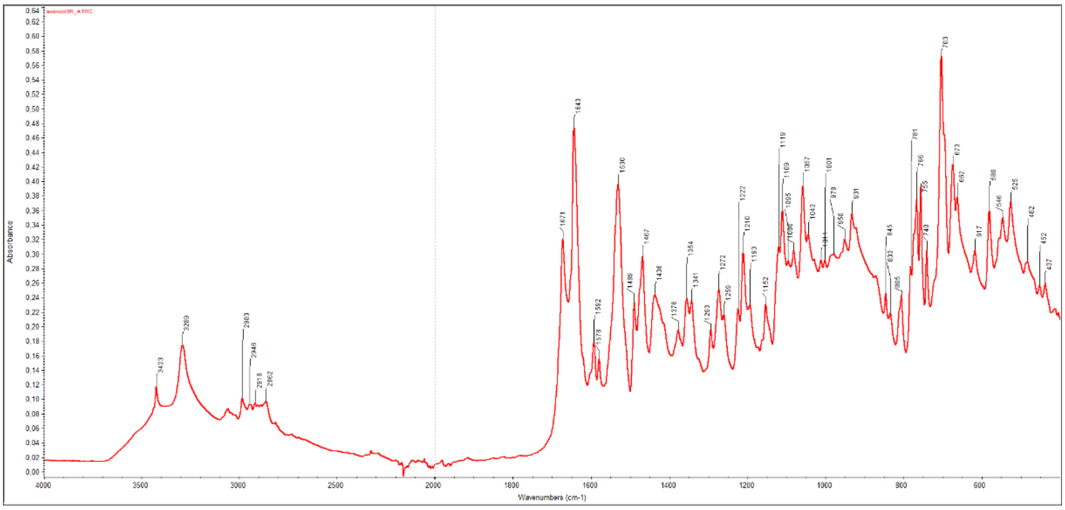

Figure S136. IR spectrum for compound **6r** measured in solid state.

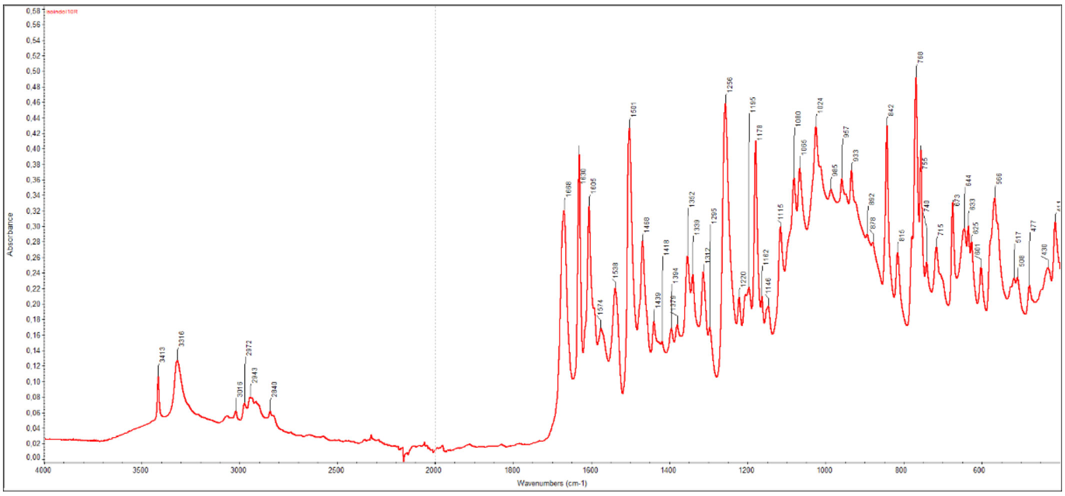

Figure S137. IR spectrum for compound **7r** measured in solid state.

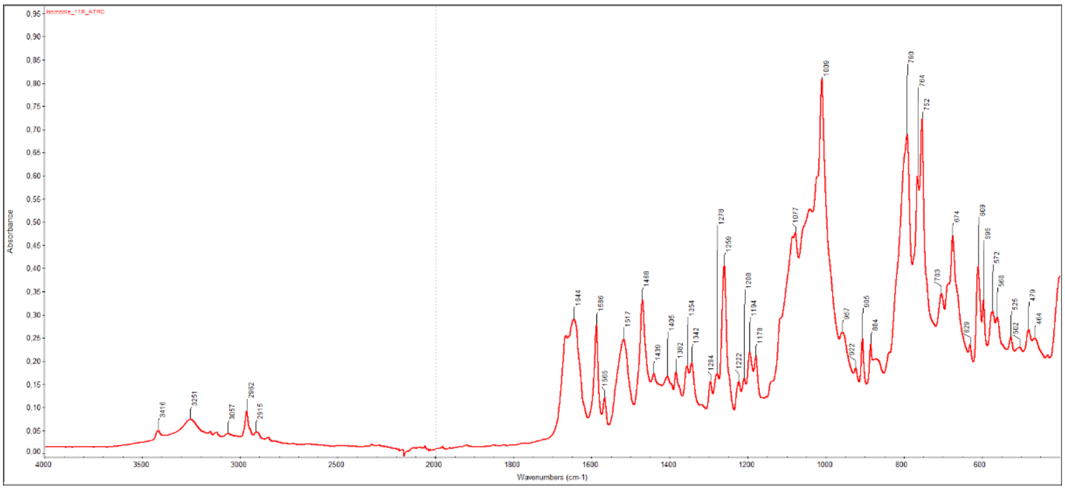

Figure S138. IR spectrum for compound **8r** measured in solid state**.**

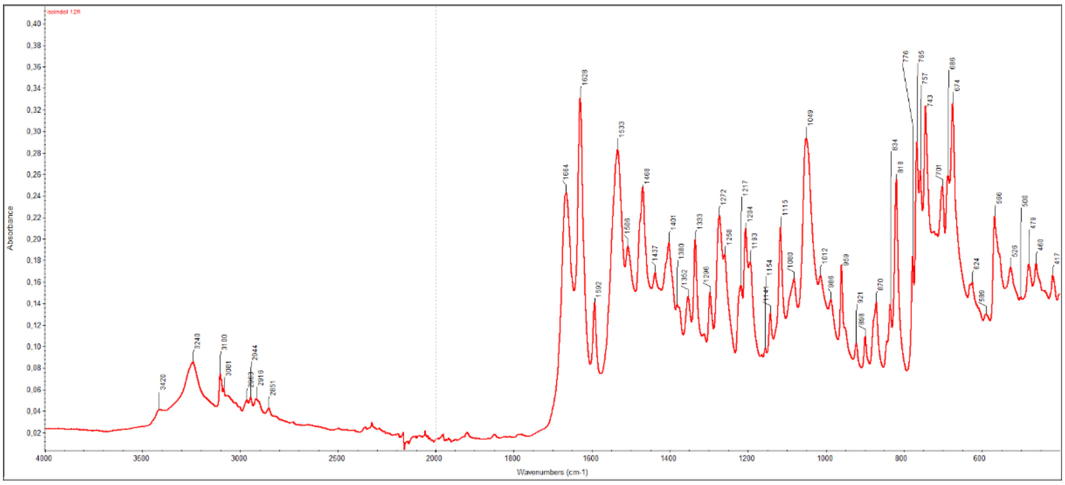

Figure S139. IR spectrum for compound **9r** measured in solid state**.**

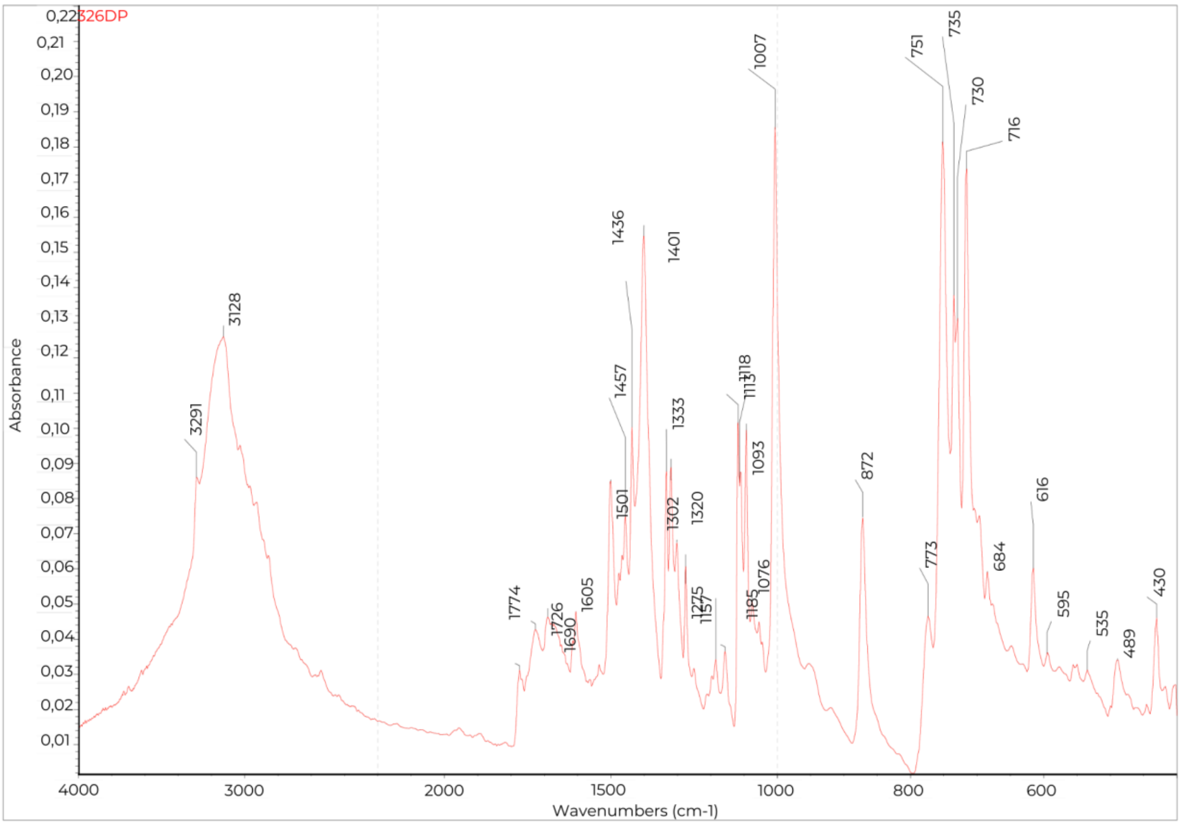

Figure S140. Example of IR spectrum for phtalocyanine **PC** measured in solid state.

10 UV-Vis spectra of prepared compounds

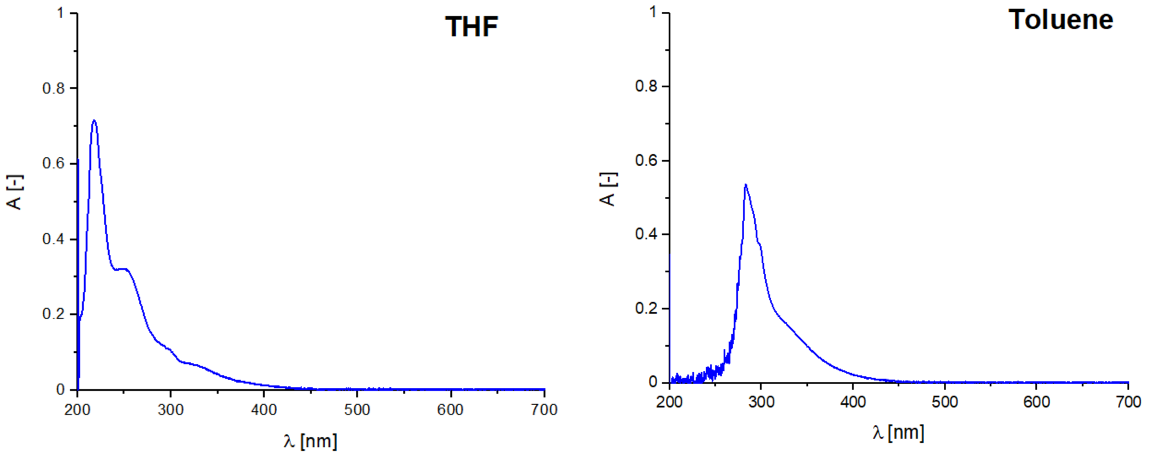

Figure S141. UV-Vis spectrum for compound **1a**.

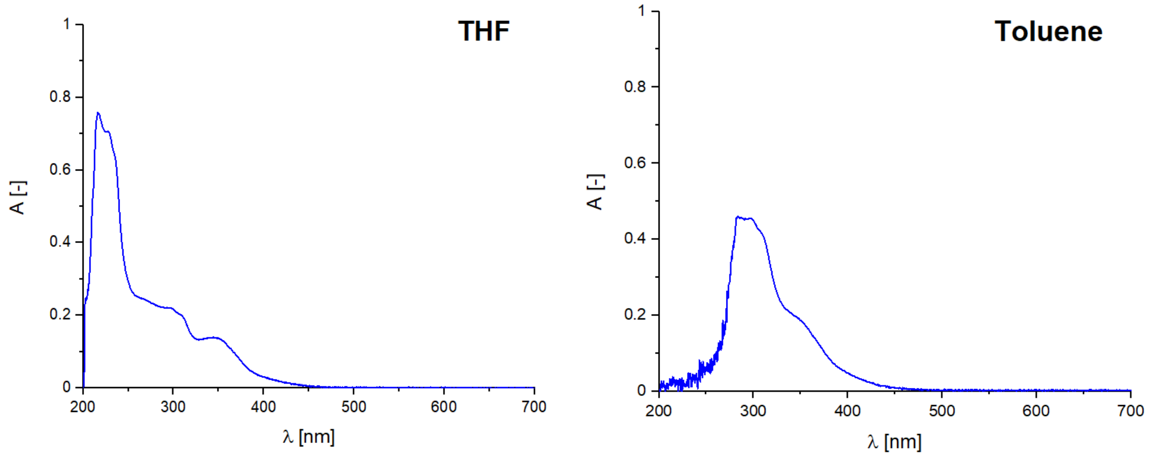

Figure S142. UV-Vis spectrum for compound **5aa**.

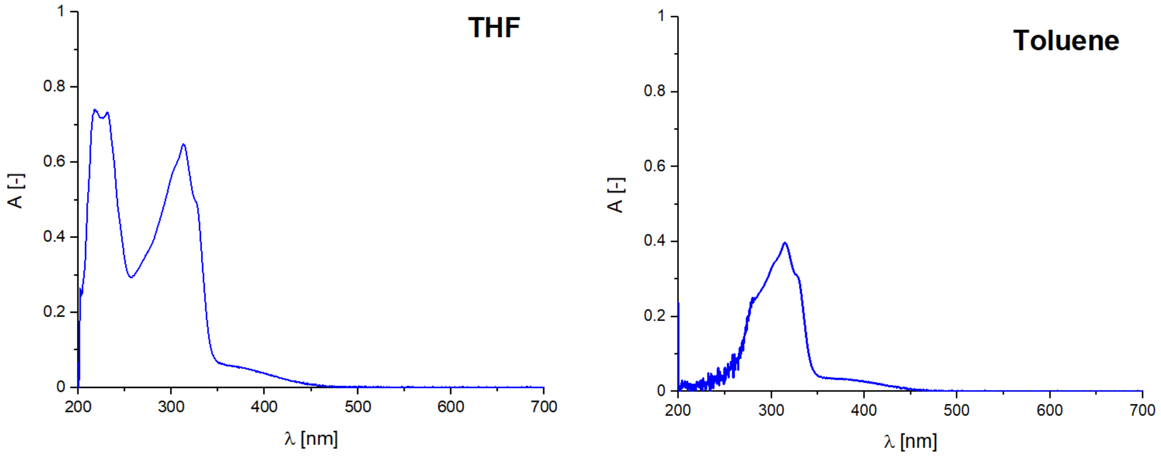

Figure S143. UV-Vis spectrum for compound **6**.

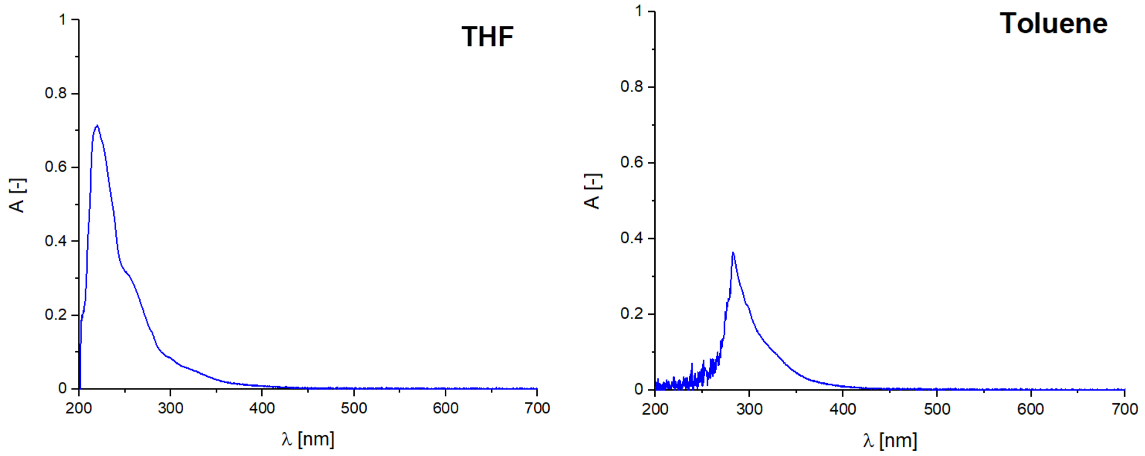

Figure S144. UV-Vis spectrum for compound **6r**.

11 Mass spectra of prepared compounds

High resolution APCI spectra in positive ion mode were measured using LTQ Orbitrap XL (Thermo Fisher Scientific) using the parameters as follows: for APCI, capillary temperature 200 °C and vaporizer temperature 375 °C. The mobile phase consisted of methanol/water (4:1), flow rate of 100 µL/min. The sample was injected using a 2-µL loop into the mobile phase flow.

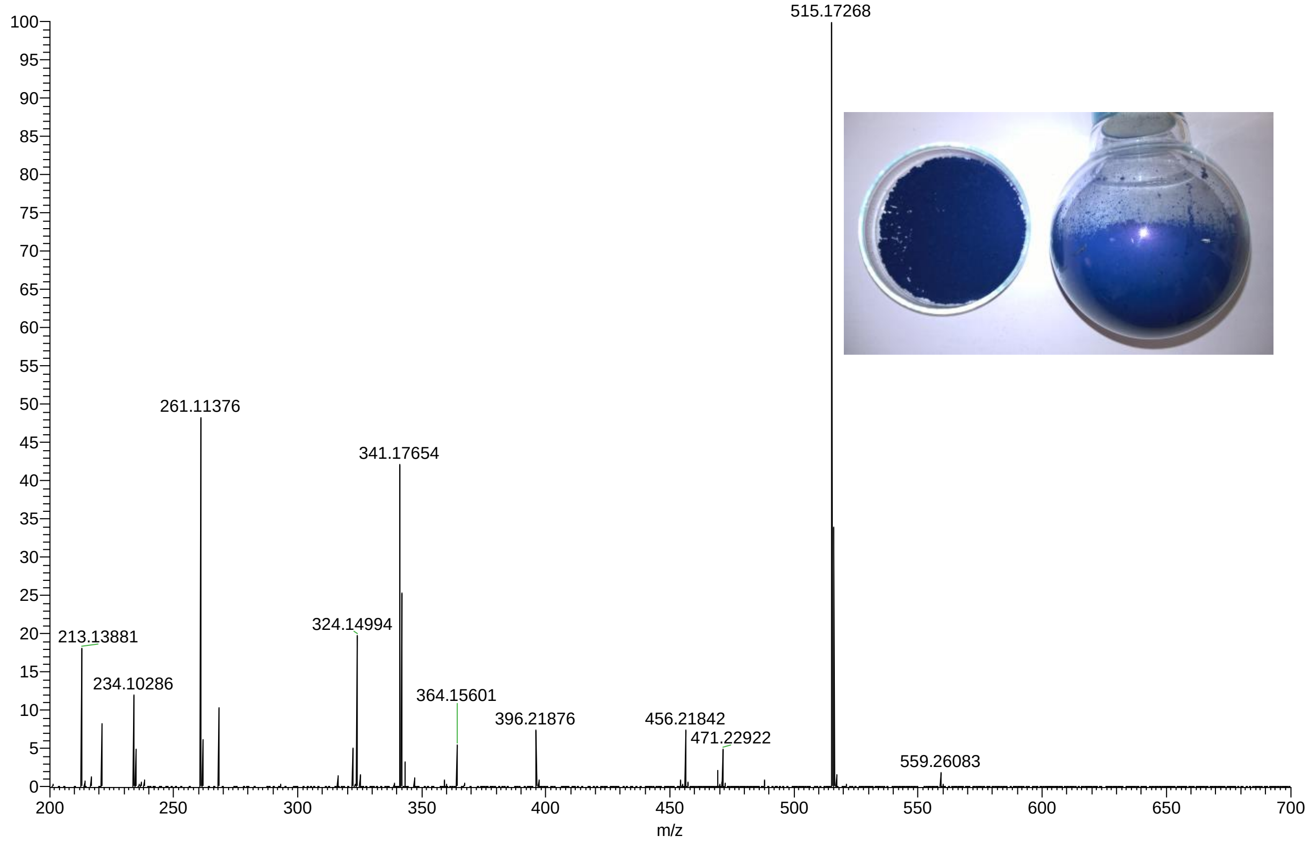

Figure S145. Mass spectrum ($APCI^+$) of **PC**: HRMS ($APCI^+$): *m/z* calculated for $C_{32}H_{19}N_8^+$ 515.17272, found 515.17268.

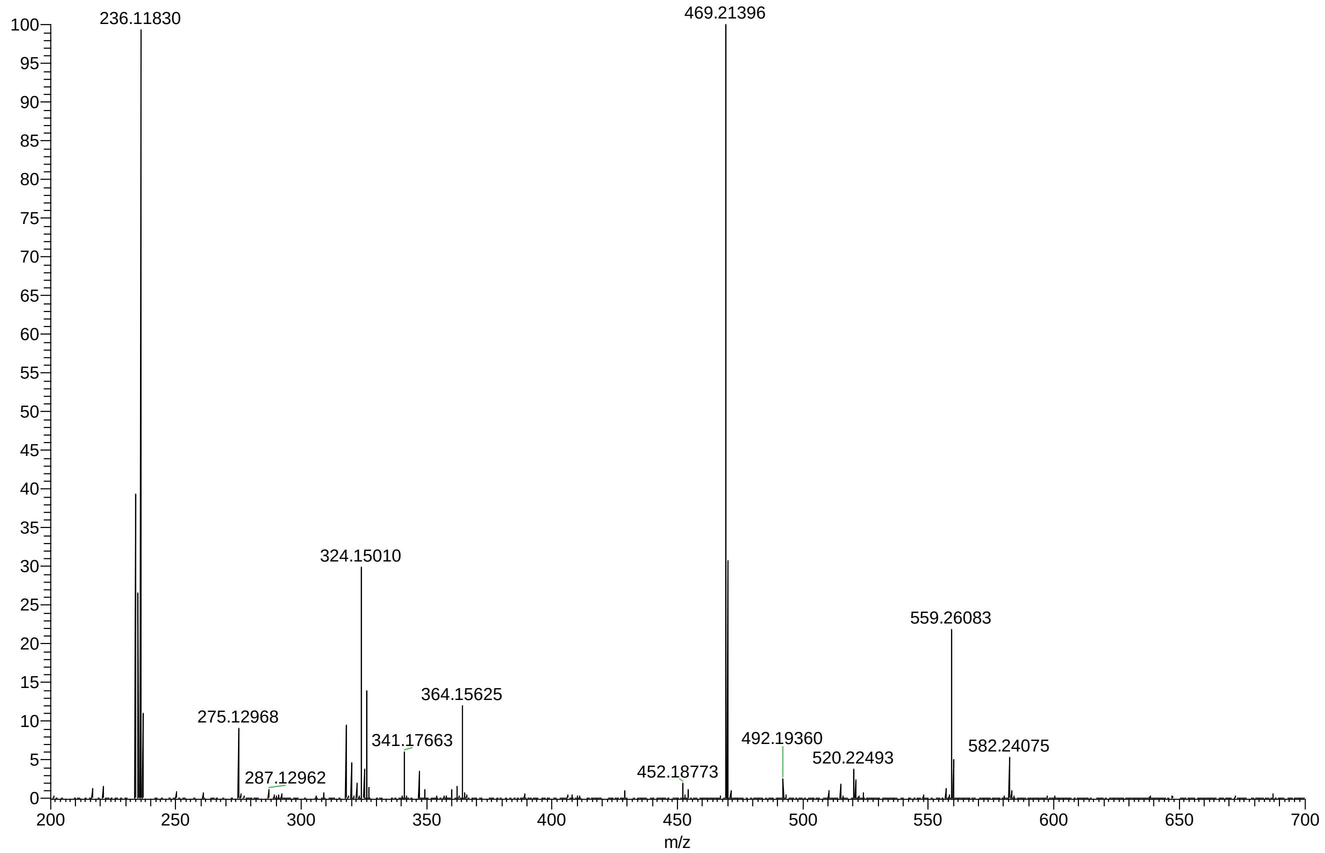

Figure S146. Mass spectrum (APCI $^+$) of **4d**: HRMS (APCI$^+$): *m/z* calculated for $C_{23}H_{18}N_5^+$ 364.15622, found 364.15625, *m/z* calculated for $C_{31}H_{22}N_7^+$ 492.19366, found 492.19360, *m/z* calculated for $C_{38}H_{28}N_7^+$ 582.24061, found 582.24075. *m/z* calculated for $C_{30}H_{25}N_6^+$ 469.21352, found 469.21396, *m/z* calculated for $C_{37}H_{31}N_6^+$ 559.26047, found 559.26083.

12 References

1. Krause, L.; Herbst-Irmer, R.; Sheldrick, G. M.; Stalke, D., Comparison of silver and molybdenum microfocus X-ray sources for single-crystal structure determination. *J. Appl. Crystallogr.* **2015,** *48*, 3-10.
2. Sheldrick, G., SHELXT - Integrated space-group and crystal-structure determination. *Acta Cryst.* **2015,** *A71*, 3-8.
3. Sheldrick, G., Crystal structure refinement with SHELXL. *Acta Cryst.* **2015,** *C71*, 3-8.
4. Bruker-AXS *APEX4 v2022.1-1*, 2022.
5. Frisch, M. J.; Trucks, G. W.; Schlegel, H. B.; Scuseria, G. E.; Robb, M. A.; Cheeseman, J. R.; Scalmani, G.; Barone, V.; Petersson, G. A.; Nakatsuji, H.; Li, X.; Caricato, M.; Marenich, A. V.; Bloino, J.; Janesko, B. G.; Gomperts, R.; Mennucci, B.; Hratchian, H. P.; Ortiz, J. V.; Izmaylov, A. F.; Sonnenberg, J. L.; Williams; Ding, F.; Lipparini, F.; Egidi, F.; Goings, J.; Peng, B.; Petrone, A.; Henderson, T.; Ranasinghe, D.; Zakrzewski, V. G.; Gao, J.; Rega, N.; Zheng, G.; Liang, W.; Hada, M.; Ehara, M.; Toyota, K.; Fukuda, R.; Hasegawa, J.; Ishida, M.; Nakajima, T.; Honda, Y.; Kitao, O.; Nakai, H.; Vreven, T.; Throssell, K.; Montgomery Jr., J. A.; Peralta, J. E.; Ogliaro, F.; Bearpark, M. J.; Heyd, J. J.; Brothers, E. N.; Kudin, K. N.; Staroverov, V. N.; Keith, T. A.; Kobayashi, R.; Normand, J.; Raghavachari, K.; Rendell, A. P.; Burant, J. C.; Iyengar, S. S.; Tomasi, J.; Cossi, M.; Millam, J. M.; Klene, M.; Adamo, C.; Cammi, R.; Ochterski, J. W.; Martin, R. L.; Morokuma, K.; Farkas, O.; Foresman, J. B.; Fox, D. J. *Gaussian 16 Rev. C.01*, Wallingford, CT, 2016.
6. Becke, A. D. Density-Functional Thermochemistry. Iii. The Role Of Exact Exchange. *The Journal of Chemical Physics* **1993**, *98* (7), 5648-5652. https://doi.org/10.1063/1.464913.
7. Lee, C.; Yang, W.; Parr, R. G. Development Of The Colle-Salvetti Correlation-Energy Formula Into A Functional Of The Electron Density. *Phys. Rev.* **1988**, *B37*, 785-789. https://doi.org/10.1103/PhysRevB.37.785.
8. Barone, V.; Cossi, M. Quantum Calculation Of Molecular Energies And Energy Gradients In Solution By A Conductor Solvent Model. *J. Phys. Chem.* **1998**, *A102*, 1995-2001. https://doi.org/10.1021/jp9716997.
9. Schlegel, H. B. Optimization Of Equilibrium Geometries And Transition Structures. *J. Comput. Chem.* **1982**, *3*, 214-218. https://doi.org/10.1002/jcc.540030212.
10. Cossi, M.; Rega, N.; Scalmani, G.; Barone, V. Energies, Structures, And Electronic Properties Of Molecules In Solution With The C-Pcm Solvation Model. *J. Comput. Chem.* **2003**, *24*, 669-681. https://doi.org/10.1002/jcc.10189.
11. Peng, C.; Ayala, P.; Shlegel, H.; Frisch, M. Using Redundant Internal Coordinates To Optimize Equilibrium Geometries And Transition States. *J. Comput. Chem* **1996**, *17*, 49-56.
12. Peng, C.; Bernhard Schlegel, H. Combining Synchronous Transit And Quasi-Newton Methods To Find Transition States. *Isr. J. Chem.* **1993**, *33*, 449-454. https://doi.org/10.1002/ijch.199300051.
13. Grimme, S.; Antony, J.; Ehrlich, S.; Krieg, H. A Consistent And Accurate Ab Initio Parametrization Of Density Functional Dispersion Correction (Dft-D) For The 94 Elements H-Pu. *J. Chem. Phys.* **2010**, *132,* 154104. https://doi.org/10.1063/1.3382344.
14. a) Spiessens, L. I.; Anteunis, M. J. O. Nmr Studies On Imidines. V. ^{1}H And ^{13}C Nuclear Magnetic Resonance Study Of The Tautomerism And Geometrical Isomerism Of 1,3-Bis(Arylimino)Isoindolines. *Bull. Soc. Chim. Belg.* **1984**, *93* (3), 205-222. https://doi.org/10.1002/bscb.19840930306. b) Spiessens, L. I.; Anteunis, M. J. O. Nmr Studies On Imidines. Vii. The Tautomerism Of Mono-N-Aryl Substituted Phthalic Imidines. A 1 H And 13 C Nuclear Magnetic Resonance Study. *Bull. Soc. Chim. Belg.* **1988**, *97* (6), 431-452. https://doi.org/10.1002/bscb.19880970604. c) Siegl, W.O. A new bis-cheiating ligand system. Synthesis and

chelating behavior. *Inorg. Chim. Acta* **1977**, 25, L65–L66. d) Tamgho, I. -S.; Engle, J. T.; Ziegler, C. J. The Syntheses And Structures Of Bis(Alkylimino)Isoindolines. *Tetrahedron Lett.* **2013**, *54* (45), 6114-6117.

15. Neufeld, R.; Stalke, D. Accurate Molecular Weight Determination Of Small Molecules Via Dosy-NMR By Using External Calibration Curves With Normalized Diffusion Coefficients. Chemical Science **2015**, *6*, 3354-3364. https://doi.org/10.1039/C5SC00670H.
